# Biocatalytic Conversion of Cyclic Ketones Bearing α‐Quaternary Stereocenters into Lactones in an Enantioselective Radical Approach to Medium‐Sized Carbocycles

**DOI:** 10.1002/anie.201800121

**Published:** 2018-03-05

**Authors:** Charlotte Morrill, Chantel Jensen, Xavier Just‐Baringo, Gideon Grogan, Nicholas J. Turner, David J. Procter

**Affiliations:** ^1^ School of Chemistry University of Manchester Manchester M13 9PL UK; ^2^ Department of Chemistry University of York, Heslington York YO10 5DD UK

**Keywords:** biocatalysis, cyclization, lactones, radicals, samarium

## Abstract

Cyclic ketones bearing α‐quaternary stereocenters underwent efficient kinetic resolution using cyclohexanone monooxygenase (CHMO) from *Acinetobacter calcoaceticus*. Lactones possessing tetrasubstituted stereocenters were obtained with high enantioselectivity (up to >99 % *ee*) and complete chemoselectivity. Preparative‐scale biotransformations were exploited in conjunction with a SmI_2_‐mediated cyclization process to access complex, enantiomerically enriched cycloheptan‐ and cycloctan‐1,4‐diols. In a parallel approach to structurally distinct products, enantiomerically enriched ketones from the resolution with an α‐quaternary stereocenter were used in a SmI_2_‐mediated cyclization process to give cyclobutanol products (up to >99 % *ee*).

The Baeyer–Villiger (BV) reaction^1^ transforms ketones into esters or lactones with predictable regioselectivity and is therefore a valuable tool for synthesis. Metal‐based catalysts^2^ and organocatalysts^3^ have been developed for catalytic enantioselective variants of the BV reaction; however, high enantioselectivity is generally limited to the transformation of activated cyclobutanone substrates, in which the release of ring strain drives the BV reaction.^4^ Baeyer–Villiger monooxygenases (BVMOs) offer an attractive alternative to the use of chemical reagents for enantioselective BV reactions. These flavin‐dependent enzymes exploit atmospheric oxygen and catalyze the transformation via an enzyme‐bound (hydro)peroxyflavin intermediate.^5^ The use of BVMOs promises distinct advantages in terms of enantioselectivity, substrate scope, and chemoselectivity not possible using chemical reagents.^6^

BV transformations of α,α‐dialkyl cyclic ketones have the potential to deliver enantiomerically enriched lactones with tetrasubstituted stereocenters and cyclic ketones bearing α‐quaternary centers. Such products possess hard‐to‐build stereocenters^7^ and are high‐value chiral building blocks for synthesis.^8^ To our knowledge, there are no reports of the enantioselective BV reaction of α,α‐dialkyl cyclic ketones using chemical reagents.2b,2g Furthermore, the ability of BVMOs to catalyze the kinetic resolution of α,α‐dialkyl cyclic ketones was, until now, unexplored (Scheme 1 A).^9^ The synthetic reach of biocatalytic reactions is greatly extended when enzymatic processes are integrated into synthetic sequences involving chemical catalysts and reagents.^10^ Herein, we describe a highly enantioselective and chemoselective, biocatalytic Baeyer–Villiger approach to unsaturated lactones **II** bearing tetrasubstituted stereocenters that proceeds by the kinetic resolution of cyclic ketones **I** bearing α‐quaternary stereocenters. Crucially, lactones **II** are not readily accessible in enantiomerically enriched form by state‐of‐the‐art chemical methods.^11^ Lactones **II** are excellent substrates for SmI_2_–H_2_O‐mediated^12^ cyclization reactions, which complete an enantioselective biocatalytic–chemical approach to important and complex cycloheptan‐ and cycloctan‐1,4‐diols **III** (Scheme 1 B): structural motifs that are notoriously hard to prepare and that are prevalent in biologically relevant targets (Scheme 1 B,C).^13^

**Scheme 1:**
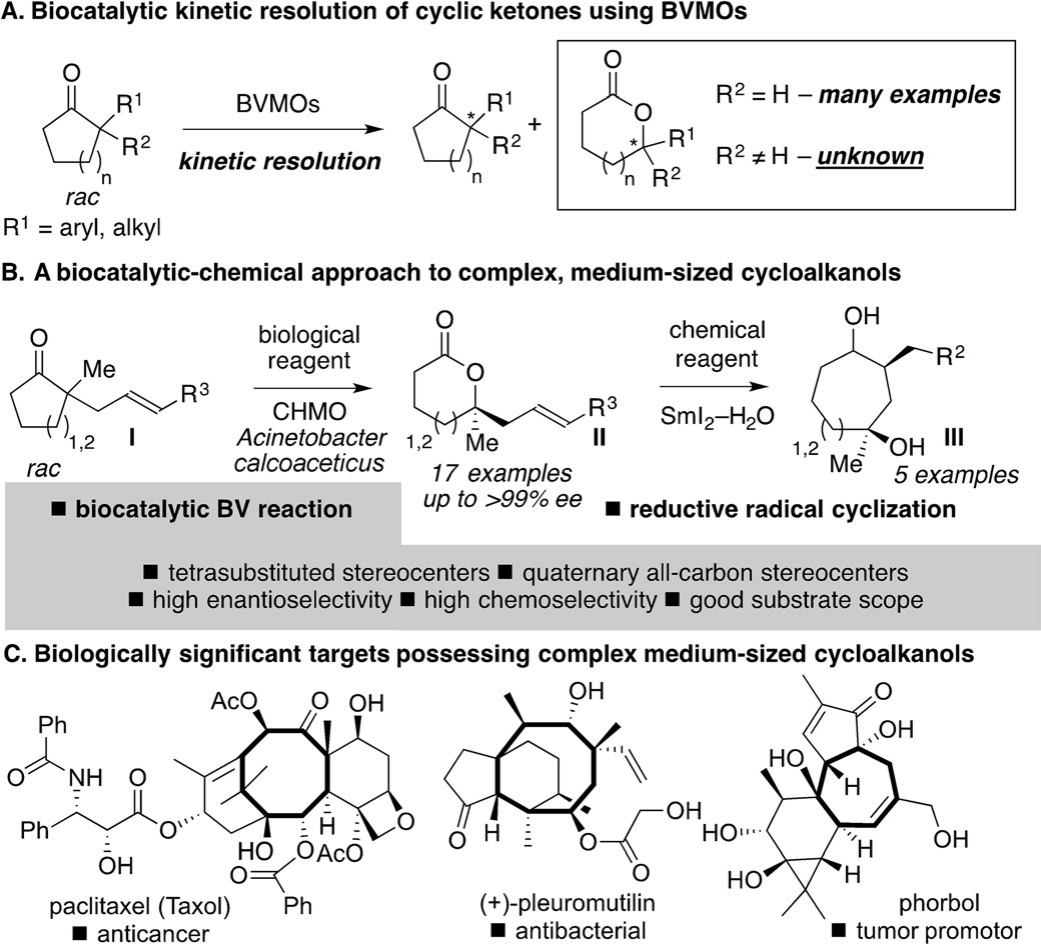
A. BVMOs in the kinetic resolution of cyclic ketones: lack of precedent for the resolution of substrates bearing α‐quaternary stereocenters. B. An approach to complex, medium‐sized cycloalkanols that exploits the synergy between a biocatalytic and a chemical process. C. The biological importance of molecules containing cycloheptanol and cyclooctanol motifs.

The biocatalytic kinetic resolution was explored using the purified CHMO_*Actineto*_ enzyme from *Acinetobacter calcoaceticus* (NCIMB 9871). A glucose/glucose dehydrogenase (GDH) recycling system was employed for catalytic regeneration of the NADPH cofactor,^14^ and the feasibility of the biotransformation was initially assessed on an analytical scale using ketone **1 a** (R^1^=Me, R^2^=H). Pleasingly, despite the lack of precedent for the resolution of substrates bearing α‐quaternary centers, lactone **2 a** was efficiently formed with >99 % *ee* at a conversion of 50 % (Table 1). A control reaction, in which **1 a** was exposed to the reaction conditions in the absence of CHMO, resulted in no conversion into the product. To assess the scope of the transformation, we prepared a range of lactones bearing various aryl groups on the alkene unit in one straightforward step from **1 a** (see the Supporting Information). Despite the presence of the bulky aryl substituents and the α‐quaternary center, we were pleased to observe unprecedented tolerance on the part of CHMO, and all six‐membered ketones **1** were transformed into seven‐membered lactones **2** with very high enantioselectivity (selectivity factors, *E*>200; Table 1).

**Table 1:** Biotransformations of six‐membered cyclic ketones bearing α‐quaternary stereocenters catalyzed by CHMO_*Acineto*_. 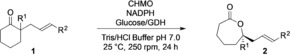

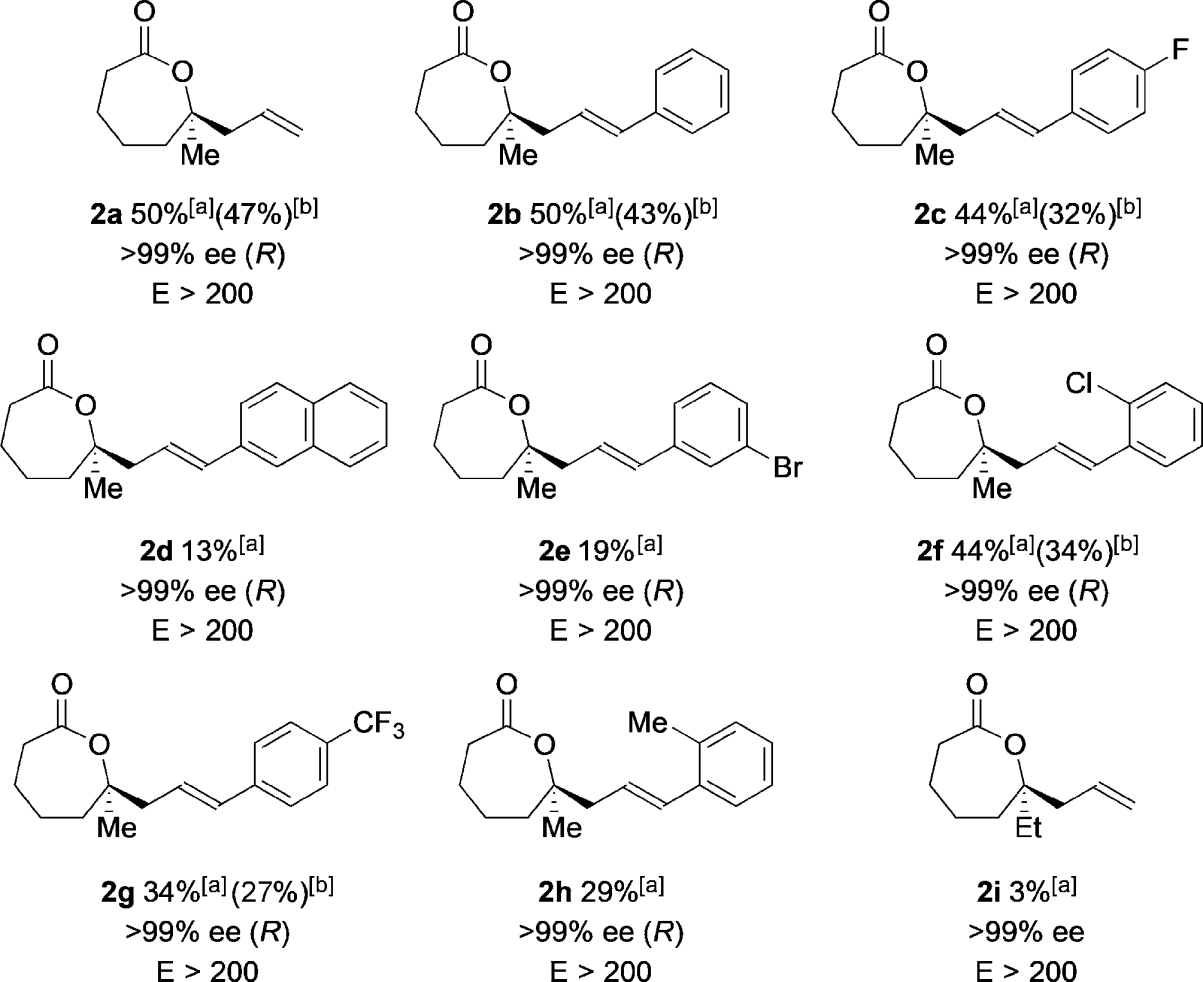

Reaction conditions for analytical‐scale biotransformations: Ketone (1 mg mL^−1^), CHMO (0.25 mg mL^−1^), NADPH (0.7 mm), GDH (0.25 mg mL^−1^), glucose (5.5 mm), Tris/HCl buffer (100 mm). [a] Conversion determined by GC or ^1^H NMR analysis of the crude reaction mixture. [b] The yield of the isolated product is shown in brackets for a preparative‐scale transformation. For a detailed description of the procedure for the preparative‐scale biotransformations, see the Supporting Information; *ee* values were determined by chiral‐stationary‐phase GC or HPLC analysis.

Selective formation of the *R* enantiomer of the lactone products was confirmed for **2 a**–**c** and inferred for the remainder.^15^ For substrates bearing a methyl substituent at the α‐quaternary center, conversions up to 50 % (the maximum theoretical value for an ideal kinetic resolution) were observed. In stark contrast to chemical oxidation of **1 b** to **2 b**, no side products resulting from competing oxidation of the alkene in the starting material and product were observed.^16^ Thus, the biocatalytic process exhibited complete chemoselectivity. Substitution was tolerated at all positions on the aromatic ring, with halogen, methyl, and trifluoromethyl substituents all accepted by the enzyme active site. Substrates **1 d** and **1 e**, bearing bulky 2‐naphthyl and 3‐bromophenyl substituents, were transformed less efficiently by the enzyme, and lower conversion was observed; however, enantioselectivity remained high. Variation of the pH value of the reaction mixture and the use of an alternative biocatalyst, cyclododecanone monooxygenase (CDMO_*Rhodo*_) from *Rhodococcus*, did not lead to improved conversion.^17^ Ketone **1 i**, in which the R^1^ group was an ethyl rather than a methyl substituent, was transformed inefficiently, although a small amount of product was formed with high enantiocontrol. Following assessment of the process on an analytical scale, transformations were performed on a preparative scale (0.2 mmol; formation of **2 a**–**c**, **2 f**). Facile separation of the product from the ketone afforded pure samples of the enantiomerically enriched lactone products. For substrates **1 a**–**c**, **1 f**, high‐value ketones bearing α‐quaternary stereocenters were obtained in high enantiomeric purity after the kinetic resolution (see below).

We also explored the scope of the biocatalytic kinetic resolution with respect to the formation of six‐membered lactones. A range of five‐membered‐ring ketones **3** was prepared and subjected to the CHMO‐catalyzed transformation (Table 2). Although the biotransformations were typically less efficient than those involving six‐membered cyclic ketones, in some cases lactone products **4** were formed with selectivity factors (*E*=17–59) indicative of useful synthetic procedures. For example, cyclopentanones **3 c** and **3 h** underwent significant conversion, and lactones **4 c** and **4 h** were obtained in 96 and 88 % *ee*, respectively. Five‐membered cyclic ketones **3 d**, **3 e**, and **3 g** containing larger aromatic substituents showed very little or no conversion into lactone products (Table 2). Selective formation of the *R* enantiomer of the lactone products was confirmed for **4 a**–**c** and inferred for the remainder.^15^ Analogous ketone substrates with a seven‐membered ring were incompatible with the biocatalytic process.^15^

**Table 2:** Biotransformations of five‐membered cyclic ketones bearing α‐quaternary stereocenters catalyzed by CHMO_*Acineto*_. 
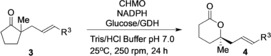

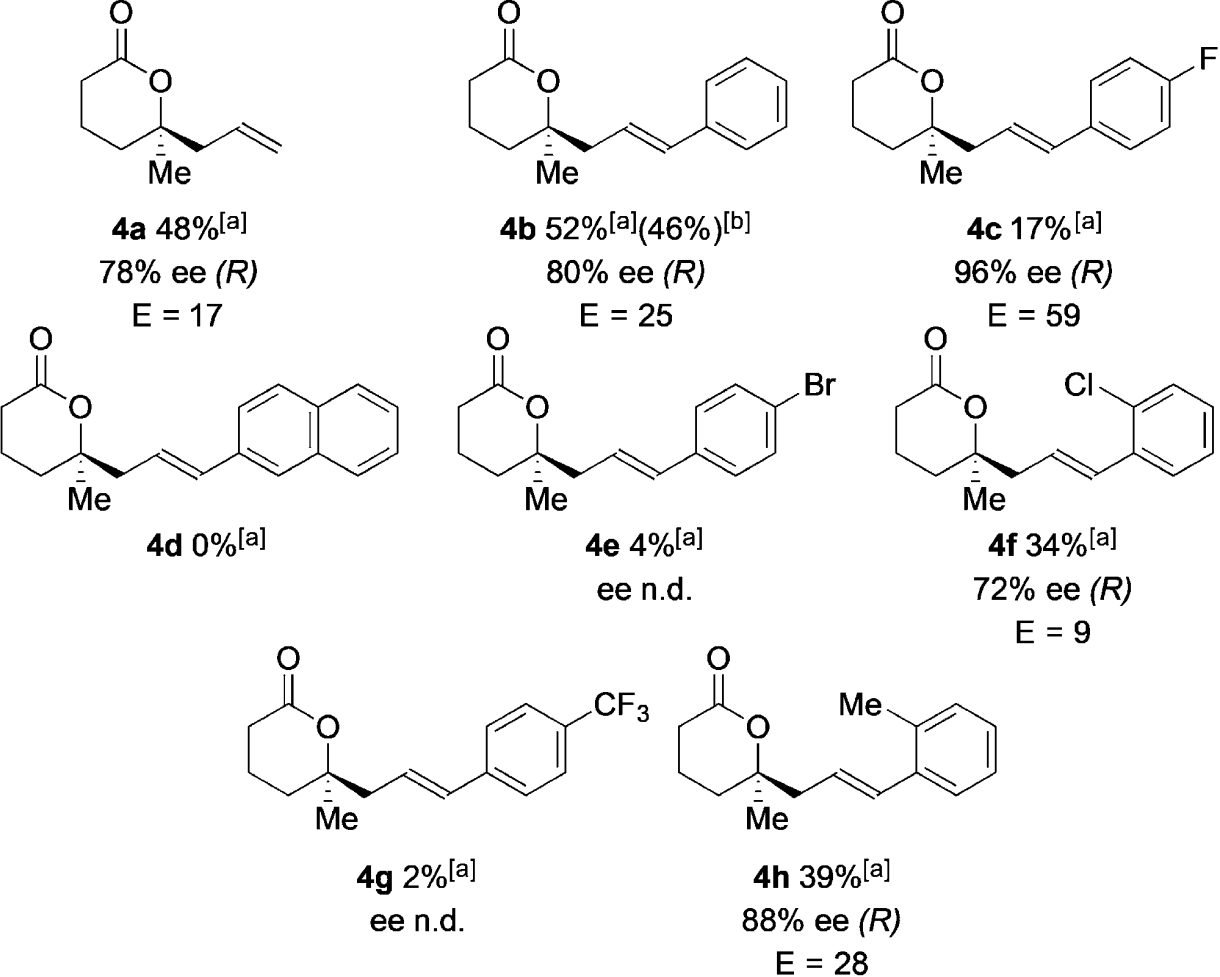

Reaction conditions for analytical‐scale biotransformations: Ketone (1 mg mL^−1^), CHMO (0.25 mg mL^−1^), NADPH (0.7 mm), GDH (0.25 mg mL^−1^), glucose (5.5 mm), Tris/HCl buffer (100 mm). [a] Conversion determined by GC or ^1^H NMR analysis of the crude reaction mixture. [b] The yield of the isolated product is shown in brackets for a preparative‐scale transformation. For a detailed description of the procedure for the preparative‐scale biotransformations, see the Supporting Information; *ee* values were determined by chiral‐stationary‐phase GC or HPLC analysis.

Berghuis and co‐workers have previously determined the structure of CHMO_*Rhodo*_, a homologue of CHMO_*Actineto*_, in its “tight” conformation in complex with ϵ‐caprolactone, the product of BV oxidation of cyclohexanone (PDB code 4RG3),^18^ and suggest that the stereoselectivity of the reaction is determined in this conformation of the enzyme. CHMO_*Acineto*_ used in this study shares 55 % amino acid sequence identity with CHMO_*Rhodo*_, and the majority of residues within the active site are conserved, thus allowing the construction of a model of CHMO_*Acineto*_ such as that previously presented by Reetz and co‐workers.^19^ The model was used as the input for an in silico docking experiment with the enantiomers of lactone product **2 a**. Figure 1 shows the *R* enantiomer of **2 a**, the preferred product of the reaction, beneath the FAD coenzyme with its alkenyl substituent accommodated in the hydrophobic pocket formed by the side chains of L435, F505, and F432, the latter having been shown through mutation to have a profound influence on the enantioselectivity of CHMO_*Acineto*_‐catalyzed reactions.^20^ The pose would also permit the accommodation of larger side chains, such as those in the *R* lactone products **2 b** and **2 c**. The role of this region in the accommodation of bulky side chains, such as that of 2‐phenyl cyclohexanone, has previously been demonstrated.^19^ Interestingly, the **2 a** lactone carbonyl group in the top pose superimposes well with the equivalent atom in the 4RG3–ϵ‐caprolactone complex, at a distance of 3.3 Å from the ribose 1‐hydroxy group and 2.7 Å from the side chain of R327, which is thought to stabilize the oxyanion in the Criegee intermediate in the same position.

**Figure 1:**
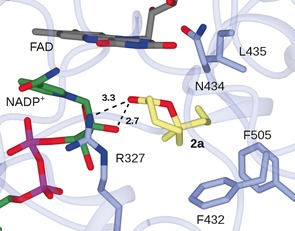
Model of CHMO_*Acineto*_ in complex with lactone product (*R*)‐**2 a** created using Autodock‐Vina.^21^ Backbone and side chains of the enzyme are shown in light blue; carbon atoms of FAD, NADP^+^, and **2 a** are shown in gray, green, and yellow, respectively. Selected interactions are indicated by black dashed lines with distance in angstroms.

Radical approaches to challenging medium‐sized carbocycles are highly prized.^22^ In particular, radical cyclization reactions to give cyclooctanes are scarce and are mostly limited to 8‐*endo* cyclization modes. We previously reported a 5‐*exo*‐trig radical cyclization approach that converts unsaturated six‐ and seven‐membered lactones into complex seven‐ and eight‐membered cycloalkanols; however, until now the process could only deliver racemic products, as enantiomerically enriched starting lactones could not readily be prepared.12g,12j Achieving enantiomeric control in SmI_2_‐mediated reactions of achiral or racemic substrates is challenging,^23^ and the use of enantiomerically enriched substrates in diastereoselective processes is a more general approach to access enantiomerically enriched products.

The enantiomerically enriched lactones **2**/**4** formed by the biocatalytic Baeyer–Villiger reaction were excellent substrates for diastereoselective radical cyclization reactions mediated by SmI_2_–H_2_O. Upon treatment with SmI_2_–H_2_O, lactones **2**/**4** underwent efficient 5‐*exo*‐trig radical cyclization to give seven‐ and eight‐membered cycloalkanols **5** in good yield. Oxidation of the crude product mixtures simplified the diastereoisomeric mixtures and afforded the cycloalkanoid products **6** with up to 6:1 dr. The reaction proceeded with no loss of enantiomeric enrichment, and important cyclooctanol motifs were obtained with >99 % *ee*.

The radical cyclization proceeds by electron transfer from Sm^II^ to the carbonyl group of the enantiomerically enriched lactones formed by the biocatalytic BV reaction. The resultant radical anions **IV** (see Table 3) undergo intramolecular addition to the alkene acceptor to give hemiketal intermediates that are reduced in situ by SmI_2_–H_2_O to give enantiomerically enriched seven‐ and eight‐membered cycloalkanol products **5** in good yield.

**Table 3:** Radical cyclization of enantiomerically enriched lactones using SmI_2_–H_2_O completes an enantioselective approach to cycloheptanols and cyclooctanols. 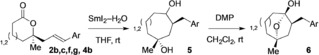

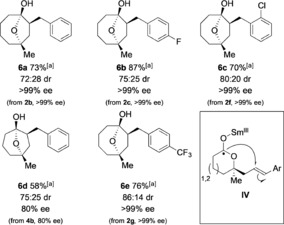

Reaction conditions: Lactone (1 equiv), SmI_2_ (8 equiv), H_2_O (800 equiv). Diastereoisomeric ratios were determined from the ^1^H NMR spectrum of the crude reaction mixture; *ee* values were determined by chiral‐stationary‐phase GC or HPLC analysis. [a] Yield of the isolated product after 2 steps. DMP=Dess–Martin periodinane.

The biocatalytic transformation of α,α‐dialkyl cyclic ketones delivers not only enantiomerically enriched lactones **2** but also enantiomerically enriched cyclic ketones **1** recovered from the kinetic resolution (Scheme 2). For example, the biocatalytic transformation to give lactone **2 b** (43 % isolated yield, >99 % *ee*) also gave ketone **1 b** (39 % isolated yield, >99 % *ee*) bearing an α‐quaternary stereocenter. Pleasingly, SmI_2_‐mediated radical cyclization of **2 b** gave enantiomerically pure cyclobutanol **7 a** with complete diastereocontrol in 84 % yield.^24^ Cyclobutanols **7 b** and **7 c** could similarly be obtained from resolved ketones bearing α‐quaternary stereocenters, **1 f** and **3 b**. Thus, biocatalytic kinetic resolution of racemic ketones **1** and **3**, when used in combination with metal‐mediated radical cyclization reactions, allows divergent access to important carbocyclic products with very different molecular architectures in enantiomerically enriched form.

**Scheme 2:**
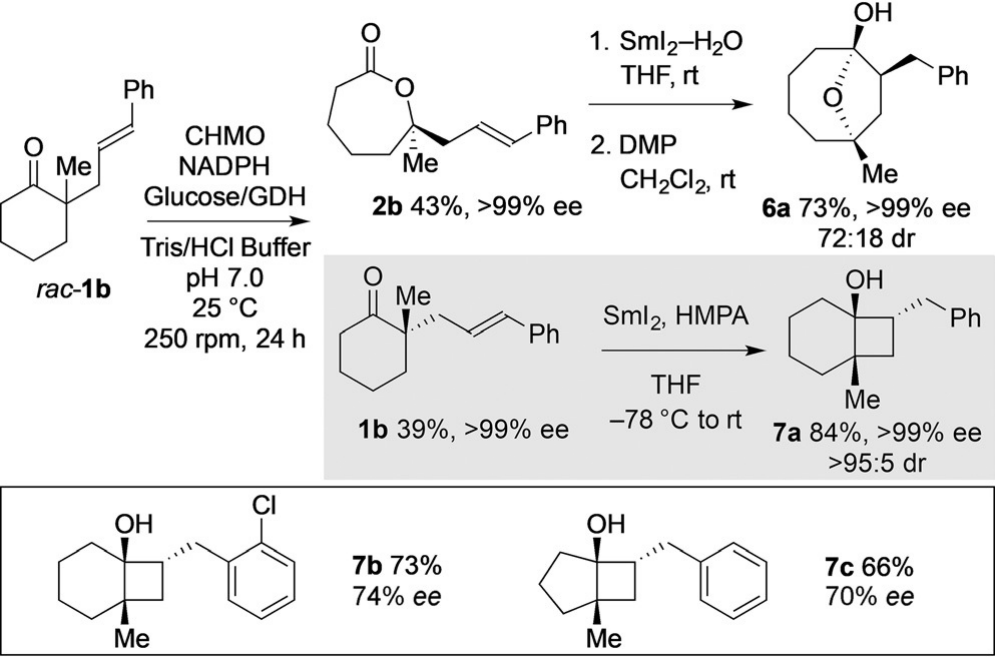
Biocatalytic kinetic resolution of *rac*‐**1 b** in a divergent, metal‐mediated radical cyclization approach to structurally distinct, enantiomerically pure molecular architectures.

In summary, racemic cyclic ketones bearing α‐quaternary stereocenters underwent efficient kinetic resolution using cyclohexanone monooxygenase (CHMO) from *Acinetobacter calcoaceticus*. The new biocatalytic process has been used in combination with new radical cyclization reactions to access important enantiomerically enriched carbocyclic scaffolds. In particular, lactones possessing tetrasubstituted stereocenters were obtained with high enantioselectivity (up to >99 % *ee*) and were exploited in SmI_2_‐mediated cyclization processes to access complex, enantiomerically enriched cycloheptan‐ and cycloctan‐1,4‐diols. In a divergent approach to structurally distinct molecular architectures, enantiomerically enriched cyclic ketones from the resolution, bearing an α‐quaternary stereocenter, were used in a SmI_2_‐mediated cyclization process to give cyclobutanol products (up to >99 % *ee*).

## Conflict of interest

The authors declare no conflict of interest.

## Supporting information

As a service to our authors and readers, this journal provides supporting information supplied by the authors. Such materials are peer reviewed and may be re‐organized for online delivery, but are not copy‐edited or typeset. Technical support issues arising from supporting information (other than missing files) should be addressed to the authors.

Supplementary
