# Supplementary material for: Biocatalytic Conversion of Cyclic Ketones Bearing α‐Quaternary Stereocenters into Lactones in an Enantioselective Radical Approach to Medium‐Sized Carbocycles
Source: Angew Chem Int Ed Engl. 2018 Mar 5;57(14):3692–6. doi: 10.1002/anie.201800121 (PMC6055628; doi:10.1002/anie.201800121)
Supplement: Supplementary file 1 — Supplementary [file ANIE-57-3692-s001.pdf]

Angewandte Chemie
Eine Zeitschrift der Gesellschaft Deutscher Chemiker

Supporting Information

# Biocatalytic Conversion of Cyclic Ketones Bearing α-Quaternary Stereocenters into Lactones in an Enantioselective Radical Approach to Medium-Sized Carbocycles

*Charlotte Morrill, Chantel Jensen, Xavier Just-Baringo, Gideon Grogan, Nicholas J. Turner,* and David J. Procter**

anie_201800121_sm_miscellaneous_information.pdf

## Table of Contents


1. General Information S1
2. Protein Production and Purification S2
3. CHMO Sequence S3
4. Modelling S3
5. Chemical Oxidation of Ketone **1b** S3
6. Experimental Section S6
    - 5.1. Preparation of Racemic Ketone Starting Materials S6
    - 5.2. CHMO-catalysed Kinetic Resolutions S15
    - 5.3. Additional unreactive substrates S38
    - 5.4. $SmI_2$-mediated cyclizations S39
7. NMR Spectra S50
8. References S94


## 1. General Information

Experiments (excluding biotransformations) were performed under an atmosphere of nitrogen using anhydrous solvents, unless otherwise stated. THF and $CH_2Cl_2$ were freshly distilled before use. THF was distilled over sodium wire and benzophenone; $CH_2Cl_2$ was distilled over calcium hydride. All other solvents and reagents used were purchased from commercial suppliers and used according to relevant guidelines. The gene of Cyclohexanone Monooxygenase from *Acinetobacter Calcoaceticus* was supplied by GeneArt.

$^1$H-NMR spectra were obtained at room temperature on a Bruker 400 or 500 MHz spectrometer. $^{13}$C-NMR were obtained at 101 or 126 MHz respectively. $^{19}$F-NMR were obtained at 376 MHz. All NMR spectra were processed using *Mestrenova*© NMR software. Chemical shifts are reported in parts per million (ppm), relative to residual chloroform (δH = 7.27 and δC = 77.0) as internal standards, and coupling constants (*J*) are reported in Hz. Splitting patterns are reported as follows: singlet (s), doublet (d), triplet (t), quartet (q), double of doublets (dd), doublet of triplets (dt), doublet of quartets (dq), doublet of doublets of doublets (ddd), doublet of doublets of triplets (ddt) and multiplet (m).

Column chromatography was carried out using 35 – 70 m, 60Å silica gel. TLC analysis was carried out on aluminium sheets coated with silica gel 60 F254, 0.2 mm thickness and visualised using potassium permanganate solution and/or UV light at 254 nm.

Mass spectra were obtained using positive and negative electrospray (ES±) or atmospheric pressure chemical ionisation (APCI) methodology. Infra-red spectra were recorded as evaporated films or neat using a FT/IR spectrometer – values are reported in $cm^{-1}$.

Enantiomeric ratios were determined by chiral HPLC analysis (Agilent system equipped with a Phenomenex® Lux 5 µm Amylose-1 (4.6 x 250 mm), Chiralpak® 5 µm IA or Chiralcel® OD-H (4.6 x 250 mm) column using HPLC grade $^{i}$PrOH/hexane) or by chiral GC analysis (Agilent system equipped with Beta DEX™ 120 (30 m x 0.25 mm) or ChiraSil® DEX CB (25m x 0.25 mm) column, injector temperature 250°C) in comparison with authentic racemic materials.

Specific rotations were measured on a Rudolph Research Analytical Autopol I Automatic Polarimeter. Melting points were measured on a Stuart Scientific capillary melting point apparatus.

## 2. Protein Production and Purification

Production and Purification of CHMO from *Acinetobacter sp.NCIMB9871*

The pET–28a vector containing the CHMO gene (1 µL) was added to *E. Coli* competent cell strain BL21 and was placed on ice for 20 minutes. The solution was heat shocked in a water bath (42°C) for 45 seconds before cooling on ice for a further 2 minutes. LB media (1 mL) was added and the mixture was incubated at 37°C for 90 minutes. The resulting mixture was then centrifuged (3 minutes, 5000 rpm) and the pellet was resuspended in supernatant (300 µL). The mixture was then spread between two kanamycin (30 µg $mL^{-1}$) LB agar plates and incubated at 37°C for 16 hours.
The starter culture (5 mL) was added to LB media (500 mL) containing kanamycin (500 µL) and the mixture was incubated at 37°C with shaking at 200 rpm. Once the OD600nm had reached approximately 0.6 the cultures were induced with IPTG (1 mM) and incubated overnight at 25°C, with shaking at 200 rpm. Cells were collected by centrifugation (15 min, 5000 rpm) and the supernatant was discarded.

The resulting cell pellet was resuspended in 100 mM Tris/HCl buffer at pH 7.0 (100 mL). The cells were disrupted by sonication (4°C, 20 second pulse, pulse off 20 seconds, 25 cycles) and the resulting suspension was centrifuged at 18000 rpm for 40 minutes. The resulting lysate was loaded onto a 5 mL His-Trap column (GE Healthcare). The His-tagged protein was purified by $Ni^{2+}$ affinity chromatography by use of an AKTA purifier. Protein was eluted by increasing gradient of buffer imidazole concentration from 30 mM to 300 mM. Fractions containing protein were combined and centrifuged at 5000 rpm using a Centricon® concentrator tube with 10 kDA MWCO. The protein was suspended in 100 mM Tris/HCl buffer at pH 7.0 to a concentration of approximately 1.5 mg $mL^{-1}$,

measured spectrophotometrically. The resulting protein was snap-frozen using liquid nitrogen and stored at -80°C.

## 3. CHMO Sequence

```
MSQKMDFDAIVIGGGFGGLYAVKKLRDELELKVQAFDKATDVAGTWYWNRYPGALTDTETHLYCYSWDKE
LLQSLEIKKKYVQGPDVRKYLQQVAEKHDLKKSYQFNTAVQSAHYNEADALWEVTTEYGDKYTARFLITA
LGLLSAPNLPNIKGINQFKGELHHTSRWPDDVSFEGKRVGVIGTGSTGVQVITAVAPLAKHLTVFQRSAQ
YSVPIGNDPLSEEDVKKIKDNYDKIWDGVWNSALAFGLNESTVPAMSVSAEERKAVFEKAWQTGGGFRFM
FETFGDIATNMEANIEAQNFIKGKIAEIVKDPAIAQKLMPQDLYAKRPLCDSGYYNTFNRDNVRLEDVKA
NPIVEITENGVKLENGDFVELDMLICATGFDAVDGNYVRMDIQGKNGLAMKDYWKEGPSSYMGVTVNNYP
NMFMVLGPNGPFTNLPPSIESQVEWISDTIQYTVENNVESIEATKEAEEQWTQTCANIAEMTLFPKAQSW
IFGANIPGKKNTVYFYLGGLKEYRSALANCKNHAYEGFDIQLQRSDIKQPANA
```

Uniprot sequence: **P12015**

## 4. Modelling

The model of CHMO$_{Acineto}$ was created using the PHYRE$^2$ server[1] with the structure of CHMO$_{Rhodo}$ in its 'tight' conformation, in complex with epsilon-caprolactone (4RG3)[2] as a model. Automated docking was performed using AUTODOCK VINA 1.1.2.[3] Coordinates for the (*R*)-lactone **2a** were prepared using ACEDRG.[4] The appropriate pdbqt files for the monomeric model of CHMO$_{Acineto}$ and the ligand, (*R*)-lactone **2a** were prepared in AUTODOCK Tools. The active site of CHMO$_{Acineto}$ beneath the FAD coenzyme was described in a grid size of 28 Å × 28 Å × 26 Å (corresponding to *x*, *y*, *z*) with 0.375 Å spacing, centred around the catalytic centre at positions 15.04 Å × 1.64 Å × 24.06 Å (corresponding to *x*, *y*, *z*), and was generated using AutoGrid in the AUTODOCK Tools interface. The dockings were performed by Autodock-VINA,in which the posed dockings were below 2 Å r.m.s.d. Results generated by VINA were visualised in AUTODOCK Tools 1.5.6, where ligand conformations were assessed based upon lowest VINA energy.

## 4. Chemical Oxidation of Ketone 1b

To a solution of ketone *rac*-**1b** (150 mg, 0.67 mmol) and $NaHCO_3$ (110 mg, 1.31 mmol) in $CH_2Cl_2$ (2.5 mL) was added a solution of *m*CPBA (77%, 168 mg, 0.73 mmol) in $CH_2Cl_2$ (2.5 mL). The reaction was stirred at room temperature for 15 h, then saturated aqueous $NaHCO_3$ (5 mL) was added. The aqueous layer was extracted with $CH_2Cl_2$ (3 x 5 mL), the organic extracts were combined, dried

($MgSO_4$) and concentrated. The crude product mixture was purified by silica gel column chromatography (hexane/EtOAc 95:5) to yield a mixture of lactone and epoxide products.

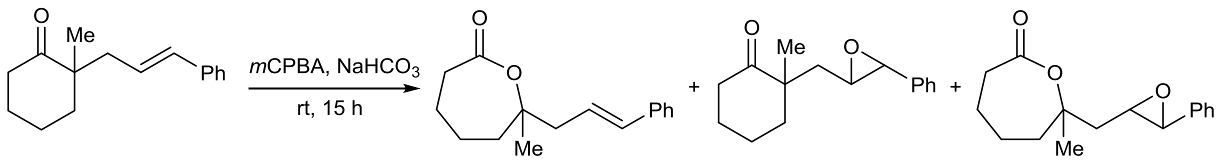


*rac*-**1b** *rac*-**2b** 21% (15%) **S1** 8% (6%) 2.5:1 d.r. **S2** 66% (25%) 2:1 d.r.

Scheme S1: Baeyer-Villiger oxidation of ketone *rac*-**1b** using *m*CPBA Conversions shown are from analysis of the $^1$H NMR data of the crude reaction mixture. Isolated yields are shown in brackets.

2-Methyl-2-((3-phenyloxiran-2-yl)methyl)cyclohexan-1-one **S1** was isolated as a colourless oil and as an 0.4:1 mixture of diastereomers (8 mg, 0.032 mmol, 6%). $^1$H NMR (500 MHz, $CDCl_3$) δ 1.24 (s, 3 H, C$H_3$ from major diastereomer), 1.28 (s, 3 H, C$H_3$ from minor diastereomer), 1.76-1.93 (m, 10.8 H, C$H_2$CH$_2$CH$_2$CH$_2$C(O) from major diastereomer + CH$_2$C$H_2$CH$_2$CH$_2$C(O) from major diastereomer + CH$_2$CH$_2$C$H_2$CH$_2$C(O) from major diastereomer + C$H_2$CH(O)CH from major diastereomer + C$H_2$CH$_2$CH$_2$CH$_2$C(O) from minor diastereomer + CH$_2$C$H_2$CH$_2$CH$_2$C(O) from minor diastereomer + CH$_2$CH$_2$C$H_2$CH$_2$C(O) from minor diastereomer + C$H_a$H$_b$CH(O)CH from minor diastereomer), 1.99 (dd, *J* = 14.4, 5.2 Hz, 0.4 H, CH$_a$$H_b$CH(O)CH from minor diastereomer), 2.35-2.52 (m, 2.8 H, CH$_2$CH$_2$CH$_2$C$H_2$C(O) from major diastereomer + CH$_2$CH$_2$CH$_2$C$H_2$C(O) from minor diastereomer), 2.96-3.00 (m, 1.4 H, CH$_2$C*H*(O)CH from major diastereomer + CH$_2$C*H*(O)CH from minor diastereomer), 3.57 (d, *J* = 2.1 Hz, 0.4 H, CH$_2$CH(O)C*H* from major diastereomer), 3.59 (CH$_2$CH(O)C*H* from minor diastereomer), 7.24-7.35 (m, 7 H, 5 x ArC*H* from major diastereomer + 5 x ArC*H* from minor diastereomer) ppm; $^{13}$C NMR (126 MHz, $CDCl_3$) δ 21.1 (CH$_2$*C*H$_2$CH$_2$CH$_2$C(O) from major + minor diastereomers), 22.8 (*C*H$_3$ from minor diastereomer), 23.4 (*C*H$_3$ from major diastereomer), 27.5 (CH$_2$CH$_2$*C*H$_2$CH$_2$C(O) from major + minor diastereomer), 38.5 (CH$_2$CH$_2$CH$_2$*C*H$_2$C(O) from minor diastereomer), 38.6 (CH$_2$CH$_2$CH$_2$*C*H$_2$C(O) from major diastereomer), 39.2 (*C*H$_2$CH$_2$CH$_2$CH$_2$C(O) from major diastereomer), 39.5 (*C*H$_2$CH$_2$CH$_2$CH$_2$C(O) from minor diastereomer), 40.6 (*C*H$_2$CH(O)CH from minor diastereomer), 40.8 (*C*H$_2$CH(O)CH from major diastereomer), 48.1 (*C* from major + minor diastereomer), 58.4 (CH$_2$CH(O)*C*H from minor diastereomer), 58.6 (CH$_2$CH(O)*C*H from major diastereomer), 59.7 (CH$_2$*C*H(O)CH from major diastereomer), 59.8 (CH$_2$*C*H(O)CH from minor diastereomer), 125.5 (2 x Ar*C*H from major + minor diastereomers), 128.1 (Ar*C*H from major + minor diastereomer), 128.4 (2 x Ar*C*H from major diastereomer), 128.5 (2 x Ar*C*H from minor diastereomer), 214.9 (C(O) from major + minor diastereomer) ppm; IR $\nu_{max}$ (thin film, $cm^{-1}$): 2932, 2863, 1703 (C=O), 1500, 1312, 1124; HRMS calcd. for $C_{16}H_{20}O_2K$ $[M+K]^+$ 283.1095, found 283.1087.

7-Methyl-7-((3-phenyloxiran-2-yl)methyl)oxepan-2-one **S2** was isolated as a colourless oil as an 0.5:1 mixture of diastereomers (43 mg, 0.17 mmol, 25%). $^1$H NMR (400 MHz, $CDCl_3$) δ 1.57 (s, 3 H, $C\mathit{H_3}$ from major diastereomer), 1.63 (s, 1.5 H, $C\mathit{H_3}$ from minor diastereomer), 1.60-1.72 (m, 1.5 H, $CH_2CH_2C\mathit{H_a}H_bCH_2C(O)O$ from major diastereomer + $CH_2CH_2C\mathit{H_a}H_bCH_2C(O)O$ from minor diastereomer), 1.78-2.02 (m, 8 H, $C\mathit{H_a}H_bCH_2CH_2CH_2C(O)O$ from major diastereomer + $CH_2C\mathit{H_2}CH_2CH_2C(O)O$ from major diastereomer + $CH_2CH_2CH_a\mathit{H_b}CH_2C(O)O$ from major diastereomer + $C\mathit{H_a}H_bCH(O)CH$ from major diastereomer + $C\mathit{H_2}CH_2CH_2CH_2C(O)O$ from minor diastereomer + $CH_2C\mathit{H_2}CH_2CH_2C(O)O$ from minor diastereomer + $CH_2CH_2CH_a\mathit{H_b}CH_2C(O)O$ from minor diastereomer + $C\mathit{H_a}H_bCH(O)CH$ from minor diastereomer), 2.05-2.17 (m, 2 H, $CH_a\mathit{H_b}CH_2CH_2CH_2C(O)O$ from major diastereomer + $CH_a\mathit{H_b}CH(O)CH$ from major diastereomer), 2.24 (dd, $J$ = 14.4, 4.2 Hz, 0.5 H, $CH_a\mathit{H_b}CH(O)CH$ from minor diastereomer), 2.64-2.82 (m, 3 H, $CH_2CH_2CH_2C\mathit{H_2}C(O)O$ from major diastereomer + $CH_2CH_2CH_2C\mathit{H_2}C(O)O$ from minor diastereomer), 3.19-3.24 (m, 1.5 H, $CH_2C\mathit{H}(O)CH$ from major diastereomer + $CH_2C\mathit{H}(O)CH$ from minor diastereomer), 3.64-3.65 (m, 1.5 H, $CH_2CH(O)C\mathit{H}$ from major diastereomer + $CH_2CH(O)C\mathit{H}$ from minor diastereomer) 7.26-7.37 (m, 7.5 H, 5 x ArC$\mathit{H}$ from major diastereomer + 5 x ArC$\mathit{H}$ from minor diastereomer) ppm; $^{13}$C NMR (101 MHz, $CDCl_3$) δ 23.3 ($CH_2CH_2\mathit{C}H_2CH_2C(O)O$ from major + minor diastereomer), 23.8 ($CH_2\mathit{C}H_2CH_2CH_2C(O)O$ from minor diastereomer), 23.9 ($CH_2\mathit{C}H_2CH_2CH_2C(O)O$ from major diastereomer), 24.4 ($\mathit{C}H_3$ from minor diastereomer), 25.2 ($\mathit{C}H_3$ from major diastereomer), 37.3 ($CH_2CH_2CH_2\mathit{C}H_2C(O)O$ from major diastereomer), 37.4 ($CH_2CH_2CH_2\mathit{C}H_2C(O)O$ from minor diastereomer), 38.6 ($\mathit{C}H_2CH_2CH_2CH_2C(O)O$ from major diastereomer), 39.5 ($\mathit{C}H_2CH_2CH_2CH_2C(O)O$ from minor diastereomer), 45.6 ($\mathit{C}H_2CH(O)CH$ from minor diastereomer), 45.9 ($\mathit{C}H_2CH(O)CH$ from major diastereomer), 58.0 ($CH_2CH(O)\mathit{C}H$ from major diastereomer), 58.1 ($CH_2CH(O)\mathit{C}H$ from minor diastereomer), 58.8 ($CH_2\mathit{C}H(O)CH$ from major + minor diastereomer), 82.3 ($\mathit{C}$ from major diastereomer), 82.4 ($\mathit{C}$ from minor diastereomer), 125.6 (2 x Ar$\mathit{C}$H from major + minor diastereomer), 128.2 (Ar$\mathit{C}$H from major + minor diastereomer), 128.5 (2 x Ar$\mathit{C}$H from major + minor diastereomer), 137.0 (Ar$\mathit{C}$ from major + minor diastereomer), 174.3 ($\mathit{C}$(O)O from minor diastereomer), 174.4 ($\mathit{C}$(O)O from major diastereomer) ppm; IR $\nu_{max}$ (thin film, cm$^{-1}$): 2930, 1713 (C=O), 1453, 1353, 1288, 1170, 1017; HRMS calcd. for $C_{16}H_{20}O_3K$ $[M+K]^+$ 299.1044, found 299.1037.

For analytical data of **1b** and **2b** see the experimental section.

# 5. Experimental Section

## 5.1. Preparation of Racemic Ketone Starting Materials

### General Procedure A: Dieckmann Condensation and Alkylation

### Allyl 1-methyl-2-oxocyclohexane-1-carboxylate (S3)[5]

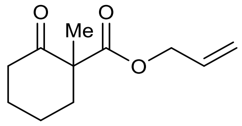

A solution of diallyl pimelate[6] (20.0 g, 94%, 78.2 mmol) in THF (13 mL) was added slowly to a suspension of sodium hydride (3.44 g, 60%, 86.0 mmol) in THF (65 mL). The reaction was stirred at 40°C for 18 h, then methyl iodide (6.3 mL, 101.7 mmol) was added the reaction was stirred for a further 3 hours. The mixture was cooled to room temperature and $H_2O$ (16 mL) was added slowly. The solution was concentrated under vacuum and extracted with EtOAc (3 x 20 mL), washed with brine (15 mL), dried ($MgSO_4$) and concentrated. The crude reaction mixture was used in the next step without further purification.

### General Procedure B: Pd-Catalysed Decarboxylation of β-keto esters

### 2-Allyl-2-methylcyclohexan-1-one (1a)[5]

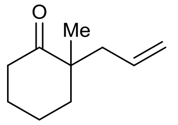

The crude reaction mixture of allyl 1-methyl-2-oxocyclohexane-1-carboxylate **S3** (78.2 mmol) was added to THF (700 mL), followed by $Pd_2(dba)_3$ (3.55 g, 3.88 mmol, 5 mol%) and $PPh_3$ (4.07 g, 15.5 mmol, 20 mol%). The resulting solution was stirred at 67°C for 5 h. The mixture was cooled to room temperature and filtered through a silica plug with the aid of $Et_2O$. The crude product was distilled under vacuum (5 mBar, 70-110°C) to yield the title product as a colourless oil (6.38 g, 41.3 mmol, 53%). $^1$H NMR (400 MHz, $CDCl_3$) δ 1.08 (s, 3 H, $C\mathit{H_3}$), 1.56–1.62 (m, 1 H, $C\mathit{H_a}H_bCH_2CH_2CH_2C(O)$), 1.72-1.87 (m, 5 H, $CH_a\mathit{H_b}CH_2CH_2CH_2C(O)$ + $CH_2C\mathit{H_2}CH_2CH_2C(O)$ + $CH_2CH_2C\mathit{H_2}CH_2C(O)$), 2.22-2.26 (m, 1 H, $C\mathit{H_a}H_bCH{=}CH_2$), 2.35-2.41 (m, 3 H, $CH_a\mathit{H_b}CH{=}CH_2$ + $CH_2CH_2CH_2C\mathit{H_2}C(O)$), 5.03–5.07 (m, 2 H, $CH_2CH{=}C\mathit{H_2}$), 5.66–5.75 (m, 1 H, $CH_2C\mathit{H}{=}CH_2$) ppm; $^{13}$C NMR (101 MHz, $CDCl_3$) δ 21.1 ($CH_2\mathit{C}H_2CH_2CH_2C(O)$), 22.6 ($\mathit{C}H_3$), 27.4 ($CH_2CH_2\mathit{C}H_2CH_2C(O)$), 38.6 ($CH_2CH_2CH_2\mathit{C}H_2C(O)$), 38.8

($CH_2CH_2CH_2CH_2C(O)$), 42.0 ($CH_2CH=CH$), 48.4 (*C*), 117.9 ($CH_2CH=CH_2$), 133.8 ($CH_2CH=CH_2$), 215.4 (*C*(O)) ppm.

**General procedure C: Cross Metathesis**

**2-Cinnamyl-2-methylcyclohexan-1-one (1b)**

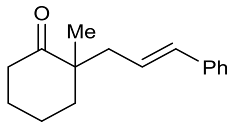


To a solution of ketone **1a** (2.00 g, 13.1 mmol) and styrene (4.52 mL, 39.4 mmol) in degassed $CH_2Cl_2$ was added Hoveyda–Grubbs 2$^{nd}$ Generation catalyst (82 mg, 0.131 mmol, 1 mol%). The reaction was stirred at room temperature under a stream of $N_2$ for 16 h. Solvent was removed under vacuum and the resulting crude was purified by silica gel column chromatography (hexane/EtOAc 100:0 to 90:10) to yield the title product as a pale yellow oil (1.27 g, 5.56 mmol, 42%). $^{1}$H NMR (400 MHz, $CDCl_3$) δ 1.14 (s, 3 H, $CH_3$), 1.64−1.87 (m, 6 H, $CH_2CH_2CH_2CH_2C(O)$ + $CH_2CH_2CH_2CH_2C(O)$ + $CH_2CH_2CH_2CH_2C(O)$), 2.39−2.48 (m, 4 H, $CH_2CH_2CH_2CH_2C(O)$ + $CH_2CH=CHAr$), 6.13 (dt, *J* = 15.8, 7.5 Hz, 1 H, $CH_2CH=CHAr$), 6.40 (d, *J* = 15.6 Hz, 1 H, $CH_2CH=CHAr$), 7.19−7.23 (m, 1 H, Ar*CH*), 7.28−7.36 (m, 4 H, 4 x Ar*CH*) ppm; $^{13}$C NMR (101 MHz, $CDCl_3$) δ 21.1 ($CH_2CH_2CH_2CH_2C(O)$), 22.9 ($CH_3$), 27.4 ($CH_2CH_2CH_2CH_2C(O)$), 38.6 ($CH_2CH_2CH_2CH_2C(O)$), 38.8 ($CH_2CH_2CH_2CH_2C(O)$), 41.2 ($CH_2CH=CHAr$), 48.9 (*C*), 125.7 ($CH_2CH=CHAr$), 126.1 (2 x Ar*C*H), 127.1 (Ar*C*H), 128.5 (2 x Ar*C*H), 133.0 (Ar*C*), 137.3 ($CH_2CH=CHAr$), 215.5 (*C*(O)) ppm; IR $\nu_{max}$ (thin film, $cm^{-1}$): 2932, 1703 (C=O), 1449, 1424, 1122, 966; HRMS calcd. for $C_{16}H_{20}ONa$ $[M+Na]^+$: 251.1406, found 251.1396.

**(*E*)-2-(3-(4-Fluorophenyl)allyl)-2-methylcyclohexan-1-one (1c)**

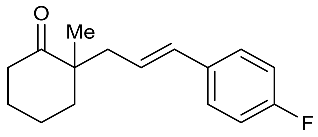


Prepared according to general procedure **C** using ketone **1a** (200 mg, 1.31 mmol), 4-fluorostyrene (0.47 mL, 3.93 mmol) and Hoveyda–Grubbs 2$^{nd}$ Generation catalyst (8 mg, 0.013 mmol, 1 mol%). Purification by silica gel column chromatography (hexane/EtOAc 100:0 to 98:2) yielded the title product as a colourless oil (125 mg, 0.507 mmol, 39%). $^{1}$H NMR (400 MHz, $CDCl_3$) δ 1.14 (s, 3 H, $CH_3$), 1.67−1.87 (m, 6 H, $CH_2CH_2CH_2CH_2C(O)$ + $CH_2CH_2CH_2CH_2C(O)$ + $CH_2CH_2CH_2CH_2C(O)$), 2.37−2.50 (m, 4 H, $CH_2CH_2CH_2CH_2C(O)$ + $CH_2CH=CHAr$), 6.04 (dt, *J* = 15.6, 7.6 Hz, 1 H, $CH_2CH=CHAr$), 6.36 (d, *J* = 15.8 Hz, $CH_2CH=CHAr$), 6.96−7.00 (m, 2 H, 2 x Ar*CH*), 7.28−7.31 (m, 2 H, 2 x Ar*CH*); $^{13}$C NMR (101 MHz, $CDCl_3$)

δ 21.1 ($CH_2CH_2CH_2CH_2C(O)$), 23.0 (*C*$H_3$), 27.4 ($CH_2CH_2$*C*$H_2CH_2C(O)$), 38.7 ($CH_2CH_2CH_2$*C*$H_2C(O)$), 38.8 (*C*$H_2CH_2CH_2CH_2C(O)$), 41.2 (*C*$H_2$CH=CHAr), 48.9 (*C*), 115.2 (d, *J* = 21.5 Hz, 2 x Ar*C*H), 125.5 ($CH_2$*C*H=CHAr), 127.5 (d, J = 8.0 Hz, 2 x Ar*C*H), 131.8 ($CH_2$CH=*C*HAr), 133.5 (Ar*C*), 162.1 (d, *J* = 246.1 Hz, Ar*C*F), 215.4 (*C*(O)) ppm; IR $\nu_{max}$ (thin film, $cm^{-1}$): 2974, 1705 (C=O), 1508, 1226, 1157, 971; $^{19}$F NMR (376 MHz, $CDCl_3$) -115.3 ppm; HRMS calcd. for $C_{16}H_{23}ONF$ $[M+NH_4]^+$: 264.1758, found 264.1761.

**(*E*)-2-Methyl-2-(3-(naphthalen-2-yl)allyl)cyclohexan-1-one (1d)**

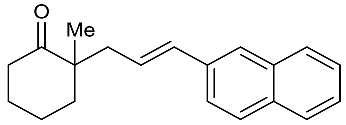

Prepared according to general procedure **C** using ketone **1a** (200 mg, 1.31 mmol), 2-vinylnapthalene (0.61 g 3.93 mmol) and Hoveyda–Grubbs 2nd Generation catalyst (8 mg, 0.013 mmol, 1 mol%). Purification by silica gel column chromatography (hexane/EtOAc 100:0 to 90:10) yielded the title product as a yellow solid (144 mg, 0.517 mmol, 39%): mp (hexane) 66-68 °C. $^1$H NMR (400 MHz, $CDCl_3$) δ 1.18 (s, 3 H, C*H*$_3$), 1.64−1.90 (m, 6 H, C*H*$_2CH_2CH_2CH_2C(O)$ + $CH_2$C*H*$_2CH_2CH_2C(O)$ + $CH_2CH_2$C*H*$_2CH_2C(O)$), 2.44−2.57 (m, 4 H, $CH_2CH_2CH_2$C*H*$_2$C(O) + C*H*$_2$CH=CHAr), 6.27 (dt, *J* = 15.5, 7.6 Hz, 1 H, $CH_2$C*H*=CHAr), 6.57 (d, *J* = 15.9 Hz, 1 H, $CH_2$CH=C*H*Ar), 7.40−7.57 (m, 2 H, 2 x ArC*H*), 7.57 (d, *J* = 8.5 Hz, 1 H, ArC*H*), 7.68 (s, 1 H, ArC*H*), 7.78 (t, *J* = 9.2 Hz, 3 H, 3 x *ArCH*); $^{13}$C NMR (101 MHz, $CDCl_3$) δ 21.1 ($CH_2$*C*$H_2CH_2CH_2C(O)$), 23.0 (*C*$H_3$), 27.4 ($CH_2CH_2$*C*$H_2CH_2C(O)$), 38.6 ($CH_2CH_2CH_2$*C*$H_2C(O)$), 38.9 (*C*$H_2CH_2CH_2CH_2C(O)$), 41.4 (*C*$H_2$CH=CHAr), 49.0 (*C*), 123.6 (Ar*C*H), 125.6 (2 x Ar*C*H), 126.1 (Ar*C*H), 126.2 ($CH_2$*C*H=CHAr), 127.6 (Ar*C*H), 127.8 (Ar*C*H), 128.0 (Ar*C*H), 132.8 (Ar*C*), 133.1 ($CH_2$CH=*C*HAr), 133.6 (Ar*C*), 134.8 (Ar*C*), 215.4 (*C*(O)) ppm; IR $\nu_{max}$ (thin film, $cm^{-1}$): 2932, 1702 (C=O), 1450, 1265, 1142, 962; HRMS calcd. for $C_{20}H_{22}ONa$ $[M+Na]^+$: 301.1563, found 301.1552.

**(*E*)-2-(3-(3-Bromophenyl)allyl)-2-methylcyclohexan-1-one (1e)**

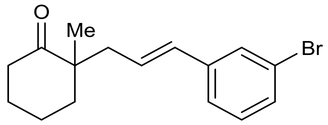

Prepared according to general procedure **C** using ketone **1a** (200 mg, 1.31 mmol), 3-bromostyrene (0.51 mL, 3.93 mmol) and Hoveyda–Grubbs 2nd Generation catalyst (8 mg, 0.013 mmol, 1 mol%). Purification by silica gel column chromatography (hexane/EtOAc 100:0 to 98:2) yielded the title product as a colourless oil (232 mg, 0.755 mmol, 58%). $^1$H NMR (400 MHz, $CDCl_3$) δ 1.14 (s, 3 H, C*H*$_3$), 1.65−1.87 (m, 6 H, C*H*$_2CH_2CH_2CH_2C(O)$ + $CH_2$C*H*$_2CH_2CH_2C(O)$ + $CH_2CH_2$C*H*$_2CH_2C(O)$), 2.37−2.50 (m, 4 H,

$CH_2CH_2CH_2CH_2$C(O) + C*H₂*CH=CHAr), 6.15 (dt, *J* = 15.6, 7.5 Hz, 1 H, $CH_2$C*H*=CHAr), 6.33 (d, *J* = 15.9 Hz, 1 H, $CH_2$CH=C*H*Ar), 7.16 (t, *J* = 7.8 Hz, 1 H, ArC*H*), 7.24 (d, *J* = 7.6 Hz, 1 H, ArC*H*), 7.36 (d, *J* = 7.8 Hz, 1 H, ArC*H*), 7.48 (s, 1 H, ArC*H*); $^{13}$C NMR (101 MHz, $CDCl_3$) δ 21.1 ($CH_2CH_2CH_2CH_2$C(O)), 23.0 ($CH_3$), 27.3 ($CH_2CH_2CH_2CH_2$C(O)), 38.6 ($CH_2CH_2CH_2CH_2$C(O)), 38.8 ($CH_2CH_2CH_2CH_2$C(O)), 41.2 ($CH_2$CH=CHAr), 48.9 (*C*), 122.7 (Ar*C*Br), 124.8 ($CH_2$*C*H=CHAr), 127.7 (Ar*C*H), 128.9 (Ar*C*H), 130.0 (2 x Ar*C*H), 131.6 ($CH_2$CH=*C*HAr), 139.6 (Ar*C*), 215.2 (*C*(O)) ppm; IR $\nu_{max}$ (thin film, $cm^{-1}$): 2973, 1732 (C=O), 1450, 1266, 1087, 1046, 879; HRMS calcd. for $C_{16}H_{23}ONBr$ $[M+NH_4]^+$: 324.0958, found 324.0961.

**(*E*)-2-(3-(2-Chlorophenyl)allyl)-2-methylcyclohexan-1-one (1f)**

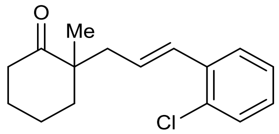


Prepared according to general procedure **C** using ketone **1a** (200 mg, 1.31 mmol), 2-chlorostyrene (0.50 mL, 3.93 mmol) and Hoveyda–Grubbs 2nd Generation catalyst (16 mg, 0.026 mmol, 2 mol%). Purification by silica gel column chromatography (hexane/EtOAc 100:0 to 98:2) yielded the title product as a pale yellow oil (213 mg, 0.810 mmol, 62%). $^1$H NMR (400 MHz, $CDCl_3$) δ 1.16 (s, 3 H, $CH_3$) 1.66–1.89 (m, 6 H, $CH_2CH_2CH_2CH_2$C(O) + $CH_2CH_2CH_2CH_2$C(O) + $CH_2CH_2CH_2CH_2$C(O)), 2.42–2.58 (m, 4 H, $CH_2CH_2CH_2CH_2$C(O) + $CH_2$CH=CHAr), 6.11 (dt, *J* = 15.8, 7.8, 1 H, $CH_2$C*H*=CHAr), 6.78 (d, *J* = 15.8, 1 H, $CH_2$CH=C*H*Ar), 7.13–7.22 (m, 2 H, 2 x ArC*H*), 7.33 (dd, *J* = 7.8, 1.5 Hz, 1 H, ArC*H*), 7.48 ((dd, *J* = 7.8, 1.5 Hz, 1 H, ArC*H*); $^{13}$C NMR (101 MHz, $CDCl_3$) δ 21.1 ($CH_2CH_2CH_2CH_2$C(O)), 23.0 ($CH_3$), 27.4 ($CH_2CH_2CH_2CH_2$C(O)), 38.8 ($CH_2CH_2CH_2CH_2$C(O)), 38.9 ($CH_2CH_2CH_2CH_2$C(O)), 41.4 ($CH_2$CH=CHAr), 48.9 (*C*), 126.8 (Ar*C*H), 126.9 (Ar*C*H), 128.1 (Ar*C*H), 128.9 ($CH_2$*C*H=CHAr), 129.3 ($CH_2$CH=*C*HAr), 129.5 (Ar*C*H), 132.6 (Ar*C*Cl), 135.6 (Ar*C*), 215.3 (*C*(O)) ppm; IR $\nu_{max}$ (thin film, $cm^{-1}$): 2933, 1703 (C=O), 1468, 1440, 1122, 967; HRMS calcd. for $C_{16}H_{20}OCl$ $[M+H]^+$: 263.1197, found 263.1198.

**(*E*)-2-Methyl-2-(3-(4-(trifluoromethyl)phenyl)allyl)cyclohexan-1-one (1g)**

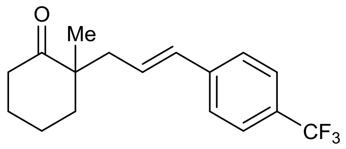


Prepared according to general procedure **C** using ketone **1a** (200 mg, 1.31 mmol), 4-(trifluoromethyl)styrene (0.58 mL, 3.93 mmol) and Hoveyda–Grubbs 2nd Generation catalyst (16 mg, 0.026 mmol, 2 mol%). Purification by silica gel column chromatography (hexane/EtOAc 100:0 to 98:2) yielded the title product as a pale yellow oil (224 mg, 0.755 mmol, 58%). $^1$H NMR (400 MHz,

$CDCl_3$) δ 1.16 (s, 3 H, $C\mathit{H}_3$), 1.65−1.88 (m, 6 H, $C\mathit{H}_2CH_2CH_2CH_2C(O)$ + $CH_2C\mathit{H}_2CH_2CH_2C(O)$ + $CH_2CH_2C\mathit{H}_2CH_2C(O)$), 2.37−2.53 (m, 4 H, $CH_2CH_2CH_2C\mathit{H}_2C(O)$ + $C\mathit{H}_2CH{=}CHAr$), 6.25 (dt, *J* = 15.8, 7.8 Hz, 1 H, $CH_2C\mathit{H}{=}CHAr$), 6.43 (d, *J* = 15.8 Hz, 1 H, $CH_2CH{=}C\mathit{H}Ar$), 7.43 (d, *J* = 8.3 Hz, 2 H, 2 x ArC*H*), 7.54 (d, *J* = 8.3 Hz, 2 H, 2 x ArC*H*) ppm; $^{13}C$ NMR (101 MHz, $CDCl_3$) δ 21.1 ($CH_2\mathit{C}H_2CH_2CH_2C(O)$), 23.0 ($\mathit{C}H_3$), 27.3 ($CH_2CH_2\mathit{C}H_2CH_2C(O)$), 38.7 ($CH_2CH_2CH_2\mathit{C}H_2C(O)$), 38.8 ($\mathit{C}H_2CH_2CH_2CH_2C(O)$), 41.4 ($\mathit{C}H_2CH{=}CHAr$), 48.9 (*C*), 122.9 (Ar*C*$CF_3$) 125.4 (q, *J* = 3.9 Hz, 2 x Ar*C*H), 126.2 (2 x Ar*C*H), 128.9 ($CH_2\mathit{C}H{=}CHAr$), 131.7 ($CH_2CH{=}\mathit{C}HAr$), 140.8 (Ar*C*), 215.1 (*C*(O)) ppm, ArC$\mathit{C}F_3$ not observed; $^{19}F$ NMR (376 MHz, $CDCl_3$) -62.5 ppm; IR $\nu_{max}$ (thin film, $cm^{-1}$): 2935, 1704 (C=O), 1614, 1323, 1162, 1120, 1067, 971; HRMS calcd. for $C_{17}H_{23}ONF_3$ $[M+NH_4]^+$: 314.1726, found 314.1729.

**(*E*)-2-Methyl-2-(3-(*o*-tolyl)allyl)cyclohexan-1-one (1h)**

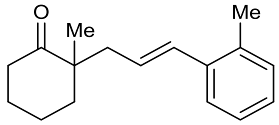


Prepared according to general procedure **C** using ketone **1a** (200 mg, 1.31 mmol), 2-methylstyrene (0.51 mL, 3.93 mmol) and Hoveyda–Grubbs 2nd Generation catalyst (16 mg, 0.026 mmol, 2 mol%). Purification by silica gel column chromatography (hexane/EtOAc 100:0 to 98:2) yielded the title product as a yellow oil (164 mg, 0.677 mmol, 52%). $^1H$ NMR (400 MHz, $CDCl_3$) δ 1.15 (s, 3 H, $C\mathit{H}_3$), 1.63−1.88 (m, 6 H, $C\mathit{H}_2CH_2CH_2CH_2C(O)$ + $CH_2C\mathit{H}_2CH_2CH_2C(O)$ + $CH_2CH_2C\mathit{H}_2CH_2C(O)$), 2.33 (s, 3 H, $ArCC\mathit{H}_3$), 2.40−2.55 (m, 4 H, $CH_2CH_2CH_2C\mathit{H}_2C(O)$ + $C\mathit{H}_2CH{=}CHAr$), 5.98 (dt, *J* = 15.8, 8.0 Hz, 1 H, $CH_2C\mathit{H}{=}CHAr$), 6.60 (d, *J* = 15.6 Hz, 1 H, $CH_2CH{=}C\mathit{H}Ar$), 7.12−7.16 (m, 3 H, 3 x ArC*H*), 7.38−7.40 (m, 1 H, ArC*H*) ppm; $^{13}C$ NMR (101 MHz, $CDCl_3$) δ 19.8 ($ArC\mathit{C}H_3$), 21.1 ($CH_2\mathit{C}H_2CH_2CH_2C(O)$), 22.9 ($\mathit{C}H_3$), 27.4 ($CH_2CH_2\mathit{C}H_2CH_2C(O)$), 38.6 ($CH_2CH_2CH_2\mathit{C}H_2C(O)$), 38.8 ($\mathit{C}H_2CH_2CH_2CH_2C(O)$), 41.4 ($\mathit{C}H_2CH{=}CHAr$), 48.9 (*C*), 125.8 (Ar*C*H), 126.0 (Ar*C*H), 127.1 (Ar*C*H), 127.2 ($CH_2\mathit{C}H{=}CHAr$), 130.1 (Ar*C*H), 131.1 ($CH_2CH{=}\mathit{C}HAr$), 135.0 (Ar*C*), 136.7 (Ar*C*$CH_3$), 215.4 (*C*(O)) ppm; IR $\nu_{max}$ (thin film, $cm^{-1}$): 2931, 1703 (C=O), 1459, 1123, 967; HRMS calcd. for $C_{17}H_{23}O$ $[M+H]^+$: 243.1743, found 243.1748.

**Allyl 1-ethyl-2-oxocyclohexane-1-carboxylate (S4)**

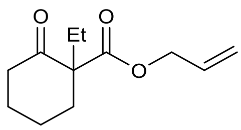


Prepared according to general procedure **A** using diallyl pimelate (1.0 g, 4.12 mmol), sodium hydride (0.18 g, 4.53 mmol) and ethyl iodide (0.43 mL, 5.36 mmol) in THF (3.6 mL) The crude reaction mixture was used in the following step without further purification.

**2-Allyl-2-ethylcyclohexan-1-one (1i)[7]**

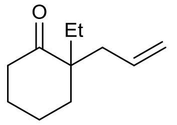


Prepared according to general procedure **B** using crude allyl 1-ethyl-2-oxocyclohexane-1-carboxylate **S2** (0.61 g, 2.90 mmol), $Pd_2(dba)_3$ (0.13 g, 0.145 mmol, 5 mol%), and $PPh_3$ (0.15 g, 0.58 mmol, 20 mol%) in THF (26 mL). Purification by silica gel column chromatography (hexane/EtOAc 95:5) yielded the title product as a yellow oil (268 mg, 1.61 mmol, 56%). $^1$H NMR (400 MHz, $CDCl_3$) 0.77 (t, *J* = 7.5 Hz, 3 H, $CH_2CH_3$), 1.49 (apparent dq, *J* = 14.8, 7.5 Hz, 1 H, $CH_aH_bCH_3$), 1.61-1.91 (m, 7 H, $CH_aH_bCH_3$ + $CH_2CH_2CH_2CH_2C(O)$ + $CH_2CH_2CH_2CH_2C(O)$ + $CH_2CH_2CH_2CH_2C(O)$), 2.22-2.44 (m, 4 H, $CH_2CH_2CH_2CH_2C(O)$ + $CH_2CH{=}CH_2$), 5.01-5.08 (m, 2 H, $CH_2CH{=}CH_2$), 5.63-5.74 (m, 1 H, $CH_2CH{=}CH_2$); $^{13}$C NMR (101 MHz, $CDCl_3$) δ 7.8 ($CH_2CH_3$), 20.8 ($CH_2CH_2CH_2CH_2C(O)$), 27.2 ($CH_2CH_3$), 27.3 ($CH_2CH_2CH_2CH_2C(O)$), 36.1 ($CH_2CH_2CH_2CH_2C(O)$), 38.5 ($CH_2CH{=}CH_2$), 39.2 ($CH_2CH_2CH_2CH_2C(O)$), 51.6 (*C*), 117.6 ($CH_2CH{=}CH_2$), 134.2 ($CH_2CH{=}CH_2$), 215.0 (*C*(O)) ppm.

**Allyl 1-methyl-2-oxocyclopentane-1-carboxylate (S5)[8]**

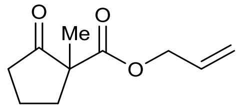


Prepared according to general procedure using **A** diallyl adipate[1] (18.0 g, 78.2 mmol), sodium hydride (3.44 g, 60%, 86.0 mmol) and methyl iodide (6.3 mL, 102 mmol) in THF (78 mL). The crude reaction mixture was used in the following step without further purification.

**2-Allyl-2-methylcyclopentan-1-one (3a)[8]**

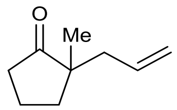


Prepared according to general procedure **B** using the crude reaction mixture of allyl 1-methyl-2-oxocyclopentane-1-carboxylate **S3** (6.35 g, 34.8 mmol), $Pd_2(dba)_3$ (1.59 g, 1.74 mmol, 5 mol%) and $PPh_3$ (1.83 g, 6.97 mmol, 20 mol%) in THF (315 mL). The crude product was purified by silica gel column chromatography (hexane/EtOAc 97:3) to yield the title product as a colourless oil (2.52 g, 18.2 mmol, 52%). $^1$H NMR (400 MHz, $CDCl_3$) δ 1.01 (s, 3 H, $CH_3$), 1.53–1.57 (m, 1 H, $CH_aH_bCH_2CH_2C(O)$), 1.69-1.80 (m, 3 H, $CH_2CH_2CH_2C(O)$ + $CH_aH_bCH_2CH_2C(O)$), 1.94–2.20 (m, 4 H, $CH_2CH_2CH_2CH_2C(O)$ + $CH_2CH{=}CHAr$), 4.88–4.95 (m, 2 H, $CH_2CH{=}CH_2$), 5.50–5.61 (m, 1 H, $CH_2CH{=}CH_2$)

ppm; $^{13}C$ NMR (101 MHz, $CDCl_3$) δ 18.7 ($CH_2\mathit{C}H_2CH_2C(O)$), 21.8 ($\mathit{C}H_3$), 35.1 ($\mathit{C}H_2CH_2CH_2C(O)$), 37.7 ($CH_2CH_2\mathit{C}H_2C(O)$), 40.1 ($\mathit{C}H_2CH=CHAr$), 48.2 (*C*), 118.2 ($CH_2CH=\mathit{C}H_2$), 133.9 ($CH_2\mathit{C}H=CH_2$), 223.2 (*C*(O)).

**2-Cinnamyl-2-methylcyclopentan-1-one (3b)**

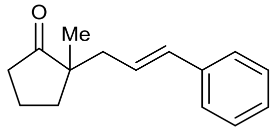


Prepared according to general procedure **C** using ketone **3a** (1.5 g, 10.9 mmol), styrene (0.50 mL, 32.6 mmol) and Hoveyda–Grubbs 2[nd] Generation catalyst (68 mg, 0.109 mmol, 1 mol%). Purification by silica gel column chromatography (hexane/EtOAc 100:0 to 98:2) yielded the title product as a pale yellow oil (0.984 g, 4.59 mmol, 42%). $^{1}H$ NMR (400 MHz, $CDCl_3$) δ 1.07 (s, 3 H, $C\mathit{H_3}$), 1.72−1.77 (m, 1 H, $C\mathit{H_a}H_bCH_2CH_2C(O)$), 1.89−1.92 (m, 2 H, $CH_2C\mathit{H_2}CH_2C(O)$), 1.97−2.04 (m, 1 H, $CH_a\mathit{H_b}CH_2CH_2C(O)$), 2.20−2.37 (m, 4 H, $CH_2CH_2C\mathit{H_2}C(O)$ + $C\mathit{H_2}CH=CHAr$), 6.12 (ddd, *J* = 15.6, 8.0, 7.3 Hz, 1 H, $CH_2C\mathit{H}=CHAr$), 6.42 (d, *J* = 15.7 Hz, 1 H, $CH_2CH=C\mathit{H}Ar$), 7.20−7.24 (m, 1 H, ArC*H*), 7.29−7.37 (m, 4 H, 4 x ArC*H*) ppm; $^{13}C$ NMR (101 MHz, $CDCl_3$) δ 18.8 ($CH_2\mathit{C}H_2CH_2C(O)$), 22.1 ($\mathit{C}H_3$), 35.2 ($\mathit{C}H_2CH_2CH_2C(O)$), 37.8 ($CH_2CH_2\mathit{C}H_2C(O)$), 40.2 ($\mathit{C}H_2CH=CHAr$), 48.8 (*C*), 125.6 ($CH_2\mathit{C}H=CHAr$), 126.1 (2 x Ar*C*H), 127.2 (ArCH), 128.5 (2 x Ar*C*H), 133.3 ($CH_2CH=\mathit{C}HAr$), 137.3 (Ar*C*), 222.7 (*C*(O)) ppm; IR $\nu_{max}$ (thin film, $cm^{-1}$) 2959, 1732 (C=O), 1449, 1161, 1060, 967; HRMS calcd. for $C_{15}H_{19}O$ $[M+H]^+$: 215.1430, found 215.1425.

**(*E*)-2-(3-(4-Fluorophenyl)allyl)-2-methylcyclopentan-1-one (3c)**

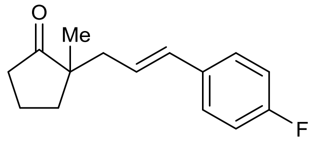


Prepared according to general procedure **C** using ketone **3a** (200 mg, 1.45 mmol), 4-fluorostyrene (0.52 mL, 4.35 mmol) and Hoveyda–Grubbs 2[nd] Generation catalyst (18 mg, 0.029 mmol, 2 mol%). Purification by silica gel column chromatography (hexane/EtOAc 100:0 to 98:2) yielded the title product as a yellow oil (120 mg, 0.517 mmol, 36%). $^{1}H$ NMR (400 MHz, $CDCl_3$) δ 0.99 (s, 3 H, $C\mathit{H_3}$), 1.64−1.70 (m, 1 H, $C\mathit{H_a}H_bCH_2CH_2C(O)$), 1.82−1.96 (m, 3 H, $CH_2C\mathit{H_2}CH_2C(O)$ + $CH_a\mathit{H_b}CH_2CH_2C(O)$) 2.10−2.30 (m, 4 H, $CH_2CH_2C\mathit{H_2}C(O)$ + $C\mathit{H_2}CH=CHAr$), 5.95 (dt, *J* = 15.5, 7.6 Hz, 1 H, $CH_2C\mathit{H}=CHAr$), 6.30 (d, *J* = 15.6 Hz, 1 H, $CH_2CH=C\mathit{H}Ar$), 6.91 (t, *J* = 8.7 Hz, 2 H, 2 x ArC*H*), 7.23 (dd, *J* = 8.5, 5.4 Hz, 2 H, 2 x ArC*H*) ppm; $^{13}C$ NMR (101 MHz, $CDCl_3$) δ 18.7 ($CH_2\mathit{C}H_2CH_2C(O)$), 22.1 ($\mathit{C}H_3$), 35.3 ($\mathit{C}H_2CH_2CH_2C(O)$), 37.7 ($CH_2CH_2\mathit{C}H_2C(O)$), 40.1 ($\mathit{C}H_2CH=CHAr$), 48.6 (*C*), 115.4 (2 x Ar*C*H), 125.4 ($CH_2CH=\mathit{C}HAr$), 127.6 (2 x Ar*C*H), 132.1 ($CH_2\mathit{C}H=CHAr$), 133.5 (Ar*C*), 162.1 (d, *J* = 247.5 Hz, Ar*C*F), 223.1 (*C*(O)) ppm; $^{19}F$ NMR

(376 MHz, $CDCl_3$) -115.1 ppm; IR $\nu_{max}$ (thin film, $cm^{-1}$): 2962, 1734 (C=O), 1600, 1508, 1227, 1158, 969; HRMS calcd. for $C_{15}H_{18}OF$ $[M+H]^+$: 233.1336, found 233.1336.

**(*E*)-2-Methyl-2-(3-(naphthalen-2-yl)allyl)cyclopentan-1-one (3d)**

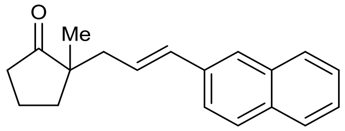


Prepared according to general procedure **C** using ketone **3a** (200 mg, 1.45 mmol), 2-vinylnapthalene (0.67 g, 4.35 mmol) and Hoveyda–Grubbs 2nd Generation catalyst (18 mg, 0.029 mmol, 2 mol%). Purification by silica gel column chromatography (hexane/EtOAc 100:0 to 98:2) yielded the title product as a yellow solid (126 mg, 0.477 mmol, 33%): mp (hexane) 42-44°C. $^1H$ NMR (400 MHz, $CDCl_3$) δ 1.10 (s, 3 H, $CH_3$), 1.74-1.81 (m, 1 H, $CH_aH_bCH_2CH_2C(O)$), 1.90-1.95 (m, 2 H, $CH_2CH_2CH_2C(O)$), 2.03-2.09 (m, 1 H, $CH_aH_bCH_2CH_2C(O)$), 2.20-2.43 (m, 4 H, $CH_2CH_2CH_2C(O)$ + $CH_2CH$=CHAr), 6.25 (dt, *J* = 15.5, 7.6 Hz, 1 H, $CH_2CH$=CHAr), 6.59 (d, *J* = 15.9 Hz, 1 H, $CH_2CH$=C*H*Ar), 7.41-7.48 (m, 2 H, 2 x ArC*H*), 7.58 (d, *J* = 8.5 Hz, 1 H, ArC*H*), 7.69 (s, 1 H, ArC*H*), 7.79 (t, *J* = 8.2 Hz, 3 H, 3 x ArC*H*) ppm; $^{13}C$ NMR (101 MHz, $CDCl_3$) δ 18.8 ($CH_2CH_2CH_2C(O)$), 22.2 ($CH_3$), 35.3 ($CH_2CH_2CH_2C(O)$), 37.8 ($CH_2CH_2CH_2C(O)$), 40.4 ($CH_2CH$=CHAr), 48.9 (*C*), 123.6 (ArCH), 125.7 (ArCH), 125.7 (Ar*C*H), 126.1 ($CH_2CH$=*C*HAr), 126.2 (ArCH), 127.6 (Ar*C*H), 127.9 (ArCH), 128.1 (Ar*C*H), 132.8 (Ar*C*), 133.4 ($CH_2$*C*H=CHAr), 133.6 (Ar*C*), 134.8 (Ar*C*), 223.2 (*C*(O)) ppm; IR $\nu_{max}$ (thin film, $cm^{-1}$): 2959, 1732 (C=O), 1454, 1161, 1060, 966; HRMS calcd. for $C_{19}H_{21}O$ $[M+H]^+$: 265.1587, found 265.1580.

**(*E*)-2-(3-(3-Bromophenyl)allyl)-2-methylcyclopentan-1-one (3e)**

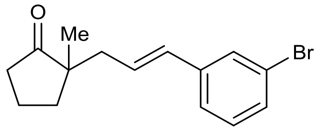


Prepared according to general procedure **C** using ketone **3a** (200 mg, 1.45 mmol), 3-bromostyrene (0.52 mL, 4.35 mmol) and Hoveyda–Grubbs 2nd Generation catalyst (18 mg, 0.029 mmol, 2 mol%). Purification by silica gel column chromatography (hexane/EtOAc 100:0 to 98:2) yielded the title product as a yellow oil (140 mg, 0.477 mmol, 33%). $^1H$ NMR (400 MHz, $CDCl_3$) δ 1.08 (s, 3 H, $CH_3$), 1.74–1.80 (m, 1 H, $CH_aH_bCH_2CH_2C(O)$), 1.88–2.04 (m, 3 H, $CH_2CH_2CH_2C(O)$ + $CH_aH_bCH_2CH_2C(O)$), 2.19–2.40 (m, 4 H, $CH_2CH_2CH_2C(O)$ + $CH_2CH$=CHAr), 6.14 (dt, *J* = 15.5, 7.6 Hz, 1 H, $CH_2CH$=CHAr), 6.36 (d, *J* = 15.9 Hz, 1 H, $CH_2CH$=C*H*Ar), 7.18 (t, *J* = 7.8 Hz, 1 H, ArC*H*), 7.26 (d, *J* = 7.8 Hz, 1 H, ArC*H*), 7.36 (d, *J* = 7.8 Hz, 1 H, ArC*H*), 7.51 (s, 1 H, ArC*H*) ppm; $^{13}C$ NMR (101 MHz, $CDCl_3$) δ 18.7 ($CH_2CH_2CH_2C(O)$), 22.1 ($CH_3$), 35.3 ($CH_2CH_2CH_2C(O)$), 37.7 ($CH_2CH_2CH_2C(O)$), 40.1 ($CH_2CH$=CHAr), 48.7 (*C*), 122.8 (Ar*C*Br),

124.8 (Ar*C*H), 127.4 ($CH_2$CH=*C*HAr), 128.9 (Ar*C*H), 130.0 (Ar*C*H), 130.1 (Ar*C*H), 131.9 ($CH_2$*C*H=CHAr), 139.5 (Ar*C*), 222.9 (*C*(O)) ppm; IR $\nu_{max}$ (thin film, $cm^{-1}$): 2959, 1732 (C=O), 1561, 1471, 1161, 1070, 965; HRMS calcd. for $C_{15}H_{21}ONBr$ $[M+NH_4]^+$: 310.0801, found 310.0801.

**(*E*)-2-(3-(2-Chlorophenyl)allyl)-2-methylcyclopentan-1-one (3f)**

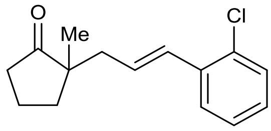


Prepared according to general procedure **C** using ketone **3a** (200 mg, 1.45 mmol), 2-chlorostyrene (0.52 mL, 4.35 mmol) and Hoveyda–Grubbs 2nd Generation catalyst (18 mg, 0.029 mmol, 2 mol%). Purification by silica gel column chromatography (hexane/EtOAc 100:0 to 98:2) yielded the title product as a yellow solid (253 mg, 1.017 mmol, 70%): mp ($CH_2Cl_2$) 36-38 °C. $^1$H NMR (400 MHz, $CDCl_3$) δ 1.08 (s, 3 H, C*H*$_3$), 1.73−1.79 (m, 1 H, C*H*$_a$H$_b$$CH_2CH_2$C(O)), 1.88−1.96 (m, 2 H, $CH_2$C*H*$_2$$CH_2$C(O)), 1.99−2.05 (m, 1 H, CH$_a$*H*$_b$$CH_2CH_2$C(O)), 2.21−2.39 (m, 4 H, $CH_2CH_2$C*H*$_2$C(O) + C*H*$_2$CH=CHAr) 6.09 (dt, *J* = 15.6, 7.7 Hz, 1 H, $CH_2$C*H*=CHAr), 6.79 (d, *J* = 15.6 Hz, 1 H, $CH_2$CH=C*H*Ar), 7.14−7.23 (m, 2 H, 2 x ArC*H*), 7.34 (d, *J* = 7.6 Hz, 1 H, ArC*H*), 7.49 (d, *J* = 7.8 Hz, 1 H, ArC*H*) ppm; $^{13}$C NMR (101 MHz, $CDCl_3$) δ 18.7 ($CH_2$*C*H$_2$$CH_2$C(O)), 22.1 (*C*H$_3$), 35.3 (*C*H$_2$$CH_2CH_2$C(O)), 37.7 ($CH_2CH_2$*C*H$_2$C(O)), 40.3 (*C*H$_2$CH=CHAr), 48.7 (*C*), 126.8 (Ar*C*H), 126.8 (Ar*C*H), 128.2 (Ar*C*H), 128.8 ($CH_2$CH=*C*HAr), 129.6 (Ar*C*H), 129.6 ($CH_2$*C*H=CHAr), 132.7 (Ar*C*Cl), 135.5 (Ar*C*), 223.0 (*C*(O)) ppm; IR $\nu_{max}$ (thin film, $cm^{-1}$): 2960, 1732 (C=O), 1469, 1440, 1162, 1033, 967; HRMS calcd. for $C_{15}H_{21}ONCl$ $[M+NH_4]^+$: 266.1306, found 266.1309.

**(*E*)-2-Methyl-2-(3-(4-(trifluoromethyl)phenyl)allyl)cyclopentan-1-one (3g)**

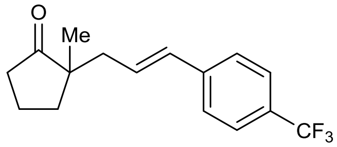


Prepared according to general procedure **C** using ketone **3a** (200 mg, 1.45 mmol), 4-(tifluoromethyl)styrene (0.52 mL, 4.35 mmol) and Hoveyda–Grubbs 2nd Generation catalyst (18 mg, 0.029 mmol, 2 mol%). Purification by silica gel column chromatography (hexane/EtOAc 100:0 to 98:2) yielded the title product as a yellow oil (123 mg, 0.435 mmol, 30%).$^1$H NMR (400 MHz, $CDCl_3$) δ 1.08 (s, 3 H, C*H*$_3$), 1.74−1.80 (m, 1 H, C*H*$_a$H$_b$$CH_2CH_2$C(O)), 1.87−2.03 (m, 3 H, $CH_2$C*H*$_2$$CH_2$C(O) + CH$_a$*H*$_b$$CH_2CH_2$C(O)), 2.18−2.40 (m, 4 H, $CH_2CH_2$C*H*$_2$C(O) + C*H*$_2$CH=CHAr), 6.23 (dt, *J* = 15.5, 7.6 Hz, 1 H, $CH_2$C*H*=CHAr), 6.45 (d, *J* = 15.9 Hz, 1 H, $CH_2$CH=C*H*Ar), 7.44 (d, *J* = 8.1 Hz, 2 H, 2 x ArC*H*), 7.55 (d, *J* = 8.3 Hz, 2 H, 2 x ArC*H*) ppm; $^{13}$C NMR (101 MHz, $CDCl_3$) δ 18.7 ($CH_2$*C*H$_2$$CH_2$C(O)), 22.1 (*C*H$_3$), 35.3

($CH_2CH_2CH_2C(O)$), 37.7 ($CH_2CH_2CH_2C(O)$), 40.2 ($CH_2CH=CHAr$), 48.7 (*C*), 122.9 (Ar*C*$CF_3$), 125.5 (q, *J* = 3.9 Hz, 2 x Ar*C*H), 126.2 (2 x Ar*C*H), 128.6 ($CH_2CH=$*C*HAr), 132.1 ($CH_2$*C*H=CHAr), 140.7 (Ar*C*), 222.8 (*C*(O)) ppm ArC$CF_3$ not observed; $^{19}$F NMR (376 MHz, $CDCl_3$) -62.5 ppm; IR $\nu_{max}$ (thin film, $cm^{-1}$): 2966, 1732 (C=O), 1614, 1413, 1324, 1264, 1163, 1122, 1066, 971; HRMS calcd. for $C_{16}H_{17}OF_3Na$ $[M+Na]^+$: 305.1124, found 305.1114.

**(*E*)-2-Methyl-2-(3-(*o*-tolyl)allyl)cyclopentan-1-one (3h)**

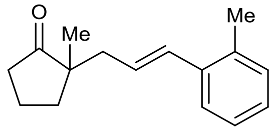

Prepared according to general procedure **C** using ketone **3a** (200 mg, 1.45 mmol), 2-methylstyrene (0.52 mL, 4.35 mmol) and Hoveyda–Grubbs 2$^{nd}$ Generation catalyst (18 mg, 0.029 mmol, 2 mol%). Purification by silica gel column chromatography (hexane/EtOAc 100:0 to 98:2) yielded the title product as a yellow oil (180 mg, 0.788 mmol, 54%). $^1$H NMR (400 MHz, $CDCl_3$) δ 1.08 (s, 3 H, $CH_3$), 1.72−1.78 (m, 1 H, $CH_aH_bCH_2CH_2C(O)$), 1.87−1.94 (m, 2 H, $CH_2CH_2CH_2C(O)$), 2.00−2.07 (m, 1 H, $CH_aH_bCH_2CH_2C(O)$), 2.20−2.39 (m, 4 H, $CH_2CH_2CH_2C(O)$ + $CH_2CH=CHAr$), 2.34 (s, 3 H, $ArCCH_3$), 5.98 (dt, *J* = 15.5, 7.6 Hz, 1 H, $CH_2CH=CHAr$), 6.62 (d, *J* = 15.4 Hz, 1 H, $CH_2CH=CHAr$), 7.15−7.19 (m, 3 H, 3 x Ar*CH*), 7.39−7.41 (m, 1 H, Ar*CH*) ppm; $^{13}$C NMR (101 MHz, $CDCl_3$) δ 18.8 ($CH_2CH_2CH_2C(O)$), 19.8 ($ArCCH_3$), 22.1 ($CH_3$), 35.2 ($CH_2CH_2CH_2C(O)$), 37.8 ($CH_2CH_2CH_2C(O)$), 40.5 ($CH_2CH=CHAr$), 48.8 (*C*), 125.6 (Ar*C*H), 126.1 (Ar*C*H), 127.0 ($CH_2CH=$*C*HAr), 127.2 (Ar*C*H), 130.2 (Ar*C*H), 131.4 ($CH_2$*C*H=CHAr), 135.1 (Ar*C*$CH_3$), 136.6 (Ar*C*), 223.2 (*C*(O)) ppm; IR $\nu_{max}$ (thin film, $cm^{-1}$): 2959, 1733 (C=O), 1456, 1161, 1060, 967; HRMS calcd. for $C_{16}H_{24}ON$ $[M+NH_4]^+$: 246.1852, found 246.1851.

## 5.2. Biocatalytic Baeyer-Villiger kinetic resolutions using CHMO

Racemic standards for GC and HPLC analysis were prepared according to literature procedures,[9] by following general procedure C, or through a procedure analogous to those described below using racemic starting materials ($SmI_2$ reactions). According to these procedures, novel compounds were obtained:

**2c**: 48%; **2e**: 37%; **2h**: 53%; **2i**: 64%; **4c**: 45%; **4d**: 37%; **4e**: 49%; **4f**: 62%; **4g**: 41%; **4h**: 34%; **6b**: 56%; **7a**: 79%; **7b**: 60%; **7c**: 52%.

**General procedure D: CHMO-Catalysed Biotransformations**

For a detailed description of the procedure for analytical scale biotransformations, see the preparation of **2a**. For a detailed description of the procedure for preparative scale biotransformations, see the preparation of **2i**.

**(*R*)-7-Allyl-7-methyloxepan-2-one (2a)**[9]

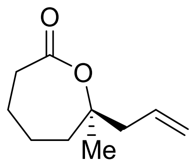

To a Falcon tube were added *rac*-**1a** (30 mg, 0.197 mmol) in EtOH (2.25 mL), NADPH (11.2 mg, 0.015 mmol), GDH (11.2 mg, 2.28 x $10^{-4}$ mmol), glucose (225 mg, 1.24 mmol), CHMO (11.2 mg, 1.86 x $10^{-4}$ mmol) and Tris/HCl buffer (12.75 mL, pH 7.0, 100 mM). The reaction was incubated at 25°C with shaking at 250 rpm for 24 hours. The mixture was extracted with EtOAc (3 x 10 mL), the organic layers were dried ($MgSO_4$) and concentrated. Purification by silica gel column chromatography (hexane/EtOAc 90:10) yielded the title product as a colourless oil (16 mg, 0.089 mmol, 47%). $^1$H NMR (400 MHz, $CDCl_3$) δ 1.44 (s, 3 H, $CH_3$), 1.62-1.70 (m, 1 H, $CH_2CH_2CH_aH_bCH_2C(O)O$), 1.76-1.88 (m, 5 H, $CH_2CH_2CH_aH_bCH_2C(O)O$ + $CH_2CH_2CH_2CH_2C(O)O$ + $CH_2CH_2CH_2CH_2C(O)O$), 2.41-2.64 (m, 2 H, $CH_2CH=CH_2$), 2.64-2.75 (m, 2 H, $CH_2CH_2CH_2CH_2C(O)O$), 5.11-5.16 (m, 2 H, $CH_2CH=CH_2$), 5.86 (ddt, *J* = 17.1, 10.1, 7.3 Hz, 1 H, $CH_2CH=CH_2$) ppm; $^{13}$C NMR (101 MHz, $CDCl_3$) δ 23.4 ($CH_2CH_2CH_2CH_2C(O)O$), 23.9 ($CH_2CH_2CH_2CH_2C(O)O$), 24.8 ($CH_3$), 37.4 ($CH_2CH_2CH_2CH_2C(O)O$), 38.4 ($CH_2CH_2CH_2CH_2C(O)O$), 46.7 ($CH_2CH=CH_2$), 82.8 (*C*), 119.0 ($CH_2CH=CH_2$), 132.8 ($CH_2CH=CH_2$), 174.7 (*C*(O)O) ppm; Specific rotation $[\alpha]_D^{30}$ -16.5 (*c* 1.2, $CHCl_3$) for an enantiomerically enriched sample of >99% e.e.; Lit[10] $[\alpha]_D^{27}$ +20.6 (*c* 3.46, hexane) for an enantiomerically enriched sampled of (S)-7-Allyl-7-methyloxepan-2-one of 98% e.e.

Enantiomeric purity of (*R*)-**2a** was determined by GC analysis in comparison with authentic racemic material (>99:1 e.r. shown; ChiraSil® DEX CB 25 m x 0.25 mm column, 50°C to 200°C at a rate of 1°C $min^{-1}$, flow rate = 1.0 mL $min^{-1}$).

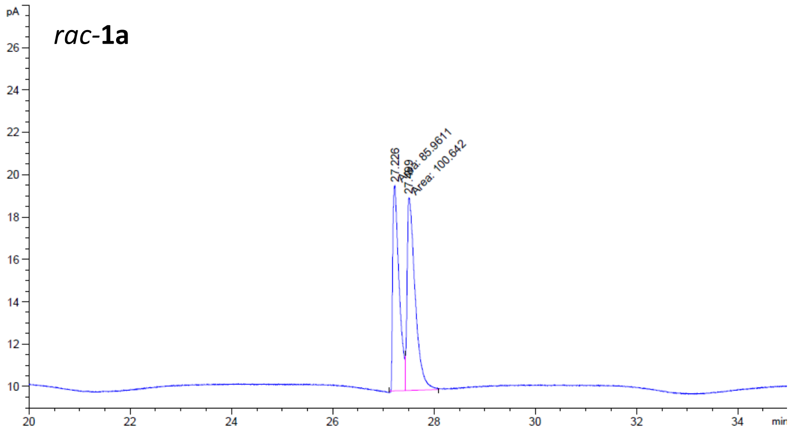


| Peak # | RetTime [min] | Type | Width [min] | Area [pA*s] | Height [pA] | Area % |
|---|---|---|---|---|---|---|
| 1 | 27.226 | MF | 0.1474 | 85.96114 | 9.72235 | 46.06622 |
| 2 | 27.499 | FM | 0.1839 | 100.64228 | 9.12023 | 53.93378 |

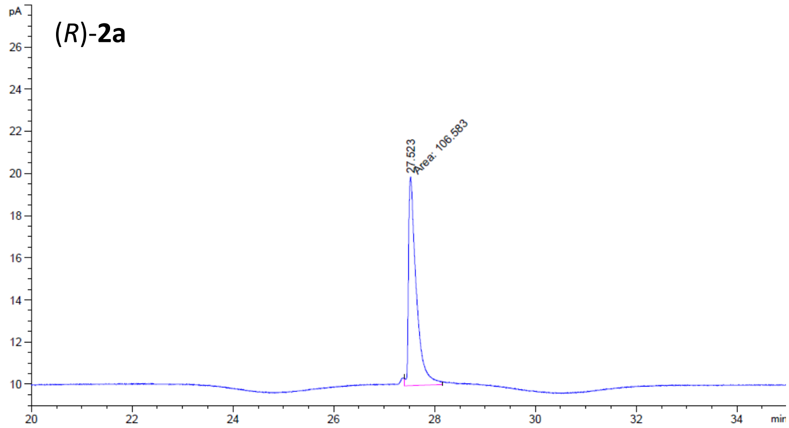


| Peak # | RetTime [min] | Type | Width [min] | Area [pA*s] | Height [pA] | Area % |
|---|---|---|---|---|---|---|
| 1 | 1.318 | BB S | 0.0240 | 3.33916e4 | 1.85779e4 | 99.68182 |
| 2 | 27.523 | MM | 0.1791 | 106.58345 | 9.91690 | 0.31818 |

### (*R,E*)-7-Cinnamyl-7-methyloxepan-2-one (2b)[9]

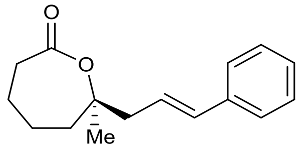


Prepared according to general procedure **D** using *rac*-**1b** (46 mg, 0.197 mmol) in EtOH (2.25 mL), NADPH (11.2 mg, 0.015 mmol), GDH (11.2 mg, 2.28 x $10^{-4}$ mmol), glucose (225 mg, 1.24 mmol), CHMO (11.2 mg, 1.86 x $10^{-4}$ mmol) and Tris/HCl buffer (12.75 mL, pH 7.0, 100 mM). Purification by silica gel column chromatography (hexane/EtOAc 90:10) yielded the title product as a colourless oil (20 mg, 0.08 mmol, 41%). $^{1}$H NMR (400 MHz, $CDCl_3$) δ 1.50 (s, 3 H, $CH_3$), 1.60-1.71 (m, 1 H, $CH_2CH_2CH_aH_bCH_2C(O)O$), 1.80-1.92 (m, 5 H, $CH_2CH_2CH_aH_bCH_2C(O)O$ + $CH_2CH_2CH_2CH_2C(O)O$ +

C*H*$_2$CH$_2$CH$_2$CH$_2$C(O)O)), 2.57-2.82 (m, 4 H, C*H*$_2$CH=CHAr + CH$_2$CH$_2$CH$_2$C*H*$_2$C(O)O)), 6.28 (dt, *J* = 15.6, 7.6 Hz, 1 H, CH$_2$C*H*=CHAr), 6.47 (d, *J* = 15.9 Hz, 1 H, CH$_2$CH=C*H*Ar), 7.22-7.25 (m, 1 H, ArC*H*), 7.32 (t, *J* = 7.4 Hz, 2 H, 2 x ArC*H*), 7.37-7.39 (m, 2 H, 2 x ArC*H*) ppm; $^{13}$C NMR δ 23.4 (CH$_2$CH$_2$*C*H$_2$CH$_2$C(O)O), 24.0 (CH$_2$*C*H$_2$CH$_2$CH$_2$C(O)O), 24.7 (*C*H$_3$), 37.4 (CH$_2$CH$_2$CH$_2$*C*H$_2$C(O)O), 38.6 (*C*H$_2$CH$_2$CH$_2$CH$_2$C(O)O), 46.4 (*C*H$_2$CH=CHAr), 83.2 (*C*), 124.4 (CH$_2$*C*H=CHAr), 126.2 (2 x Ar*C*H), 127.4 (Ar*C*H), 128.6 (2 x Ar*C*H), 134.0 (CH$_2$CH=*C*HAr), 137.1 (Ar*C*), 174.8 (*C*(O)O) ppm; Specific rotation $[\alpha]_D^{30}$ -10.6 (*c* 1.4, CHCl$_3$) for an enantiomerically enriched sample of >99% e.e.

Enantiomeric purity of (*R*)-**2b** was determined by HPLC analysis in comparison with authentic racemic material (>99:1 e.r. shown; Lux 5 µm Amylose-1 column, 80:20 hexanes:$^i$PrOH, 1.0 mL min$^{-1}$, 20°C, 254 nm).

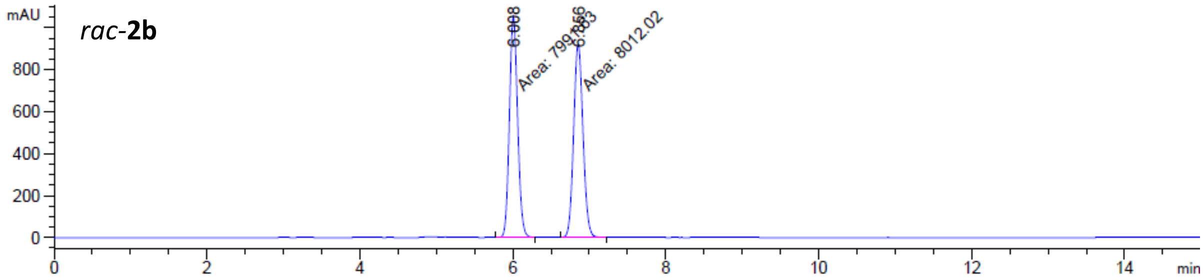


| Peak # | RetTime [min] | Type | Width [min] | Area [mAU*s] | Height [mAU] | Area % |
|---|---|---|---|---|---|---|
| 1 | 6.008 | MM | 0.1262 | 7991.62842 | 1055.66541 | 49.9363 |
| 2 | 6.856 | MM | 0.1444 | 8012.01563 | 924.84979 | 50.0637 |

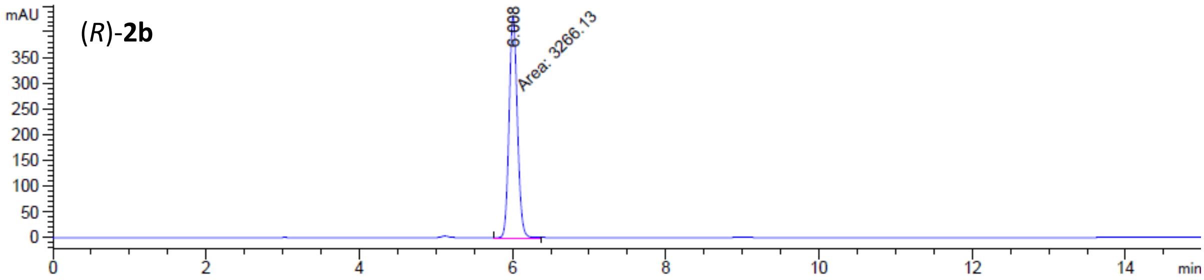


| Peak # | RetTime [min] | Type | Width [min] | Area [mAU*s] | Height [mAU] | Area % |
|---|---|---|---|---|---|---|
| 1 | 6.008 | MM | 0.1262 | 3266.13013 | 431.20129 | 100.0000 |

Absolute stereochemistry was assigned by comparison with a sample of (*S*,*E*)-7-cinnamyl-7-methyloxepan-2-one[6] (92:8 e.r. shown).

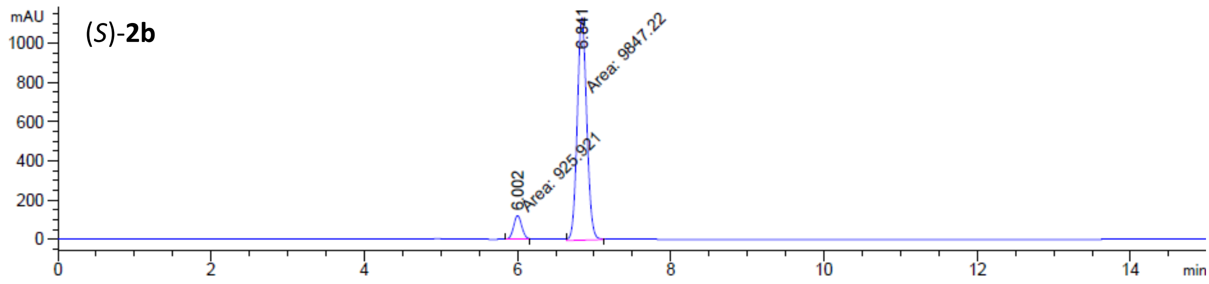


| Peak # | RetTime [min] | Type | Width [min] | Area [mAU*s] | Height [mAU] | Area % |
|---|---|---|---|---|---|---|
| 1 | 6.002 | MM | 0.1205 | 931.14288 | 128.81091 | 8.2886 |
| 2 | 6.841 | MM | 0.1448 | 1.03029e4 | 1186.07056 | 91.7114 |

The remaining starting material from the reaction could be isolated and was obtained as a pale yellow oil (18 mg, 0.079 mmol, 39%). Enantiomeric purity of (*S*)-**1b** was determined by HPLC analysis in comparison with authentic racemic material (>99:1 e.r. shown, Chiralpak® 5 µm IA column, 99.5:0.5 hexanes:$^{i}$PrOH, 0.6 mL min$^{-1}$, 20°C, 254 nm)

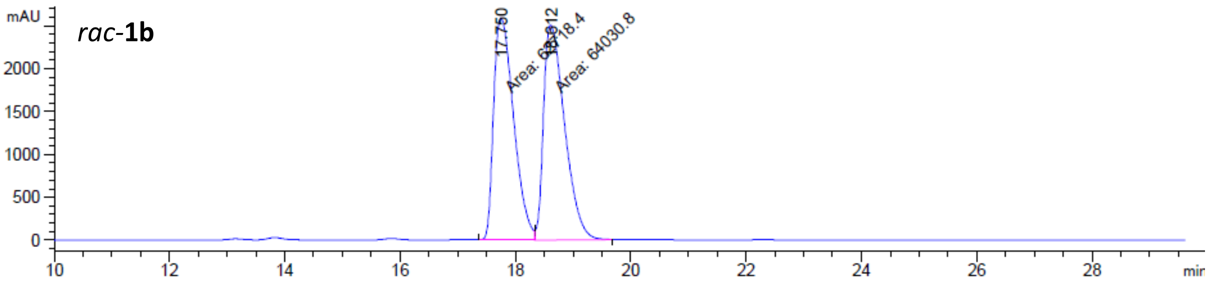


| Peak # | RetTime [min] | Type | Width [min] | Area [mAU*s] | Height [mAU] | Area % |
|---|---|---|---|---|---|---|
| 1 | 17.750 | MF | 0.4030 | 6.27184e4 | 2593.76685 | 49.4823 |
| 2 | 18.612 | FM | 0.4255 | 6.40308e4 | 2508.12891 | 50.5177 |

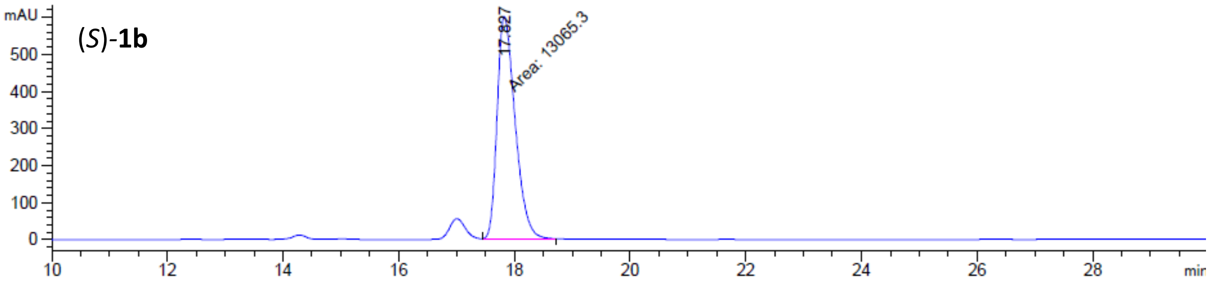


| Peak # | RetTime [min] | Type | Width [min] | Area [mAU*s] | Height [mAU] | Area % |
|---|---|---|---|---|---|---|
| 1 | 17.827 | MM | 0.3621 | 1.30653e4 | 601.42206 | 100.0000 |

**(*R,E*)-7-(3-(4-Fluorophenyl)allyl)-7-methyloxepan-2-one (2c)**

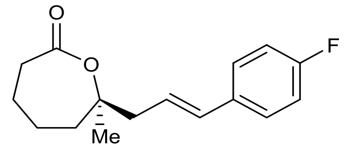


Prepared according to general procedure **D** using *rac*-**1c** (49 mg, 0.197 mmol) in EtOH (2.25 mL), NADPH (11.2 mg, 0.015 mmol), GDH (11.2 mg, 2.28 x $10^{-4}$ mmol), glucose (225 mg, 1.24 mmol), CHMO (11.2 mg, 1.86 x $10^{-4}$ mmol) and Tris/HCl buffer (12.75 mL, pH 7.0, 100 mM). Purification by silica gel column chromatography (hexane/EtOAc 90:10) yielded the title product as a colourless oil (19 mg, 0.071 mmol, 32%). $^{1}$H NMR (400 MHz, $CDCl_3$) δ 1.49 (s, 3 H, $CH_3$), 1.65-1.71 (m, 1 H, $CH_2CH_2CH_aH_bCH_2C(O)O$)), 1.80-1.91 (m, 5 H, $CH_2CH_2CH_aH_bCH_2C(O)O$ + $CH_2CH_2CH_2CH_2C(O)O$ + $CH_2CH_2CH_2CH_2C(O)O$)), 2.54-2.79 (m, 4 H, $CH_2CH$=CHAr + $CH_2CH_2CH_2CH_2C(O)O$)), 6.20 (dt, *J* = 15.8, 8.2 Hz, 1 H, $CH_2CH$=CHAr), 6.42 (d, *J* = 15.8 Hz, 1 H, $CH_2CH$=C*H*Ar), 7.00 (t, *J* = 8.7 Hz, 2 H, 2 x ArC*H*), 7.32-7.36 (m, 2 H, 2 x ArC*H*) ppm; $^{13}$C NMR (101 MHz, $CDCl_3$) δ 23.4 ($CH_2CH_2CH_2CH_2C(O)O$), 24.0 ($CH_2CH_2CH_2CH_2C(O)O$), 24.7 (*C*$H_3$), 37.4 ($CH_2CH_2CH_2CH_2C(O)O$), 38.7 ($CH_2CH_2CH_2CH_2C(O)O$), 46.4 (*C*$H_2$CH=CHAr), 83.1 (*C*), 115.4 (2 x Ar*C*H), 124.2 ($CH_2$*C*H=CHAr), 127.7 (2 x Ar*C*H), 132.7 ($CH_2$CH=*C*HAr), 133.3 (Ar*C*), 162.2 (d, *J* = 246.5 Hz, Ar*C*F), 174.8 (*C*(O)O) ppm; IR $\nu_{max}$ (thin film, $cm^{-1}$): 2933, 1711 (C=O), 1507, 1333, 1224, 1178, 1103, 1017; HRMS calcd. for $C_{16}H_{19}O_2FNa$ $[M+Na]^+$: 285.1261, found 285.1255. Specific rotation $[\alpha]_D^{30}$ -11.5 (*c* 1.2, $CHCl_3$) for an enantiomerically enriched sample of >99% e.e.

Enantiomeric purity of (*R*)-**2c** was determined by HPLC analysis in comparison with authentic racemic material (>99:1 e.r. shown; Lux 5 µm Amylose-1 column, 80:20 hexanes:$^{i}$PrOH, 1.0 mL $min^{-1}$, 20°C, 254 nm).

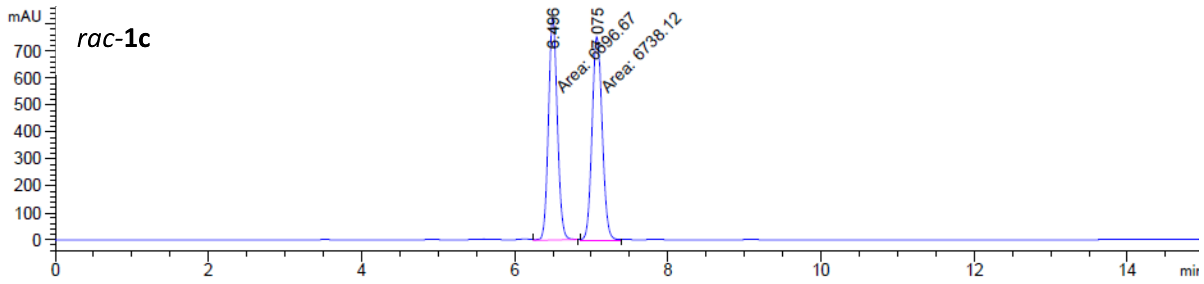


| Peak # | RetTime [min] | Type | Width [min] | Area [mAU*s] | Height [mAU] | Area % |
|---|---|---|---|---|---|---|
| 1 | 6.496 | MM | 0.1355 | 6696.66602 | 823.67255 | 49.8457 |
| 2 | 7.075 | MM | 0.1487 | 6738.11865 | 755.04071 | 50.1543 |

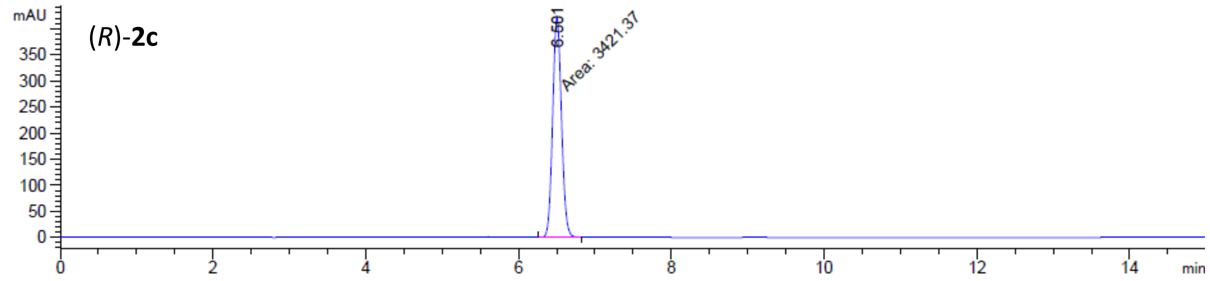


| Peak # | RetTime [min] | Type | Width [min] | Area [mAU*s] | Height [mAU] | Area % |
|---|---|---|---|---|---|---|
| 1 | 6.501 | MM | 0.1349 | 3421.37012 | 422.57538 | 100.0000 |

Absolute stereochemistry was assigned by comparison with a sample of (*S,E*)-7-(3-(4-fluorophenyl)allyl)-7-methyloxepan-2-one[6] (92:8 e.r. shown).

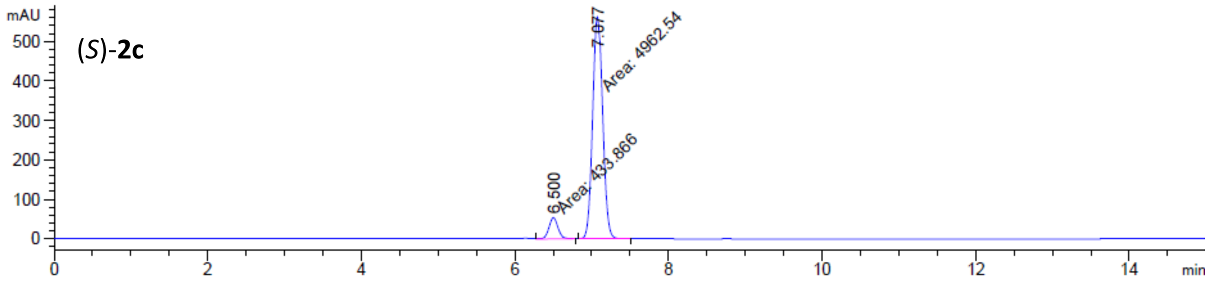


| Peak # | RetTime [min] | Type | Width [min] | Area [mAU*s] | Height [mAU] | Area % |
|---|---|---|---|---|---|---|
| 1 | 6.500 | MM | 0.1356 | 433.86572 | 53.32559 | 8.0399 |
| 2 | 7.077 | MM | 0.1471 | 4962.53809 | 562.26251 | 91.9601 |

**(*R,E*)-7-Methyl-7-(3-(naphthalen-2-yl)allyl)oxepan-2-one (2d)**[9]

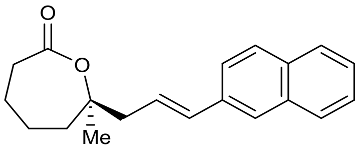


Prepared according to general procedure **D** using *rac*-**1d** (6 mg, 0.02 mmol), NADPH in EtOH (300 µL), NADPH (1.5 mg, 0.002 mmol), GDH (1.5 mg, 3.04 x $10^{-5}$ mmol), glucose (30 mg, 0.167 mmol), CHMO (1.5 mg, 2.46 x $10^{-5}$ mmol) and Tris/HCl buffer (2.7 mL, pH 7.0, 100 mM). Purification by silica gel column chromatography yielded the title product as a colourless oil (0.8 mg, 0.002 mmol, 13%). $^1$H NMR (400 MHz, CDCl$_3$) δ 1.53 (s, 3 H, C*H$_3$*), 1.67-1.73 (m, 1 H, CH$_2$CH$_2$C*H$_a$*H$_b$CH$_2$C(O)O)), 1.81-1.95 (m, 5 H, CH$_2$CH$_2$CH$_a$*H$_b$*CH$_2$C(O)O + CH$_2$C*H$_2$*CH$_2$CH$_2$C(O)O + C*H$_2$*CH$_2$CH$_2$CH$_2$C(O)O)), 2.63-2.83 (m, 4 H, C*H$_2$*CH=CHAr + CH$_2$CH$_2$CH$_2$C*H$_2$*C(O)O), 6.41 (dt, *J* = 15.8, 8.0 Hz, 1 H, CH$_2$C*H*=CHAr), 6.63 (d, *J* = 15.8 Hz, 1 H, CH$_2$CH=C*H*Ar), 7.42-7.48 (m, 2 H, 2 x ArC*H*), 7.61 (dd, *J* = 8.5, 1.3 Hz, 1 H, ArC*H*), 7.72 (s, 1 H, ArC*H*) 7.78-7.81 (m, 3 H, 3 x ArC*H*) ppm; $^{13}$C NMR (101 MHz, CDCl$_3$) δ 23.4 (CH$_2$CH$_2$*C*H$_2$CH$_2$C(O)O),

24.0 ($CH_2$*C*$H_2CH_2CH_2$C(O)O), 24.7 (*C*$H_3$), 37.4 ($CH_2CH_2CH_2$*C*$H_2$C(O)O), 38.6 (*C*$H_2CH_2CH_2CH_2$C(O)O), 46.6 (*C*$H_2$CH=CHAr), 83.2 (*C*), 123.6 (Ar*C*H), 124.9 ($CH_2$*C*H=CHAr), 125.8 (Ar*C*H), 125.9 (Ar*C*H), 126.2 (Ar*C*H), 127.6 (Ar*C*H), 127.9 (Ar*C*H), 128.2 (Ar*C*H), 132.9 (Ar*C*), 133.6 (Ar*C*), 134.1 $CH_2$CH=*C*HAr, 134.5 (Ar*C*), 174.8 (C(O)O) ppm. Specific rotation $[\alpha]_D^{30}$ +23.1 (*c* 0.14, $CHCl_3$) for an enantiomerically enriched sample of >99% e.e.

Enantiomeric purity of (*R*)-**2d** was determined by HPLC analysis in comparison with authentic racemic material (>99:1 e.r. shown; Lux 5 µm Amylose-1 column, 80:20 hexanes:$^i$PrOH, 1.0 mL min$^{-1}$, 20°C, 254 nm).

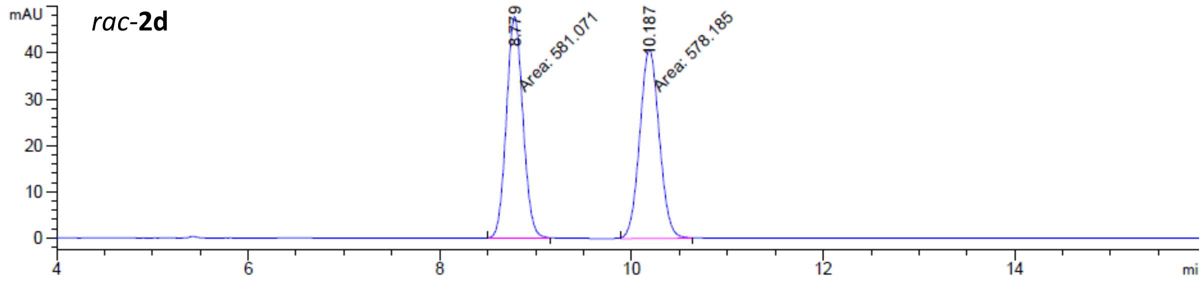


| Peak # | RetTime [min] | Type | Width [min] | Area [mAU*s] | Height [mAU] | Area % |
|---|---|---|---|---|---|---|
| 1 | 8.779 | MM | 0.2019 | 581.07123 | 47.96709 | 50.1245 |
| 2 | 10.187 | MM | 0.2359 | 578.18475 | 40.85089 | 49.8755 |

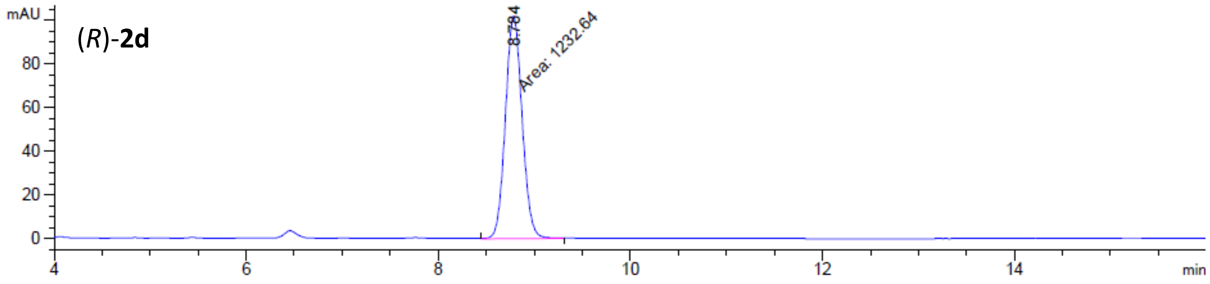


| Peak # | RetTime [min] | Type | Width [min] | Area [mAU*s] | Height [mAU] | Area % |
|---|---|---|---|---|---|---|
| 1 | 8.784 | MM | 0.2019 | 1232.64209 | 101.75729 | 100.0000 |

**(*R,E*)-7-(3-(3-Bromophenyl)allyl)-7-methyloxepan-2-one (2e)**

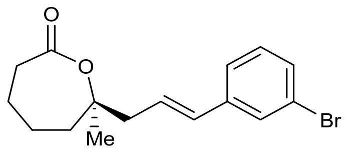


Prepared according to general procedure **D** using *rac*-**1e** (61 mg, 0.197 mmol) in EtOH (2.25 mL), NADPH (11.2 mg, 0.015 mmol), GDH (11.2 mg, 2.28 x $10^{-4}$ mmol), glucose (225 mg, 1.24 mmol), CHMO (11.2 mg, 1.86 x $10^{-4}$ mmol) and Tris/HCl buffer (12.75 mL, pH 7.0, 100 mM). Purification by

silica gel column chromatography (hexane/EtOAc 90:10) yielded the title product as a colourless oil (7 mg, 0.021 mmol, 11%). $^{1}$H NMR (400 MHz, $CDCl_3$) δ 1.49 (s, 3 H, $CH_3$), 1.65-1.70 (m, 1 H, $CH_2CH_2CH_aH_bCH_2C(O)O)$), 1.79-1.90 (m, 5 H, $CH_2CH_2CH_aH_bCH_2C(O)O$ + $CH_2CH_2CH_2CH_2C(O)O$ + $CH_2CH_2CH_2CH_2C(O)O)$), 2.55-2.81 (m, 4 H, $CH_2CH$=CHAr + $CH_2CH_2CH_2CH_2C(O)O$), 6.29 (dt, *J* = 15.8, 8.3 Hz, 1 H, $CH_2CH$=CHAr), 6.39 (d, *J* = 15.8 Hz, 1 H, $CH_2CH$=C*H*Ar), 7.17 (t, *J* = 8.0 Hz, 1 H, ArC*H*), 7.28 (d, *J* = 8.3 Hz, 1 H, ArC*H*), 7.35 (d, *J* = 7.8 Hz, 1 H, ArC*H*), 7.52 (s, 1 H, ArC*H*) ppm; $^{13}$C NMR (101 MHz, $CDCl_3$) δ 23.4 ($CH_2CH_2CH_2CH_2C(O)O$), 24.0 ($CH_2CH_2CH_2CH_2C(O)O$), 24.6 ($CH_3$), 37.4 ($CH_2CH_2CH_2CH_2C(O)O$), 38.7 ($CH_2CH_2CH_2CH_2C(O)O$), 46.5 ($CH_2CH$=CHAr), 82.9 (*C*), 122.8 (Ar*C*Br), 124.9 (ArCH), 126.2 ($CH_2CH$=CHAr), 129.1 (Ar*C*H), 130.0 (ArCH), 130.2 (Ar*C*H), 132.5 ($CH_2CH$=*C*HAr), 139.2 (Ar*C*), 174.7 (*C*(O)O) ppm; IR $\nu_{max}$ (thin film, $cm^{-1}$): 2933, 1711 (C=O), 1590, 1471, 1289, 1178, 1109, 1017; HRMS calcd. for $C_{16}H_{19}O_2BrNa$ $[M+Na]^+$: 345.0461, found 345.0454. Specific rotation $[\alpha]_D^{30}$ -2.2 (*c* 0.6, $CHCl_3$) for an enantiomerically enriched sample of >99% e.e.

Enantiomeric purity of (*R*)-**2e** was determined by HPLC analysis in comparison with authentic racemic material (>99:1 e.r. shown; Lux 5 µm Amylose-1 column, 80:20 hexanes:$^{i}$PrOH, 1.0 mL/min, 20°C, 254 nm).

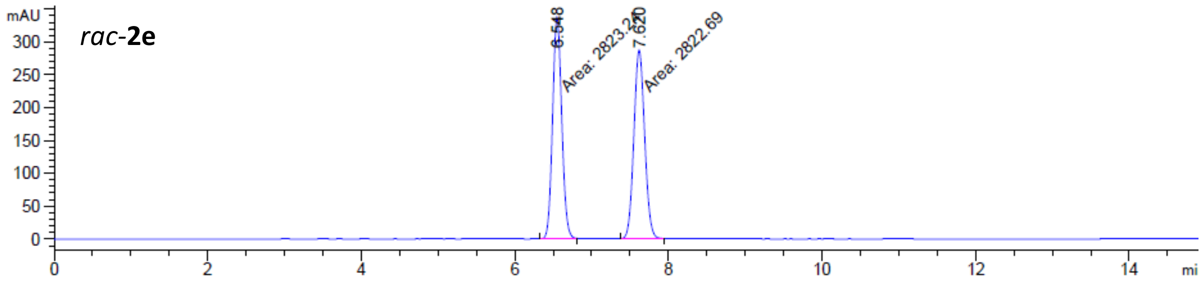


| Peak # | RetTime [min] | Type | Width [min] | Area [mAU*s] | Height [mAU] | Area % |
|---|---|---|---|---|---|---|
| 1 | 6.548 | MM | 0.1396 | 2823.24170 | 337.08838 | 50.0049 |
| 2 | 7.620 | MM | 0.1641 | 2822.69043 | 286.61276 | 49.9951 |

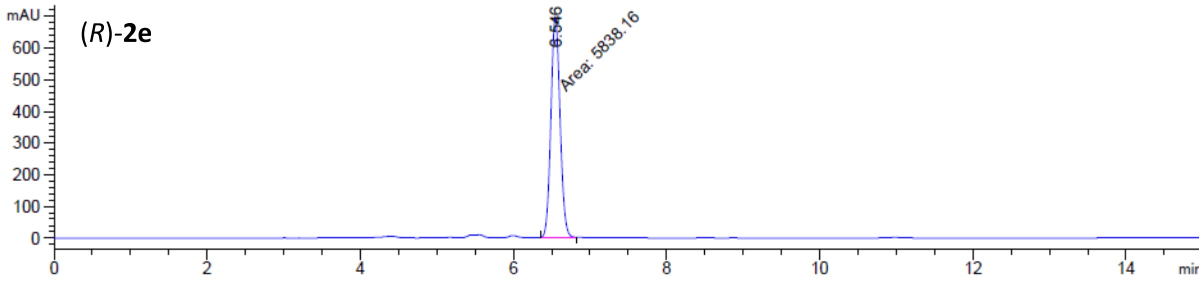


| Peak # | RetTime [min] | Type | Width [min] | Area [mAU*s] | Height [mAU] | Area % |
|---|---|---|---|---|---|---|
| 1 | 6.546 | MM | 0.1396 | 5838.15820 | 696.94080 | 100.0000 |

**(*R,E*)-7-(3-(2-Chlorophenyl)allyl)-7-methyloxepan-2-one (2f)**[9]

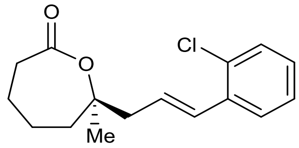

Prepared according to general procedure **D** using *rac*-**1f** (52 mg, 0.197 mmol) in EtOH (2.25 mL), NADPH (11.2 mg, 0.015 mmol), GDH (11.2 mg, $2.28 \times 10^{-4}$ mmol), glucose (225 mg, 1.24 mmol), CHMO (11.2 mg, $1.86 \times 10^{-4}$ mmol) and Tris/HCl buffer (12.75 mL, pH 7.0, 100 mM). Purification by silica gel column chromatography (hexane/EtOAc 90:10) yielded the title product as a colourless oil (20 mg, 0.071 mmol, 26%). $^1$H NMR (400 MHz, $CDCl_3$) δ 1.53 (s, 3 H, $CH_3$), 1.65-1.73 (m, 1 H, $CH_2CH_2CH_aH_bCH_2C(O)O$)), 1.81-1.95 (m, 5 H, $CH_2CH_2CH_aH_bCH_2C(O)O$ + $CH_2CH_2CH_2CH_2C(O)O$ + $CH_2CH_2CH_2CH_2C(O)O$)), 2.62-2.83 (m, 4 H, $CH_2CH=CHAr$ + $CH_2CH_2CH_2CH_2C(O)O$), 6.30 (dt, *J* = 15.7, 7.4 Hz, 1 H, $CH_2CH=CHAr$), 6.86 (d, *J* = 15.9 Hz, 1 H, $CH_2CH=CHAr$), 7.19-7.26 (m, 2 H, 2 x Ar*CH*), 7.36 (dd, *J* = 7.8, 1.5 Hz, 1 H, Ar*CH*), 7.56 (dd, *J* = 7.6, 1.7 Hz, 1 H, Ar*CH*) ppm; $^{13}$C NMR (101 MHz, $CDCl_3$) δ 23.4 ($CH_2CH_2CH_2CH_2C(O)O$), 24.0 ($CH_2CH_2CH_2CH_2C(O)O$), 24.8 ($CH_3$), 37.4 ($CH_2CH_2CH_2CH_2C(O)O$), 38.7 ($CH_2CH_2CH_2CH_2C(O)O$), 46.5 ($CH_2CH=CHAr$), 83.0 (*C*), 126.8 (Ar*C*H), 126.9 (Ar*C*H), 127.5 ($CH_2CH=CHAr$), 128.4 (Ar*C*H), 129.6 (Ar*C*H), 130.2 ($CH_2CH=CHAr$), 132.7 (Ar*C*Cl), 135.3 (Ar*C*), 174.7 (*C*(O)O) ppm; Specific rotation $[\alpha]_D^{30}$ +11.7 (*c* 0.9, $CHCl_3$) for an enantiomerically enriched sample of >99% e.e.

Enantiomeric purity of (*R*)-**2f** was determined by HPLC analysis in comparison with authentic racemic material (>99:1 e.r. shown; Lux 5 µm Amylose-1 column, 80:20 hexanes:$^i$PrOH, 1.0 mL min$^{-1}$, 20°C, 254 nm).

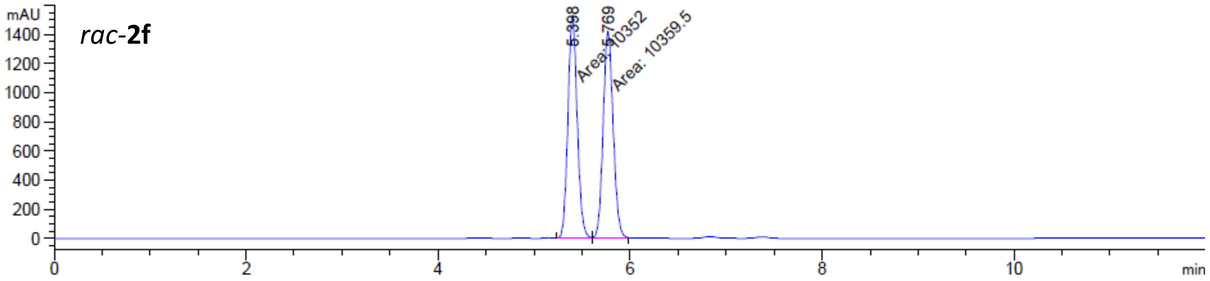


| Peak # | RetTime [min] | Type | Width [min] | Area [mAU*s] | Height [mAU] | Area % |
|---|---|---|---|---|---|---|
| 1 | 5.398 | MM | 0.1137 | 1.04823e4 | 1536.05017 | 50.0999 |
| 2 | 5.769 | MM | 0.1214 | 1.04405e4 | 1432.78601 | 49.9001 |

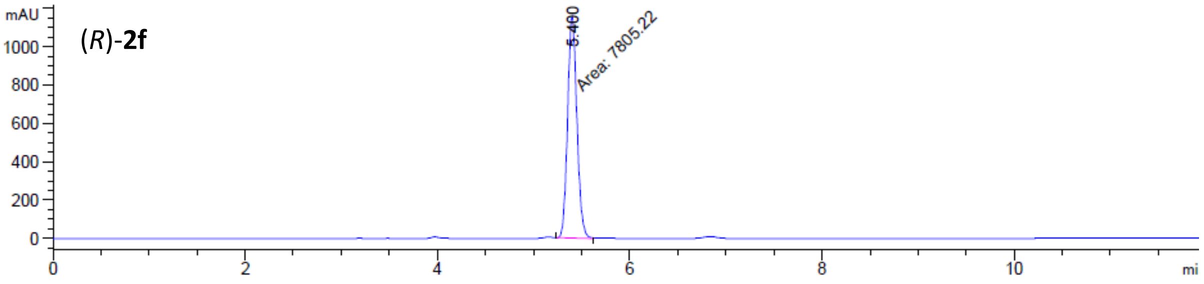


| Peak # | RetTime [min] | Type | Width [min] | Area [mAU*s] | Height [mAU] | Area % |
|---|---|---|---|---|---|---|
| 1 | 5.400 | MM | 0.1124 | 7805.22314 | 1156.92249 | 100.0000 |

**(*R,E*)-7-Methyl-7-(3-(4-(trifluoromethyl)phenyl)allyl)oxepan-2-one (2g)**[9]

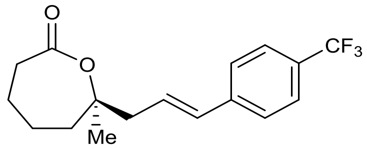


Prepared according to general procedure **D** using *rac*-**1g** (59 mg, 0.197 mmol) in EtOH (2.25 mL), NADPH (11.2 mg, 0.015 mmol), GDH (11.2 mg, 2.28 x $10^{-4}$ mmol), glucose (225 mg, 1.24 mmol), CHMO (11.2 mg, 1.86 x $10^{-4}$ mmol) and Tris/HCl buffer (12.75 mL, pH 7.0, 100 mM). Purification by silica gel column chromatography (hexane/EtOAc 90:10) yielded the title product as a colourless oil (17 mg, 0.05 mmol, 27%). $^{1}$H NMR (400 MHz, $CDCl_3$) δ 1.51 (s, 3 H, $CH_3$), 1.65-1.70 (m, 1 H, $CH_2CH_2CH_aH_bCH_2C(O)O$), 1.81-1.92 (m, 5 H, $CH_2CH_2CH_aH_bCH_2C(O)O$ + $CH_2CH_2CH_2CH_2C(O)O$ + $CH_2CH_2CH_2CH_2C(O)O$), 2.57-2.82 (m, 4 H, $CH_2$CH=CHAr + $CH_2CH_2CH_2CH_2C(O)O$), 6.41 (dt, *J* = 15.9, 7.1 Hz, 1 H, $CH_2CH$=CHAr), 6.50 (d, *J* = 16.1 Hz, 1 H, $CH_2$CH=C*H*Ar), 7.47 (d, *J* = 8.1 Hz, 2 H, 2 x ArC*H*), 7.56 (d, *J* = 8.3 Hz, 2 H, 2 x ArC*H*) ppm; $^{13}$C NMR (101 MHz, $CDCl_3$) δ 23.3 ($CH_2CH_2CH_2CH_2C(O)O$), 24.0 ($CH_2CH_2CH_2CH_2C(O)O$), 24.5 ($CH_3$), 37.4 ($CH_2CH_2CH_2CH_2C(O)O$), 38.8 ($CH_2CH_2CH_2CH_2C(O)O$), 46.7 ($CH_2$CH=CHAr), 82.9 (*C*), 125.5 (q, *J* = 3.9 Hz, 2 x Ar*C*H), 126.3 (2 x Ar*C*H), 127.4 ($CH_2$*C*H=CHAr), 132.6 ($CH_2$CH=*C*HAr), 140.5 (Ar*C*), 174.6 (*C*(O)O) ppm, Ar*C*$CF_3$ and ArC*C*$F_3$ not observed. Specific rotation $[\alpha]_D^{30}$ -10.2 (*c* 0.6, $CHCl_3$) for an enantiomerically enriched sample of >99% e.e.

Enantiomeric purity of (*R*)-**2g** was determined by HPLC analysis in comparison with authentic racemic material (>99:1 e.r. shown; Lux 5 µm Amylose-1 column, 85:15 hexanes:$^{i}$PrOH, 0.7 mL min$^{-1}$, 20°C, 254 nm).

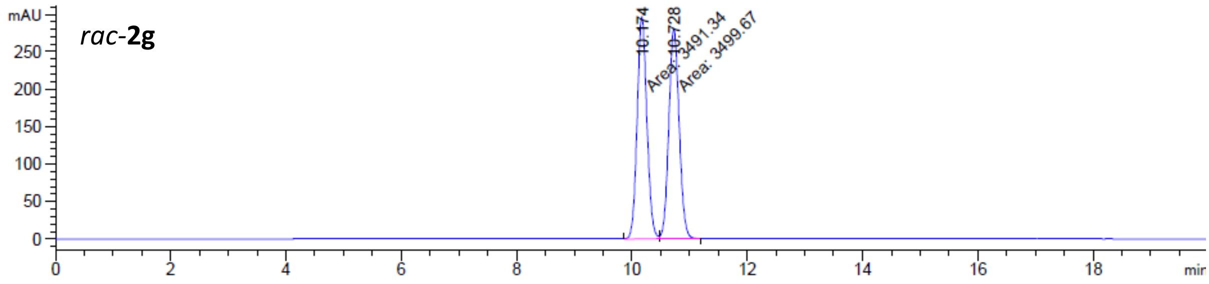


| Peak # | RetTime [min] | Type | Width [min] | Area [mAU*s] | Height [mAU] | Area % |
|---|---|---|---|---|---|---|
| 1 | 10.174 | MF | 0.1964 | 3491.33643 | 296.26495 | 49.9404 |
| 2 | 10.728 | FM | 0.2079 | 3499.67163 | 280.58661 | 50.0596 |

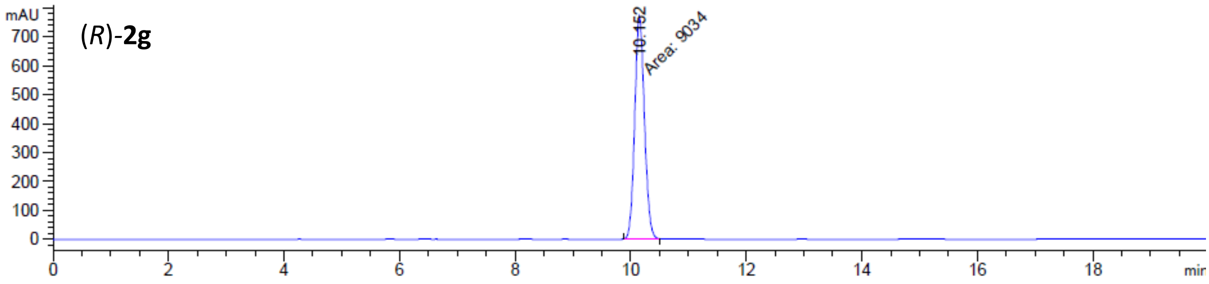


| Peak # | RetTime [min] | Type | Width [min] | Area [mAU*s] | Height [mAU] | Area % |
|---|---|---|---|---|---|---|
| 1 | 10.152 | MM | 0.1958 | 9034.00488 | 768.79077 | 100.0000 |

### (*R,E*)-7-Methyl-7-(3-(o-tolyl)allyl)oxepan-2-one (2h)

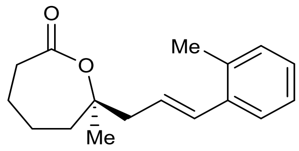


Prepared according to general procedure **D** using *rac*-**1h** (6 mg, 0.023 mmol), NADPH in EtOH (300 µL), NADPH (1.5 mg, 0.002 mmol), GDH (1.5 mg, 3.04 x $10^{-5}$ mmol), glucose (30 mg, 0.167 mmol), CHMO (1.5 mg, 2.46 x $10^{-5}$ mmol) and Tris/HCl buffer (2.7 mL, pH 7.0, 100 mM). Purification by silica gel column chromatography (hexane/EtOAc 90:10) yielded the title product as a colourless oil (1.4 mg, 0.006 mmol, 24%). $^1$H NMR (400 MHz, $CDCl_3$) δ 1.51 (s, 3 H, $CH_3$) 1.66-1.72 (m, 1 H, $CH_2CH_2CH_aH_bCH_2C(O)O$)),), 1.80-1.93 (m, 5 H, $CH_2CH_2CH_aH_bCH_2C(O)O$ + $CH_2CH_2CH_2CH_2C(O)O$ + $CH_2CH_2CH_2CH_2C(O)O$), 2.35 (s, 3 H, $ArCCH_3$), 2.61-2.82 (m, 4 H, $CH_2CH$=CHAr + $CH_2CH_2CH_2CH_2C(O)O$), 6.13 (dt, *J* = 15.8, 7.5 Hz, 1 H, $CH_2CH$=CHAr), 6.68 (d, *J* = 15.8 Hz, 1 H, $CH_2CH$=CHAr), 7.14-7.18 (m, 3 H, 3 x ArC*H*), 7.43-7.45 (m, 1 H, ArC*H*) ppm; $^{13}$C NMR (101 MHz, $CDCl_3$) δ 19.8 ($ArCCH_3$), 23.4 ($CH_2CH_2CH_2CH_2C(O)O$), 24.0 ($CH_2CH_2CH_2CH_2C(O)O$), 24.9 ($CH_3$), 37.4 ($CH_2CH_2CH_2CH_2C(O)O$), 38.6 ($CH_2CH_2CH_2CH_2C(O)O$) 46.5 ($CH_2CH$=CHAr), 83.1 (*C*), 125.7 (ArCH), 125.8 ($CH_2CH$=CHAr), 126.1 (ArCH), 127.4 (ArCH), 130.2 (Ar*C*H), 132.0 ($CH_2CH$=*C*HAr), 135.1 ($ArCCH_3$), 136.3 (Ar*C*), 174.7 (*C*(O)O)

ppm; IR $\nu_{max}$ (thin film, $cm^{-1}$): 2932, 2860, 1711 (C=O), 1458, 1352, 1286, 1177, 1087, 1017; HRMS calcd. for $C_{17}H_{22}O_2Na$ $[M+Na]^+$: 281.1512, found 281.1503. Specific rotation $[\alpha]_D^{30}$ -12.8 (*c* 0.1, $CHCl_3$) for an enantiomerically enriched sample of >99% e.e.

Enantiomeric purity of (*R*)-**2h** was determined by HPLC analysis in comparison with authentic racemic material (>99:1 e.r. shown; Lux 5 µm Amylose-1 column, 85:15 hexanes:$^i$PrOH, 0.7 mL $min^{-1}$, 20°C, 254 nm).

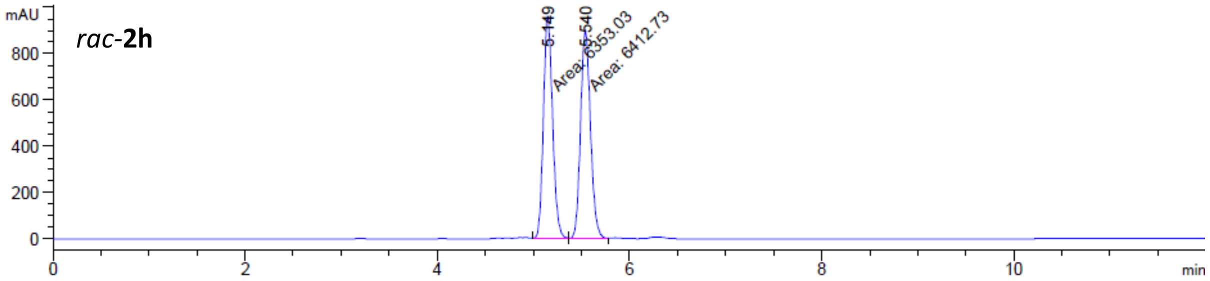


| Peak # | RetTime [min] | Type | Width [min] | Area [mAU*s] | Height [mAU] | Area % |
|---|---|---|---|---|---|---|
| 1 | 5.149 | MF | 0.1102 | 6353.02637 | 961.07758 | 49.7661 |
| 2 | 5.540 | FM | 0.1192 | 6412.73242 | 896.73645 | 50.2339 |

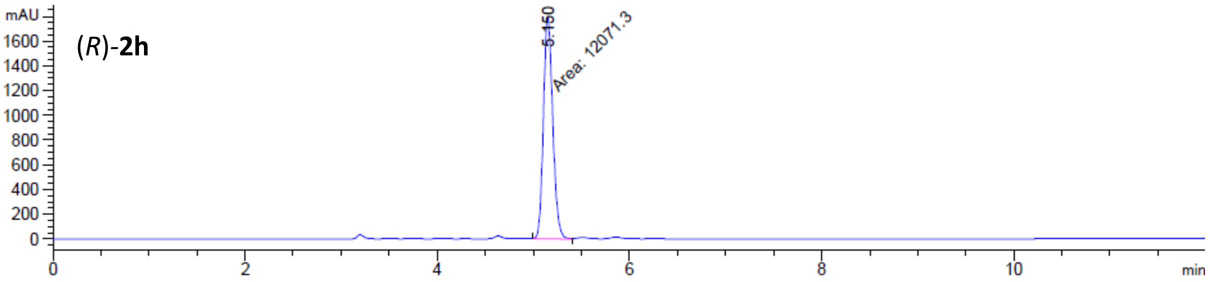


| Peak # | RetTime [min] | Type | Width [min] | Area [mAU*s] | Height [mAU] | Area % |
|---|---|---|---|---|---|---|
| 1 | 5.150 | MM | 0.1116 | 1.20713e4 | 1803.09875 | 100.0000 |

**(*R*)-7-Allyl-7-ethyloxepan-2-one (2i)**

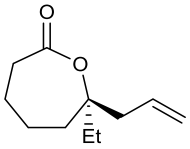


Ketone *rac*-**1i** was subjected to the conditions in general procedure **D** using *rac*-**1i** (1 mg, 0.006 mmol), NADPH in EtOH (50 µL), NADPH (0.25 mg, 3.36 x $10^{-4}$ mmol), GDH (0.25 mg, 5.08 x $10^{-6}$ mmol), glucose (0.5 mg, 0.003 mmol), CHMO (0.25 mg, 4.10 x $10^{-6}$ mmol) and Tris/HCl buffer (450 µL, pH 7.0, 100 mM). The reaction mixture was extracted with EtOAc (500 µL) and analysed directly by GC. $^1$H NMR (400 MHz, $CDCl_3$) δ 0.96 (t, *J* = 7.5 Hz, 3 H, $CH_2CH_3$), 1.65-1.92 (m, 8 H,

$\mathit{CH_2}CH_2CH_2CH_2C(O)O$ + $CH_2\mathit{CH_2}CH_2CH_2C(O)O$ + $CH_2CH_2\mathit{CH_2}CH_2C(O)O$ + $\mathit{CH_2}CH_3$), 2.45-2.55 ($\mathit{CH_2}CH{=}CH_2$), 2.65-2.75 (m, 2 H, $CH_2CH_2CH_2\mathit{CH_2}C(O)O$), 5.12-5.17 (m, 2 H, $CH_2CH{=}\mathit{CH_2}$), 5.78-5.88 (m, 1 H, $CH_2\mathit{CH}{=}CH_2$); $^{13}C$ NMR (101 MHz, $CDCl_3$) δ 7.7 ($CH_2\mathit{CH_3}$), 23.3 ($CH_2\mathit{CH_2}CH_2CH_2C(O)O$), 23.4 ($CH_2CH_2\mathit{CH_2}CH_2C(O)O$), 30.0 ($\mathit{CH_2}CH_3$), 36.2 ($\mathit{CH_2}CH_2CH_2CH_2C(O)O$), 37.4 ($CH_2CH_2CH_2\mathit{CH_2}C(O)O$), 42.3 ($\mathit{CH_2}CH{=}CH_2$), 85.1 ($\mathit{C}$), 118.8 ($CH_2CH{=}\mathit{CH_2}$), 132.7 ($CH_2\mathit{CH}{=}CH_2$), 174.8 ($\mathit{C}(O)O$) ppm; IR $\nu_{max}$ (thin film, $cm^{-1}$): 2923, 2851, 1703 (C=O), 1353, 1286, 1103 1008; HRMS calcd. for $C_{11}H_{18}O_2Na$ $[M+Na]^+$: 205.1199, found 205.1200.

Reaction conversion and enantiomeric purity of (*R*)-**2i** were determined by GC analysis in comparison with authentic racemic material (3% conversion, >99:1 e.r. shown, ChiraSil® DEX CB 25 m x 0.25 mm column, 50°C to 160°C at a rate of 1°C $min^{-1}$, flow rate 1.0 mL $min^{-1}$).

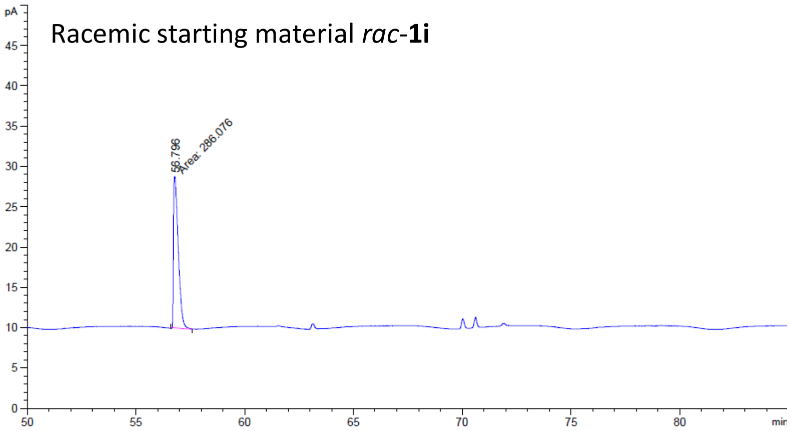


| Peak # | RetTime [min] | Type | Width [min] | Area [pA*s] | Height [pA] | Area % |
|---|---|---|---|---|---|---|
| 1 | 56.733 | MM | 0.2718 | 408.13089 | 25.02252 | 1.000e2 |

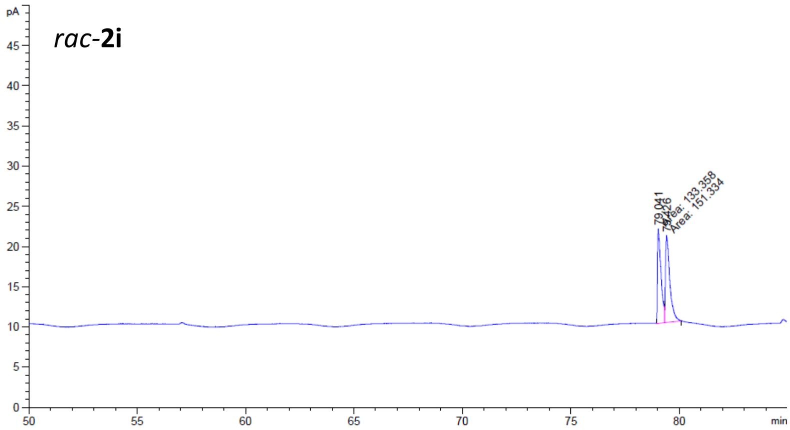


| Peak # | RetTime [min] | Type | Width [min] | Area [pA*s] | Height [pA] | Area % |
|---|---|---|---|---|---|---|
| 1 | 79.041 | MF | 0.1884 | 133.35817 | 11.79802 | 46.84298 |
| 2 | 79.426 | FM | 0.2327 | 151.33374 | 10.84035 | 53.15702 |

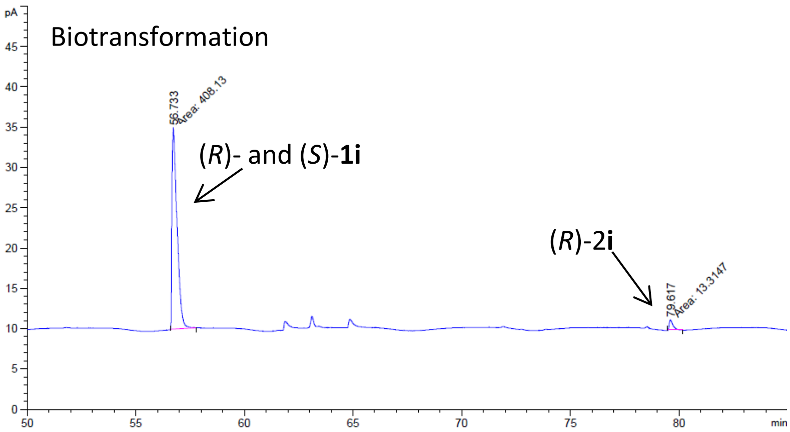


| Peak # | RetTime [min] | Type | Width [min] | Area [pA*s] | Height [pA] | Area % |
|---|---|---|---|---|---|---|
| 1 | 56.733 | MM | 0.2737 | 408.12997 | 24.85206 | 96.84069 |
| 2 | 79.617 | MM | 0.1819 | 13.31474 | 1.21980 | 3.15931 |

**(*R*)-6-Allyl-6-methyltetrahydro-2H-pyran-2-one (4a)**[11]

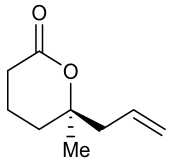


Prepared according to general procedure **D** using *rac*-**3a** (1 mg, 0.006 mmol), NADPH in EtOH (50 µL), NADPH (0.25 mg, 3.36 x $10^{-4}$ mmol), GDH (0.25 mg, 5.08 x $10^{-6}$ mmol), glucose (0.5 mg, 0.003 mmol), CHMO (0.25 mg, 4.10 x $10^{-6}$ mmol) and Tris/HCl buffer (450 µL, pH 7.0, 100 mM). The reaction mixture was extracted with EtOAc (500 µL) and analysed directly by GC. $^{1}$H NMR (400 MHz, $CDCl_3$) δ 1.38 (s, 3 H, $CH_3$), 1.65-1.70 (m, 1 H, $CH_aH_bCH_2CH_2C(O)O$), 1.78-1.92 (m, 3 H, $CH_aH_bCH_2CH_2C(O)O$ + $CH_2CH_2CH_2C(O)O$), 2.42-2.56 (m, 4 H, $CH_2CH_2CH_2C(O)O$) + $CH_2CH{=}CH_2$), 5.12-5.18 (m, 2 H, $CH_2CH{=}CH_2$), 5.80 (ddt, *J* = 17.1, 10.0, 7.3 Hz, 1 H, $CH_2CH{=}CH_2$) ppm; $^{13}$C NMR (101 MHz, $CDCl_3$) δ 16.6 ($CH_2CH_2CH_2C(O)O$), 26.4 ($CH_3$), 29.4 ($CH_2CH_2CH_2C(O)O$), 31.5 ($CH_2CH_2CH_2C(O)O$), 46.2 ($CH_2CH{=}CH_2$) 83.7 (*C*), 119.4 ($CH_2CH{=}CH_2$) 132.4 ($CH_2CH{=}CH_2$) 171.2 (*C*(O)O) ppm.

Reaction conversion and enantiomeric purity of (*R*)-**4a** were determined by GC analysis in comparison with authentic racemic material (60% conversion, 86:14 e.r. shown, ChiraSil® DEX CB 25 m x 0.25 mm column, 100°C to 150°C at a rate of 1°C $min^{-1}$, flow rate 1.0 mL $min^{-1}$).

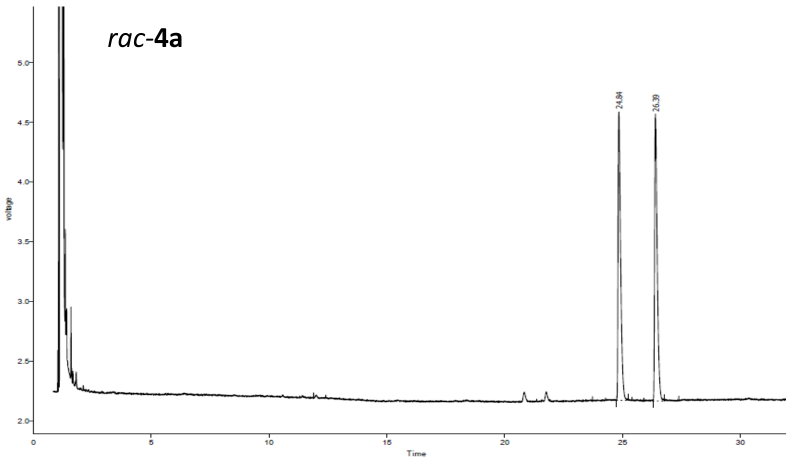


| | Reten. Time [min] | Area [mV.s] | Height [mV] | Area [%] | Height [%] | W05 [min] |
|---|---|---|---|---|---|---|
| 1 | 24.843 | 18.383 | 2.411 | 49.8 | 50.2 | 0.12 |
| 2 | 26.393 | 18.507 | 2.394 | 50.2 | 49.8 | 0.12 |
| | Total | 36.890 | 4.805 | 100.0 | 100.0 | |

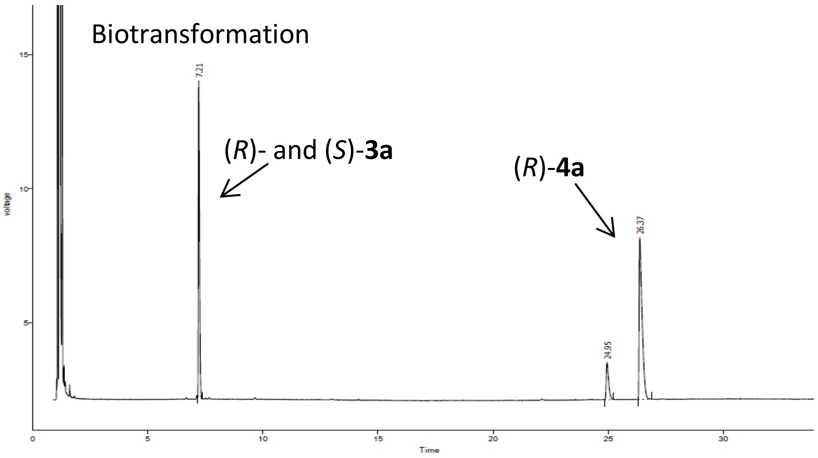


| | Reten. Time [min] | Area [mV.s] | Height [mV] | Area [%] | Height [%] | W05 [min] |
|---|---|---|---|---|---|---|
| 1 | 7.213 | 42.262 | 11.806 | 39.6 | 61.3 | 0.06 |
| 2 | 24.950 | 10.164 | 1.387 | 9.5 | 7.2 | 0.11 |
| 3 | 26.367 | 54.362 | 6.052 | 50.9 | 31.4 | 0.14 |
| | Total | 106.787 | 19.245 | 100.0 | 100.0 | |

**(*R,E*)-6-Cinnamyl-6-methyltetrahydro-2H-pyran-2-one (4b)**[11]

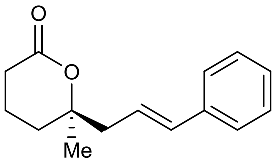


Prepared according to general procedure **D** using *rac*-**3b** (43 mg, 0.197 mmol) in EtOH (2.25 mL), NADPH (11.2 mg, 0.015 mmol), GDH (11.2 mg, 2.28 x $10^{-4}$ mmol), glucose (225 mg, 1.24 mmol), CHMO (11.2 mg, 1.86 x $10^{-4}$ mmol) and Tris/HCl buffer (12.75 mL, pH 7.0, 100 mM). Purification by

silica gel column chromatography (hexane/EtOAc 90:10) yielded the title product as a colourless oil (17 mg, 0.074 mmol, 37%). $^1$H NMR (400 MHz, $CDCl_3$) δ 1.42 (s, 3 H, C$H_3$), 1.68-1.73 (m, 1 H, C$H_a$H$_b$$CH_2CH_2$C(O)O), 1.84-1.93 (m, 3 H, $CH_a$$H_b$$CH_2CH_2$C(O)O + $CH_2$C$H_2$$CH_2$C(O)O), 2.47-2.54 (m, 2 H, $CH_2CH_2$C$H_2$C(O)O), 2.58 (dd, *J* = 7.4, 1.1 Hz, 2 H, C$H_2$CH=CHAr), 6.21 (dt, *J* = 15.8, 7.5 Hz, 1 H, $CH_2$C*H*=CHAr), 6.48 (d, *J* = 15.9 Hz, 1 H, $CH_2$CH=C*H*Ar), 7.22-7.26 (m, 1 H, ArC*H*), 7.30-7.34 (m, 2 H, 2 x ArC*H*), 7.36-7.38 (m, 2 H, 2 x ArC*H*) ppm; $^{13}$C NMR (101 MHz, $CDCl_3$) δ 16.6 ($CH_2$*C*$H_2CH_2$C(O)O), 26.5 (*C*$H_3$), 29.3 ($CH_2CH_2$*C*$H_2$C(O)O), 31.6 (*C*$H_2CH_2CH_2$C(O)O), 45.4 (*C*$H_2$CH=CHAr), 84.0 (*C*), 123.8 ($CH_2$*C*H=CHAr), 126.2 (2 x Ar*C*H), 127.5 (ArC*H*), 128.6 (2 x Ar*C*H), 134.3 ($CH_2$CH=*C*HAr), 137.0 (Ar*C*), 171.1 (*C*(O)O) ppm. Specific rotation $[\alpha]_D^{30}$ +18.1 (*c* 0.9, $CHCl_3$) for an enantiomerically enriched sample of 80% e.e.

Enantiomeric purity of (*R*)-**4b** was determined by HPLC analysis in comparison with authentic racemic material (90:10 e.r. shown; Lux 5 µm Amylose-1 column, 80:20 hexanes:$^i$PrOH, 1.0 mL min$^{-1}$, 20$^\circ$C, 254 nm).

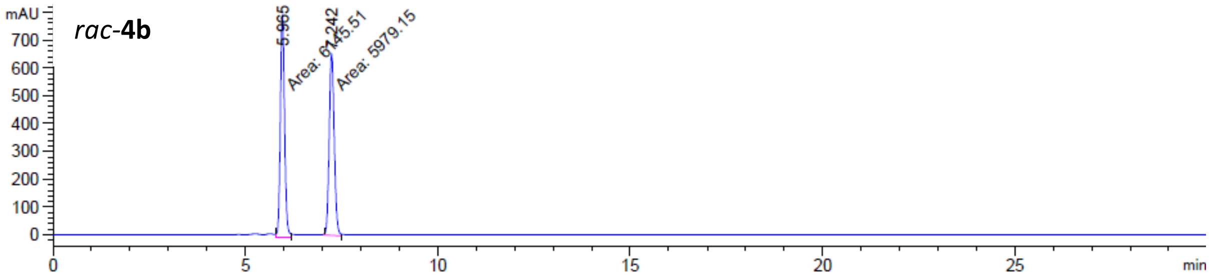


| Peak # | RetTime [min] | Type | Width [min] | Area [mAU*s] | Height [mAU] | Area % |
|---|---|---|---|---|---|---|
| 1 | 5.965 | MM | 0.1282 | 6145.50928 | 798.69446 | 50.6860 |
| 2 | 7.242 | MM | 0.1525 | 5979.15479 | 653.29315 | 49.3140 |

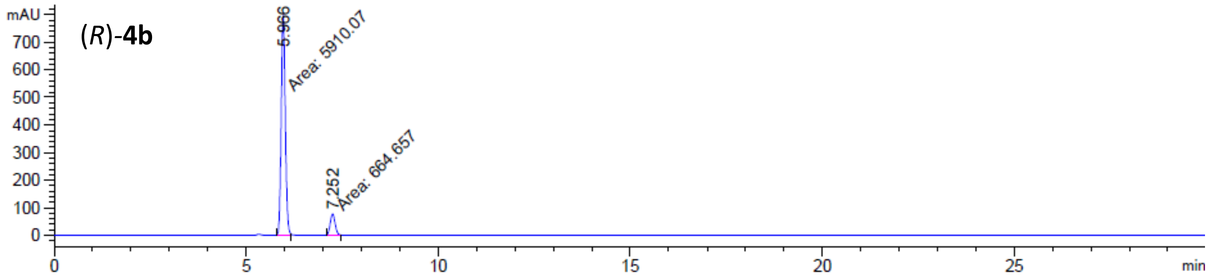


| Peak # | RetTime [min] | Type | Width [min] | Area [mAU*s] | Height [mAU] | Area % |
|---|---|---|---|---|---|---|
| 1 | 5.966 | MM | 0.1243 | 5910.07275 | 792.64209 | 89.8907 |
| 2 | 7.252 | MM | 0.1464 | 664.65723 | 75.65756 | 10.1093 |

Absolute stereochemistry was assigned by comparison with a sample of (*S,E*)-6-cinnamyl-6-methyltetrahydro-2H-pyran-2-one[8] (89:11 e.r. shown).

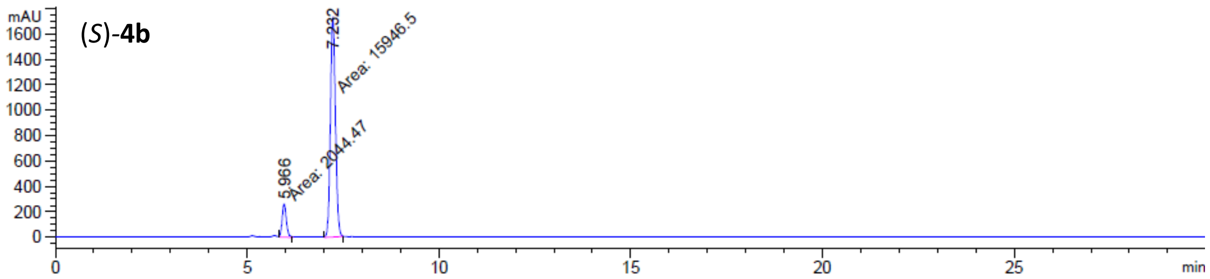


| Peak # | RetTime [min] | Type | Width [min] | Area [mAU*s] | Height [mAU] | Area % |
|---|---|---|---|---|---|---|
| 1 | 5.966 | MM | 0.1293 | 2044.46655 | 263.47360 | 11.3639 |
| 2 | 7.232 | MM | 0.1538 | 1.59465e4 | 1728.40491 | 88.6361 |

**(*R,E*)-6-(3-(4-Fluorophenyl)allyl)-6-methyltetrahydro-2H-pyran-2-one (4c)**

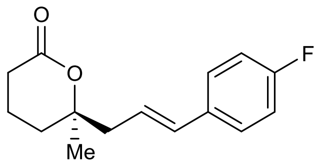


Prepared according to general procedure **D** using *rac*-**3c** (46 mg, 0.197 mmol), NADPH in EtOH (2.25 mL), (11.2 mg, 0.015 mmol), GDH (11.2 mg, 2.28 x $10^{-4}$ mmol), glucose (225 mg, 1.24 mmol), CHMO (11.2 mg, 1.86 x $10^{-4}$ mmol) and Tris/HCl buffer (12.75 mL, pH 7.0, 100 mM). Purification by silica gel column chromatography (hexane/EtOAc 90:10) yielded the title product as a colourless oil (6 mg, 0.024 mmol, 12%). $^1$H NMR (400 MHz, $CDCl_3$) δ 1.41 (s, 3 H, C*H*$_3$), 1.68-1.74 (m, 1 H, C*H*$_a$H$_b$CH$_2$CH$_2$C(O)O), 1.81 (m, 3 H, CH$_a$*H*$_b$CH$_2$CH$_2$C(O)O + CH$_2$C*H*$_2$CH$_2$C(O)O), 2.41-2.58 (m, 4 H, CH$_2$CH$_2$C*H*$_2$C(O)O) + C*H*$_2$CH=CHAr), 6.12 (dt, *J* = 15.6, 7.6 Hz, 1 H, CH$_2$C*H*=CHAr), 6.43 (d, *J* = 15.6 Hz, 1 H, CH$_2$CH=C*H*Ar), 6.99 (t, *J* = 8.7 Hz, 2 H, 2 x ArC*H*), 7.32 (dd, *J* = 8.5, 5.6 Hz, 2 H, 2 x ArC*H*) ppm; $^{13}$C NMR (101 MHz, $CDCl_3$) δ 16.5 (CH$_2$*C*H$_2$CH$_2$C(O)O), 26.4 (*C*H$_3$), 29.3 (CH$_2$CH$_2$*C*H$_2$C(O)O), 31.7 (*C*H$_2$CH$_2$CH$_2$C(O)O), 45.3 (*C*H$_2$CH=CHAr), 83.9 (*C*), 115.4 (d, *J* = 21.6 Hz, 2 x Ar*C*H), 123.5 (CH$_2$*C*H=CHAr), 127.6 (d, *J* = 7.9 Hz, 2 x Ar*C*H), 133.0 (CH$_2$CH=*C*HAr), 133.1 (Ar*C*), 160.9 (d, *J* = 246.6 Hz, Ar*C*F), 171.1 (*C*(O)O) ppm; $^{19}$F NMR (376 MHz, $CDCl_3$) -114.7 ppm; IR $\nu_{max}$ (thin film, cm$^{-1}$): 2940, 1722 (C=O), 1508, 1099, 1087, 1053;; HRMS calcd. for $C_{15}H_{17}O_2FNa$ [M+Na]$^+$: 271.1105, found 271.1099. Specific rotation $[\alpha]_D^{30}$ +22.6 (*c* 0.4, $CHCl_3$) for an enantiomerically enriched sample of 97% e.e.

Enantiomeric purity of (*R*)-**4c** was determined by HPLC analysis in comparison with authentic racemic material (98.5:1.5 e.r. shown; Lux 5 μm Amylose-1 column, 80:20 hexanes:$^i$PrOH, 1.0 mL min$^{-1}$, 20°C, 254 nm).

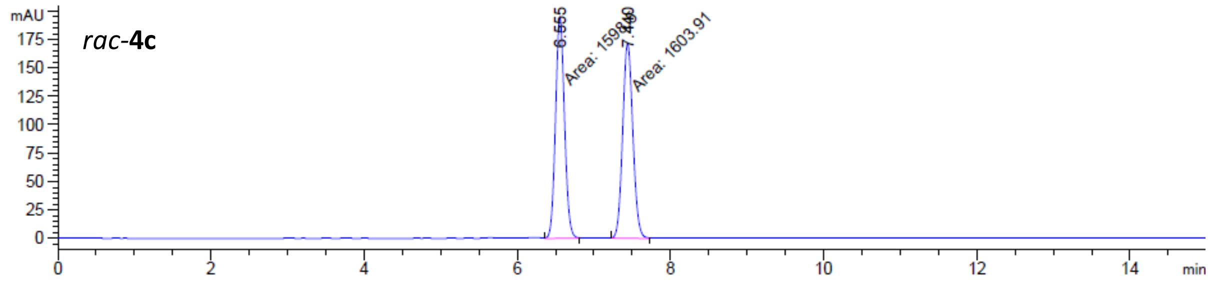


| Peak # | RetTime [min] | Type | Width [min] | Area [mAU*s] | Height [mAU] | Area % |
|---|---|---|---|---|---|---|
| 1 | 6.555 | MM | 0.1366 | 1598.49548 | 194.97868 | 49.9155 |
| 2 | 7.440 | MM | 0.1556 | 1603.90576 | 171.75266 | 50.0845 |

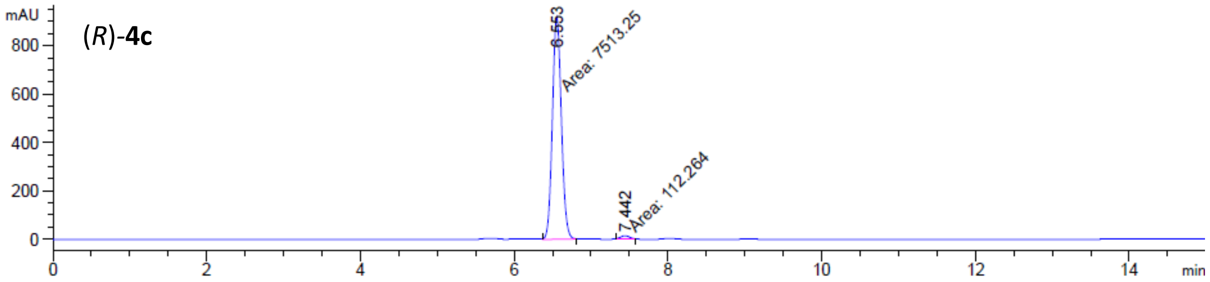


| Peak # | RetTime [min] | Type | Width [min] | Area [mAU*s] | Height [mAU] | Area % |
|---|---|---|---|---|---|---|
| 1 | 6.553 | MM | 0.1361 | 7513.24854 | 920.31403 | 98.5278 |
| 2 | 7.442 | MM | 0.1393 | 112.26443 | 13.43107 | 1.4722 |

Absolute stereochemistry was assigned by comparison with a sample of (*S,E*)-6-(3-(4-fluorophenyl)allyl)-6-methyltetrahydro-2H-pyran-2-one[8] (89:11 e.r. shown).

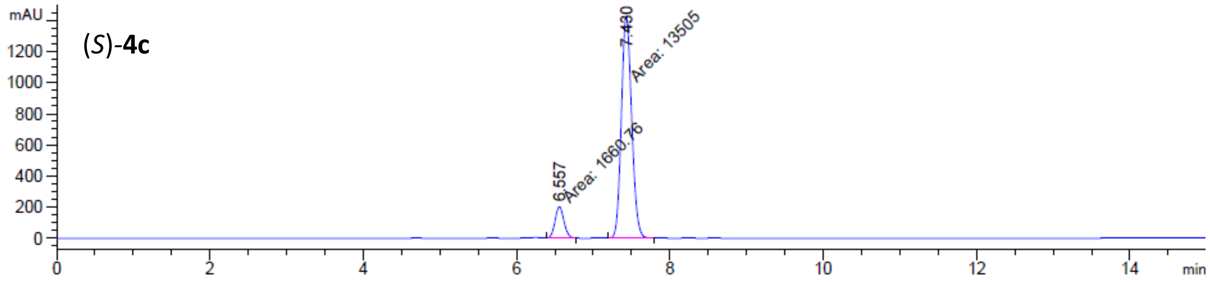


| Peak # | RetTime [min] | Type | Width [min] | Area [mAU*s] | Height [mAU] | Area % |
|---|---|---|---|---|---|---|
| 1 | 6.557 | MM | 0.1363 | 1660.75793 | 203.06351 | 10.9507 |
| 2 | 7.430 | MM | 0.1575 | 1.35050e4 | 1429.32166 | 89.0493 |

**(*E*)-6-Methyl-6-(3-(naphthalen-2-yl)allyl)tetrahydro-2H-pyran-2-one (4d)**

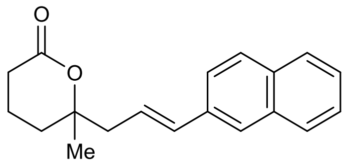

Ketone *rac*-**3d** (6 mg, 0.023 mmol) , was exposed to the conditions of general procedure **D** using NADPH (1.5 mg, 0.002 mmol), GDH (1.5 mg, 3.04 x $10^{-5}$ mmol), glucose (30 mg, 0.167 mmol), CHMO (1.5 mg, 2.46 x $10^{-5}$ mmol) and Tris/HCl buffer (2.7 mL, pH 7.0, 100 mM). The crude reaction mixture was analysed by $^1$H NMR, no conversion to product was observed.

Data for racemic standard: $^1$H NMR (400 MHz, $CDCl_3$) δ 1.45 (s, 3 H, C*H*$_3$), 1.72-1.76 (m, 1 H, C*H$_a$*H$_b$CH$_2$CH$_2$C(O)O), 1.86-1.96 (m, 3 H, CH$_a$*H$_b$*CH$_2$CH$_2$C(O)O + CH$_2$C*H$_2$*CH$_2$C(O)O), 2.46-2.65 (m, 4 H, CH$_2$CH$_2$C*H$_2$*C(O)O) + C*H$_2$*CH=CHAr), 6.34 (dt, *J* = 15.6, 7.6 Hz, 1 H, CH$_2$C*H*=CHAr), 6.64 (d, *J* = 15.8 Hz, 1 H, CH$_2$CH=C*H*Ar), 7.42-7.49 (m, 2 H, 2 x ArC*H*), 7.59 (d, *J* = 8.5 Hz, 1 H, ArC*H*), 7.72 (s, 1 H, ArC*H*), 7.78-7.82 (m, 3 H, 3 x ArC*H*) ; $^{13}$C NMR (101 MHz, $CDCl_3$) δ 16.7 (CH$_2$*C*H$_2$CH$_2$C(O)O), 26.6 (*C*H$_3$), 29.4 (CH$_2$CH$_2$*C*H$_2$C(O)O), 31.8 (*C*H$_2$CH$_2$CH$_2$C(O)O), 45.6 (*C*H$_2$CH=CHAr), 84.1 (*C*), 123.5 (Ar*C*H), 124.3 (CH$_2$*C*H=CHAr), 125.8 (Ar*C*H), 125.9 (Ar*C*H), 126.3 (Ar*C*H), 127.7 (Ar*C*H), 127.9 (Ar*C*H), 128.2 (Ar*C*H), 132.9 (Ar*C*), 133.6 (Ar*C*), 134.4 (CH$_2$CH=*C*HAr), 134.5 (Ar*C*), 171.2 (*C*(O)O) ppm; IR $\nu_{max}$ (thin film, $cm^{-1}$): 3055, 2974, 1720 (C=O), 1506, 1380, 1253, 1089; HRMS calcd. for $C_{19}H_{20}O_2Na$ $[M+Na]^+$: 303.1358, found 303.1356.

**(*E*)-6-(3-(3-Bromophenyl)allyl)-6-methyltetrahydro-2H-pyran-2-one (4e)**

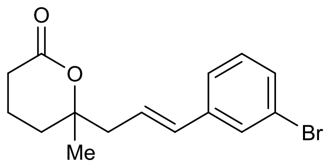


Ketone *rac*-**3e** (6 mg, 0.023 mmol) was exposed to the conditions of general procedure **D** using NADPH (1.5 mg, 0.002 mmol), GDH (1.5 mg, 3.04 x $10^{-5}$ mmol), glucose (30 mg, 0.167 mmol), CHMO (1.5 mg, 2.46 x $10^{-5}$ mmol) and Tris/HCl buffer (2.7 mL, pH 7.0, 100 mM). The crude reaction mixture was analysed by $^1$H NMR, 2% conversion to product was observed, e.e. n.d.

Data for racemic standard: $^1$H NMR (400 MHz, $CDCl_3$) δ 1.42 (s, 3 H, C*H$_3$*), 1.70-1.76 (m, 1 H, C*H$_a$*H$_b$CH$_2$CH$_2$C(O)O), 1.83-1.96 (m, 3 H, CH$_a$*H$_b$*CH$_2$CH$_2$C(O)O + CH$_2$C*H$_2$*CH$_2$C(O)O), 2.43-2.61 (m, 4 H, CH$_2$CH$_2$C*H$_2$*C(O)O) + C*H$_2$*CH=CHAr), 6.23 (dt, *J* = 15.8, 8.3 Hz, 1 H, CH$_2$C*H*=CHAr), 6.41 (d, *J* = 15.8 Hz, 1 H, CH$_2$CH=C*H*Ar), 7.18 (t, *J* = 7.8 Hz, 1 H, ArC*H*), 7.27-7.29 (m, 1 H, ArC*H*), 7.36 (d, *J* = 8.0 Hz, 1 H, ArC*H*), 7.52 (s, 1 H, ArC*H*) ppm; $^{13}$C NMR (101 MHz, $CDCl_3$) δ 16.6 (CH$_2$*C*H$_2$CH$_2$C(O)O), 26.5 (*C*H$_3$), 29.3 (CH$_2$CH$_2$*C*H$_2$C(O)O), 31.8 (*C*H$_2$CH$_2$CH$_2$C(O)O), 45.4 (*C*H$_2$CH=CHAr), 83.8 (*C*), 127.8 (Ar*C*Br), 124.9 (CH$_2$*C*H=CHAr), 125.5 (Ar*C*H), 129.1 (Ar*C*H), 130.1 (Ar*C*H), 130.3 (Ar*C*H) 132.9 (CH$_2$CH=*C*HAr), 139.1 (Ar*C*), 171.0 (*C*(O)O) ppm; IR $\nu_{max}$ (thin film, $cm^{-1}$): 2933, 1710 (C=O), 1469, 1440, 1333, 1290, 1101, 1017; HRMS calcd. for $C_{15}H_{17}O_2BrNa$ $[M+Na]^+$: 331.0304, found 331.0306.

**(*R,E*)-6-(3-(2-Chlorophenyl)allyl)-6-methyltetrahydro-2H-pyran-2-one (4f)[11]**

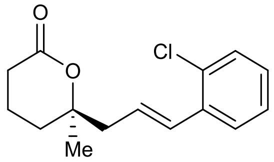

Prepared according to general procedure **D** using *rac*-**3f** (1 mg, 0.006 mmol), NADPH in EtOH (50 µL), NADPH (0.25 mg, 3.36 x $10^{-4}$ mmol), GDH (0.25 mg, 5.08 x $10^{-6}$ mmol), glucose (0.5 mg, 0.003 mmol), CHMO (0.25 mg, 4.10 x $10^{-6}$ mmol) and Tris/HCl buffer (450 µL, pH 7.0, 100 mM). The reaction mixture was extracted with EtOAc (500 µL) and analysed directly by GC. $^1$H NMR (400 MHz, $CDCl_3$) δ 1.43 (s, 3 H, C$H_3$), 1.70-1.76 (m, 1 H, C$H_a$$H_b$$CH_2CH_2$C(O)O), 1.83-1.96 (m, 3 H, $CH_a$$H_b$$CH_2CH_2$C(O)O + $CH_2$C$H_2$$CH_2$C(O)O), 2.43-2.63 (m, 4 H, $CH_2CH_2$C$H_2$C(O)O) + C$H_2$CH=CHAr), 6.19 (dt, *J* = 15.8, 7.8 Hz, 1 H, $CH_2$C*H*=CHAr), 6.85 (d, *J* = 15.8 Hz, 1 H, $CH_2$CH=C*H*Ar), 7.16-7.24 (m, 2 H, 2 x ArC*H*), 7.34 (d, *J* = 7.8 Hz, 1 H, ArC*H*), 7.51 (d, *J* = 7.5 Hz, 1 H, ArC*H*); $^{13}$C NMR (101 MHz, $CDCl_3$) δ 16.6 ($CH_2$*C*$H_2$$CH_2$C(O)O), 26.5 (*C*$H_3$), 29.3 ($CH_2CH_2$*C*$H_2$C(O)O), 31.8 (*C*$H_2$$CH_2CH_2$C(O)O), 45.5 (*C*$H_2$CH=CHAr), 83.8 (*C*), 126.8 (Ar*C*H), 126.8 (Ar*C*H), 126.9 ($CH_2$CH=CHAr), 128.5 (Ar*C*H), 129.6 (Ar*C*H), 130.5 ($CH_2$CH=*C*HAr), 132.7 (Ar*C*Cl), 135.1 (Ar*C*) 171.1 (*C*(O)O) ppm. IR $\nu_{max}$ (thin film, $cm^{-1}$): 2917, 1721 (C=O), 1469, 1290, 1166, 1089; HRMS calcd. for $C_{15}H_{17}O_2ClNa$ $[M+Na]^+$: 287.0809, found 287.0808.

Reaction conversion and enantiomeric purity of (*R*)-**4f** were determined by GC analysis in comparison with authentic racemic material (34% conversion, 86:14 e.r. shown, ChiraSil® DEX CB 25 m x 0.25 mm column, 50°C to 200°C at a rate of 1°C $min^{-1}$, flow rate 1.0 mL $min^{-1}$).

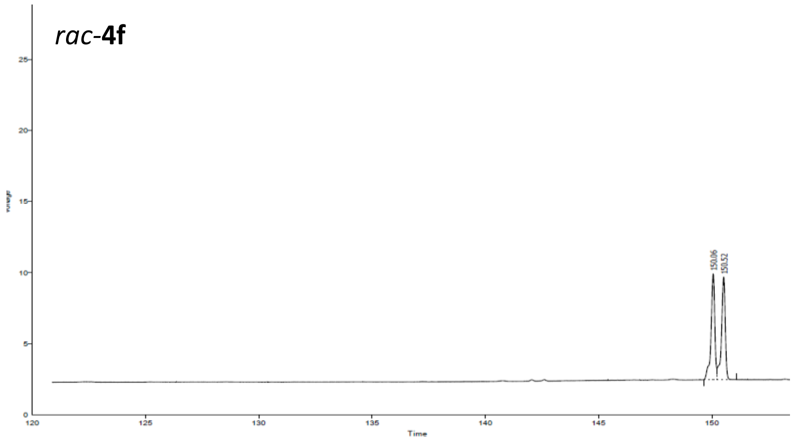


| | Reten. Time [min] | Area [mV.s] | Height [mV] | Area [%] | Height [%] | W05 [min] |
|---|---|---|---|---|---|---|
| 1 | 150.057 | 85.500 | 7.448 | 50.3 | 50.8 | 0.16 |
| 2 | 150.523 | 84.590 | 7.217 | 49.7 | 49.2 | 0.17 |
| | Total | 170.090 | 14.665 | 100.0 | 100.0 | |

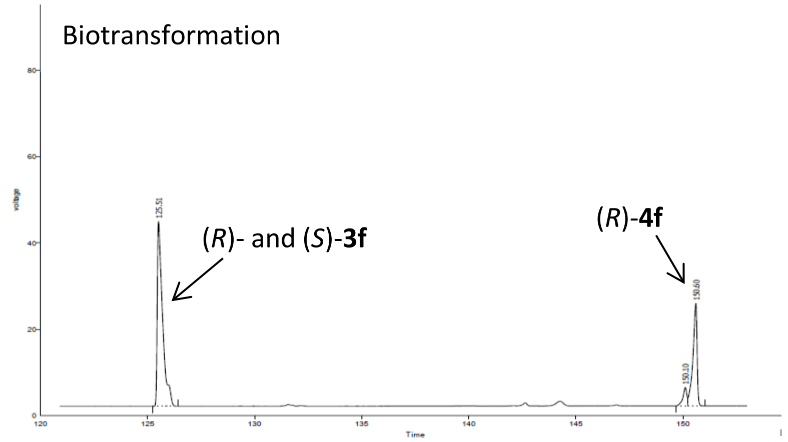


| | Reten. Time [min] | Area [mV.s] | Height [mV] | Area [%] | Height [%] | W05 [min] |
|---|---|---|---|---|---|---|
| 1 | 125.513 | 742.446 | 42.832 | 66.2 | 60.5 | 0.25 |
| 2 | 150.103 | 54.672 | 4.258 | 4.9 | 6.0 | 0.20 |
| 3 | 150.603 | 323.962 | 23.713 | 28.9 | 33.5 | 0.20 |
| | Total | 1121.080 | 70.803 | 100.0 | 100.0 | |

**(*E*)-6-Methyl-6-(3-(4-(trifluoromethyl)phenyl)allyl)tetrahydro-2H-pyran-2-one (4g)**

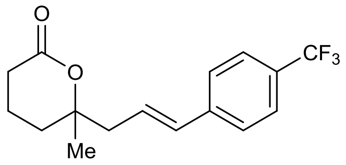


Ketone *rac*-**3g** (6 mg, 0.023 mmol), was exposed to the conditions of general procedure **D** using NADPH (1.5 mg, 0.002 mmol), GDH (1.5 mg, 3.04 x $10^{-5}$ mmol), glucose (30 mg, 0.167 mmol), CHMO (1.5 mg, 2.46 x $10^{-5}$ mmol) and Tris/HCl buffer (2.7 mL, pH 7.0, 100 mM). The crude reaction mixture was analysed by $^{1}$H NMR, 4% conversion to product was observed, e.e. n.d.

Data for racemic standard: $^{1}$H NMR (400 MHz, $CDCl_3$) δ 1.43 (s, 3 H, $CH_3$), 1.71-1.78 (m, 1 H, $CH_aH_bCH_2CH_2C(O)O$), 1.83-1.96 (m, 3 H, $CH_aH_bCH_2CH_2C(O)O$ + $CH_2CH_2CH_2C(O)O$), 2.43-2.65 (m, 4 H, $CH_2CH_2CH_2C(O)O$) + $CH_2CH$=CHAr), 6.33 (dt, *J* = 15.8, 7.5 Hz, 1 H, $CH_2CH$=CHAr), 6.51 (d, *J* = 15.8 Hz, 1 H, $CH_2CH$=C*H*Ar), 7.46 (d, *J* = 8.0 Hz, 2 H, 2 x ArC*H*), 7.56 (d, *J* = 8.3 Hz, 2 H, 2 x ArC*H*); $^{13}$C NMR (101 MHz, $CDCl_3$) 16.6 ($CH_2CH_2CH_2C(O)O$), 26.5 ($CH_3$), 29.3 ($CH_2CH_2CH_2C(O)O$), 31.9 ($CH_2CH_2CH_2C(O)O$), 45.5 ($CH_2CH$=CHAr), 83.7 (*C*), 125.5 (q, *J* = 271.9 Hz, Ar$CCF_3$), 125.6 (q, *J* = 3.9 Hz, 2 x Ar*C*H), 126.4 (2 x Ar*C*H), 126.8 ($CH_2CH$=CHAr), 129.4 (q, *J* = 32.5 Hz, $CF_3$), 133.0 ($CH_2CH$=*C*HAr), 140.4 (Ar*C*), 171.0 (*C*(O)O) ppm; $^{19}$F NMR (376 MHz, $CDCl_3$) -62.5 ppm; IR $\nu_{max}$ (thin film, $cm^{-1}$): 2925, 1721 (C=O), 1614, 1414, 1321, 1161, 1065; HRMS calcd. for $C_{16}H_1O_2F_3$ $[M-H]^-$: 297.1108, found 297.1108.

**(*R,E*)-6-Methyl-6-(3-(o-tolyl)allyl)tetrahydro-2H-pyran-2-one (4h)**

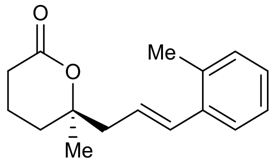

Prepared according to general procedure **D** using *rac*-**3h** (1 mg, 0.006 mmol), NADPH in EtOH (50 µL), NADPH (0.25 mg, 3.36 x $10^{-4}$ mmol), GDH (0.25 mg, 5.08 x $10^{-6}$ mmol), glucose (0.5 mg, 0.003 mmol), CHMO (0.25 mg, 4.10 x $10^{-6}$ mmol) and Tris/HCl buffer (450 µL, pH 7.0, 100 mM). The reaction mixture was extracted with EtOAc (500 µL) and analysed directly by GC. $^{1}$H NMR (400 MHz, $CDCl_3$) δ 1.44 (s, 3 H, C$H_3$), 1.57-1.76 (m, 1 H, C$H_a$H$_b$CH$_2$CH$_2$C(O)O), 1.85-1.94 (m, 3 H, CH$_a$$H_b$CH$_2$CH$_2$C(O)O + CH$_2$C$H_2$CH$_2$C(O)O), 2.35 (s, 3 H, ArCC$H_3$), 2.43-2.62 (m, 4 H CH$_2$CH$_2$C$H_2$C(O)O) + C$H_2$CH=CHAr), 6.07 (dt, *J* = 15.6, 7.5 Hz, 1 H, CH$_2$C*H*=CHAr), 6.69 (d, *J* = 15.8 Hz, 1 H, CH$_2$CH=C*H*Ar), 7.14-7.18 (m, 3 H, 3 x ArC*H*), 7.42 (dd, *J* = 4.6, 3.9 Hz, 1 H, ArC*H*); $^{13}$C NMR (101 MHz, $CDCl_3$) 16.6 (CH$_2$*C*H$_2$CH$_2$C(O)O), 19.8 (ArC*C*H$_3$), 26.6 (*C*H$_3$), 29.4 (CH$_2$CH$_2$*C*H$_2$C(O)O), 31.7 (*C*H$_2$CH$_2$CH$_2$C(O)O), 45.7 (*C*H$_2$CH=CHAr), 84.0 (*C*), 125.2 (CH$_2$*C*H=CHAr), 125.7 (Ar*C*H), 126.1 (ArCH), 127.4 (Ar*C*H), 130.3 (Ar*C*H), 132.3 (CH$_2$CH=*C*HAr), 135.1 (Ar*C*), 136.2 (Ar*C*), 171.2 (*C*(O)O) ppm; IR $\nu_{max}$ (thin film, cm$^{-1}$): 2917, 1735 (C=O), 1685, 1293, 1256, 1088, 1053; HRMS calcd. for $C_{16}H_{20}O_2Na$ [M+Na]$^+$: 267.1356, found 267.1349.

Reaction conversion and enantiomeric purity of (*R*)-**4h** were determined by GC analysis in comparison with authentic racemic material (39% conversion, 94:6 e.r. shown, ChiraSil® DEX CB 25 m x 0.25 mm column, 50°C to 200°C at a rate of 1°C min$^{-1}$, flow rate 1.0 mL min$^{-1}$).

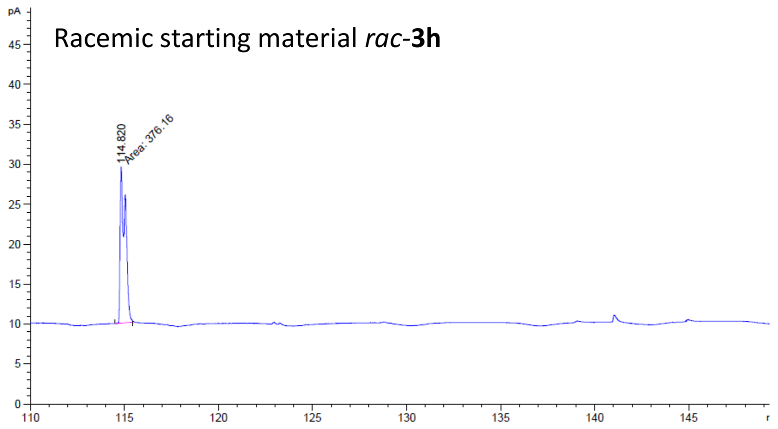


```
Peak RetTime Type  Width     Area      Height     Area
  #   [min]        [min]   [pA*s]      [pA]         %
----|-------|----|-------|----------|----------|--------|
   1 114.820 MM    0.3203  376.15970   19.57586 1.000e2
```

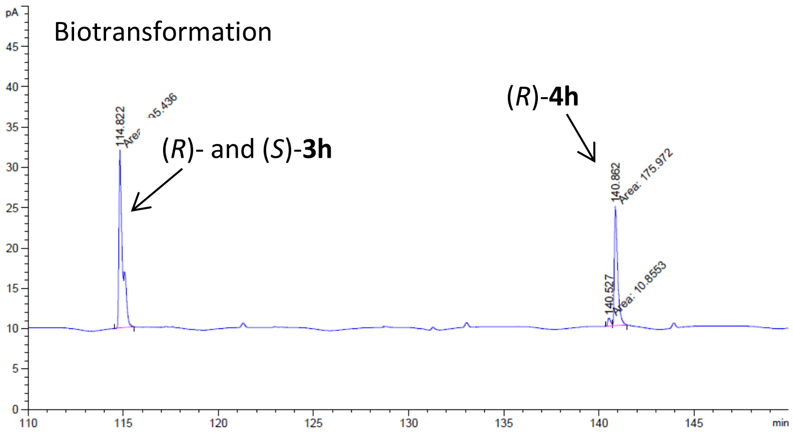


| Peak # | RetTime [min] | Type | Width [min] | Area [pA*s] | Height [pA] | Area % |
|---|---|---|---|---|---|---|
| 1 | 114.822 | MM | 0.2233 | 295.43588 | 22.05362 | 61.26034 |
| 2 | 140.527 | MF | 0.1824 | 10.85530 | 9.92070e-1 | 2.25091 |
| 3 | 140.862 | FM | 0.1977 | 175.97168 | 14.83417 | 36.48875 |

### 5.3. Additional Unreactive Substrates

Seven-membered ketones **S6** and **S7** were prepared from literature procedures[12] and were exposed to the biotransformation conditions described in general procedure **D**. The crude reaction mixture was analysed by $^1$H NMR, which indicated that no starting material had been converted to the desired products.

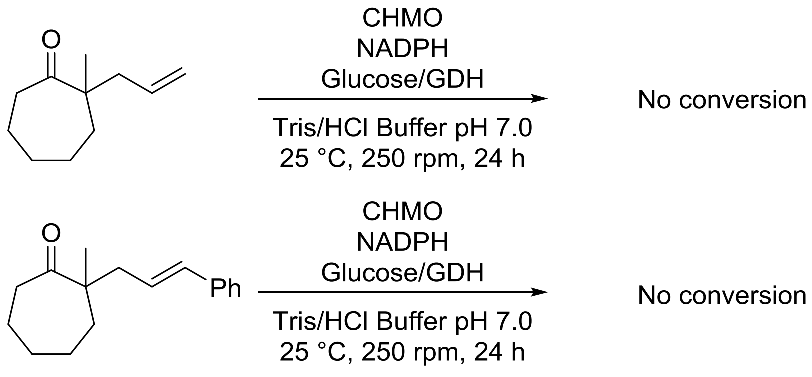


Scheme S2: Unsuccessful biostransformations of seven-membered ketones.

**5.4. $SmI_2$-Mediated Cyclizations**

**General Procedure E: $SmI_2$-$H_2O$-mediated radical lactone cyclisation followed by DMP oxidation**

**(1*S*,6*R*,8*S*)-8-Benzyl-6-methyl-9-oxabicyclo[4.2.1]nonan-1-ol (6a)[9]**

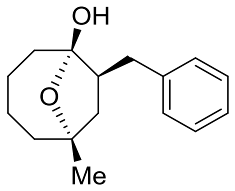


To a vial charged with 0.1 M $SmI_2$ solution in THF (4.58 mL, 0.458 mmol) was added degassed distilled $H_2O$ (0.82 mL, 45.8 mmol) to generate a deep purple solution. Following stirring for approximately 5 minutes, a solution of lactone (*R*)-**2b** (14 mg, 0.057 mmol) in THF was added slowly. The reaction was stirred at room temperature until decolourisation occurred. A saturated aqueous solution of Rochelle's salt (3 mL) was added and the mixture was extracted with $Et_2O$ (3 x 2 mL). The organic layers were combined, washed with saturated aqueous NaCl (3 mL), dried ($MgSO_4$) and concentrated. The resulting crude cyclooctandiol was dissolved in $CH_2Cl_2$ (0.8 mL) and Dess-Martin Periodinane (36 mg, 0.086 mmol) was added in one portion at 0°C. The reaction was allowed to warm to room temperature and stirred for 7 hours. The mixture was quenched using a 1:1 mixture of saturated aqueous solutions of $Na_2S_2O_3$ and $NaHCO_3$ (3 mL), then extracted with $CH_2Cl_2$ (3 x 2 mL). The organic layers were combined, dried ($MgSO_4$) and concentrated. The crude mixture was purified by silica gel column chromatography (hexane/EtOAc 95:5) to yield the title product as a 72:28 mixture of diastereomers (10.3 mg, 0.041 mmol, 73%). The major diastereomer could be isolated and was obtained as a colourless oil. $^1$H NMR (400 MHz, $CDCl_3$) δ 1.28 (s, 3 H, C*H*$_3$), 1.56-1.92 (m, 9 H, C*H*$_2$CH$_2$CH$_2$CH$_2$COH + CH$_2$C*H*$_2$CH$_2$CH$_2$COH + CH$_2$CH$_2$C*H*$_2$CH$_2$COH + CH$_2$CH$_2$CH$_2$C*H*$_a$H$_b$COH + C*H*$_2$CHCH$_2$Ar), 2.17-2.33 (m, 2 H, CH$_2$CH$_2$CH$_2$CH$_a$*H*$_b$COH + C*H*CH$_2$Ar), 2.57 (t, *J* = 12.1 Hz, 1 H, C*H*$_a$H$_b$Ar), 3.01 (dd, *J* = 12.3, 1.3 Hz, 1 H, CH$_a$*H*$_b$Ar), 7.15-7.23 (m, 3 H, 3 x ArC*H*), 7.28-7.33 (m, 2 H, 2 x ArC*H*); $^{13}$C NMR (101 MHz, $CDCl_3$) 22.4 (CH$_2$CH$_2$*C*H$_2$CH$_2$COH), 25.0 (CH$_2$*C*H$_2$CH$_2$CH$_2$COH), 31.1 (*C*H$_3$), 36.0 (*C*H$_2$Ar), 37.8 (CH$_2$CH$_2$CH$_2$*C*H$_2$COH), 41.8 (*C*H$_2$CH$_2$CH$_2$CH$_2$COH), 42.9 (*C*H$_2$CHCH$_2$Ar), 51.8 (*C*HCH$_2$Ar), 78.9 (CH$_3$*C*O), 107.9 (O*C*OH), 126.0 (Ar*C*H), 128.4 (2 x Ar*C*H), 128.6 (2 x Ar*C*H), 140.7 (Ar*C*) ppm; Specific rotation $[\alpha]_D^{26}$ +12.6 (*c* 0.47, $CHCl_3$) for an enantiomerically enriched sample of >99% e.e

Enantiomeric purity of (1*S*,6*R*,8*S*)**-6a** was determined by HPLC analysis in comparison with authentic racemic material (>99:1 e.r. shown; Lux 5 μm Amylose-1 column, 95:5 hexanes:$^i$PrOH, 0.5 mL min$^{-1}$, 20°C, 210 nm).

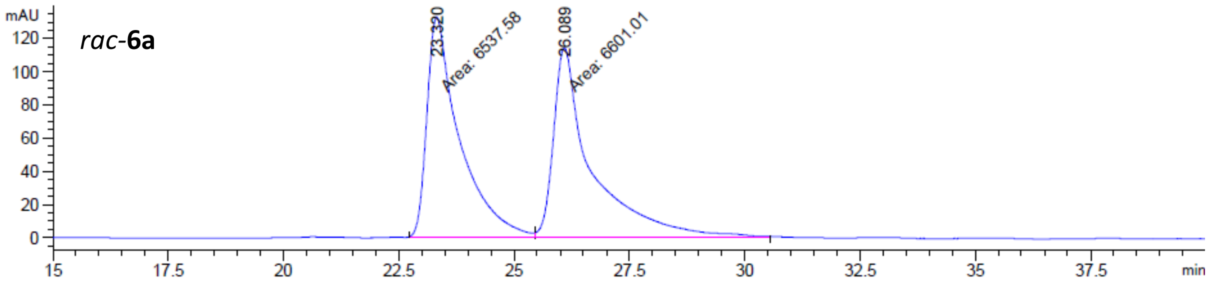


| Peak # | RetTime [min] | Type | Width [min] | Area [mAU*s] | Height [mAU] | Area % |
|---|---|---|---|---|---|---|
| 1 | 23.320 | MF | 0.8216 | 6537.58105 | 132.62585 | 49.7586 |
| 2 | 26.089 | FM | 0.9639 | 6601.00732 | 114.13433 | 50.2414 |

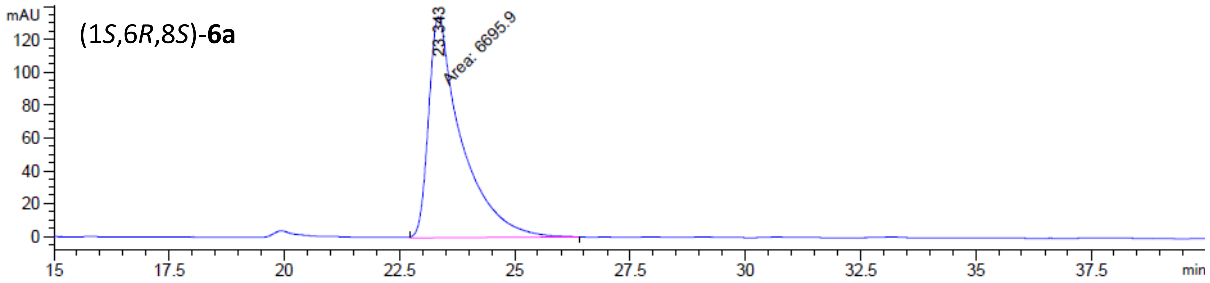


| Peak # | RetTime [min] | Type | Width [min] | Area [mAU*s] | Height [mAU] | Area % |
|---|---|---|---|---|---|---|
| 1 | 23.343 | MM | 0.8293 | 6695.90186 | 134.57529 | 100.0000 |

**(1S,6R,8S)-8-(4-fluorobenzyl)-6-methyl-9-oxabicyclo[4.2.1]nonan-1-ol (6b)**

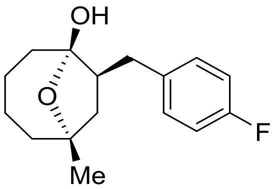


Prepared according to general procedure **E** using lactone (*R*)-**2c** (9 mg, 0.034 mmol), $SmI_2$, 0.1 M in THF (2.7 mL, 0.27 mmol) and $H_2O$ (0.49 mL, 27 mmol), followed by Dess-Martin Periodinane (22 mg, 0.051 mmol) in $CH_2Cl_2$ (0.48 mL). Purification by silica gel column chromatography (hexane/EtOAc 95:5) yielded the title product as an 75:25 mixture of diastereomers (7.8 mg, 0.030 mmol, 87%). Major diastereomer: $^1$H NMR (400 MHz, $CDCl_3$) δ 1.28 (s, 3 H, $CH_3$), 1.63-1.86 (m, 9 H, $\mathit{CH_2}CH_2CH_2CH_2COH$ + $CH_2\mathit{CH_2}CH_2CH_2COH$ + $CH_2CH_2\mathit{CH_2}CH_2COH$ + $CH_2CH_2CH_2\mathit{CH_a}H_bCOH$ + $\mathit{CH_2}CHCH_2Ar$), 2.04-2.28 (m, 2 H, $CH_2CH_2CH_2CH_a\mathit{H_b}COH$ + $C\mathit{H}CH_2Ar$), 2.53 (apparent t, *J* = 12.2 Hz, 1 H, $\mathit{CH_a}H_bAr$), 2.97 (d, *J* = 12.7 Hz, 1 H, $CH_a\mathit{H_b}Ar$), 6.96-7.00 (m, 2 H, 2 x ArC*H*), 7.14-7.17 (m, 2 H, 2 x ArC*H*); $^{13}$C NMR (101 MHz, $CDCl_3$) 22.5 ($CH_2CH_2\mathit{C}H_2CH_2COH$), 25.0 ($CH_2\mathit{C}H_2CH_2CH_2COH$), 31.2 ($\mathit{C}H_3$), 35.2 ($\mathit{C}H_2Ar$), 37.9 ($CH_2CH_2CH_2\mathit{C}H_2COH$), 41.9 ($\mathit{C}H_2CH_2CH_2CH_2COH$), 42.9 ($\mathit{C}H_2CHCH_2Ar$), 52.0 ($\mathit{C}HCH_2Ar$), 78.6 ($CH_3\mathit{C}O$), 107.6 ($O\mathit{C}OH$), 115.2 (d, *J* = 21.1 Hz, 2 x Ar*C*H), 129.9 (d, *J* = 7.8 Hz, 2 x

ArCH), 133.4 (ArC), ArCF not observed; $^{19}$F NMR (376 MHz, $CDCl_3$) -117.4 ppm; IR $\nu_{max}$ (thin film, $cm^{-1}$): 3384 (O-H), 2963, 2829, 1643, 1498, 1365, 1241, 1145, 1090; HRMS calcd. for $C_{16}H_{21}O_2FNa$ $[M+Na]^+$: 278.1418, found 287.1404; Specific rotation $[\alpha]_D^{26}$ +26.3 (*c* 0.12, $CHCl_3$) for an enantiomerically enriched sample of >99% e.e.

Enantiomeric purity of (1S,6R,8S)-**6b** was determined by HPLC analysis in comparison with authentic racemic material (>99:1 e.r. shown; Lux 5 µm Amylose-1 column, 98:2 hexanes:$^i$PrOH, 0.5 mL $min^{-1}$, 20°C, 210 nm).

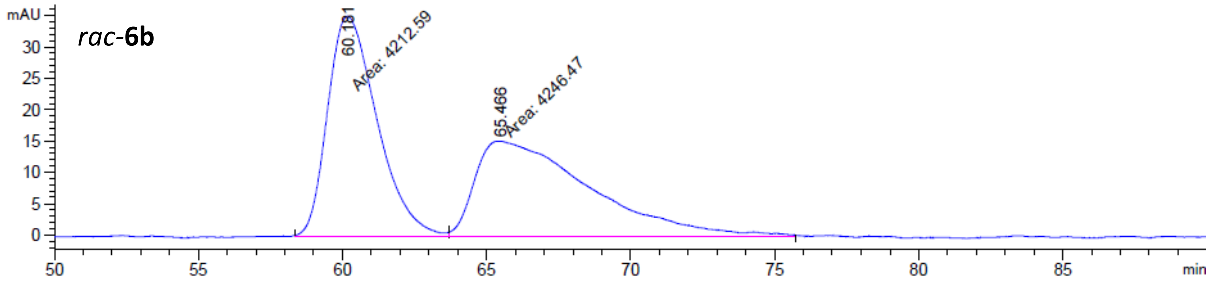


| Peak # | RetTime [min] | Type | Width [min] | Area [mAU*s] | Height [mAU] | Area % |
|---|---|---|---|---|---|---|
| 1 | 60.181 | MF | 1.9961 | 4212.58740 | 35.17375 | 49.7997 |
| 2 | 65.466 | FM | 4.6382 | 4246.47363 | 15.25906 | 50.2003 |

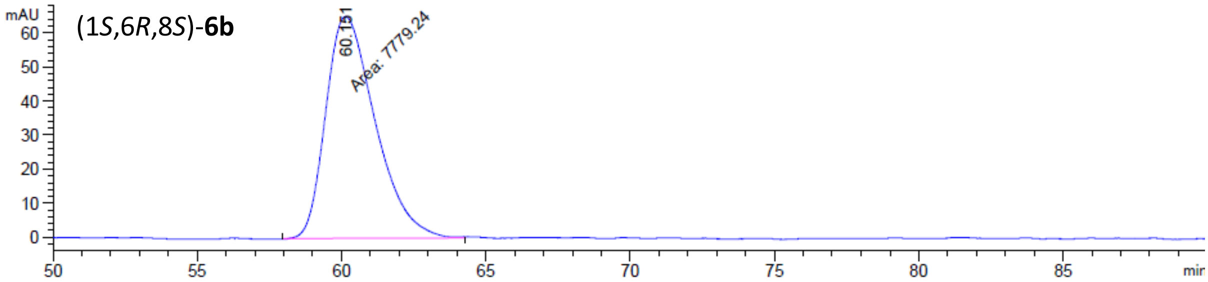


| Peak # | RetTime [min] | Type | Width [min] | Area [mAU*s] | Height [mAU] | Area % |
|---|---|---|---|---|---|---|
| 1 | 60.151 | MM | 1.9901 | 7779.24219 | 65.14886 | 100.0000 |

**(1*S*,6*R*,8*S*)-8-(2-Chlorobenzyl)-6-methyl-9-oxabicyclo[4.2.1]nonan-1-ol (6c)[9]**

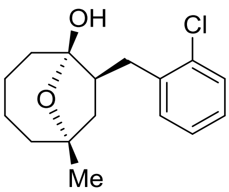


Prepared according to general procedure **E** using lactone (*R*)-**2f** (15 mg, 0.054 mmol), $SmI_2$, 0.1 M in THF (4.3 mL, 0.43 mmol) and $H_2O$ (0.77 mL, 43 mmol), followed by Dess-Martin Periodinane (34 mg, 0.081 mmol) in $CH_2Cl_2$ (0.75 mL). Purification by silica gel column chromatography (hexane/EtOAc 95:5) yielded the title product as an 80:20 mixture of diastereomers (10.6 mg, 0.038 mmol, 70%).

The major diastereomer could be isolated and was obtained as a pale yellow oil. $^{1}$H NMR (400 MHz, $CDCl_3$) δ 1.27 (s, 3 H, $CH_3$), 1.55-1.93 (m, 9 H, $CH_2CH_2CH_2CH_2COH$ + $CH_2CH_2CH_2CH_2COH$ + $CH_2CH_2CH_2CH_2COH$ + $CH_2CH_2CH_2CH_aH_bCOH$ + $CH_2CHCH_2Ar$), 2.17-2.21 (m, 1 H, $CH_2CH_2CH_2CH_aH_bCOH$), 2.32-2.38 (m, 1 H, $CHCH_2Ar$), 2.75 (dd, $J$ = 13.2, 11.2 Hz, 1 H, $CH_aH_bAr$), 3.12 (dd, $J$ = 13.2, 3.9 Hz, 1 H, $CH_aH_bAr$), 7.15-7.26 (m, 3 H, 3 x ArC$H$), 7.36 (dd, $J$ = 7.5, 1.5 Hz, 1 H, ArC$H$); $^{13}$C NMR (101 MHz, $CDCl_3$) 22.4 ($CH_2CH_2CH_2CH_2COH$), 25.1 ($CH_2CH_2CH_2CH_2COH$), 31.1 ($CH_3$), 33.2 ($CH_2Ar$), 37.6 ($CH_2CH_2CH_2CH_2COH$), 41.8 ($CH_2CH_2CH_2CH_2COH$), 42.2 ($CH_2CHCH_2Ar$), 50.4 ($CHCH_2Ar$), 78.6 ($CH_3CO$), 107.7 (O$C$OH), 126.8 (Ar$C$H), 127.6 (Ar$C$H), 129.7 (Ar$C$H), 130.6 (Ar$C$H), 133.9 (Ar$C$Cl), 138.3 (Ar$C$) ppm; Specific rotation $[\alpha]_D^{26}$ -4.9 ($c$ 0.8, $CHCl_3$) for an enantiomerically enriched sample of >99% e.e.

Enantiomeric purity of (1*S*,6*R*,8*S*)-**6c** was determined by HPLC analysis in comparison with authentic racemic material (>99:1 e.r. shown; Lux 5 µm Amylose-1 column, 85:15 hexanes:$^{i}$PrOH, 0.3 mL min$^{-1}$, 20°C, 210 nm).

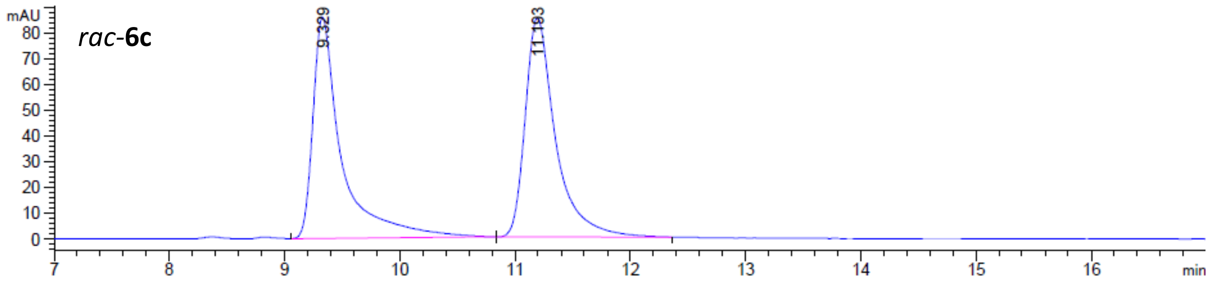


| Peak # | RetTime [min] | Type | Width [min] | Area [mAU*s] | Height [mAU] | Area % |
|---|---|---|---|---|---|---|
| 1 | 9.329 | BB | 0.2422 | 23.56791 | 1.37319 | 48.9781 |
| 2 | 11.192 | BB | 0.2612 | 24.55139 | 1.36919 | 51.0219 |

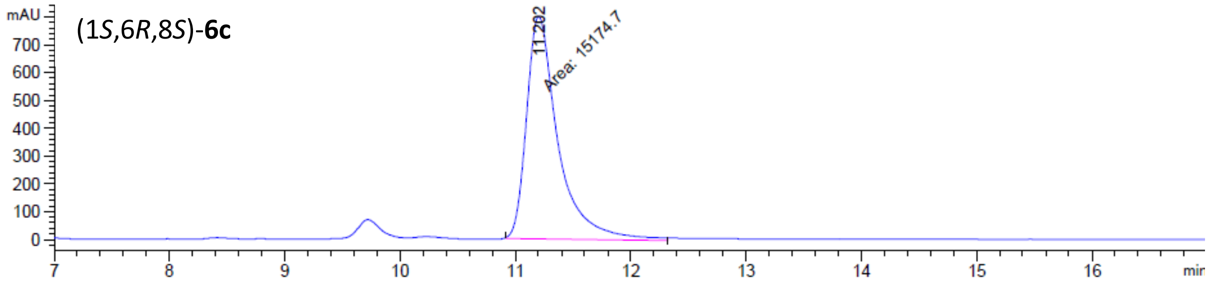


| Peak # | RetTime [min] | Type | Width [min] | Area [mAU*s] | Height [mAU] | Area % |
|---|---|---|---|---|---|---|
| 1 | 11.202 | MM | 0.3278 | 260.23608 | 13.23344 | 100.0000 |

**(1*S*,5*R*,7*S*)-7-Benzyl-5-methyl-8-oxabicyclo[3.2.1]octan-1-ol (6d)[11]**

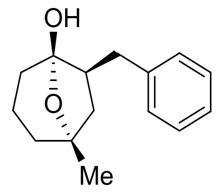


Prepared according to general procedure **E** using lactone (*R*)-**4b** (15 mg, 0.065 mmol), $SmI_2$, 0.1 M in THF (5.2 mL, 0.52 mmol) and $H_2O$ (0.93 mL, 52 mmol), followed by Dess-Martin Periodinane (41 mg, 0.098 mmol) in $CH_2Cl_2$ (0.91 mL). Purification by silica gel column chromatography ($CHCl_3$/$Et_2O$ 98:2 to hexane/EtOAc 95:5) yielded the title product as a 75:25 mixture of diastereomers (8.7 mg, 0.037 mmol, 58%). Major diastereomer: $^1$H NMR (400 MHz, $CDCl_3$) δ 1.31 (s, 3 H, $CH_3$), 1.38 (dd, *J* = 13.2, 5.1 Hz, 1 H, $CH_aH_bCHCH_2Ar$), 1.51 (dd, *J* = 12.9, 7.5 Hz, 1 H, $CH_aH_bCHCH_2Ar$), 1.62-1.68 (m, 2 H, $CH_2CH_2CH_aH_bCOH$ + $CH_2CH_aH_bCH_2COH$), 1.79-2.00 (m, 4 H, $CH_2CH_2CH_2COH$ + $CH_2CH_2CH_aH_bCOH$ + $CH_2CH_aH_bCH_2COH$), 2.32-2.36 (m, 1 H, $CHCH_2Ar$), 2.67 (dd, *J* = 13.4, 11.7 Hz, 1 H, $CH_aH_bAr$), 2.95 (dd, *J* = 13.6, 4.3 Hz, 1 H, $CH_aH_bAr$), 7.19-7.23 (m, 3 H, 3 x ArC*H*), 7.28-7.32 (m, 2 H, 2 x ArC*H*); $^{13}$C NMR (101 MHz, $CDCl_3$) 19.0 ($CH_2CH_2CH_2COH$), 27.2 ($CH_3$), 31.5 ($CH_2CH_2CH_2COH$), 34.8 ($CH_2Ar$), 35.6 ($CH_2CHCH_2Ar$), 39.5 ($CH_2CH_2CH_2COH$), 50.9 ($CHCH_2Ar$), 79.3 ($CH_3CO$), 104.5 (OCOH), 126.0 (ArCH), 128.5 (2 x ArCH), 128.5 (2 x ArCH), 140.9 (ArC) ppm; Specific rotation $[\alpha]_D^{26}$ +42.4 (*c* 0.24, $CHCl_3$) for an enantiomerically enriched sample of >99% e.e

Enantiomeric purity of (1*S*,6*R*,8*S*)-**6d** was determined by GC analysis in comparison with authentic racemic material (90:10 e.r. shown, Beta DEX™ 120 30 m x 0.25 mm column, 50°C to 200°C at a rate of 1°C $min^{-1}$, flow rate 1.0 mL $min^{-1}$).

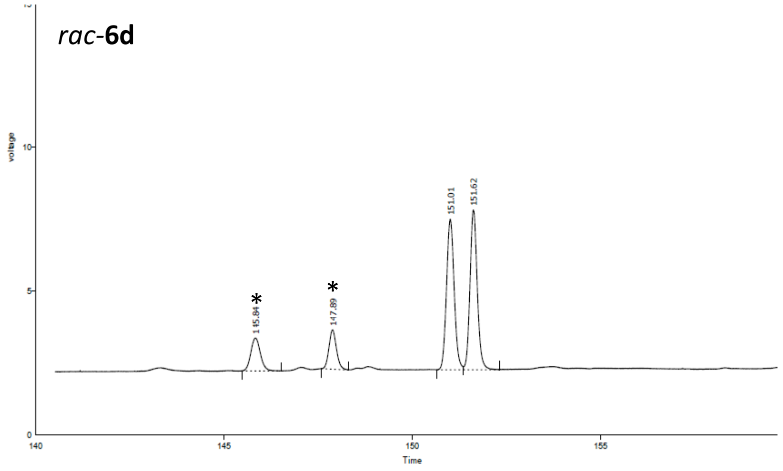


* minor diastereomer

| | Reten. Time [min] | Area [mV.s] | Height [mV] | Area [%] | Height [%] | W05 [min] |
|---|---|---|---|---|---|---|
| 1 | 145.843 | 20.256 | 1.141 | 10.8 | 8.6 | 0.27 |
| 2 | 147.890 | 19.021 | 1.371 | 10.1 | 10.3 | 0.21 |
| 3 | 151.013 | 74.250 | 5.235 | 39.5 | 39.4 | 0.22 |
| 4 | 151.623 | 74.680 | 5.553 | 39.7 | 41.8 | 0.21 |
| | Total | 188.206 | 13.300 | 100.0 | 100.0 | |

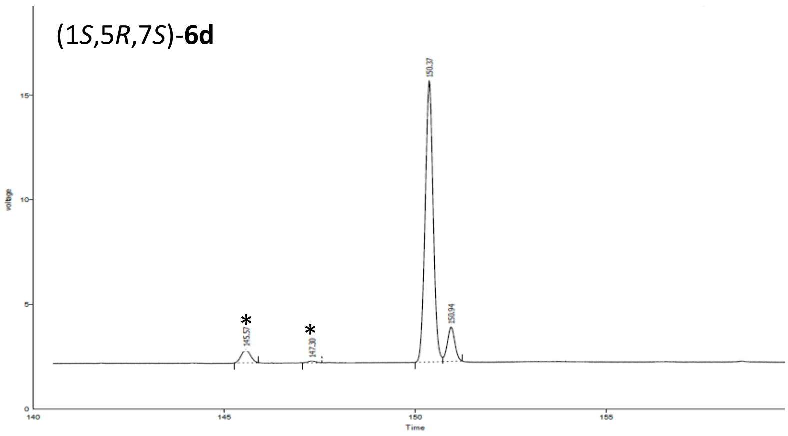


* minor diastereomer

| | Reten. Time [min] | Area [mV.s] | Height [mV] | Area [%] | Height [%] | W05 [min] |
|---|---|---|---|---|---|---|
| 1 | 145.573 | 10.699 | 0.630 | 4.6 | 4.0 | 0.27 |
| 2 | 147.297 | 1.342 | 0.093 | 0.6 | 0.6 | 0.23 |
| 3 | 150.367 | 196.283 | 13.442 | 85.1 | 85.0 | 0.23 |
| 4 | 150.940 | 22.408 | 1.642 | 9.7 | 10.4 | 0.21 |
| | Total | 230.731 | 15.807 | 100.0 | 100.0 | |

**(1*S*,6*R*,8*S*)-6-Methyl-8-(4-(trifluoromethyl)benzyl)-9-oxabicyclo[4.2.1]nonan-1-ol (6e)**[9]

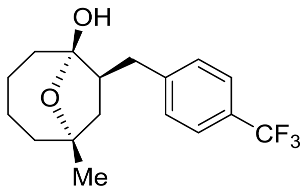


Prepared according to general procedure **E** using lactone (*R*)-**2g** (13 mg, 0.042 mmol), $SmI_2$, 0.1 M in THF (3.3 mL, 0.33 mmol) and $H_2O$ (0.59 mL, 33 mmol), followed by Dess-Martin Periodinane (21 mg, 0.049 mmol) in $CH_2Cl_2$ (0.8 mL). Purification by silica gel column chromatography (hexane/EtOAc 95:5) yielded the title product as an 86:14 mixture of diastereomers (10 mg, 0.032 mmol, 76%). Major diastereomer: $^1$H NMR (400 MHz, $CDCl_3$) δ 1.28 (s, 3 H, C$H_3$), 1.53-1.88 (m, 9 H, C$H_2$CH$_2$CH$_2$CH$_2$COH + CH$_2$C$H_2$CH$_2$CH$_2$COH + CH$_2$CH$_2$C$H_2$CH$_2$COH + CH$_2$CH$_2$CH$_2$C$H_a$H$_b$COH + C$H_2$CHCH$_2$Ar), 2.13-2.18 (m, 1 H, CH$_2$CH$_2$CH$_2$CH$_a$$H_b$COH), 2.27-2.36 (m, 1 H, C$H$CH$_2$Ar), 2.61 (dd, $J$ = 13.1, 11.3 Hz, 1 H, C$H_a$H$_b$Ar), 3.06 (dd, $J$ = 13.2, 3.6 Hz, 1 H, CH$_a$$H_b$Ar), 7.31-7.36 (m, 2 H, 2 x ArC$H$), 7.54-7.56 (m, 2 H, 2 x ArC$H$); $^{13}$C NMR (101 MHz, $CDCl_3$) 22.4 (CH$_2$CH$_2$$C$H$_2$CH$_2$COH), 25.0

($CH_2CH_2CH_2CH_2COH$), 31.1 ($CH_3$), 35.9 ($CH_2Ar$), 37.9 ($CH_2CH_2CH_2CH_2COH$), 41.8 ($CH_2CH_2CH_2CH_2COH$), 42.7 ($CH_2CHCH_2Ar$), 51.5 ($CHCH_2Ar$), 78.9 ($CH_3CO$), 107.7 (OCOH), 125.4 (q, *J* = 2.9 Hz, 2 x Ar*C*H), 128.9 (2 x Ar*C*H), 129.1 (q, *J* = 32.3 Hz, Ar*C*$CF_3$), 144.9 (Ar*C*), ArC$CF_3$ not observed; $^{19}$F NMR (376 MHz, $CDCl_3$) -62.3 ppm; Specific rotation $[\alpha]_D^{26}$ -27.7 (*c* 1.1, $CHCl_3$) for an enantiomerically enriched sample of >99% e.e

Enantiomeric purity of (1*S*,6*R*,8*S*)-**6e** was determined by HPLC analysis in comparison with authentic racemic material (>99:1 e.r. shown; Chiralcel OD-H column, 99:1 hexanes:$^i$PrOH, 0.25 mL min$^{-1}$, 20°C, 220 nm).

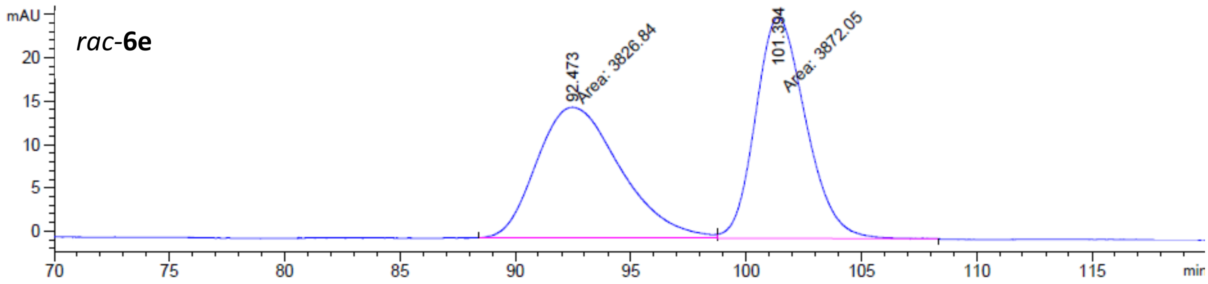


| Peak # | RetTime [min] | Type | Width [min] | Area [mAU*s] | Height [mAU] | Area % |
|---|---|---|---|---|---|---|
| 1 | 92.473 | MF | 4.2147 | 3826.84229 | 15.13297 | 49.7064 |
| 2 | 101.394 | FM | 2.5278 | 3872.05249 | 25.52990 | 50.2936 |

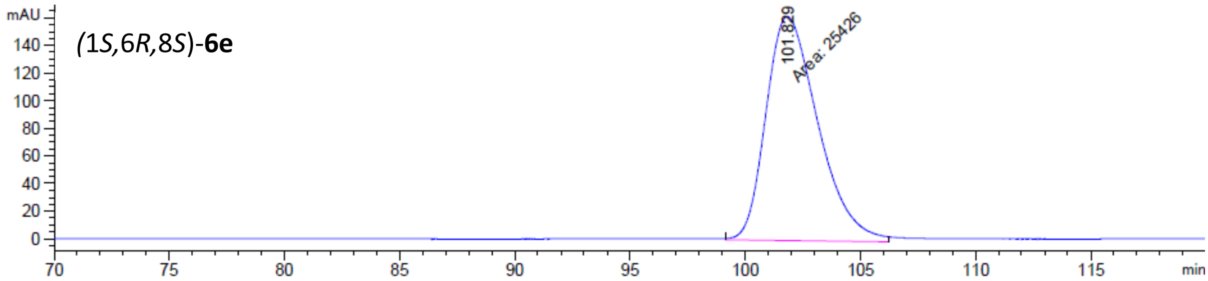


| Peak # | RetTime [min] | Type | Width [min] | Area [mAU*s] | Height [mAU] | Area % |
|---|---|---|---|---|---|---|
| 1 | 101.829 | MM | 2.6156 | 2.54260e4 | 162.01291 | 100.0000 |

**General Procedure F: $SmI_2$-HMPA-mediated radical ketone cyclisation**

**(1*S*,6*S*,8*R*)-8-Benzyl-6-methylbicyclo[4.2.0]octan-1-ol (7a)**

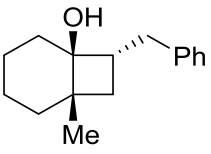


To a solution of $SmI_2$, 0.1 M in THF (1.7 mL, 0.17 mmol) was added degassed HMPA (0.12 mL, 0.68 mmol) at -78°C to generate a deep purple solution. The solution was stirred for 5 minutes before a

solution of ketone (*S*)-**1b** (13 mg, 0.056 mmol) in THF was added slowly. The reaction was allowed to warm to room temperature and stirred until decolourisation occurred. A saturated aqueous solution of Rochelle's salt (2 mL) was added and the aqueous layer was extracted with $Et_2O$ (3 x 2 mL). The organic layers were combined and washed with HCl (2 x 5 mL), $NaHCO_3$ (3 mL) and NaCl (3 mL), dried ($MgSO_4$) and concentrated. Purification by silica gel column chromatography (hexane/EtOAc 95:5) yielded the title product as a single diastereomer and as a pale yellow oil (11 mg, 0.48 mmol, 84%). $^1$H NMR (500 MHz, $CDCl_3$) δ 1.11 (s, 3 H, C*H*$_3$), 1.22-1.28 (m, 1 H, C$H_aH_b$$CH_2CH_2CH_2$COH), 1.32-1.44 (m, 5 H, $CH_aH_b$$CH_2CH_2CH_2$COH + $CH_2CH_aH_b$$CH_2CH_2$COH + $CH_2CH_2CH_aH_b$$CH_2$COH + C$H_2$CH$CH_2$Ar), 1.55-1.63 (m, 2 H, $CH_2CH_aH_b$$CH_2CH_2$COH + $CH_2CH_2CH_aH_b$$CH_2$COH), 1.68-1.71 (m, 1 H, $CH_2CH_2CH_2CH_aH_b$COH), 1.82-1.88 (m, 1 H, $CH_2CH_2CH_2CH_aH_b$COH), 2.41-2.48 (m, 1 H, C*H*$CH_2$Ar), 2.59 (dd, *J* = 13.9, 9.1 Hz, 1 H, CH$CH_aH_b$Ar), 2.85 (dd, *J* = 13.9, 6.0 Hz, 1 H, CH$CH_aH_b$Ar), 7.16-7.20 (m, 3 H, 3 x ArC*H*), 7.26-7.29 (m, 2 H, 2 x ArC*H*); $^{13}$C NMR (126 MHz, $CDCl_3$) δ 20.3 ($CH_2CH_2CH_2CH_2$COH), 22.1 ($CH_2CH_2CH_2CH_2$COH), 23.4 (*C*$H_3$), 30.6 (*C*$H_2$CH$CH_2$Ar), 30.9 ($CH_2CH_2CH_2CH_2$COH), 33.5 (*C*$H_2CH_2CH_2CH_2$COH), 35.5 (CH*C*$H_2$Ar), 40.3 (*C*$CH_3$), 46.9 (*C*H$CH_2$Ar), 74.9 (*C*OH), 125.7 (Ar*C*H), 128.3 (2 x Ar*C*H), 128.5 (2 x Ar*C*H), 141.4 (Ar*C*); IR $\nu_{max}$ (thin film, cm$^{-1}$): 3427 (O-H), 3026, 2926, 1494, 1453, 1062 989; HRMS calcd. for $C_{16}H_{22}OK$ $[M+K]^+$: 269.1302, found 269.1300; Specific rotation $[\alpha]_D^{26}$ +27.7 (*c* 1.4, $CHCl_3$) for an enantiomerically enriched sample of >99% e.e.

The relative stereochemistry of the product was assigned by nOe analysis:

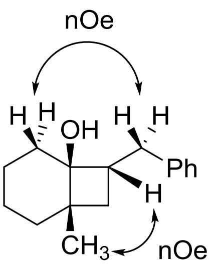


Enantiomeric purity of (1*S*,6*S*,8*R*)-**7a** was determined by HPLC analysis in comparison with authentic racemic material (>99:1 e.r. shown, Lux 5 µm Amylose-1 column, 80:20 hexanes:$^i$PrOH, 1 mL min$^{-1}$, 20°C, 220 nm).

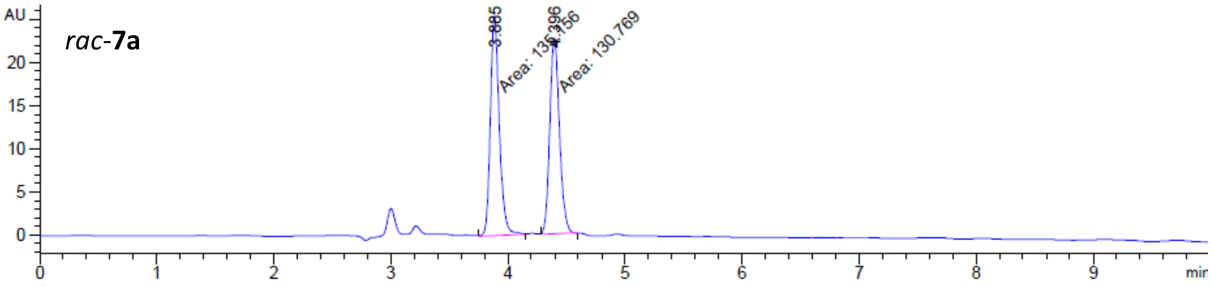

| Peak # | RetTime [min] | Type | Width [min] | Area [mAU*s] | Height [mAU] | Area % |
|---|---|---|---|---|---|---|
| 1 | 3.885 | MM | 0.0884 | 135.15642 | 25.48845 | 50.8249 |
| 2 | 4.396 | MM | 0.0958 | 130.76918 | 22.75356 | 49.1751 |

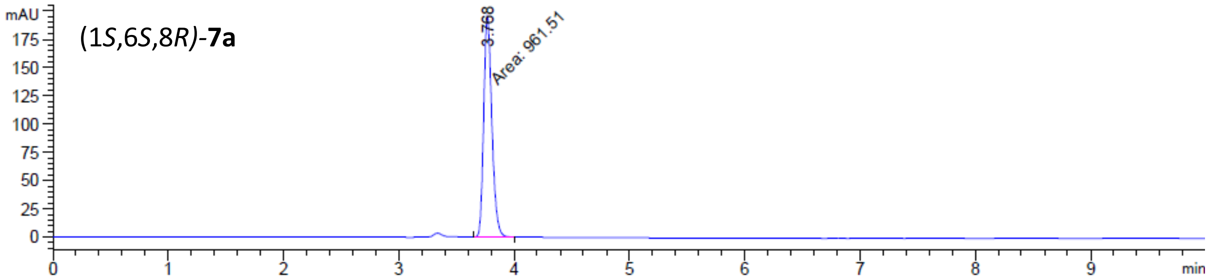


| Peak # | RetTime [min] | Type | Width [min] | Area [mAU*s] | Height [mAU] | Area % |
|---|---|---|---|---|---|---|
| 1 | 3.768 | MM | 0.0820 | 961.51044 | 195.40604 | 100.0000 |

**(1*S*,6*S*,8*S*)-8-(2-Chlorobenzyl)-6-methylbicyclo[4.2.0]octan-1-ol (7b)**

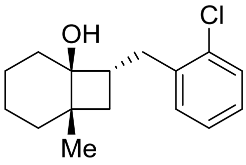


Prepared according to general procedure **F** using (*S*)-**1f** (19 mg, 0.072 mmol), $SmI_2$ (2.2 mL, 0.22 mmol) and HMPA (0.15 mL, 0.87 mmol). Purification by silica gel column chromatography (hexane/EtOAc 95:5) yielded the title product as a pale yellow oil (14 mg, 0.053 mmol, 73%). $^1$H NMR (500 MHz, $CDCl_3$) δ 1.12 (s, 3 H, C*H*$_3$), 1.21-1.43 (m, 6 H, C*H*$_2$CH$_2$CH$_2$CH$_2$COH + CH$_2$CH$_2$C*H*$_2$CH$_2$COH + CH$_2$CH$_2$CH$_2$C*H*$_2$COH), 1.59-1.65 (m, 2 H, CH$_2$C*H*$_2$CH$_2$CH$_2$COH), 1.70-1.74 (m, 1 H, C*H*$_a$H$_b$CHCH$_2$Ar), 1.83-1.89 (m, 1 H, CH$_a$*H*$_b$CHCH$_2$Ar), 2.50 (qd, *J* = 9.3, 5.6 Hz, 1 H, C*H*CH$_2$Ar), 2.72 (dd, *J* = 14.2, 9.0 Hz, 1 H, CHC*H*$_a$H$_b$Ar), 2.98 (dd, *J* = 14.2, 5.6 Hz, 1 H, CHCH$_a$*H*$_b$Ar), 7.12 (td, *J* = 7.6, 1.8 Hz, 1 H, ArC*H*), 7.17 (t, *J* = 7.4 Hz, 1 H, ArCH), 7.27-7.27 (m, 1 H, ArC*H*), 7.33 (d, *J* = 7.8 Hz, 1 H, ArC*H*); $^{13}$C NMR (126 MHz, $CDCl_3$) δ 20.4 (CH$_2$*C*H$_2$CH$_2$CH$_2$COH), 22.1 (CH$_2$CH$_2$*C*H$_2$CH$_2$COH), 23.4 (*C*H$_3$), 30.4 (CH$_2$CH$_2$CH$_2$*C*H$_2$COH), 30.9 (*C*H$_2$CHCH$_2$Ar), 32.8 (CH*C*H$_2$Ar), 33.5 (*C*H$_2$CH$_2$CH$_2$CH$_2$COH), 40.5 (*C*CH$_3$), 45.3 (*C*HCH$_2$Ar), 74.9 (*C*OH), 126.6 (Ar*C*H), 127.1 (Ar*C*H), 129.4 (Ar*C*H), 130.4 (Ar*C*H), 133.8 (Ar*C*Cl), 138.9 (Ar*C*) ppm; IR $\nu_{max}$ (thin film, cm$^{-1}$): 3430 (O-H), 2950, 2848, 1495, 1452, 1077, 1020; HRMS calcd. for $C_{16}H_{21}OClNa$ [M+Na]$^+$: 287.1173, found 287.1167; Specific rotation $[\alpha]_D^{27}$ -30.7 (*c* 0.4, $CHCl_3$) for an enantiomerically enriched sample of 74% e.e.

Enantiomeric purity of (1*S*,6*S*,8S*)*-**7b** was determined by HPLC analysis in comparison with authentic racemic material (87:13 e.r. shown, Lux 5 µm Amylose-1 column, 95:5 hexanes:$^i$PrOH, 1 mL min$^{-1}$, 20°C, 220 nm).

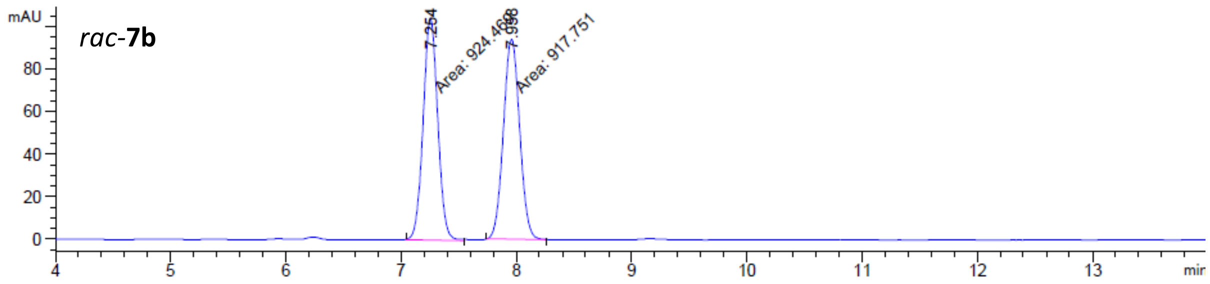


| Peak # | RetTime [min] | Type | Width [min] | Area [mAU*s] | Height [mAU] | Area % |
|---|---|---|---|---|---|---|
| 1 | 7.254 | MM | 0.1474 | 924.46863 | 104.52144 | 50.1823 |
| 2 | 7.958 | MM | 0.1625 | 917.75104 | 94.15018 | 49.8177 |

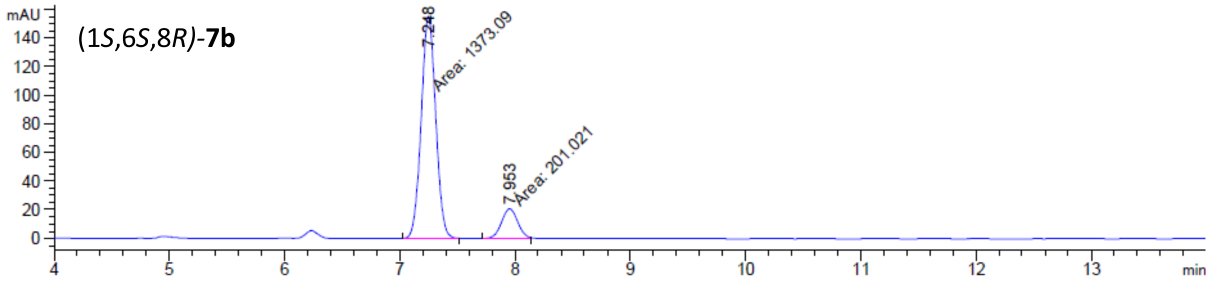


| Peak # | RetTime [min] | Type | Width [min] | Area [mAU*s] | Height [mAU] | Area % |
|---|---|---|---|---|---|---|
| 1 | 7.248 | MM | 0.1471 | 1373.08960 | 155.59962 | 87.2295 |
| 2 | 7.953 | MM | 0.1626 | 201.02135 | 20.60976 | 12.7705 |

**(1*S*,5*S*,7*R*)-7-Benzyl-5-methylbicyclo[3.2.0]heptan-1-ol (7c)**

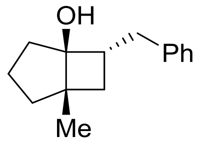


Prepared according to general procedure **F** using (*S*)-**4b** (18 mg, 0.084 mmol), $SmI_2$ (2.5 mL, 0.25 mmol) and HMPA (0.18 mL, 1.01 mmol). Purification by silica gel column chromatography yielded the title product as a colourless oil (12 mg, 0.056 mmol, 66%). $^1$H NMR (400 MHz, $CDCl_3$) δ 1.13 (s, 3 H, $CH_3$), 1.39-1.64 (m, 5 H, $CH_2CH_2CH_2COH$ + $CH_2CH_2CH_2COH$ + $CH_aH_bCHCH_2Ar$), 1.65-1.84 (m, 2 H, $CH_2CH_2CH_2COH$), 2.24 (dd, *J* = 13.4, 6.6 Hz, 1 H, $CH_aH_bCHCH_2Ar$), 2.46-2.55 (m, 1 H, $CHCH_2Ar$), 2.60 (dd, *J* = 13.6, 9.6 Hz, 1 H, $CHCH_aH_bAr$), 2.86 (dd, *J* = 13.6, 5.6 Hz, 1 H, $CHCH_aH_bAr$), 7.16-7.21 (m, 3 H, 3 x ArCH), 7.25-7.30 (m, 2 H, 2 x ArCH); $^{13}$C NMR (101 MHz, $CDCl_3$) δ 19.9 ($CH_3$), 23.5 ($CH_2CH_2CH_2COH$), 31.4 ($CH_2CH_2CH_2COH$), 35.5 ($CHCH_2Ar$), 35.6 ($CH_2CHCH_2Ar$), 38.3 ($CH_2CH_2CH_2COH$), 44.2 ($CHCH_2Ar$), 46.0 ($CCH_3$), 83.7 (*C*OH), 125.7 (Ar*C*H), 128.3 (2 x Ar*C*H), 128.5 (2 x Ar*C*H), 140.9 (Ar*C*) ppm; IR $\nu_{max}$ (thin film, cm$^{-1}$): 3455 (O-H), 2927, 2857, 1453, 1442, 1157, 1051, 1040; HRMS calcd. for $C_{16}H_{22}OK$ $[M+K]^+$: 269.1302, found 269.1300; HRMS calcd. for $C_{15}H_{20}OK$ $[M+K]^+$: 255.1146, found 255.1139; Specific rotation $[\alpha]_D^{27}$ -49.9 (*c* 1.0, $CHCl_3$) for an enantiomerically enriched sample of 70% e.e.

Enantiomeric purity of (1*S*,5*S*,7*R*)-**7c** was determined by HPLC analysis in comparison with authentic racemic material (85:15 e.r. shown, Lux 5 µm Amylose-1 column, 95:5 hexanes:$^{i}$PrOH, 1 mL min$^{-1}$, 20°C, 220 nm).

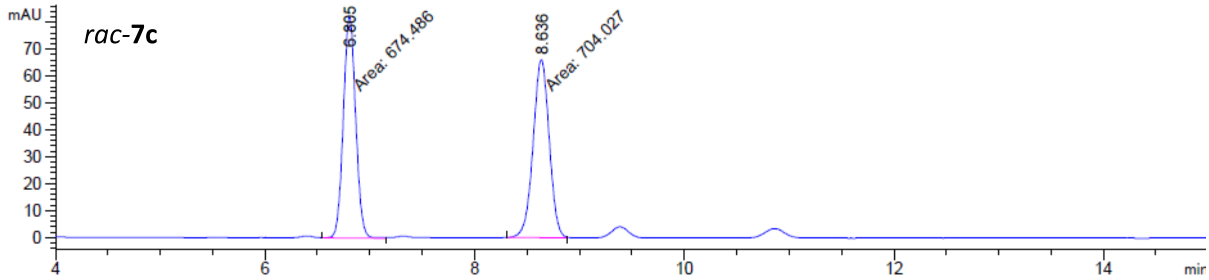


| Peak # | RetTime [min] | Type | Width [min] | Area [mAU*s] | Height [mAU] | Area % |
|---|---|---|---|---|---|---|
| 1 | 6.805 | MM | 0.1364 | 674.48639 | 82.43330 | 48.9285 |
| 2 | 8.636 | MM | 0.1775 | 704.02734 | 66.11521 | 51.0715 |

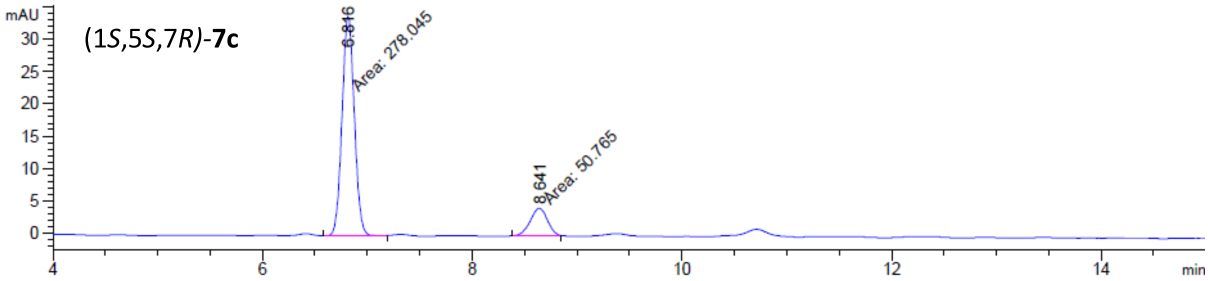


| Peak # | RetTime [min] | Type | Width [min] | Area [mAU*s] | Height [mAU] | Area % |
|---|---|---|---|---|---|---|
| 1 | 6.816 | MM | 0.1363 | 278.04453 | 33.98831 | 84.5610 |
| 2 | 8.641 | MM | 0.1957 | 50.76503 | 4.32409 | 15.4390 |

## 6. NMR Spectra

### 2-Methyl-2-((3-phenyloxiran-2-yl)methyl)cyclohexan-1-one (S1)

500MHz, $CDCl_3$

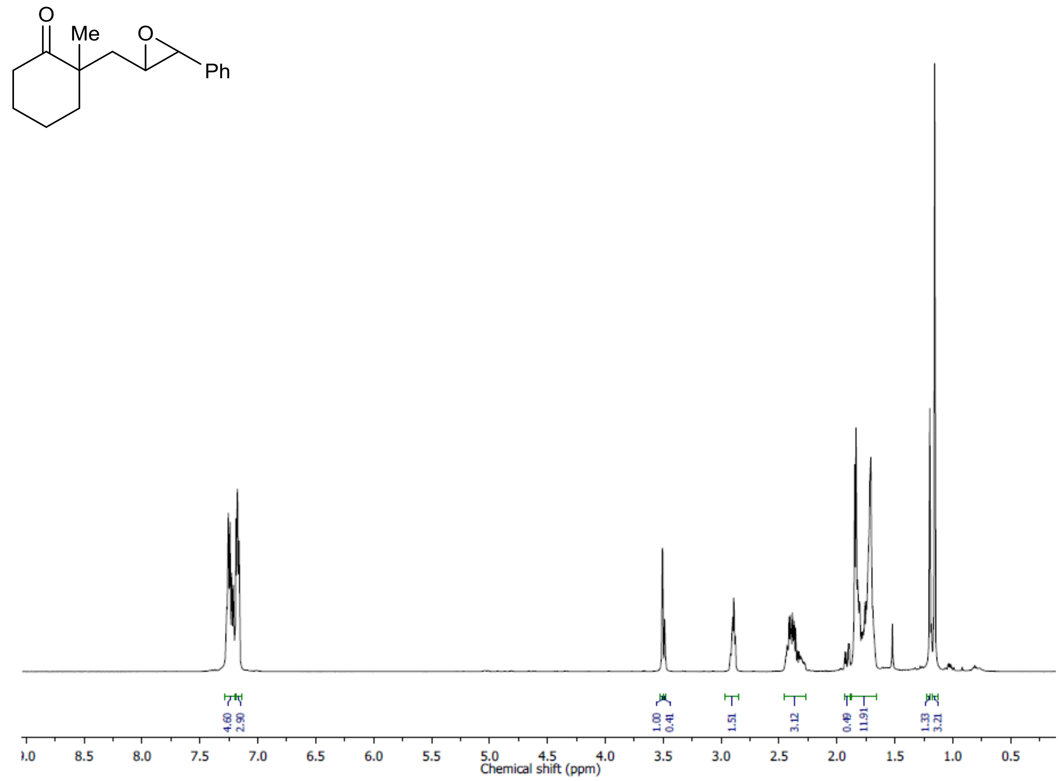


126 MHz, $CDCl_3$

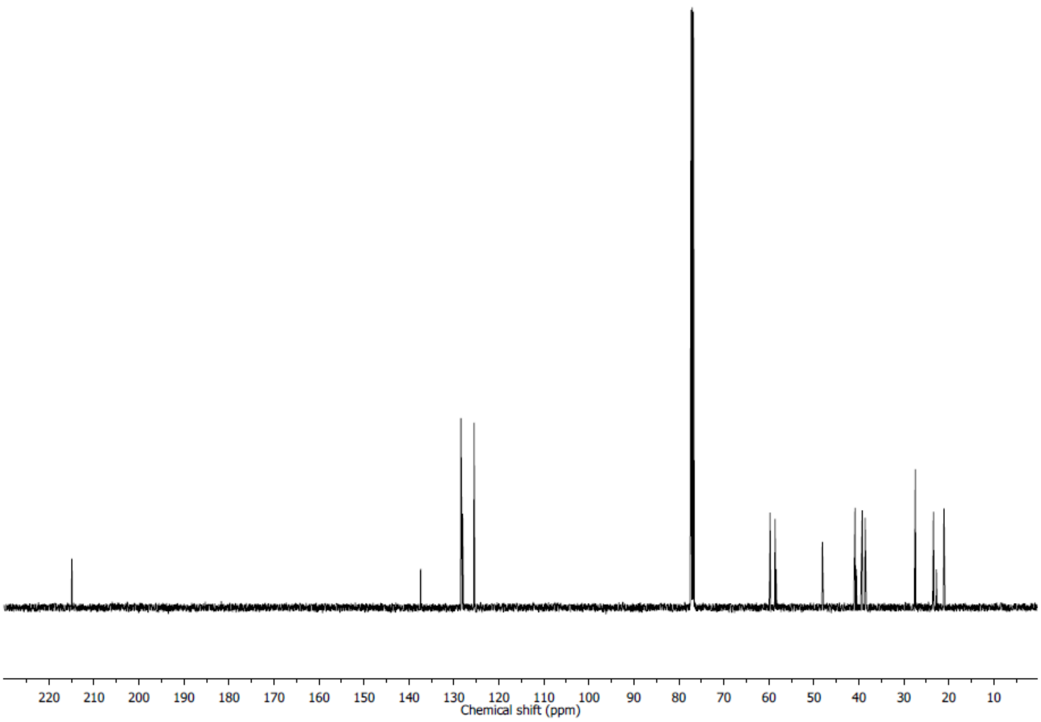

**7-Methyl-7-((3-phenyloxiran-2-yl)methyl)oxepan-2-one (S2)**

400 MHz, $CDCl_3$

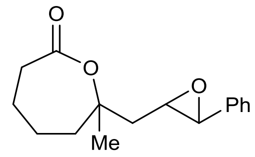


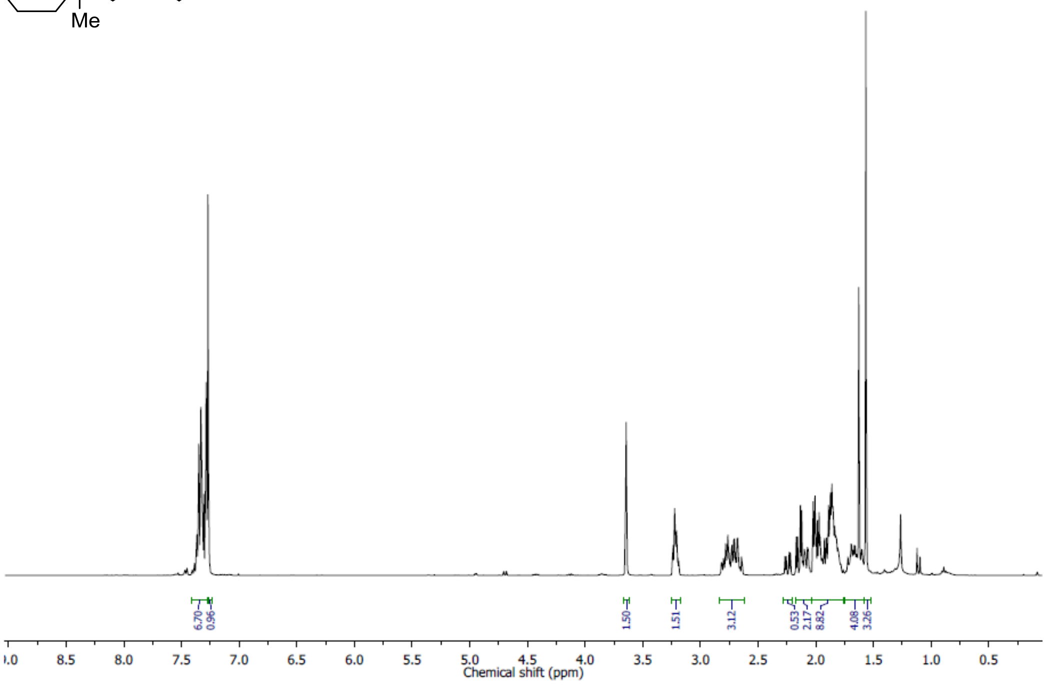


101 MHz, $CDCl_3$

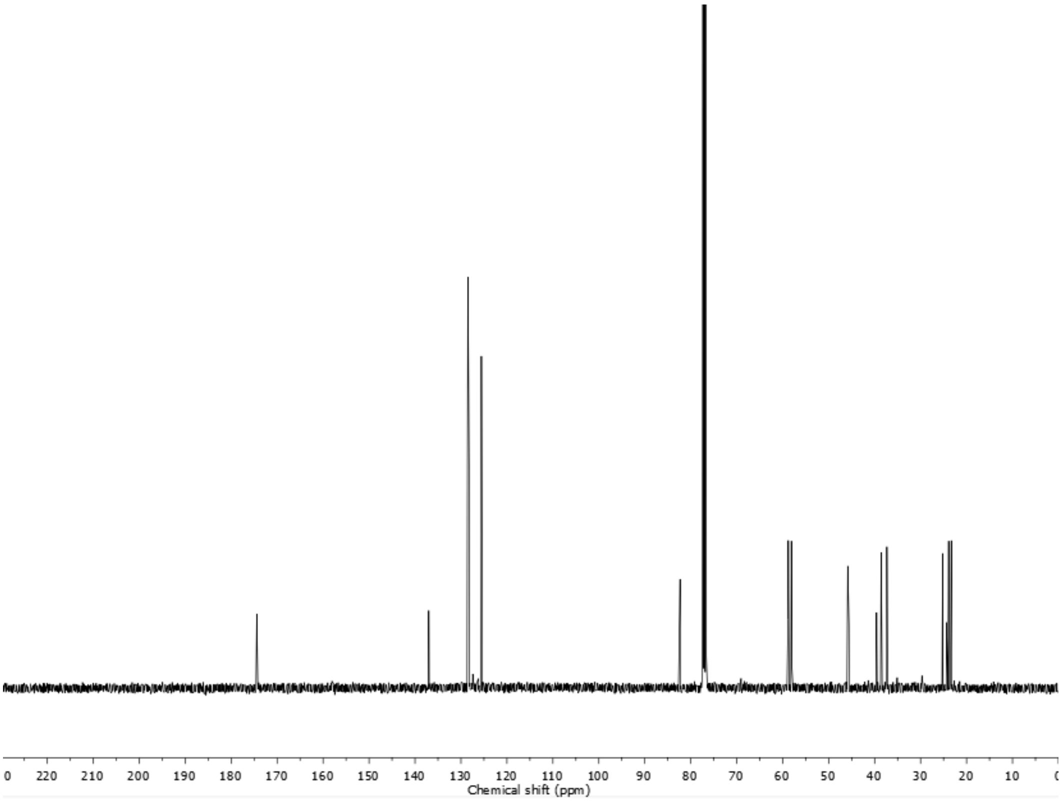

**2-Allyl-2-methylcyclohexan-1-one (1a)**

400 MHz, $CDCl_3$

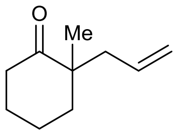


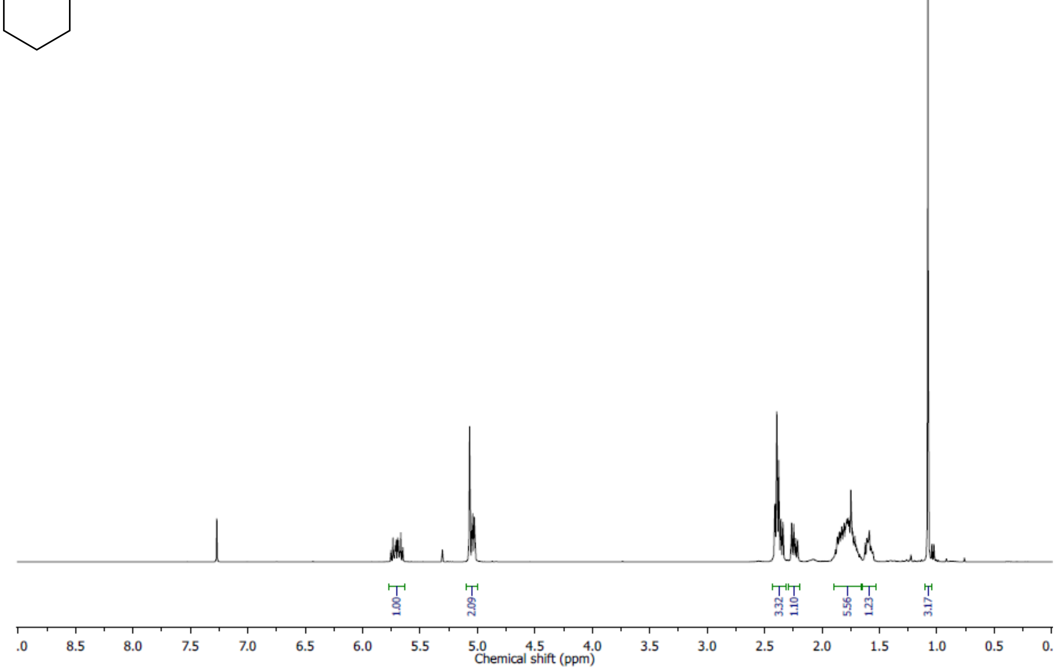


101 MHz, $CDCl_3$

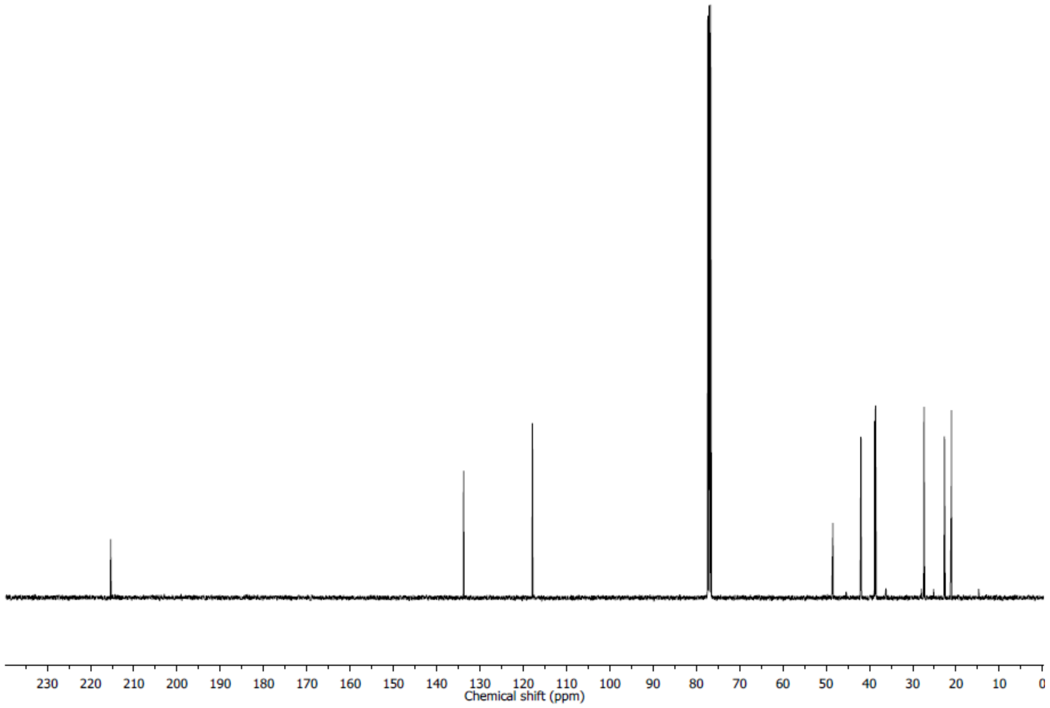

**(*E*)-2-Cinnamyl-2-methylcyclohexan-1-one (1b)**

400 MHz, $CDCl_3$

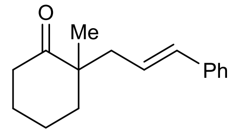


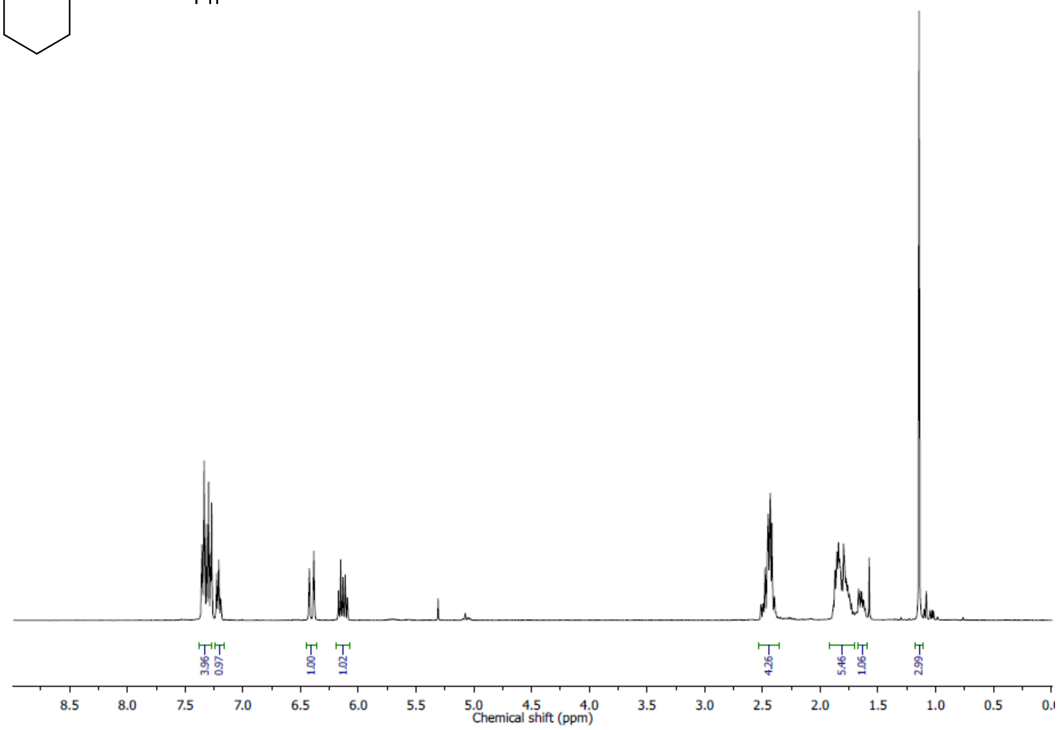


101 MHz, $CDCl_3$

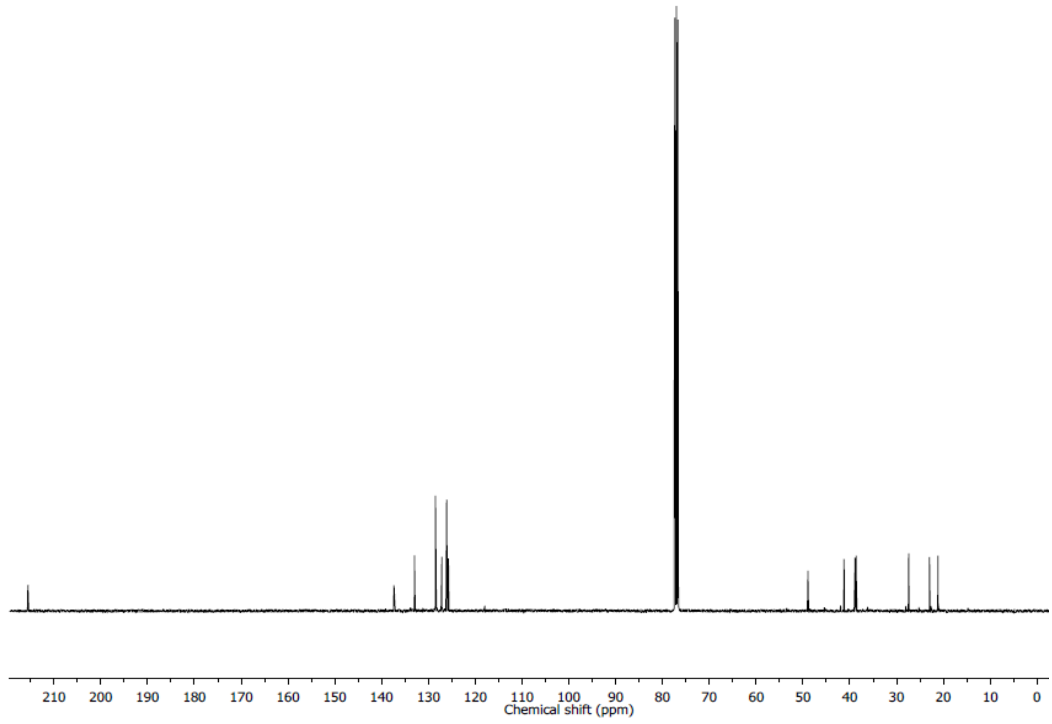

**(*E*)-2-(3-(4-Fluorophenyl)allyl)-2-methylcyclohexan-1-one (1c)**

400 MHz, $CDCl_3$

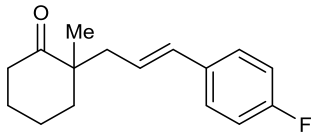


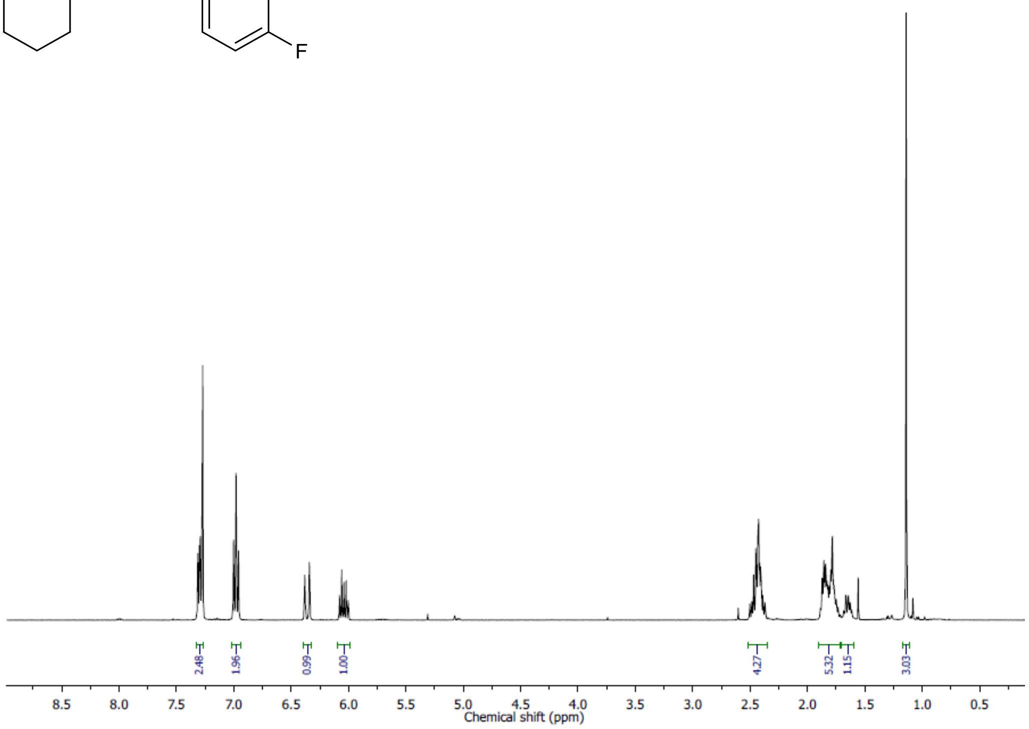


101 MHz, $CDCl_3$

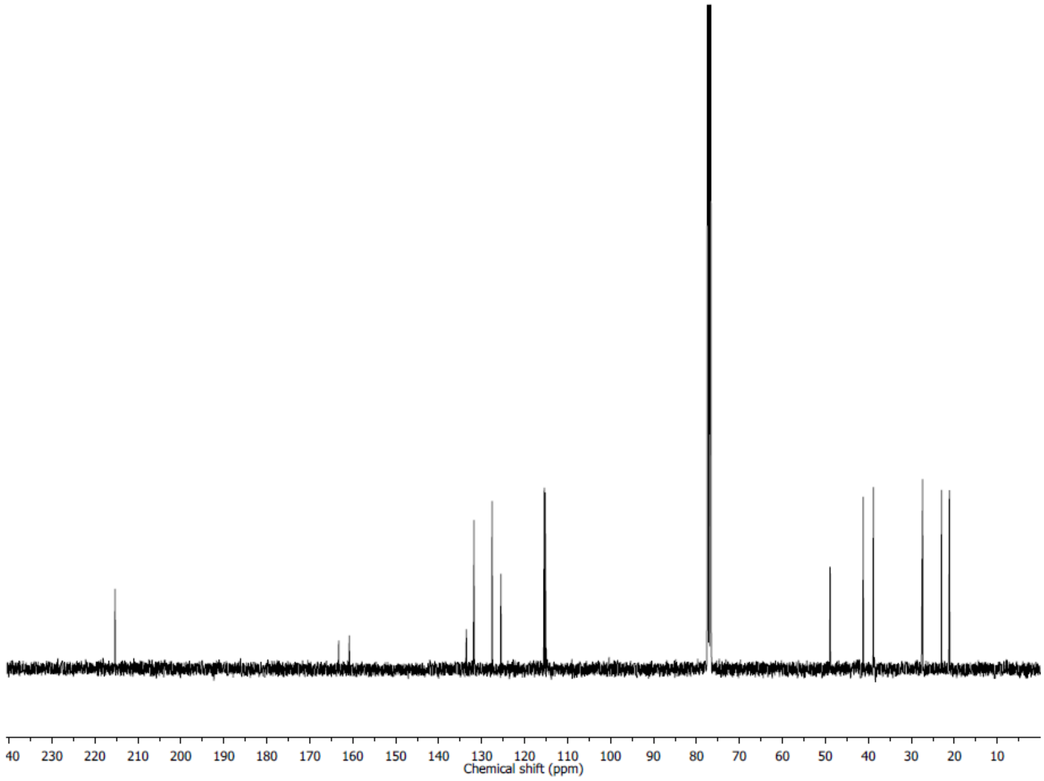

**(*E*)-2-Methyl-2-(3-(naphthalen-2-yl)allyl)cyclohexan-1-one (1d)**

500 MHz, $CDCl_3$

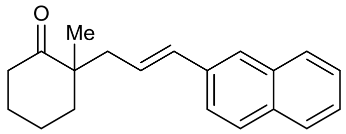


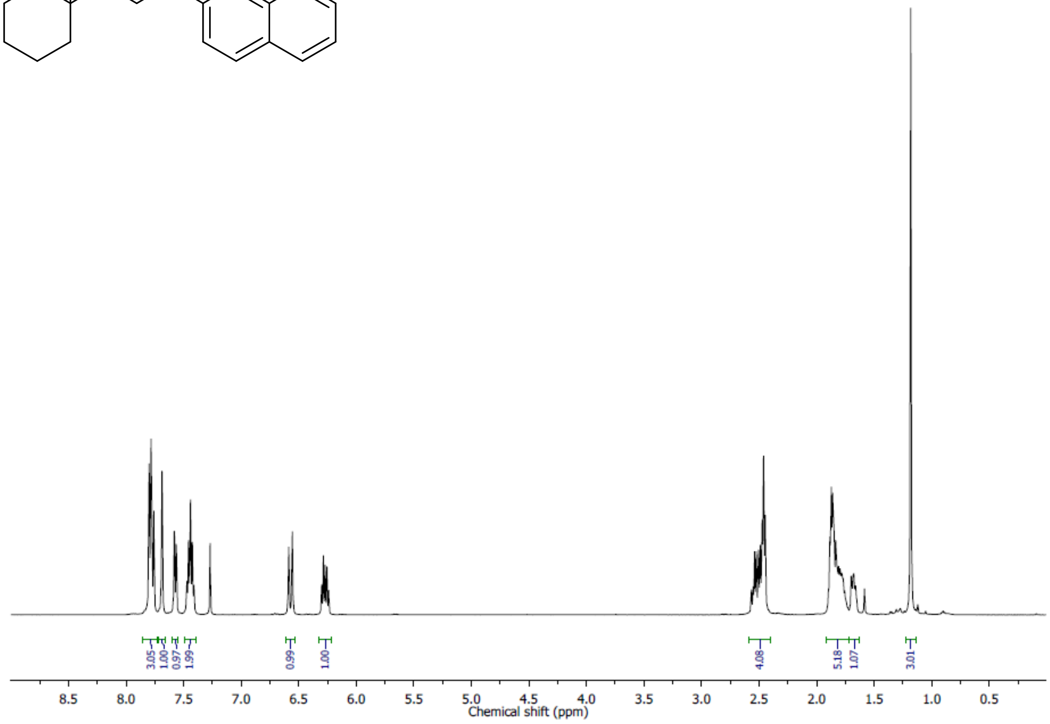


126 MHz, $CDCl_3$

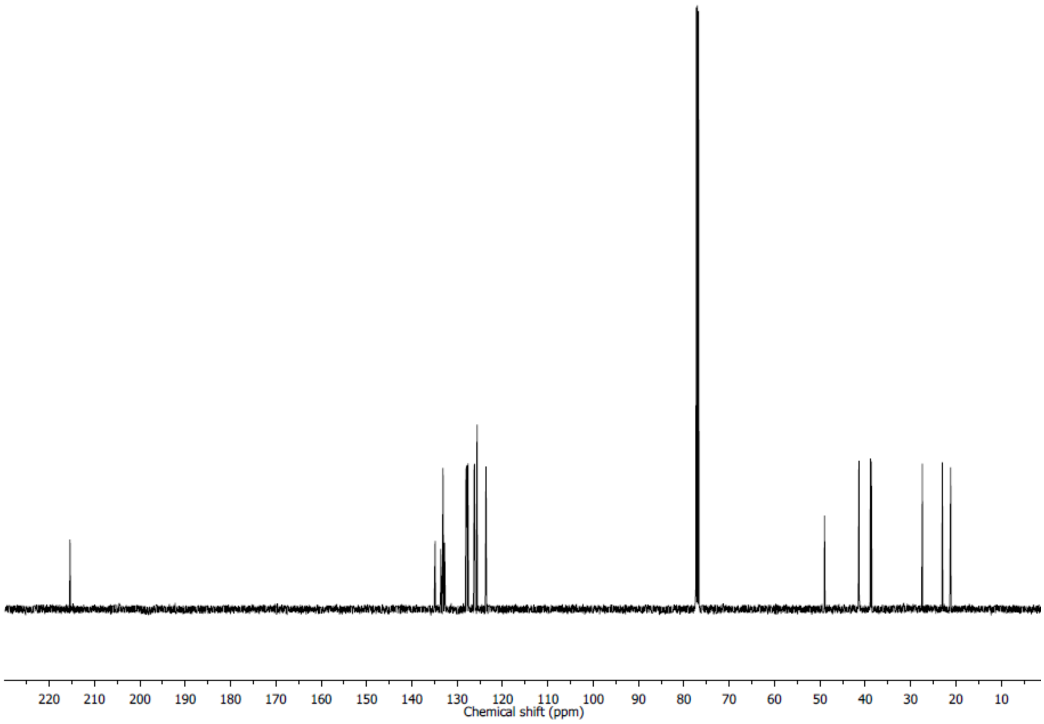

**(*E*)-2-(3-(3-Bromophenyl)allyl)-2-methylcyclohexan-1-one (1e)**

400 MHz, $CDCl_3$

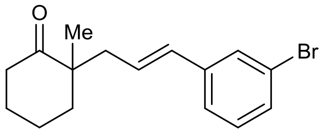


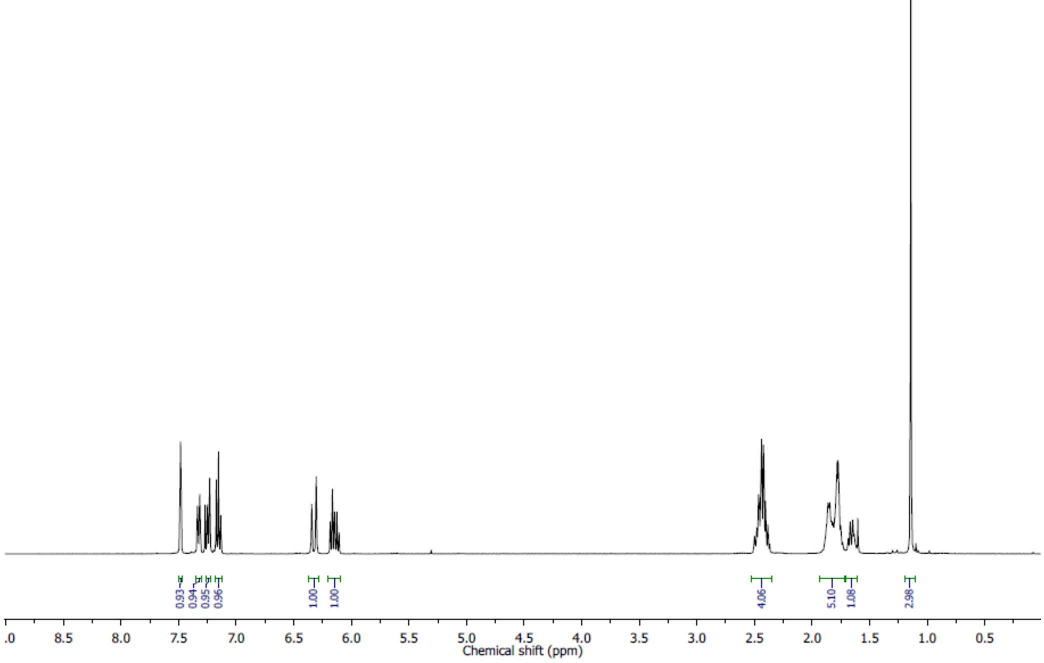


101 MHz, $CDCl_3$

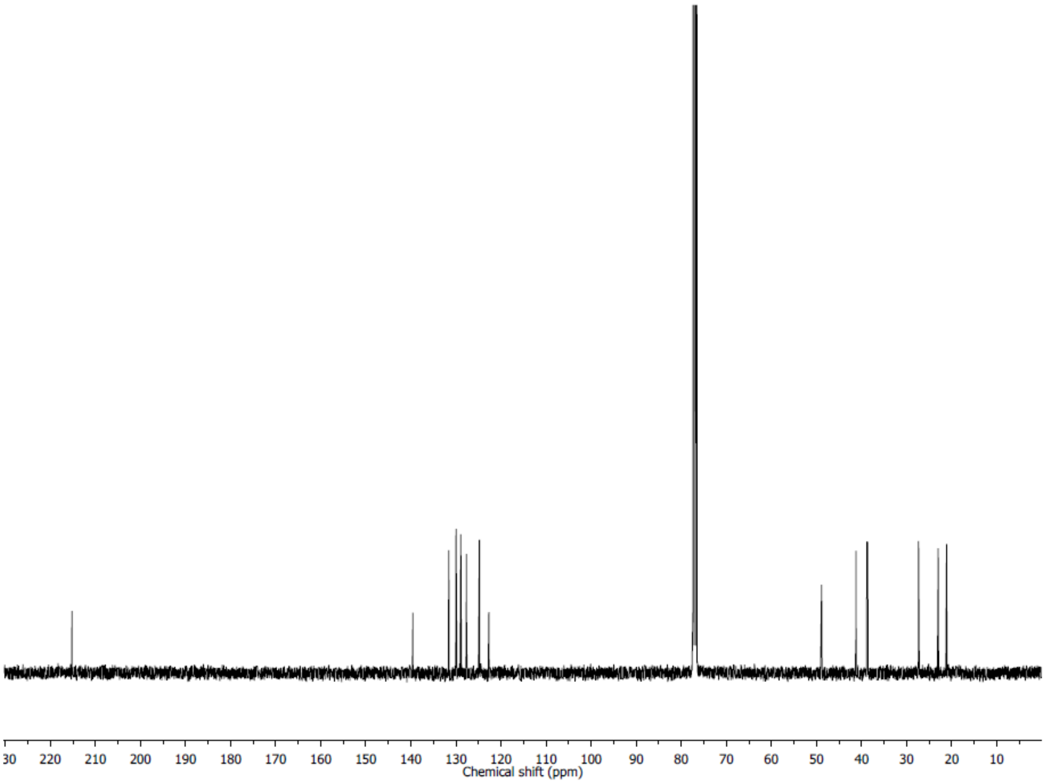

**(*E*)-2-(3-(2-Chlorophenyl)allyl)-2-methylcyclohexan-1-one (1f)**

400 MHz, $CDCl_3$

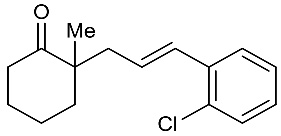


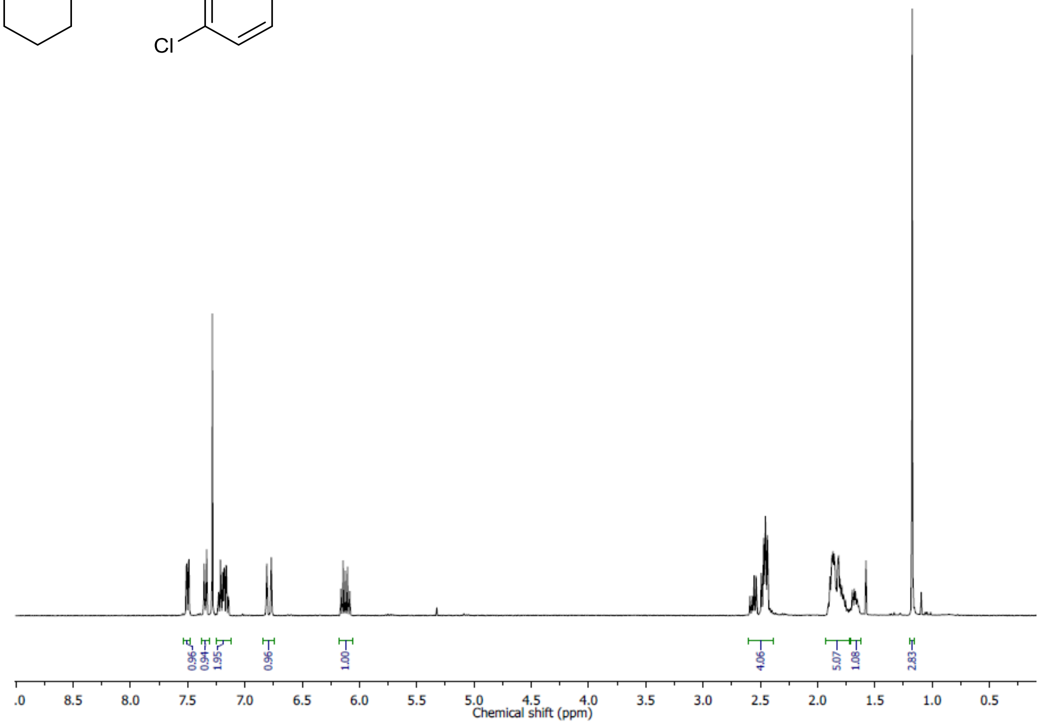


101 MHz, $CDCl_3$

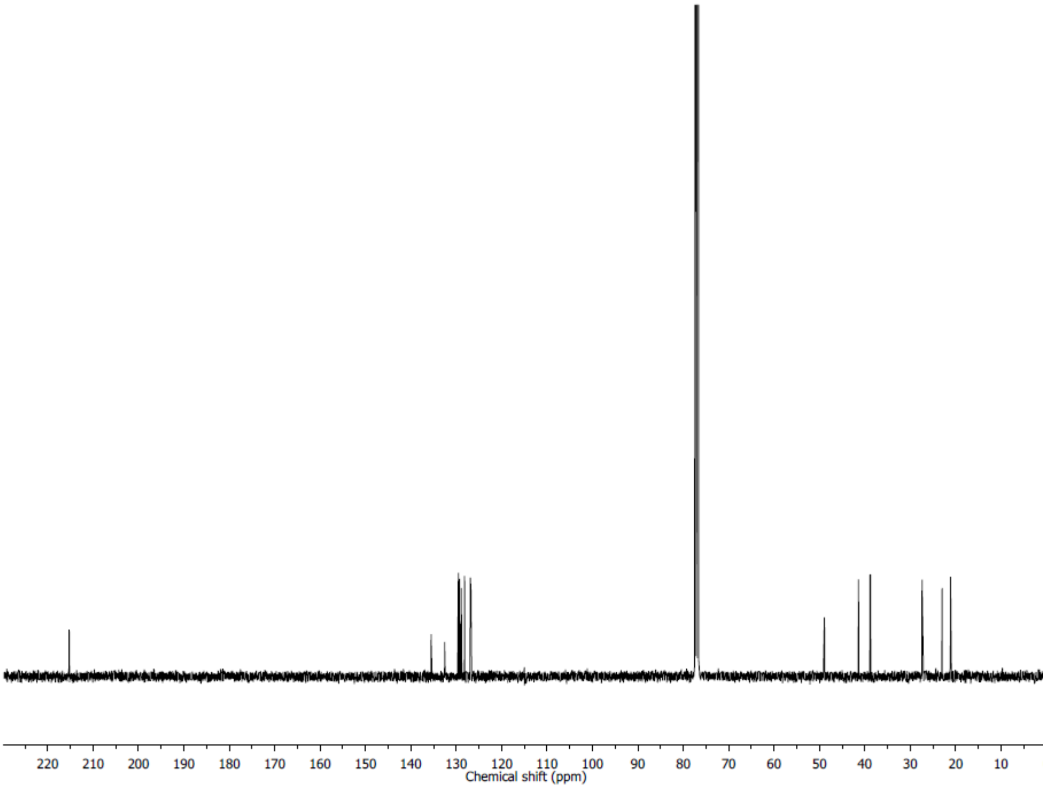

**(*E*)-2-Methyl-2-(3-(4-(trifluoromethyl)phenyl)allyl)cyclohexan-1-one (1g)**

400 MHz, $CDCl_3$

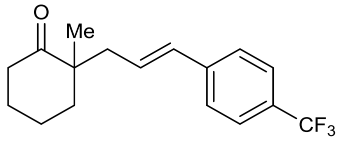


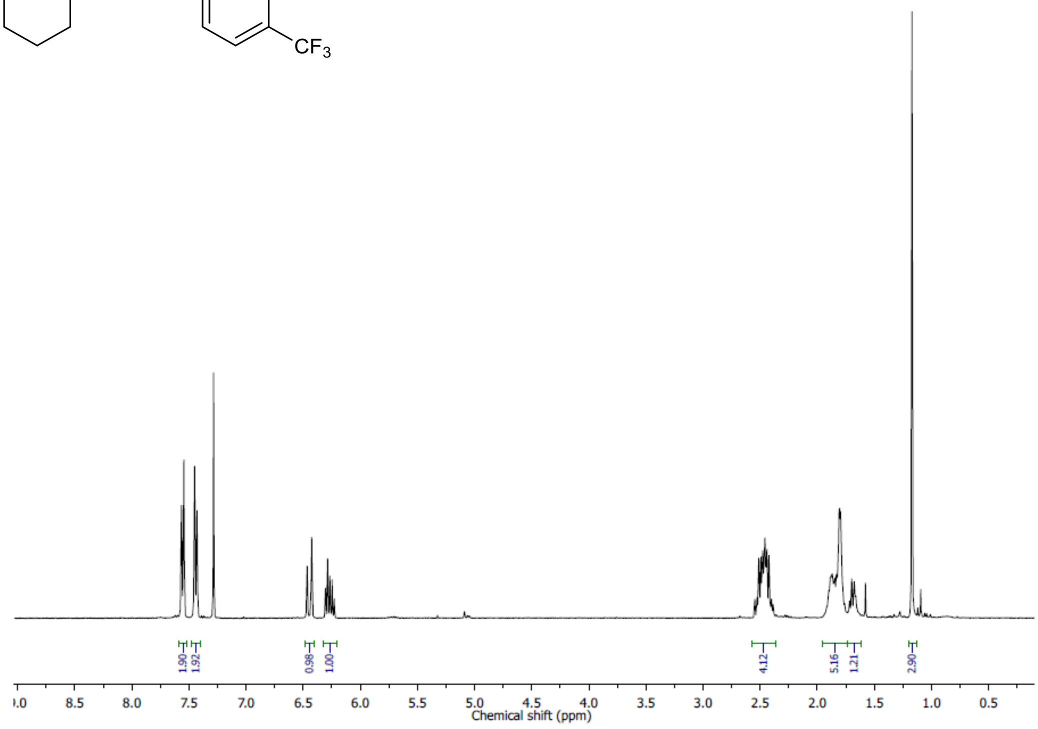


101 MHz, $CDCl_3$

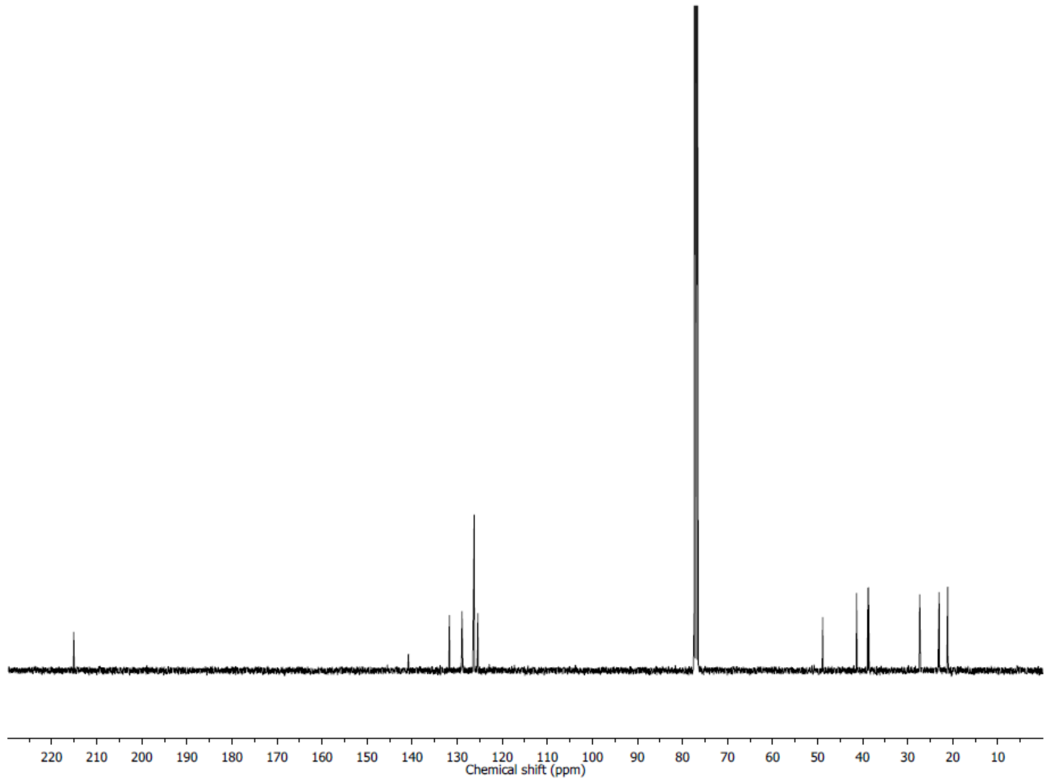

**(*E*)-2-Methyl-2-(3-(*o*-tolyl)allyl)cyclohexan-1-one (1h)**

400 MHz, $CDCl_3$

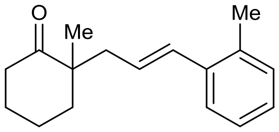


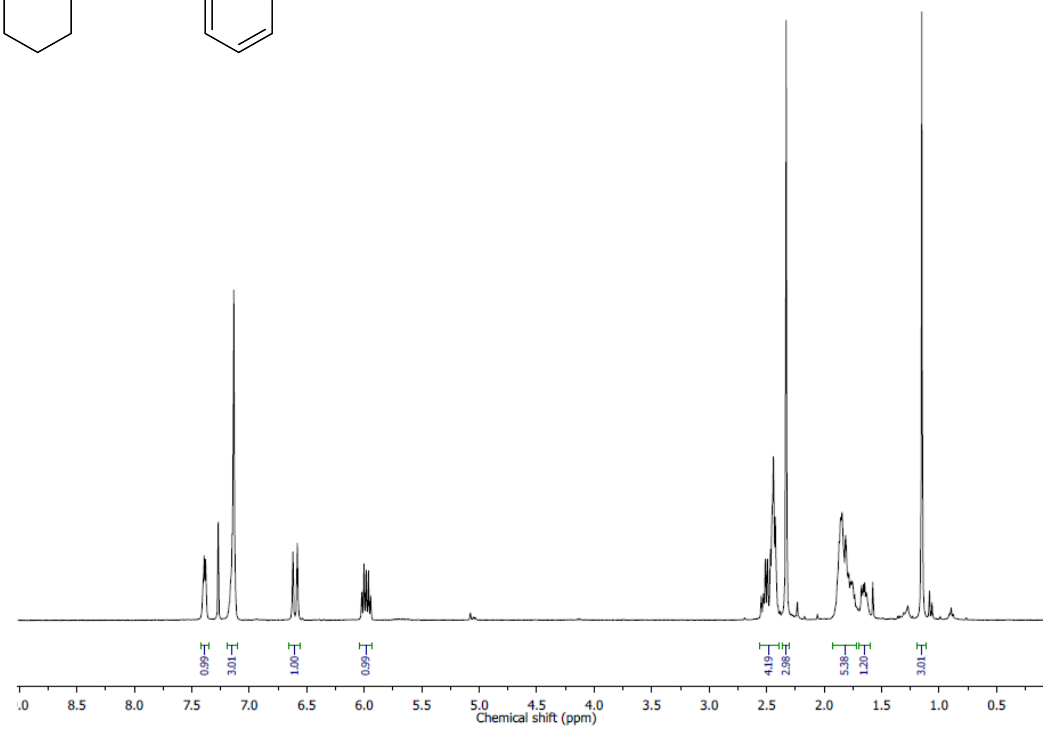


101 MHz, $CDCl_3$

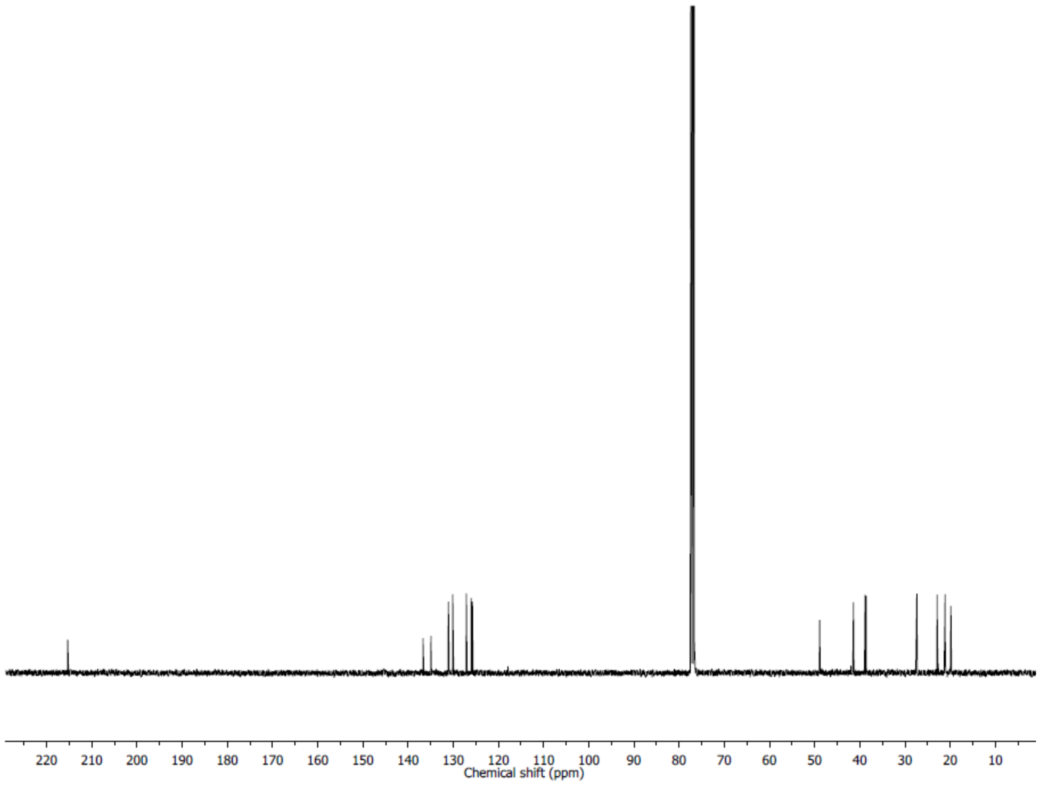

**2-Allyl-2-ethylcyclohexan-1-one (1i)**

400 MHz, $CDCl_3$

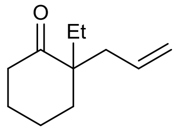


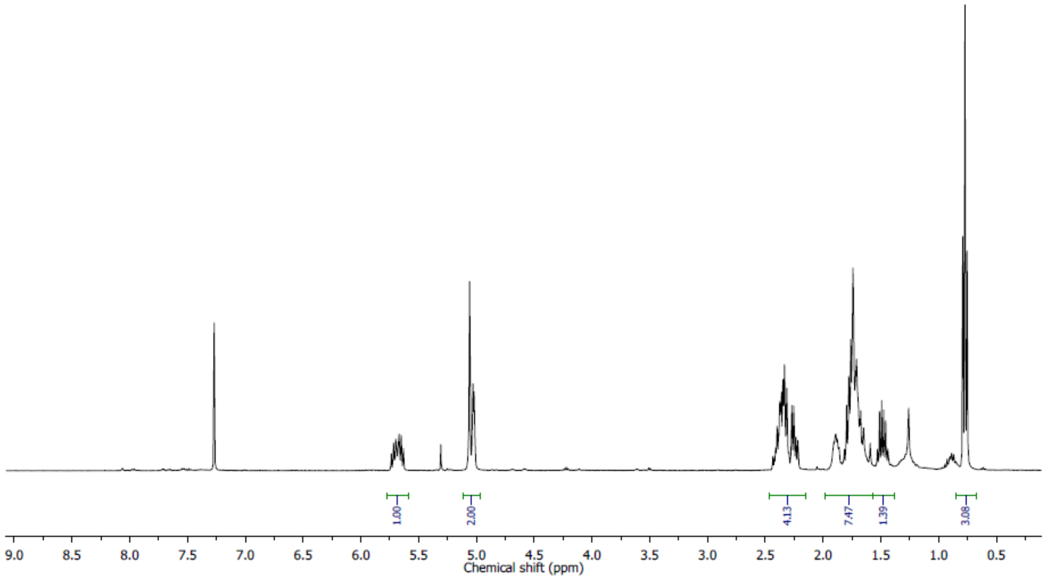


101 MHz, $CDCl_3$

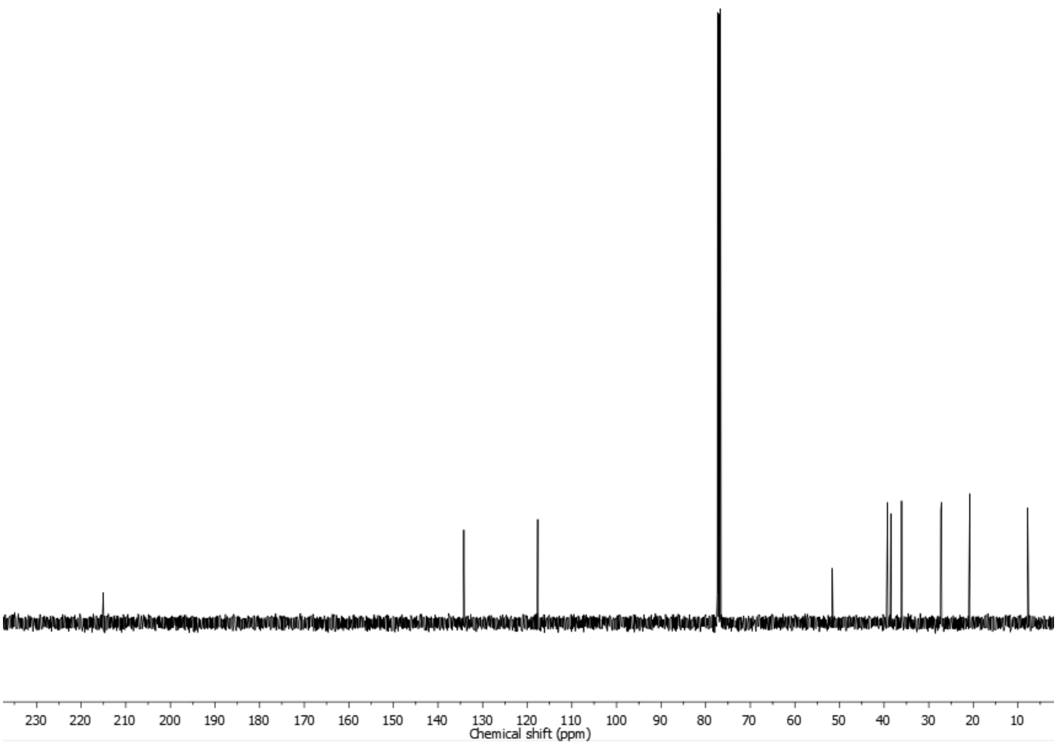

**2-Allyl-2-methylcyclopentan-1-one (3a)**

400 MHz, $CDCl_3$

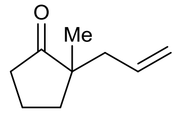


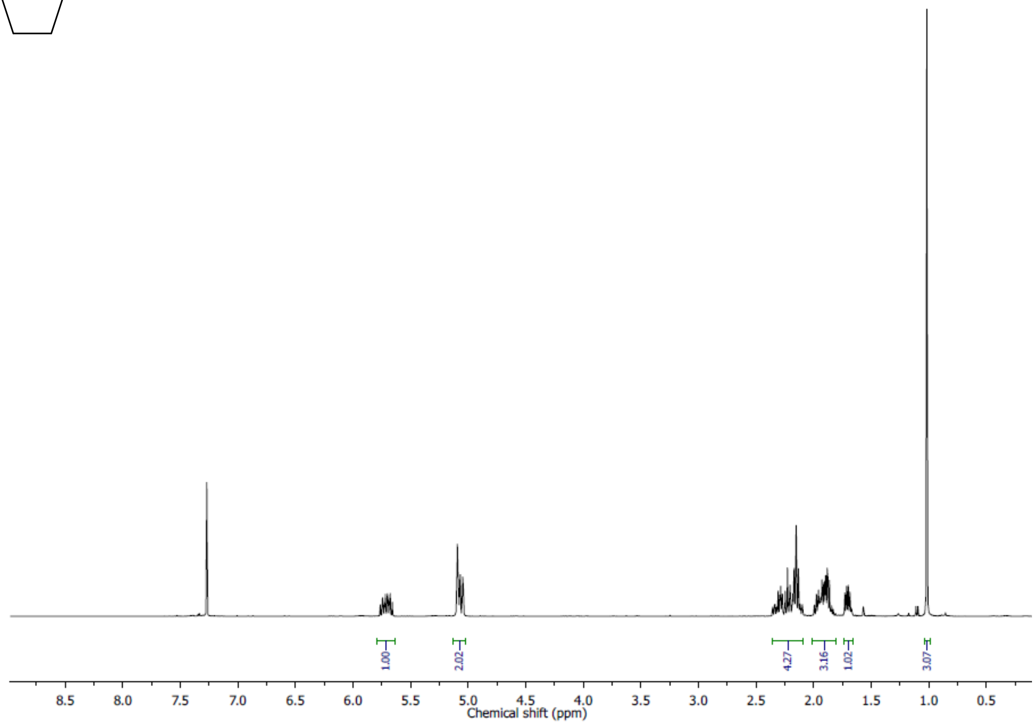


101 MHz, $CDCl_3$

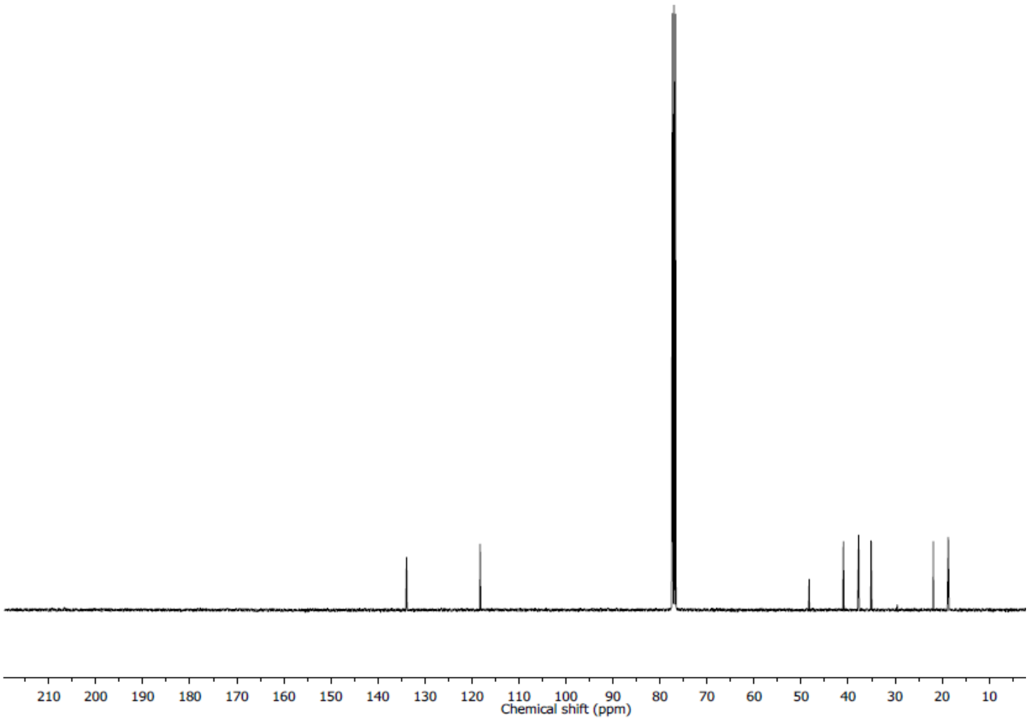

**(*E*)-2-Cinnamyl-2-methylcyclopentan-1-one (3b)**

400 MHz, $CDCl_3$

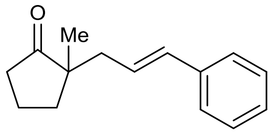


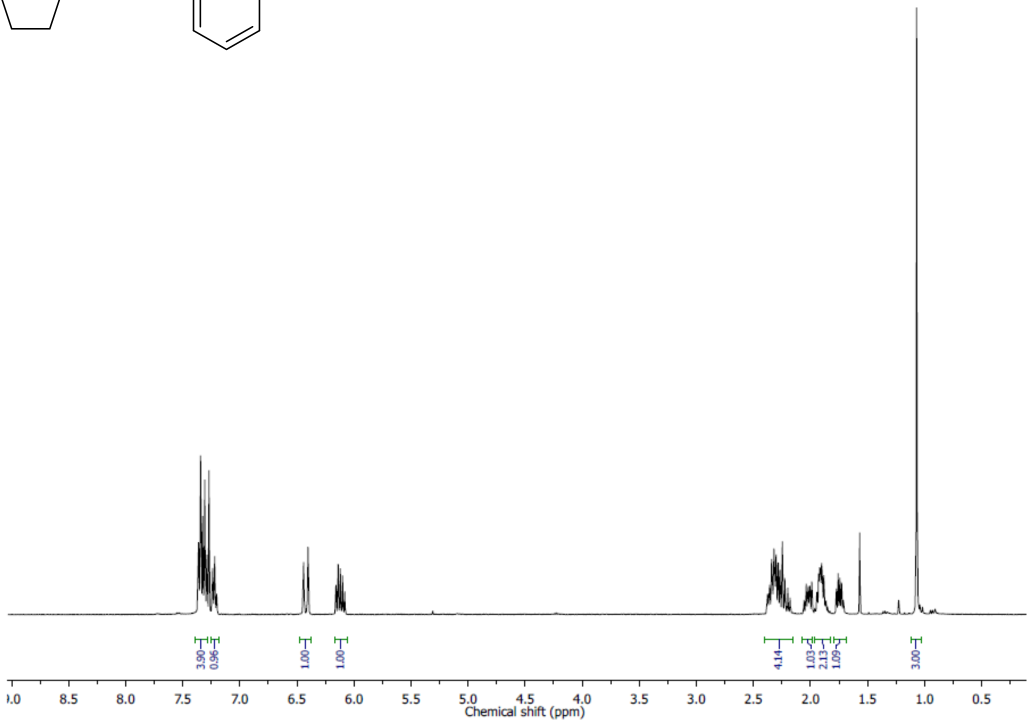


101 MHz, $CDCl_3$

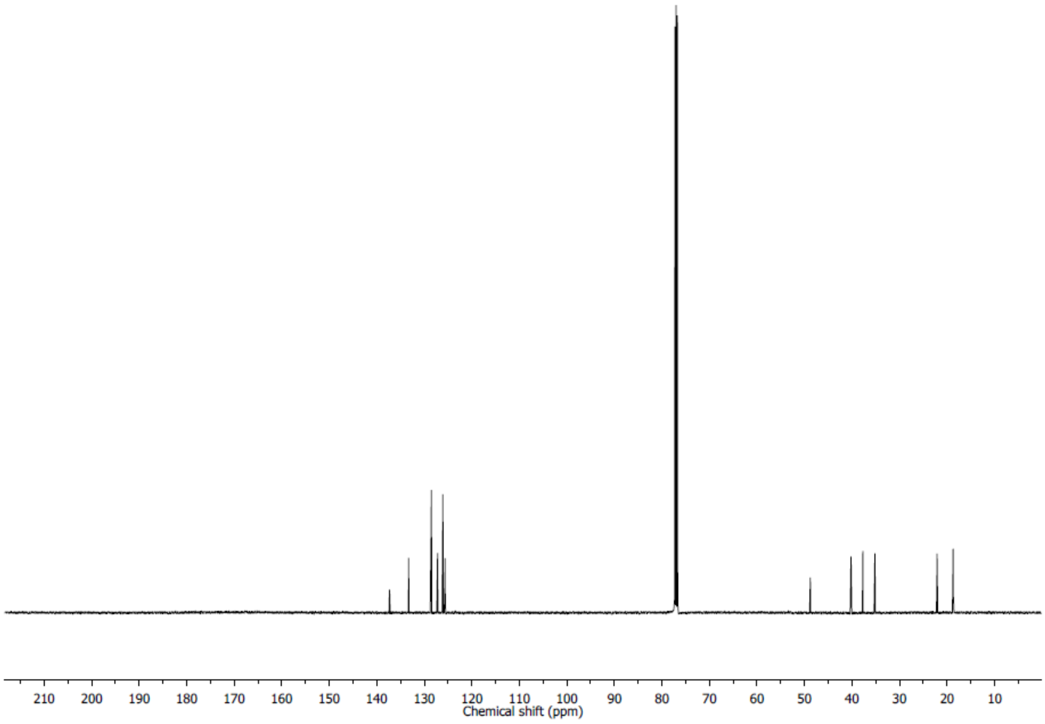

**(*E*)-2-(3-(4-Fluorophenyl)allyl)-2-methylcyclopentan-1-one (3c)**

400 MHz, $CDCl_3$

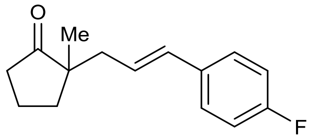


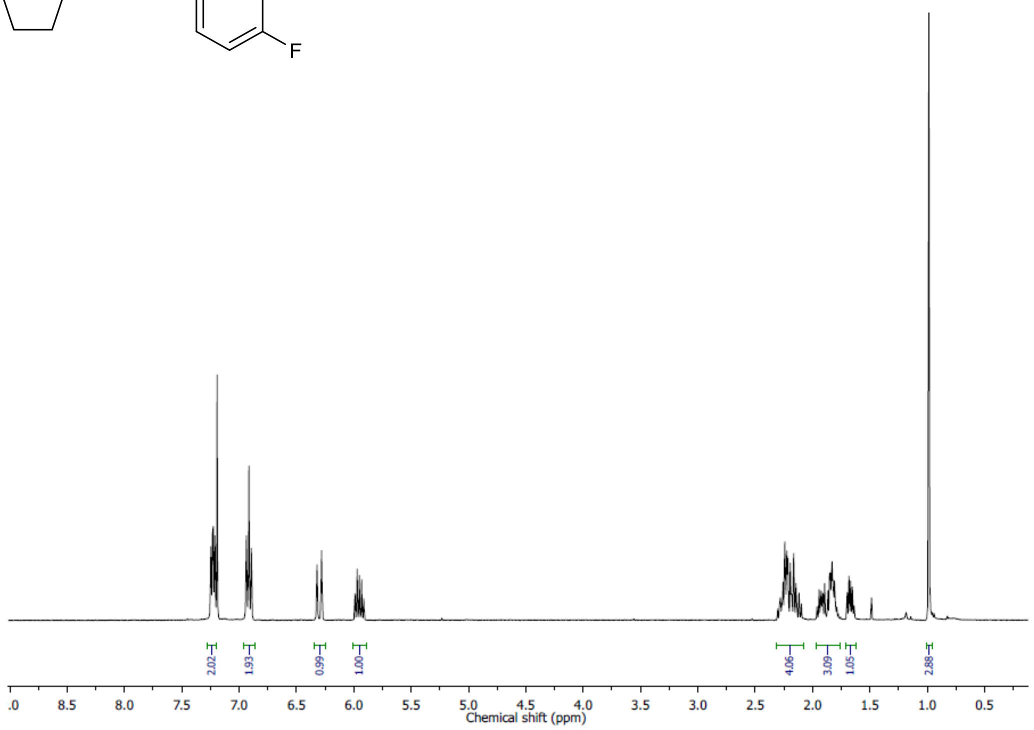


101 MHz, $CDCl_3$

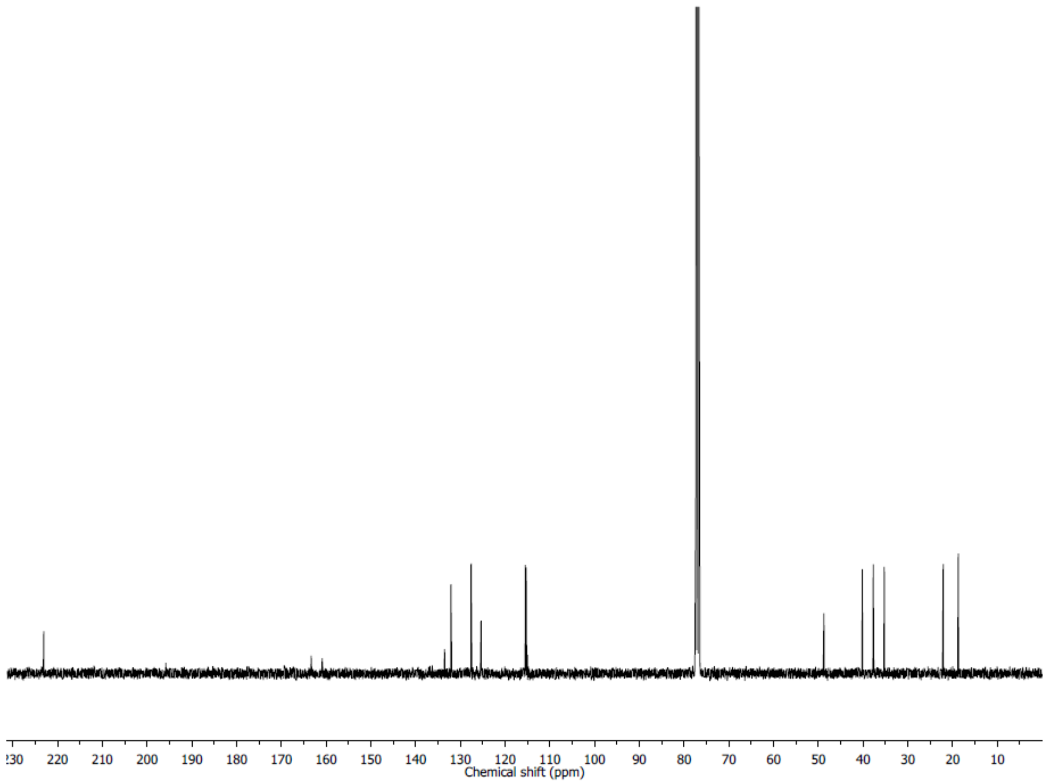

**(*E*)-2-Methyl-2-(3-(naphthalen-2-yl)allyl)cyclopentan-1-one (3d)**

400 MHz, $CDCl_3$

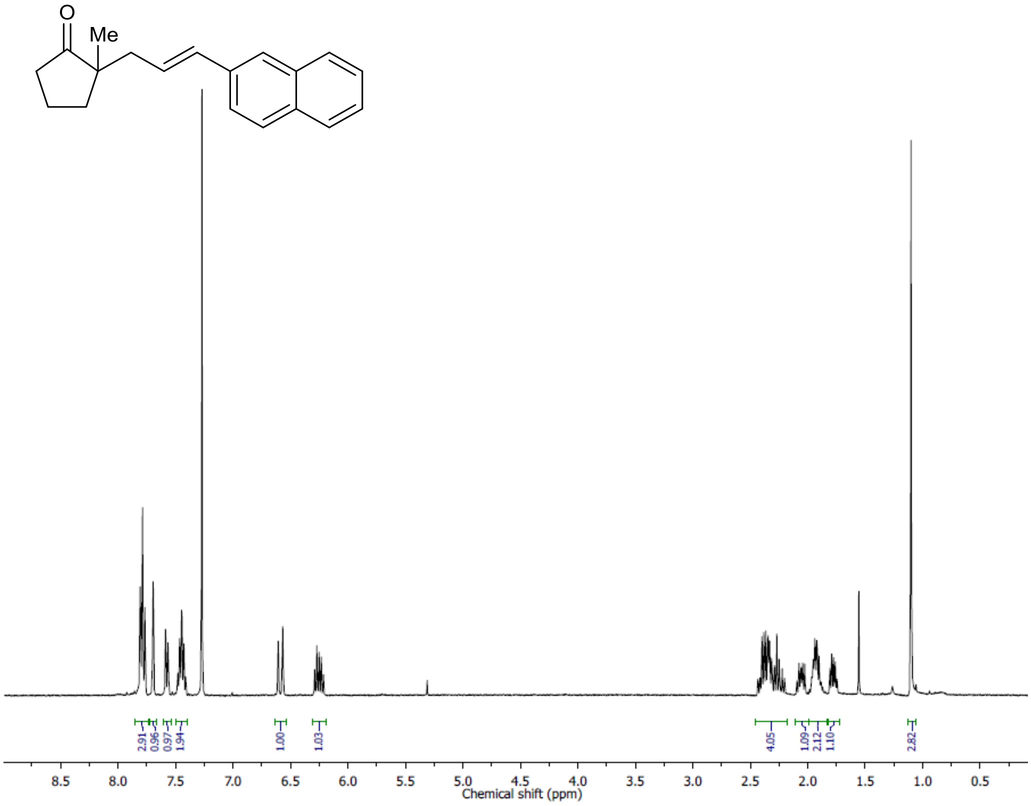


101 MHz, $CDCl_3$

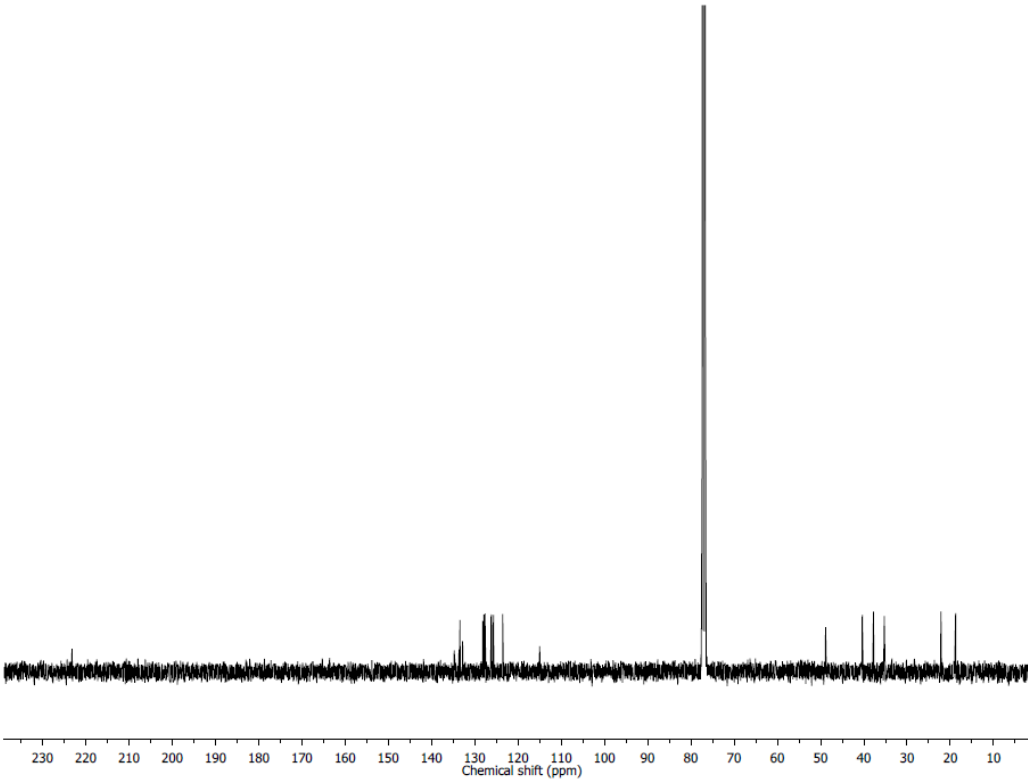

**(*E*)-2-(3-(3-Bromophenyl)allyl)-2-methylcyclopentan-1-one (3e)**

400 MHz, $CDCl_3$

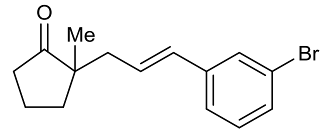


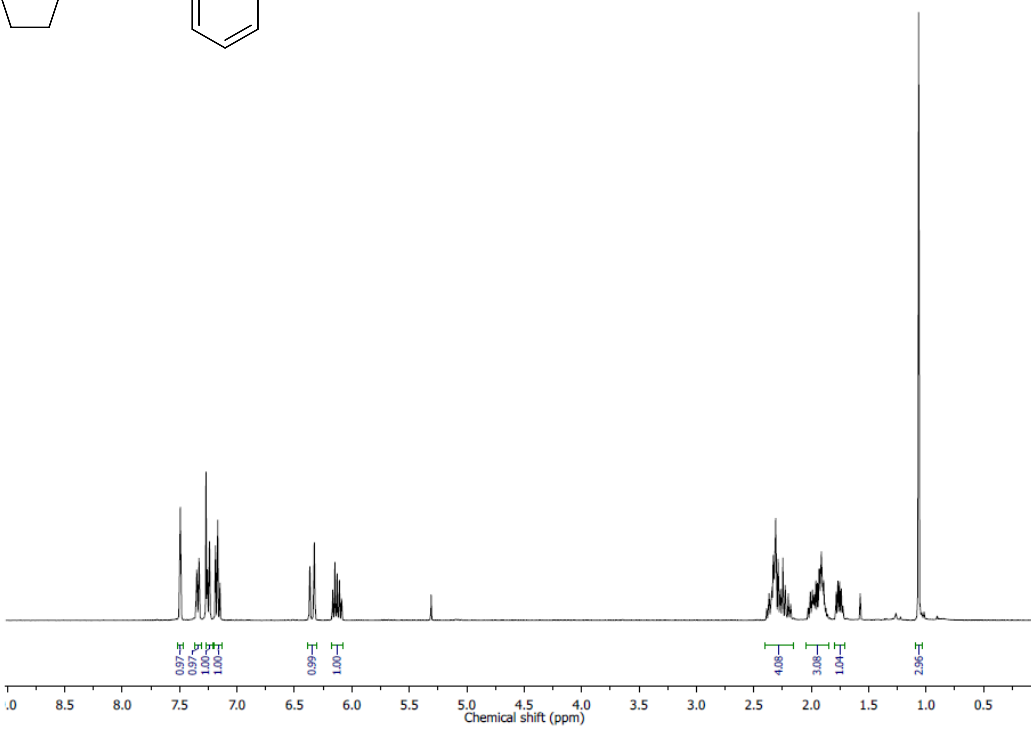


101 MHz, $CDCl_3$

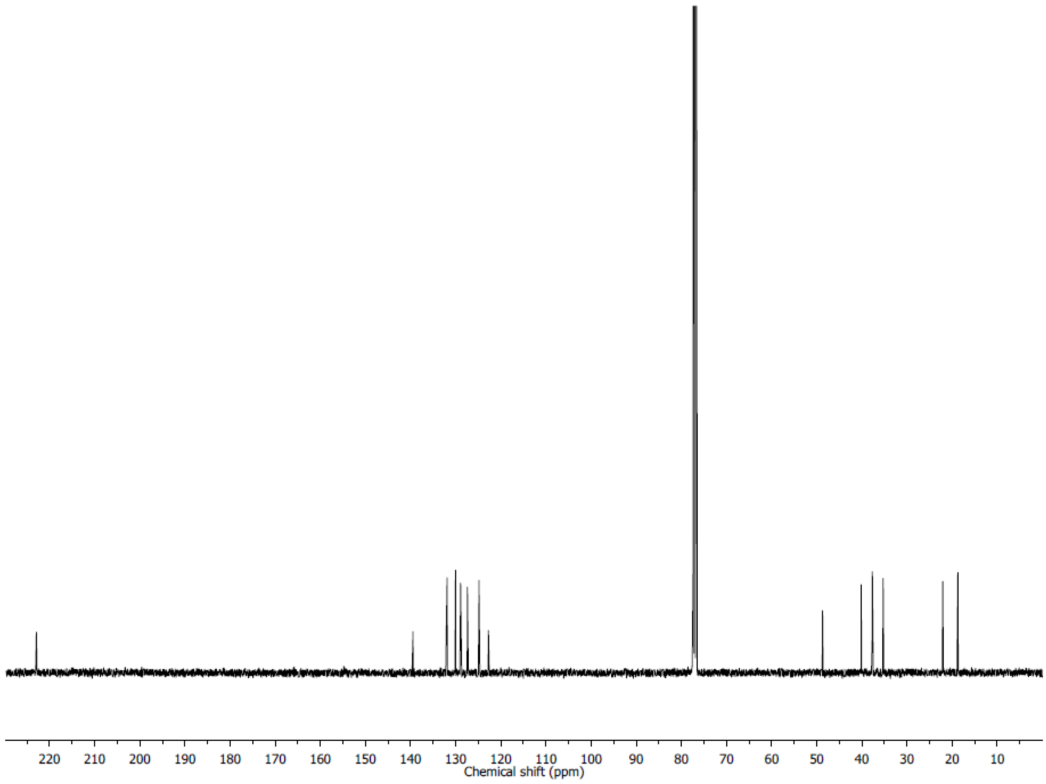

**(*E*)-2-(3-(2-Chlorophenyl)allyl)-2-methylcyclopentan-1-one (3f)**

400 MHz, $CDCl_3$

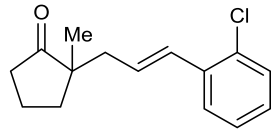


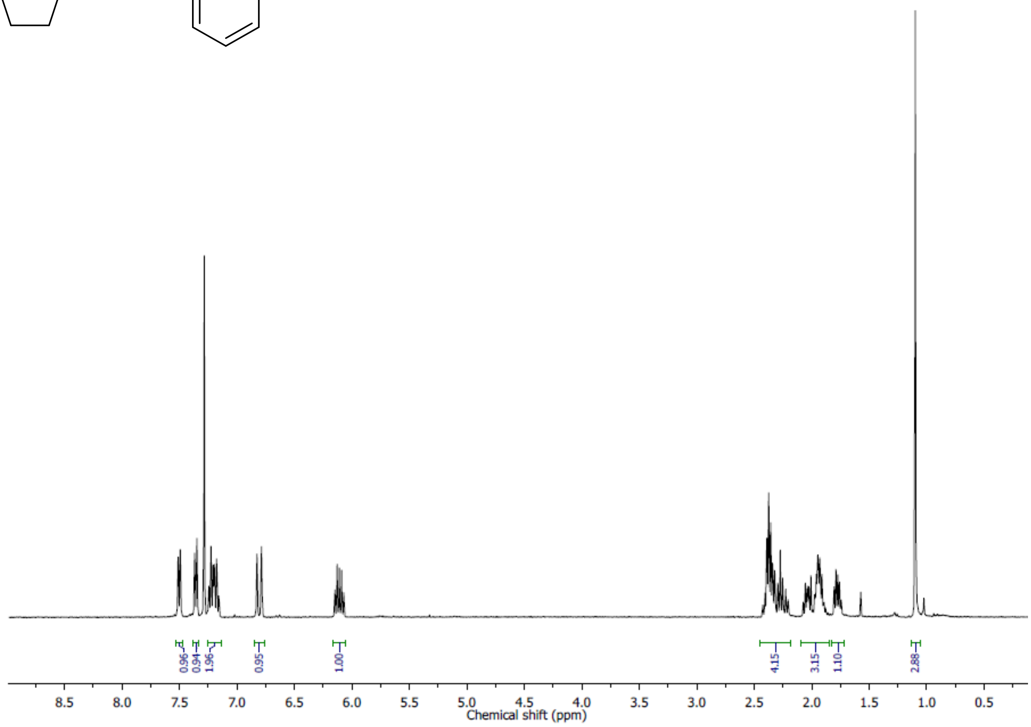


101 MHz, $CDCl_3$

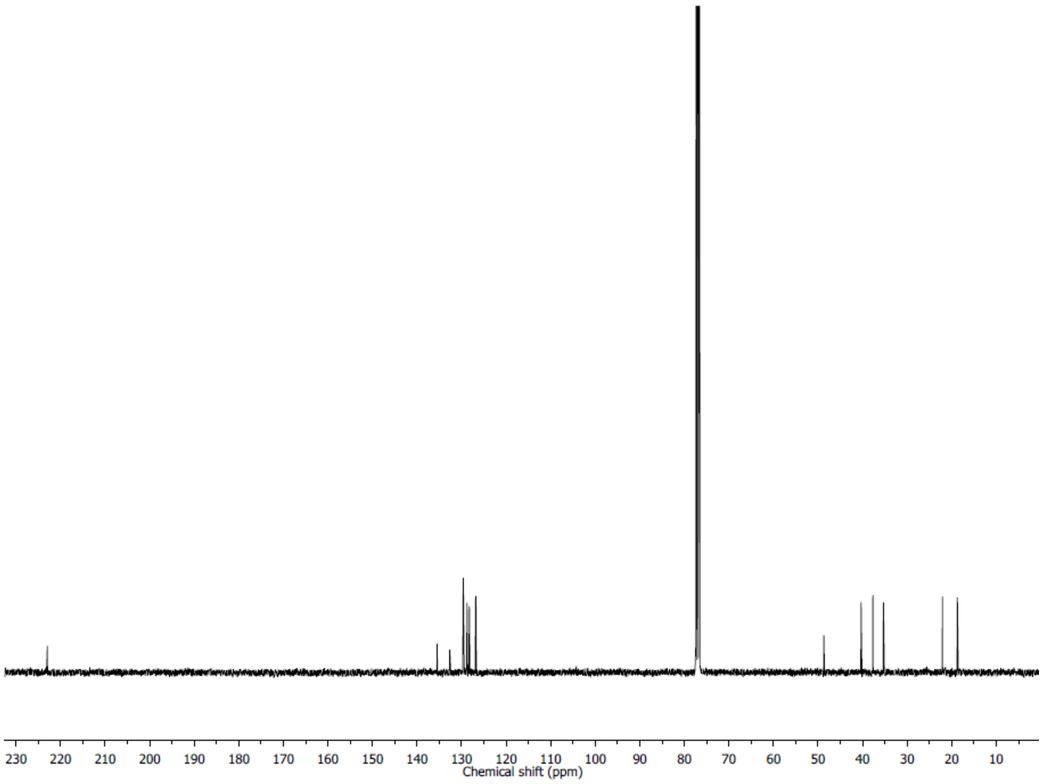

**(*E*)-2-Methyl-2-(3-(4-(trifluoromethyl)phenyl)allyl)cyclopentan-1-one (3g)**

400 MHz, $CDCl_3$

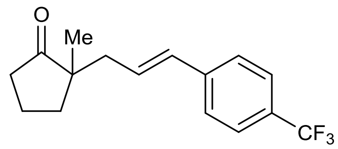


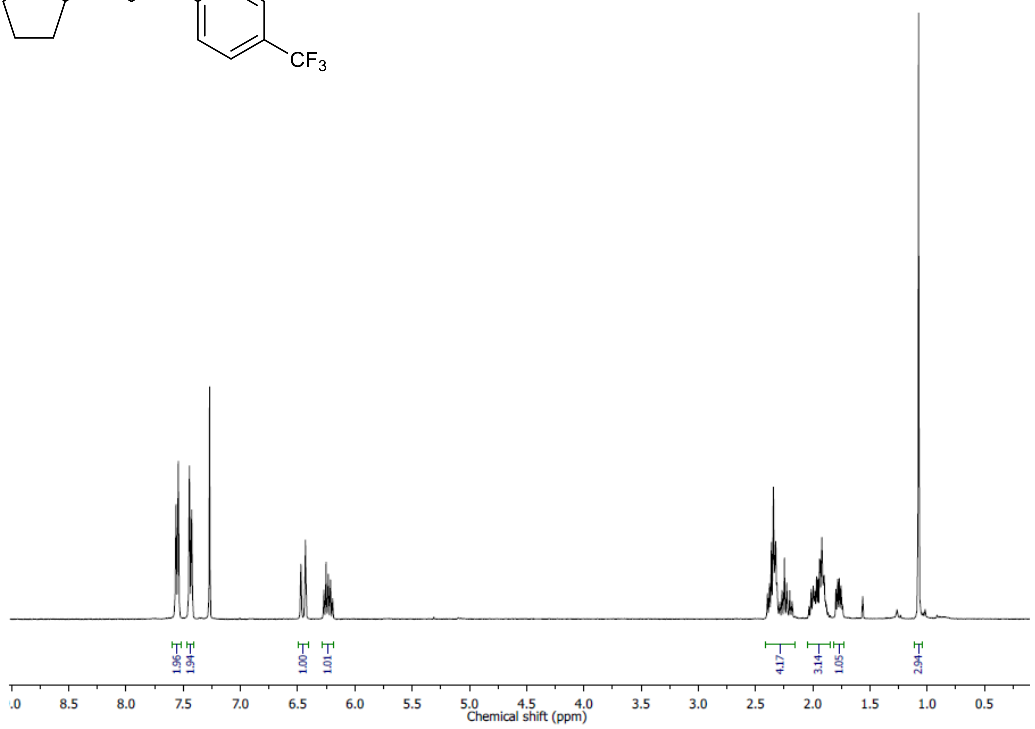


101 MHz, $CDCl_3$

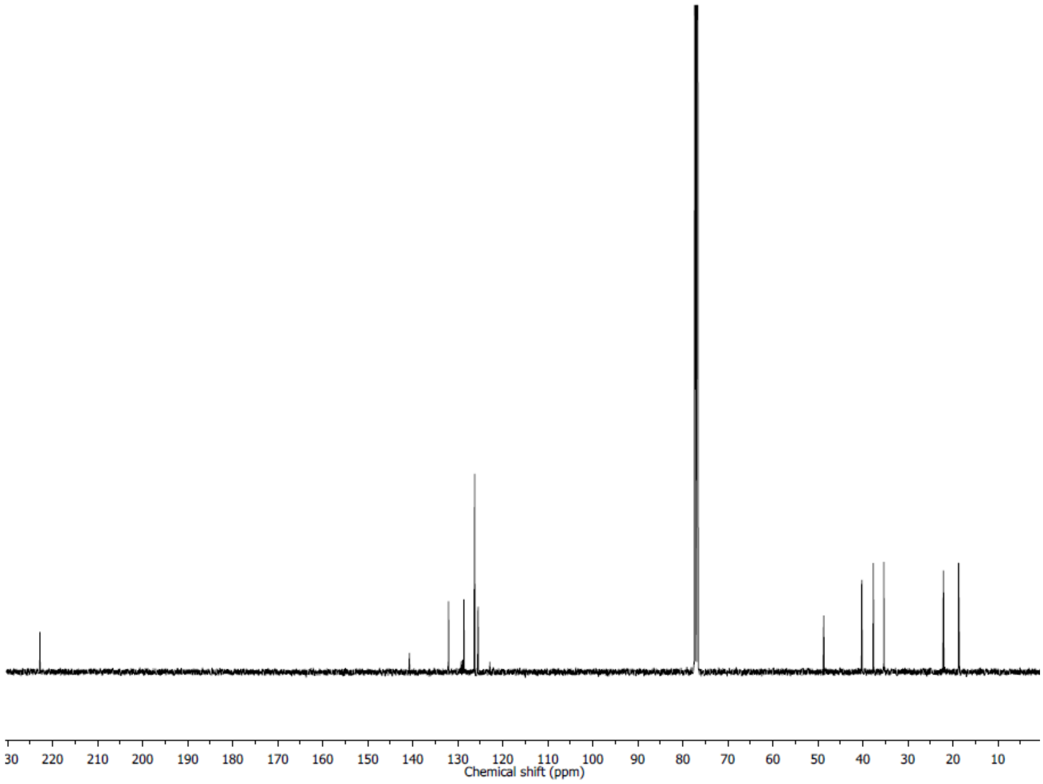

**(*E*)-2-Methyl-2-(3-(*o*-tolyl)allyl)cyclopentan-1-one (3h)**

400 MHz, $CDCl_3$

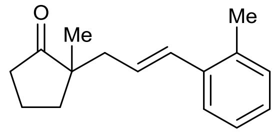


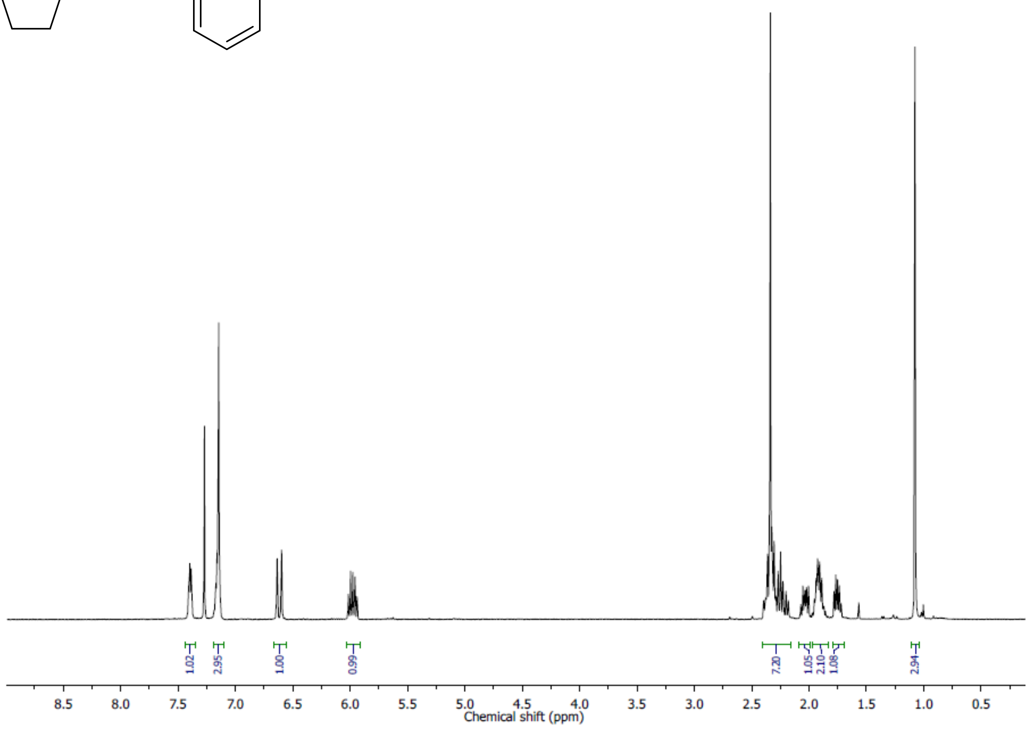


101 MHz, $CDCl_3$

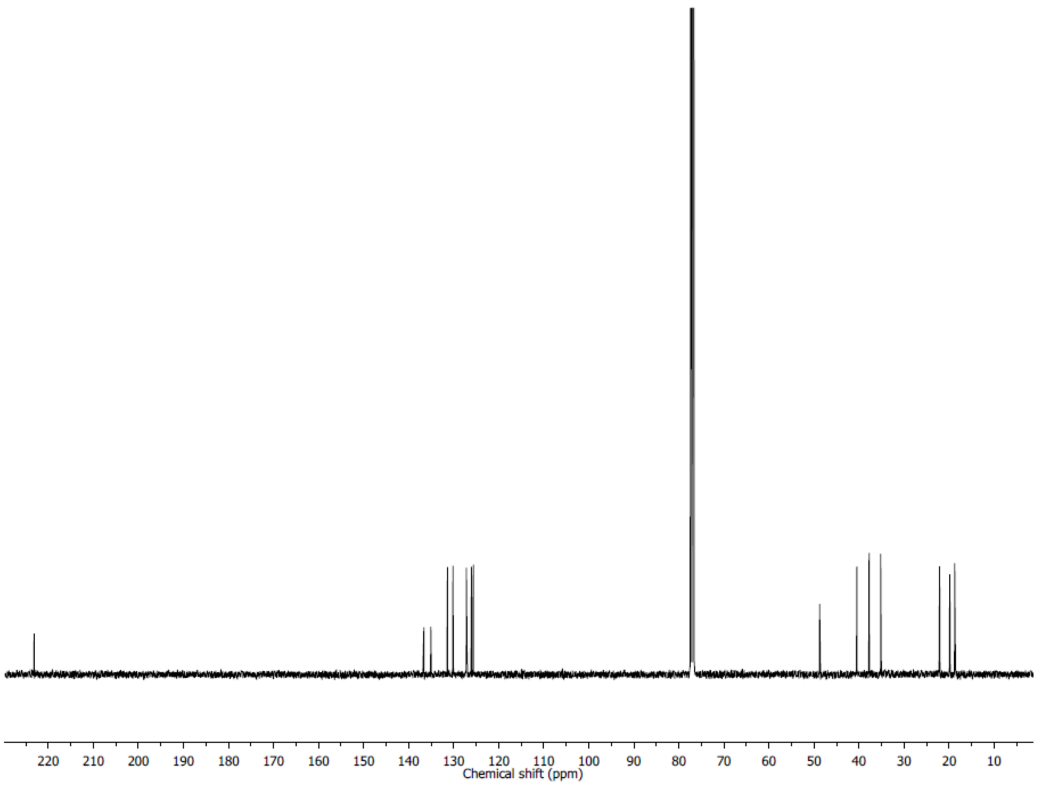

**(*R*)-7-Allyl-7-methyloxepan-2-one (2a)**

400 MHz, $CDCl_3$

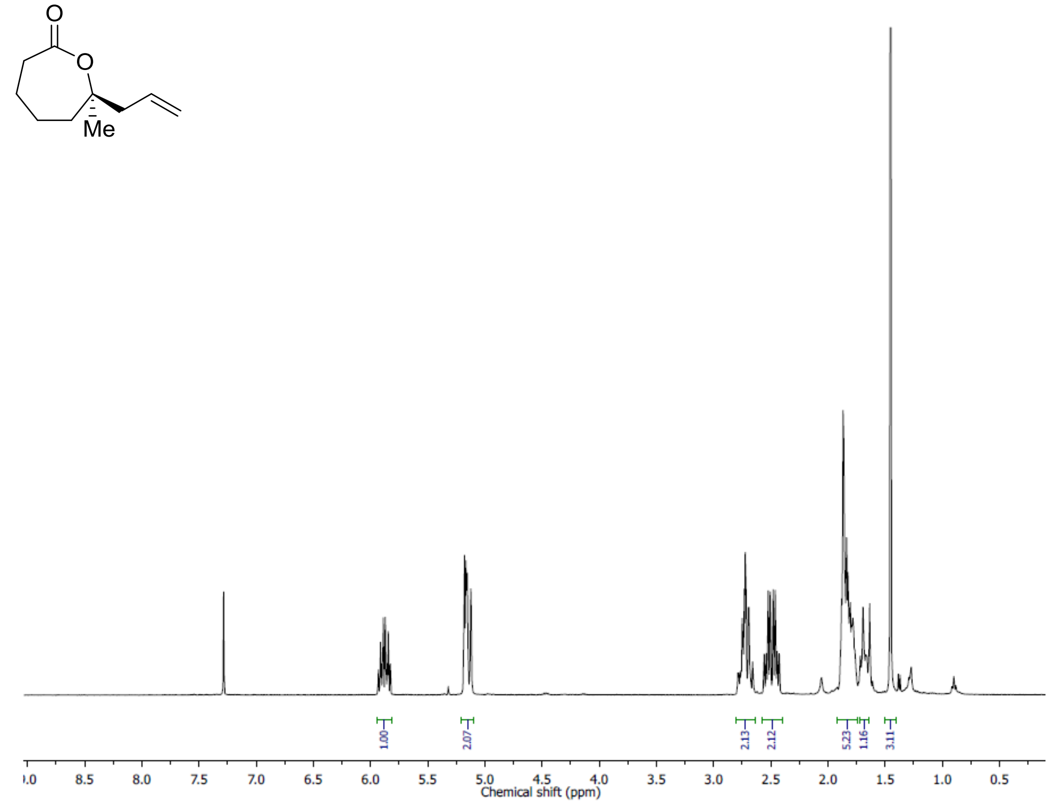


101 MHz, $CDCl_3$

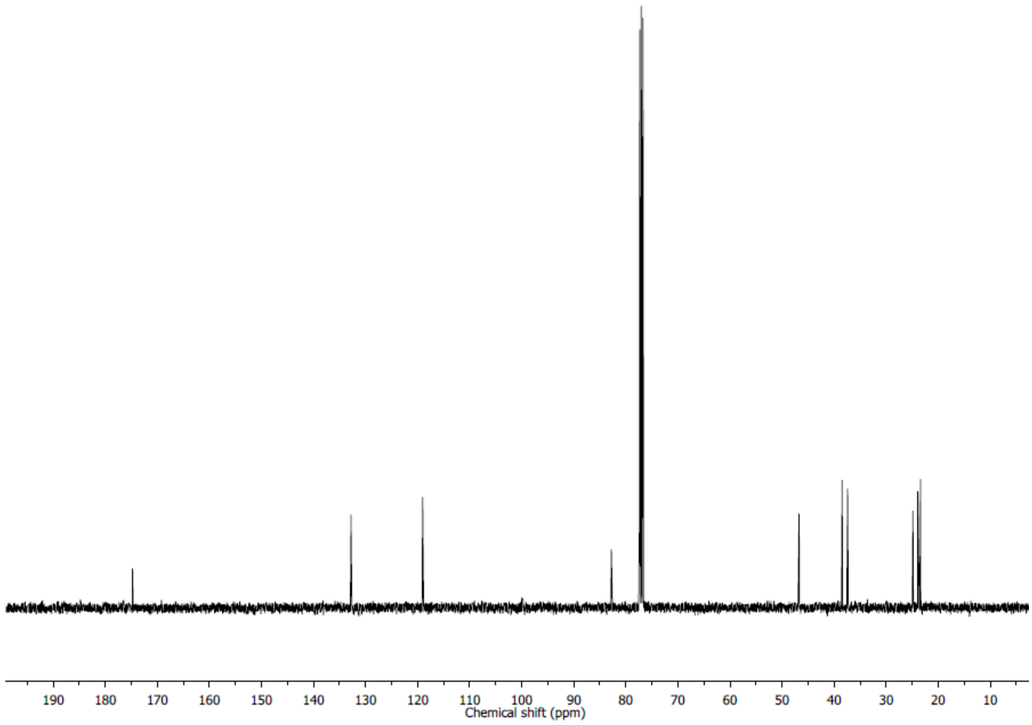

**(*R,E*)-7-Cinnamyl-7-methyloxepan-2-one (2b)**

400 MHz, $CDCl_3$

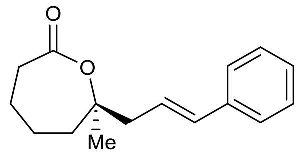


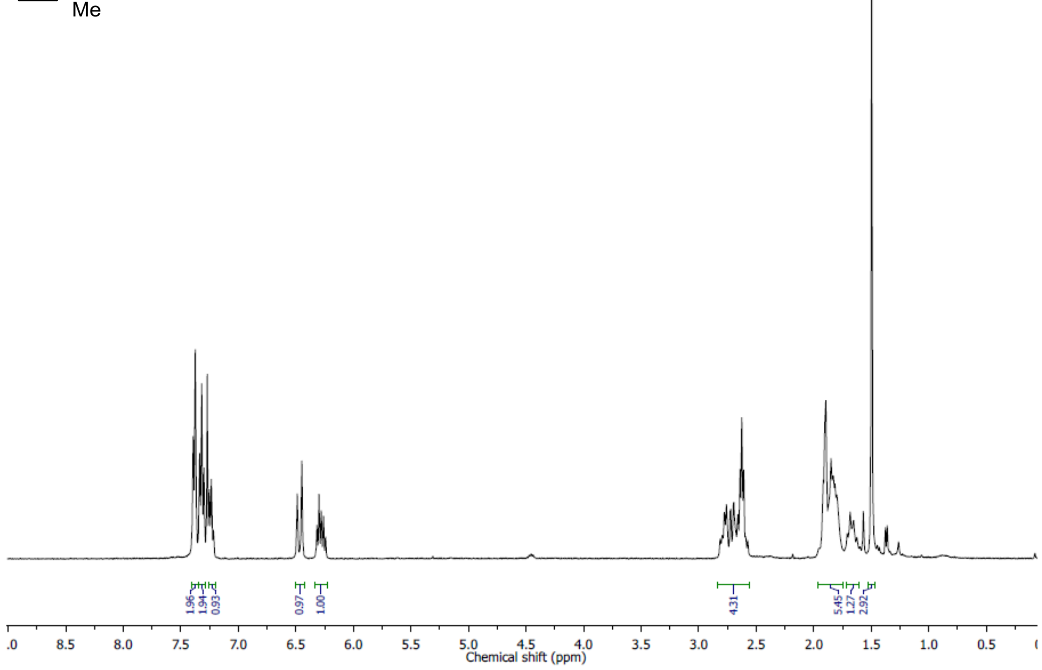


101 MHz, $CDCl_3$

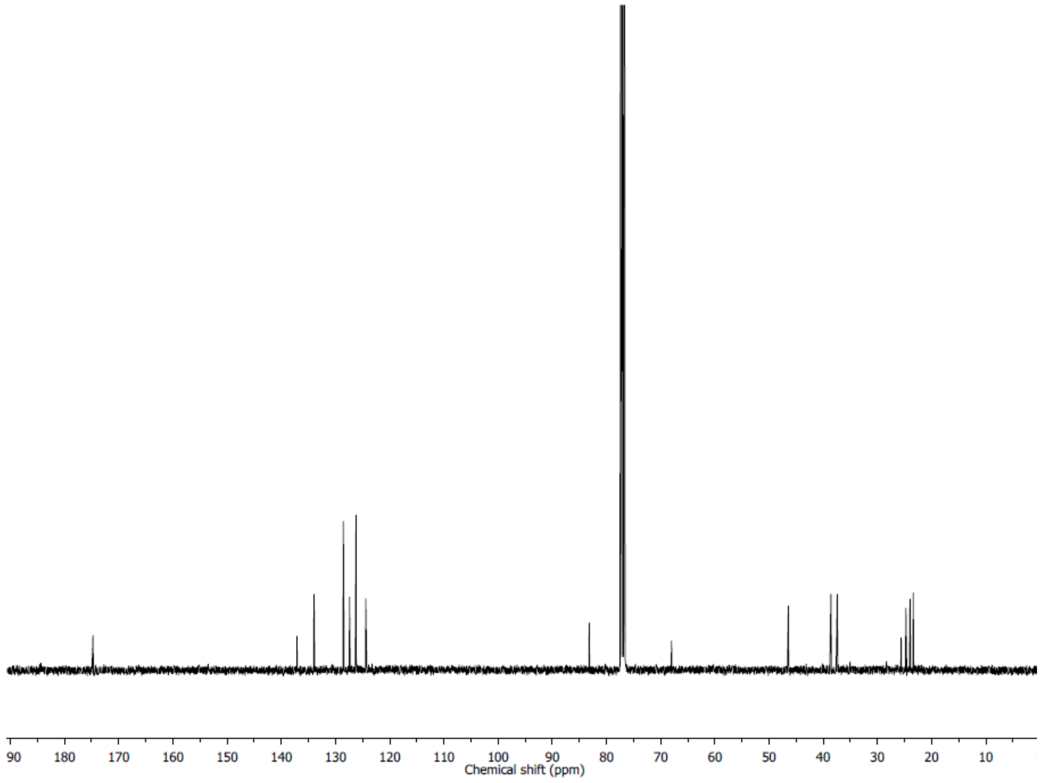

**(*R,E*)-7-(3-(4-Fluorophenyl)allyl)-7-methyloxepan-2-one (2c)**

400 MHz, $CDCl_3$

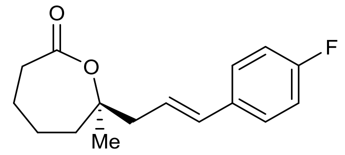


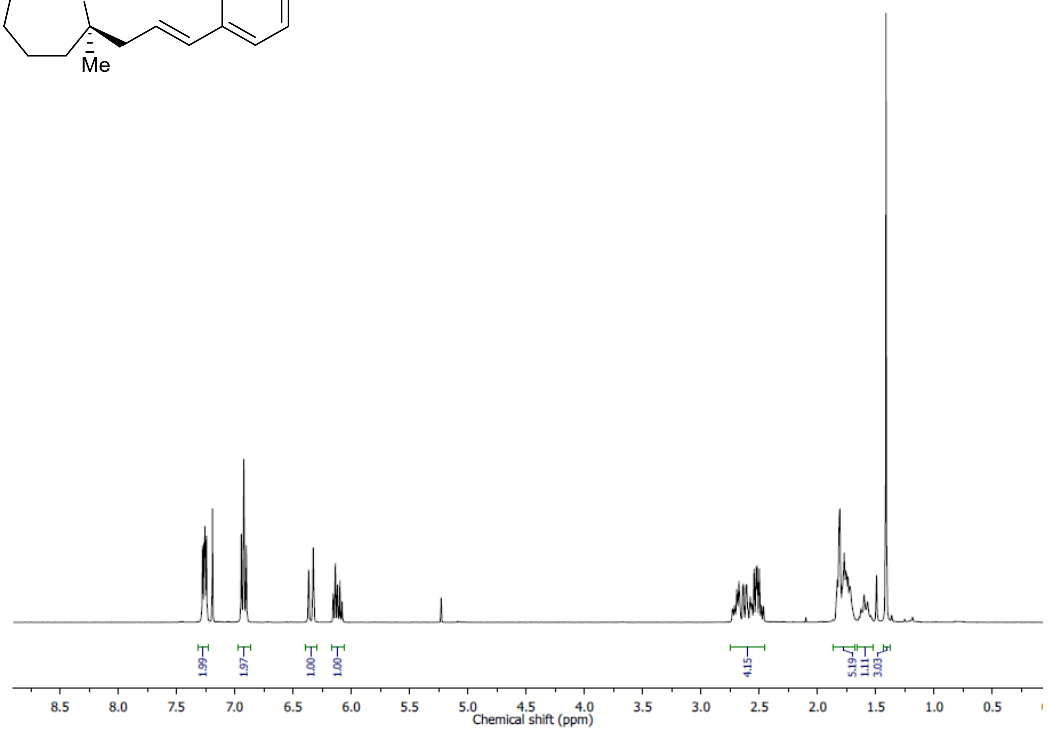


101 MHz, $CDCl_3$

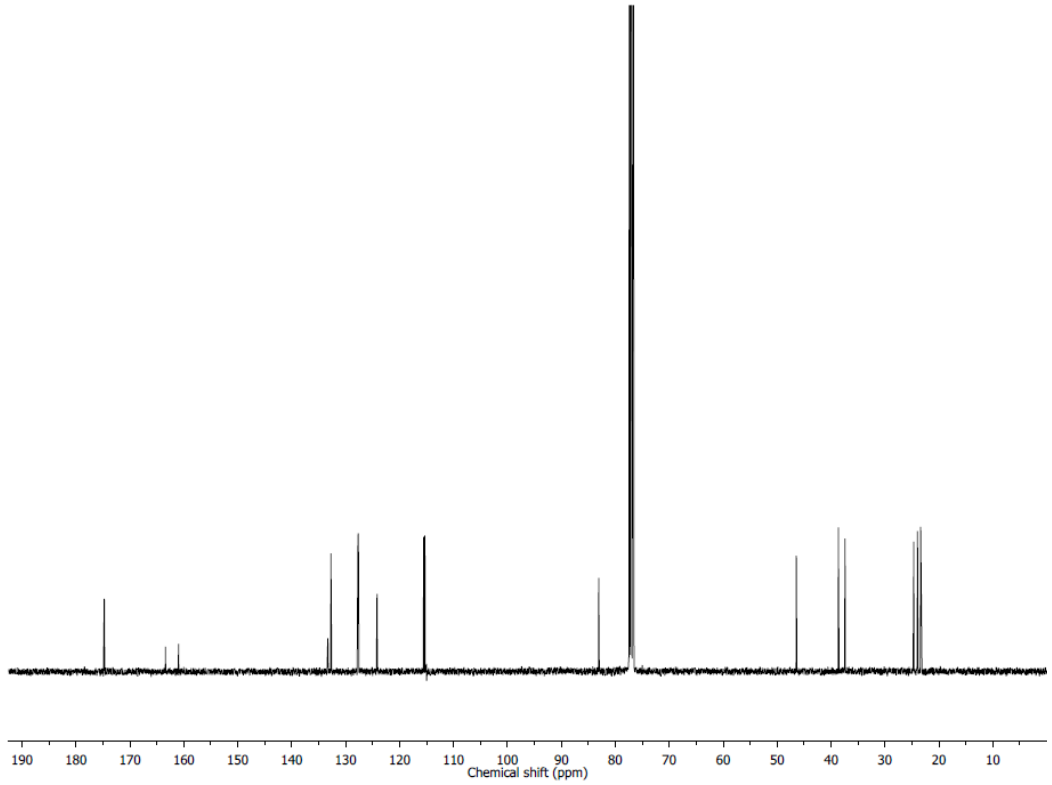

**(*R,E*)-7-Methyl-7-(3-(naphthalen-2-yl)allyl)oxepan-2-one (2d)**

400 MHz, $CDCl_3$

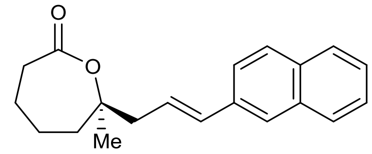


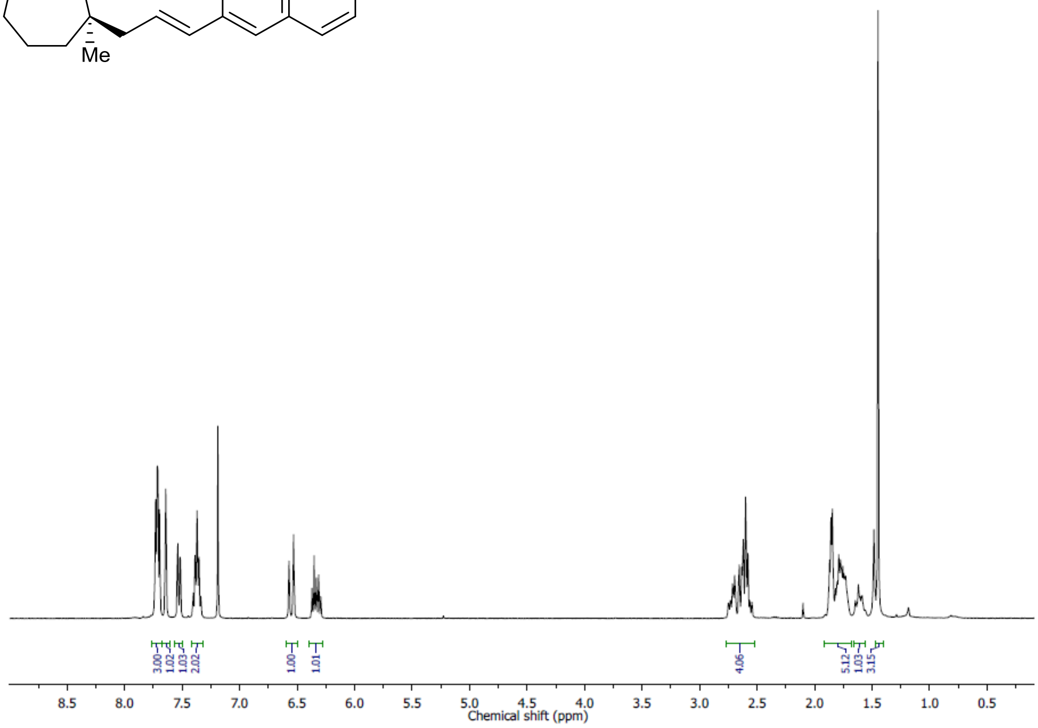


101 MHz, $CDCl_3$

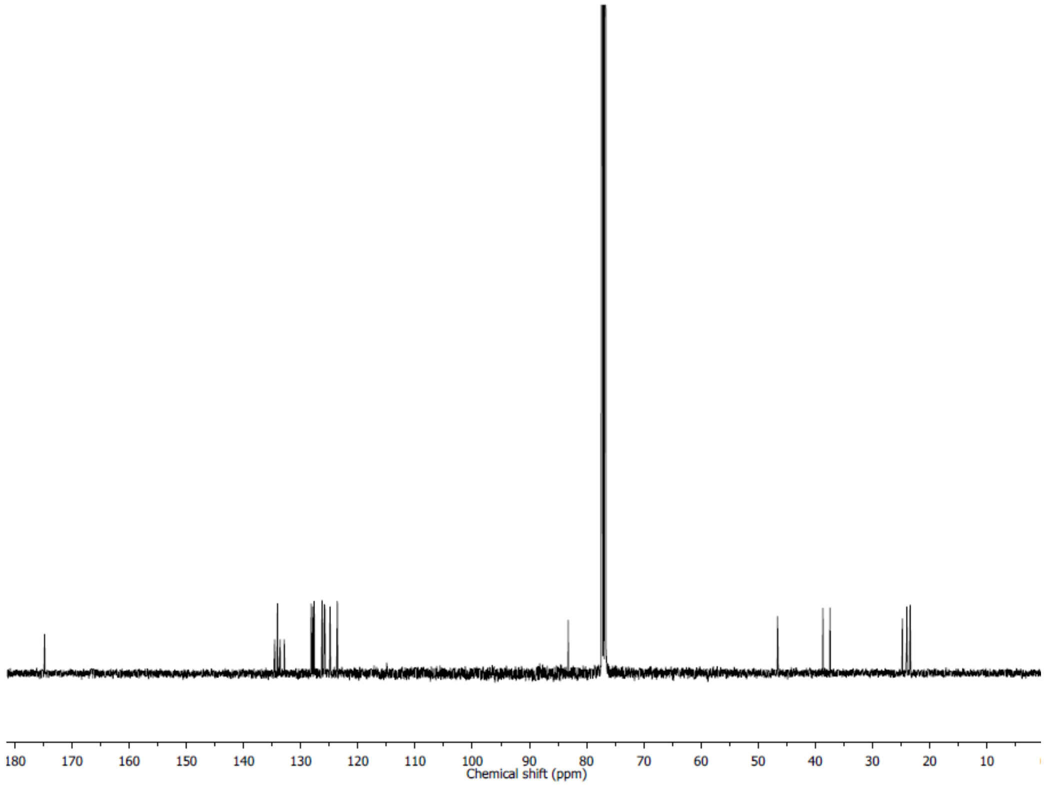

**(*R,E*)-7-(3-(3-Bromophenyl)allyl)-7-methyloxepan-2-one (2e)**

400 MHz, $CDCl_3$

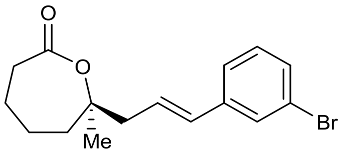


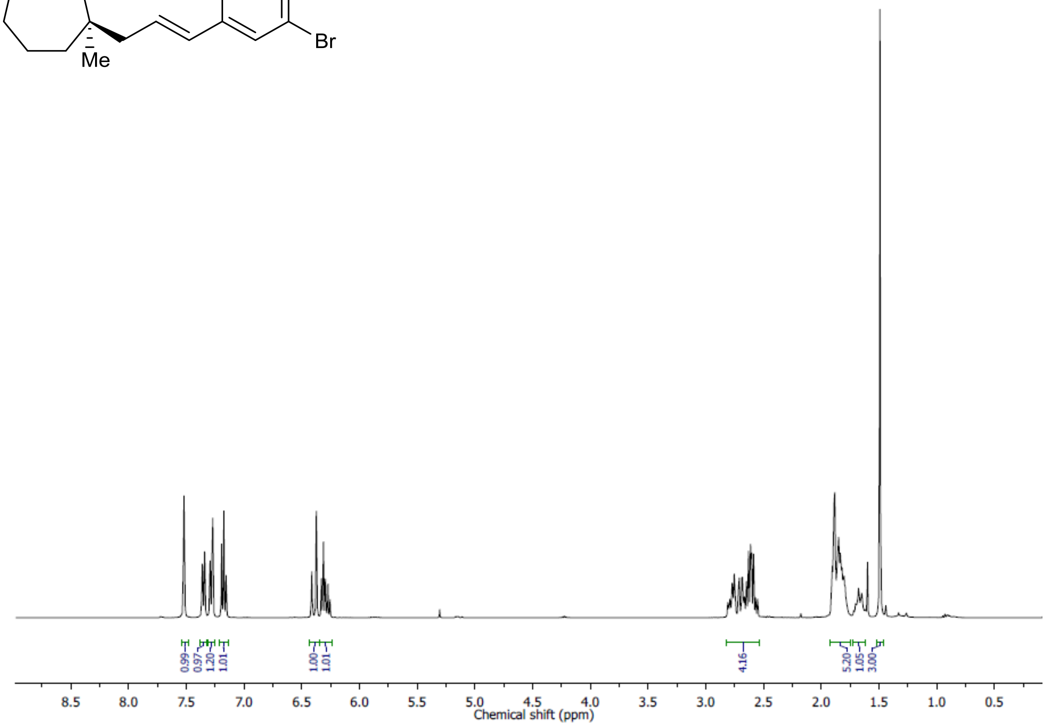


101 MHz, $CDCl_3$

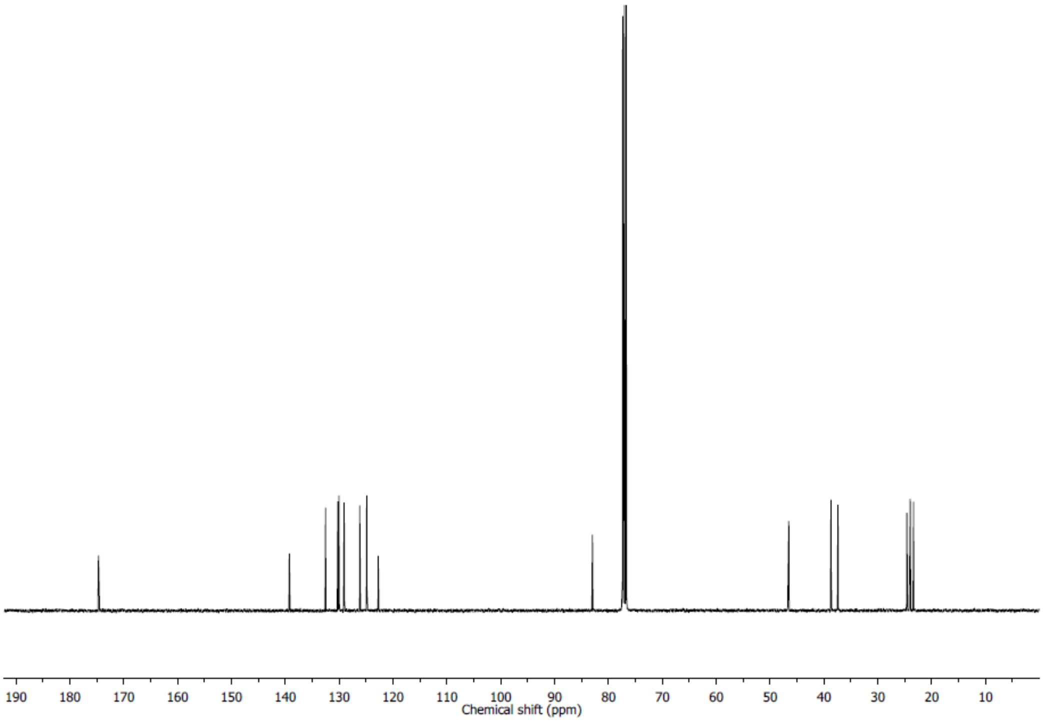

**(*R,E*)-7-(3-(2-Chlorophenyl)allyl)-7-methyloxepan-2-one (2f)**

400 MHz, $CDCl_3$

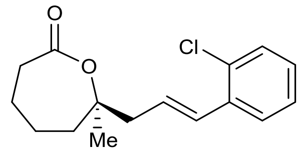


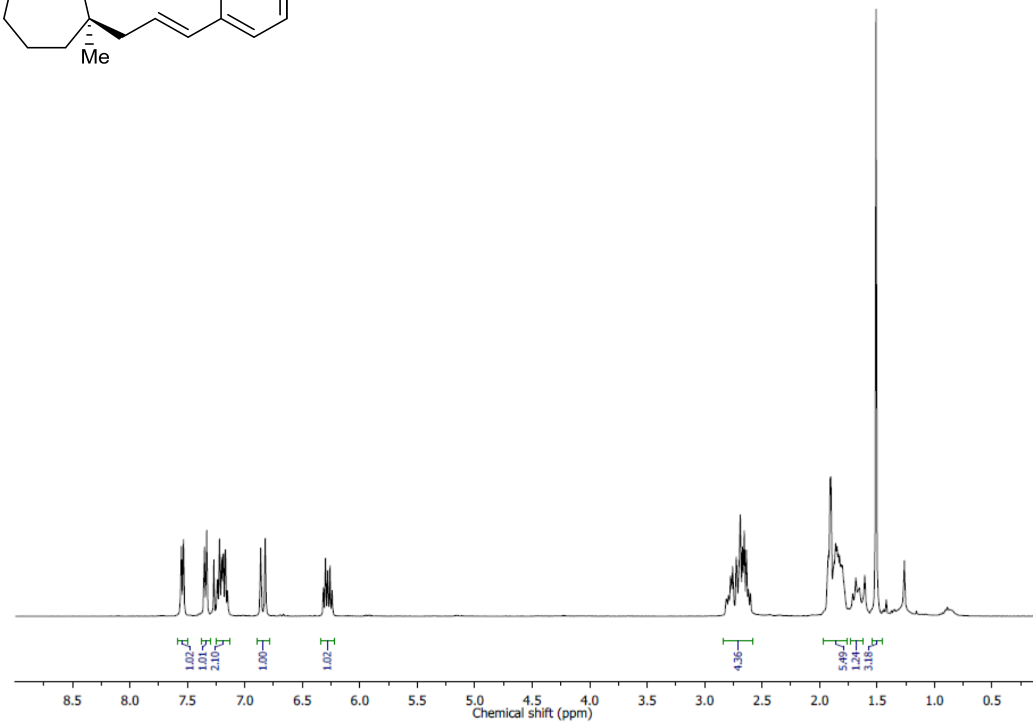


101 MHz, $CDCl_3$

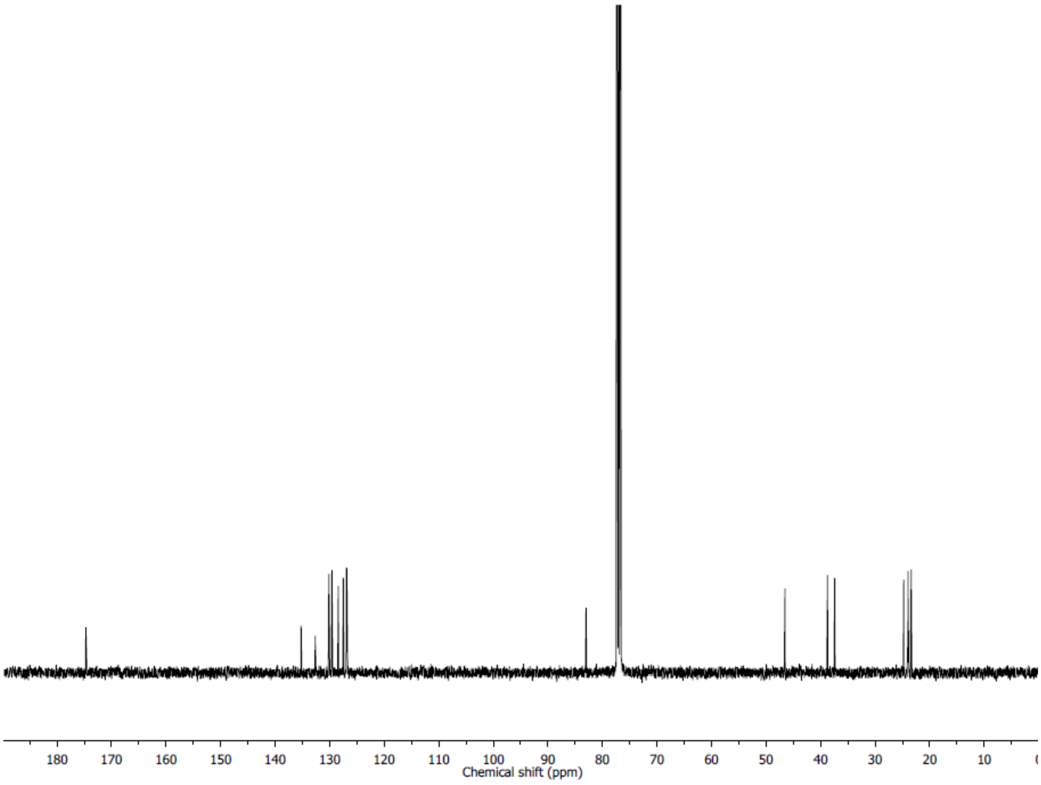

**(*R,E*)-7-Methyl-7-(3-(4-(trifluoromethyl)phenyl)allyl)oxepan-2-one (2g)**

400 MHz, $CDCl_3$

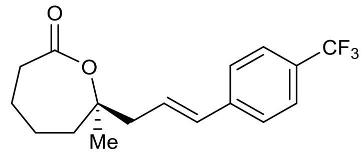


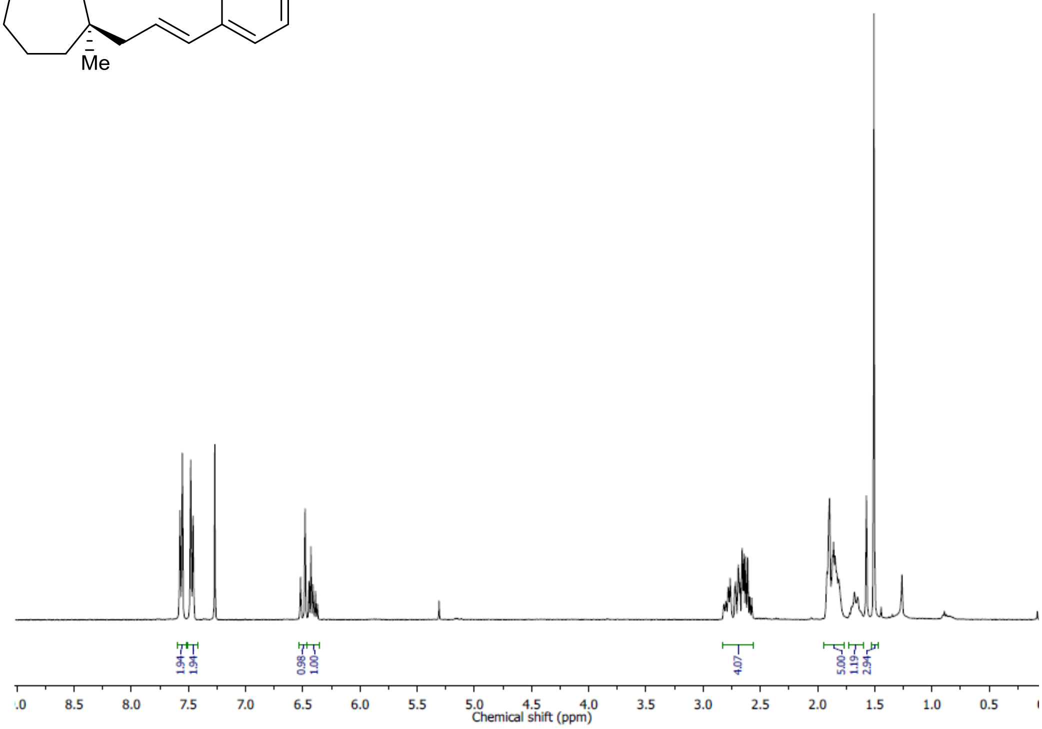


101 MHz, $CDCl_3$

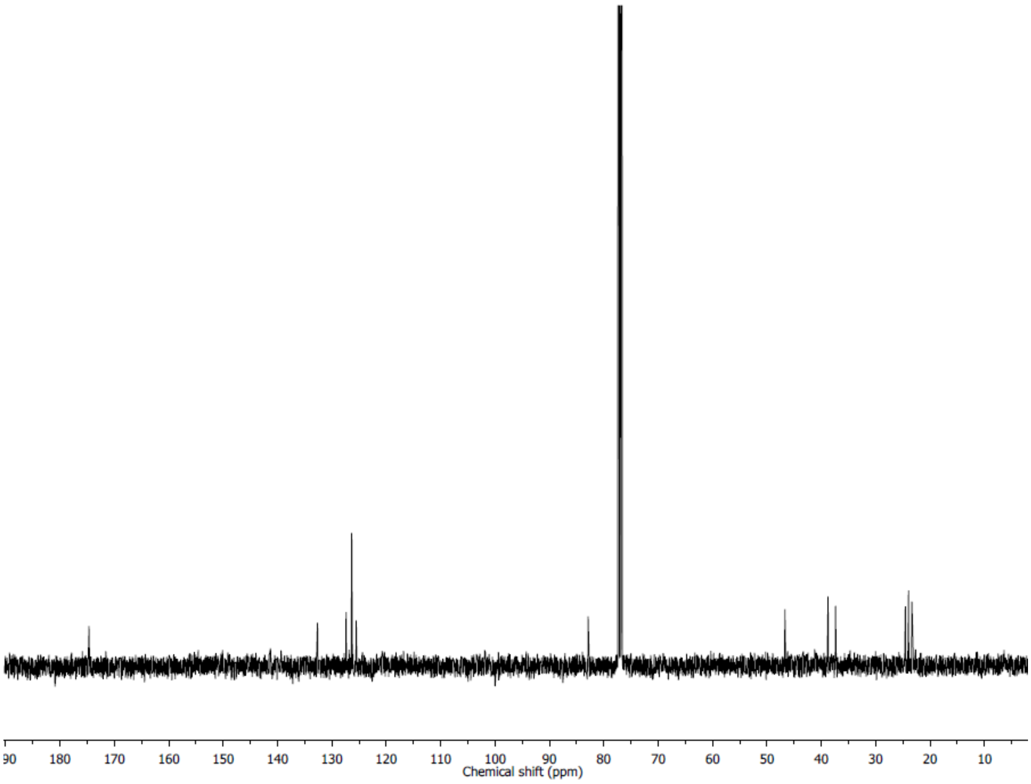

**(*R,E*)-7-Methyl-7-(3-(o-tolyl)allyl)oxepan-2-one (2h)**

400 MHz, $CDCl_3$

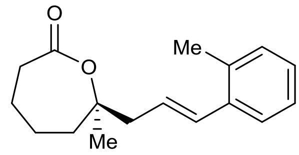


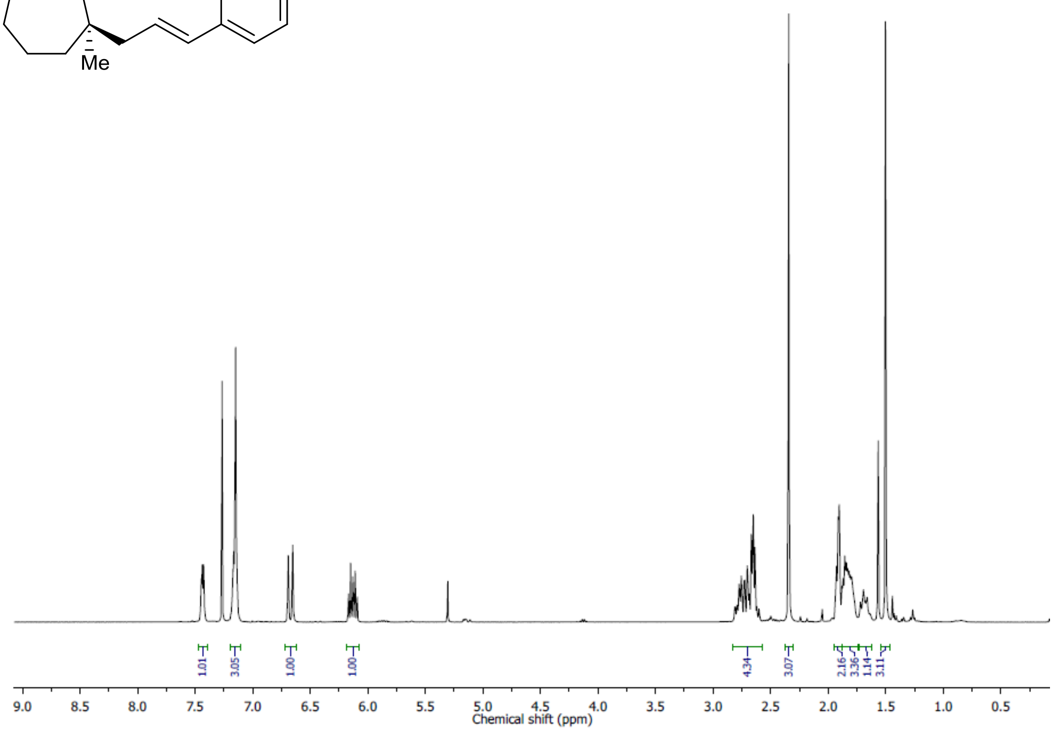


101 MHz, $CDCl_3$

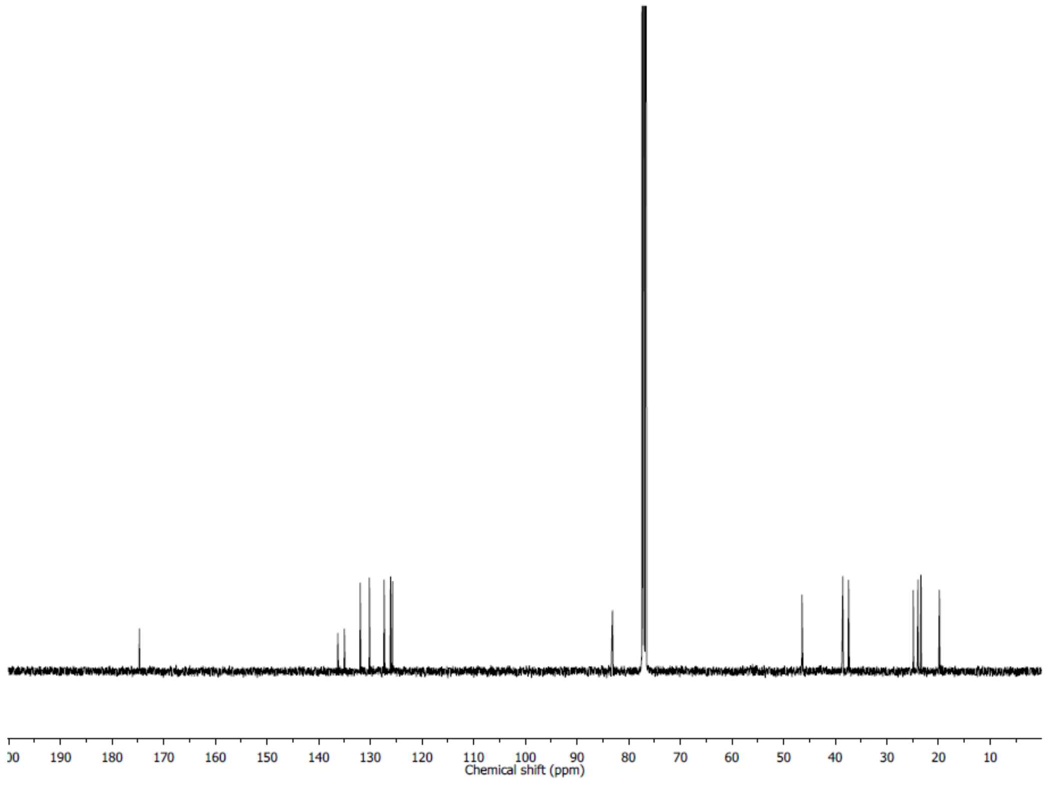

**(R)-7-Allyl-7-ethyloxepan-2-one (2i)**

400 MHz, $CDCl_3$

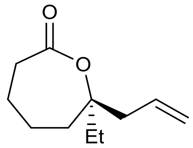


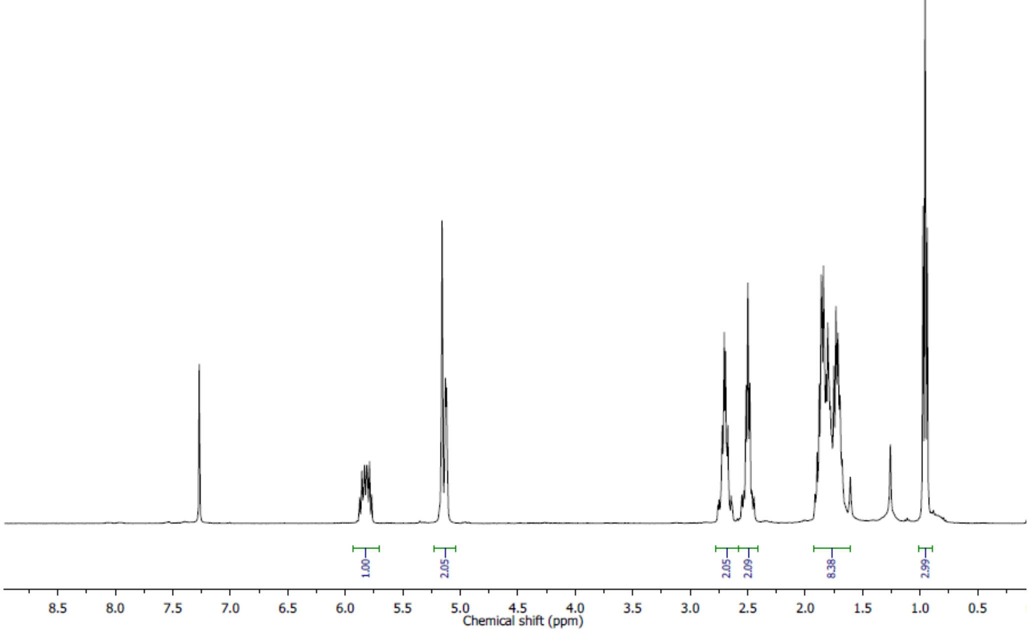


101 MHz, $CDCl_3$

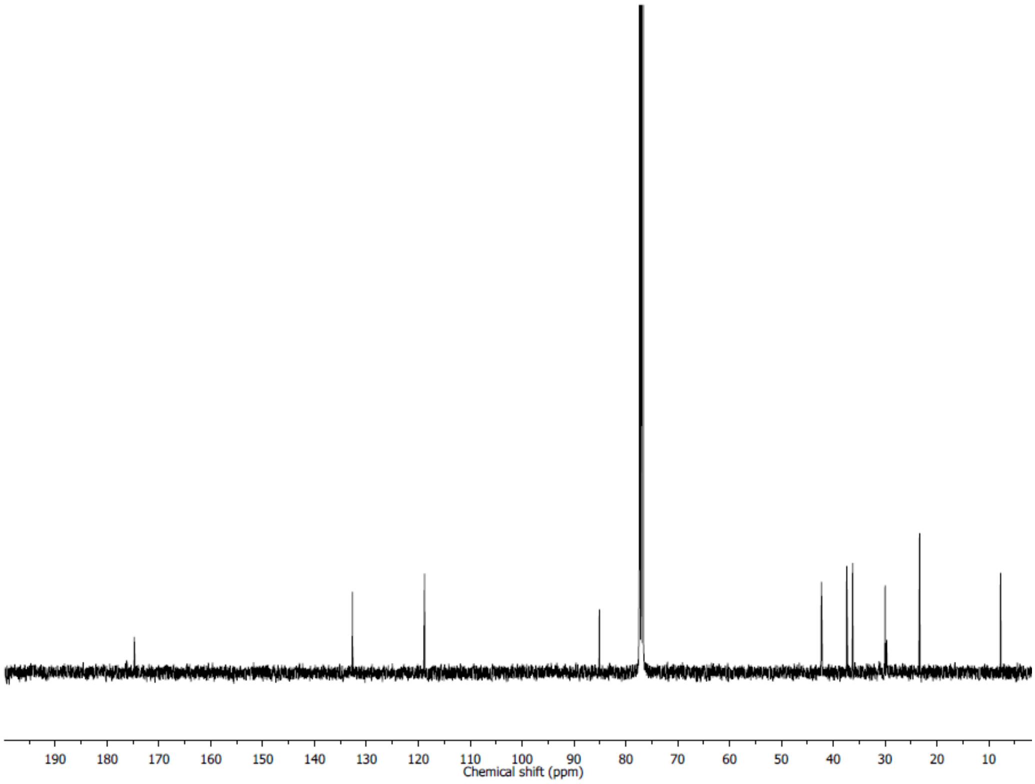

**(*R*)-6-Allyl-6-methyltetrahydro-2H-pyran-2-one (4a)**

400 MHz, $CDCl_3$

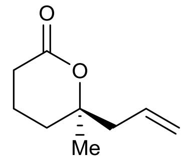


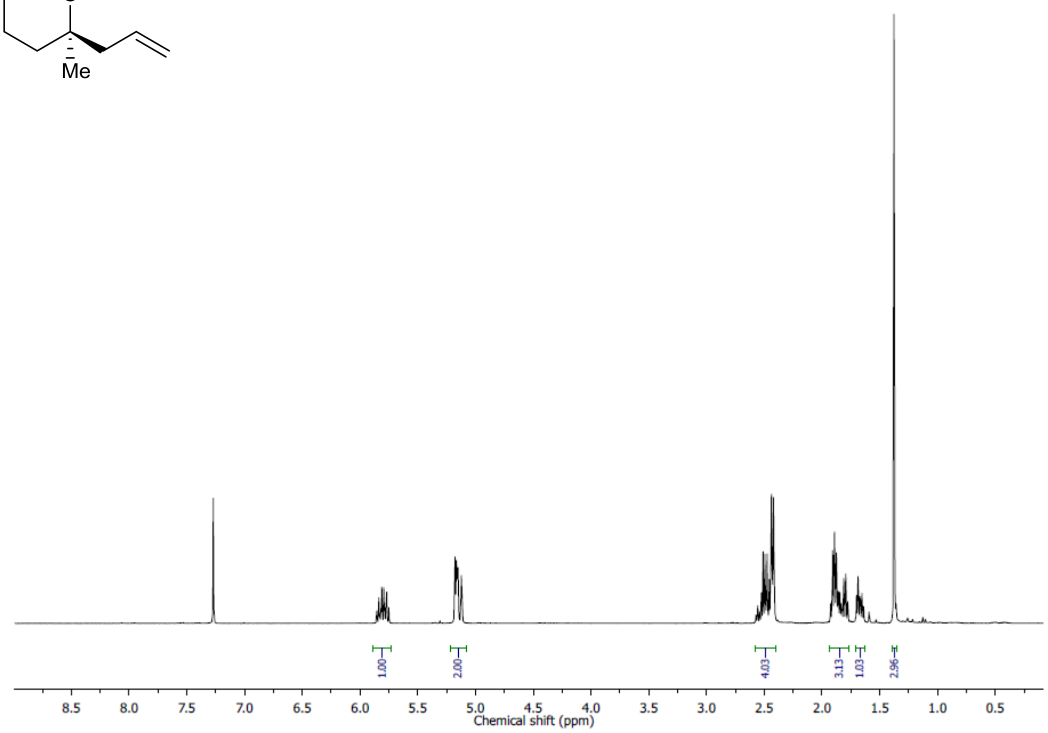


101 MHz, $CDCl_3$

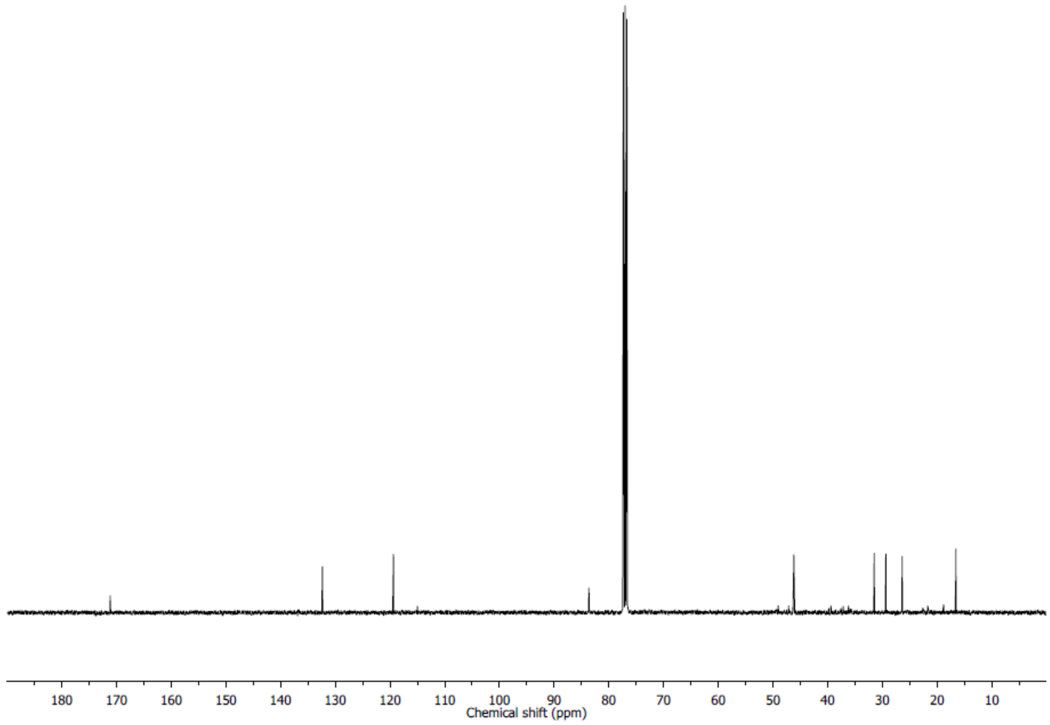

**(*R,E*)-6-Cinnamyl-6-methyltetrahydro-2H-pyran-2-one (4b)**

400 MHz, $CDCl_3$

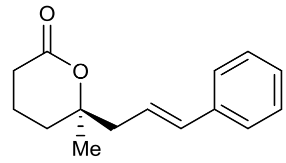


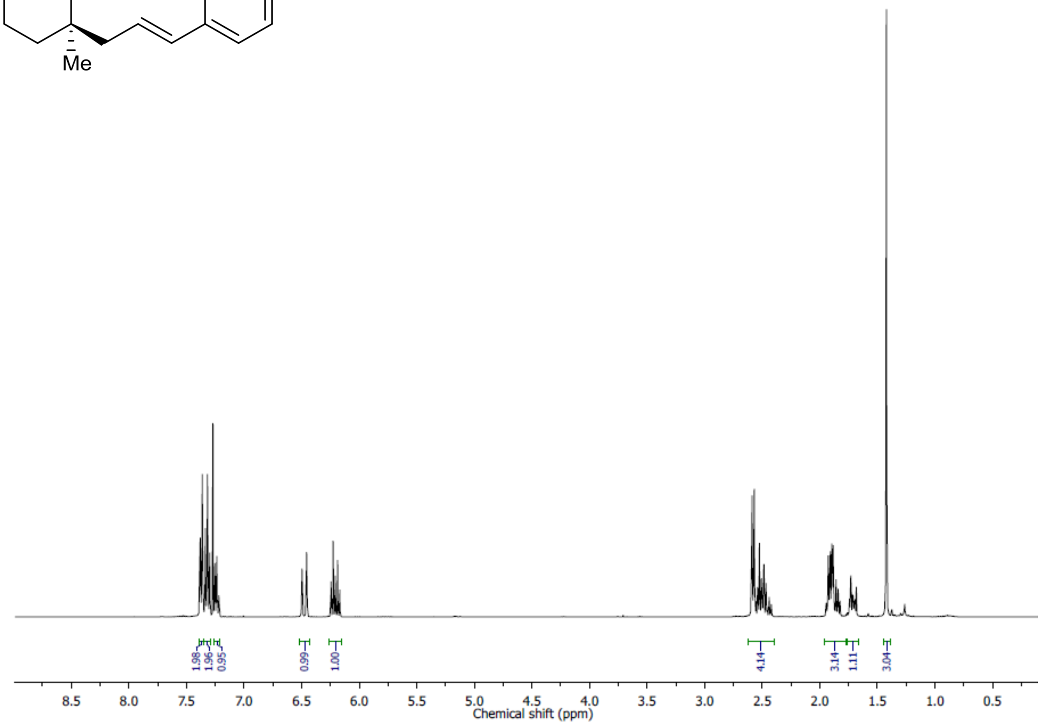


101 MHz, $CDCl_3$

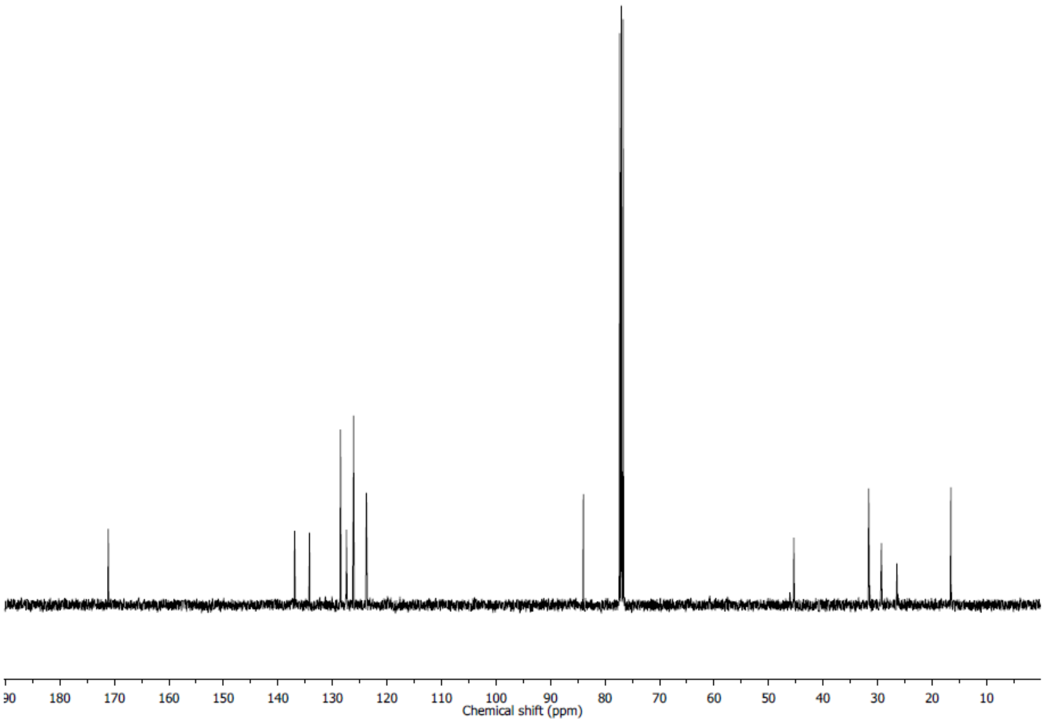

**(*R,E*)-6-(3-(4-Fluorophenyl)allyl)-6-methyltetrahydro-2H-pyran-2-one (4c)**

400 MHz, $CDCl_3$

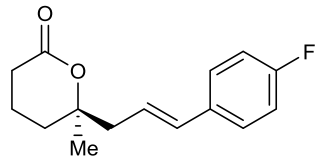


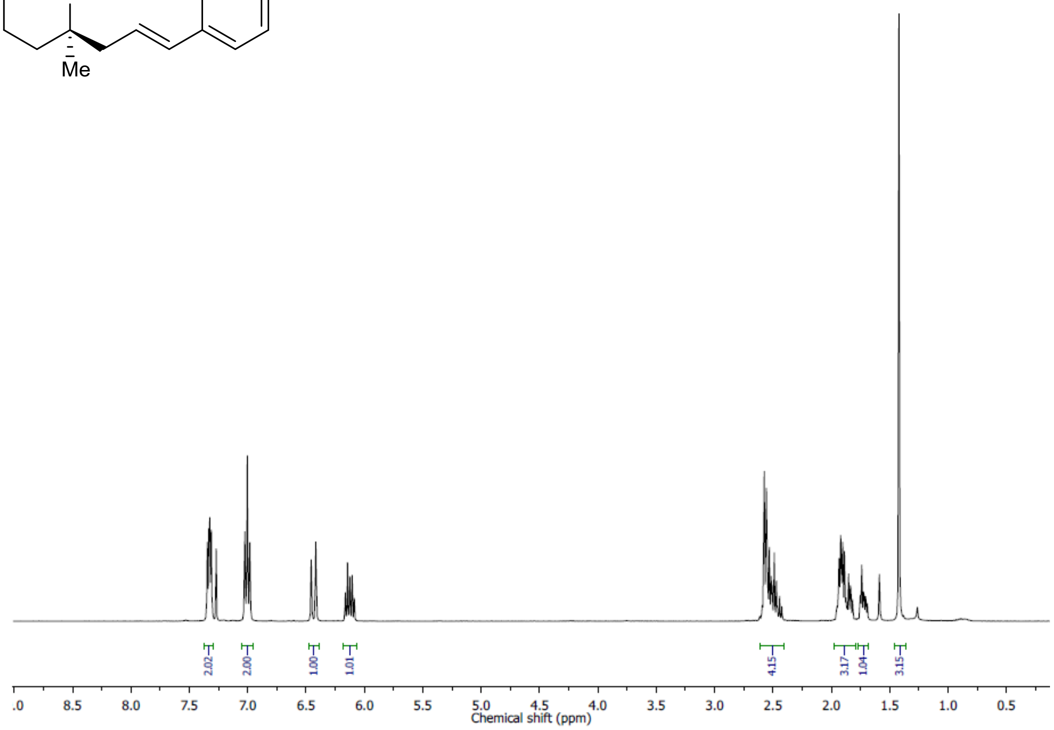


101 MHz, $CDCl_3$

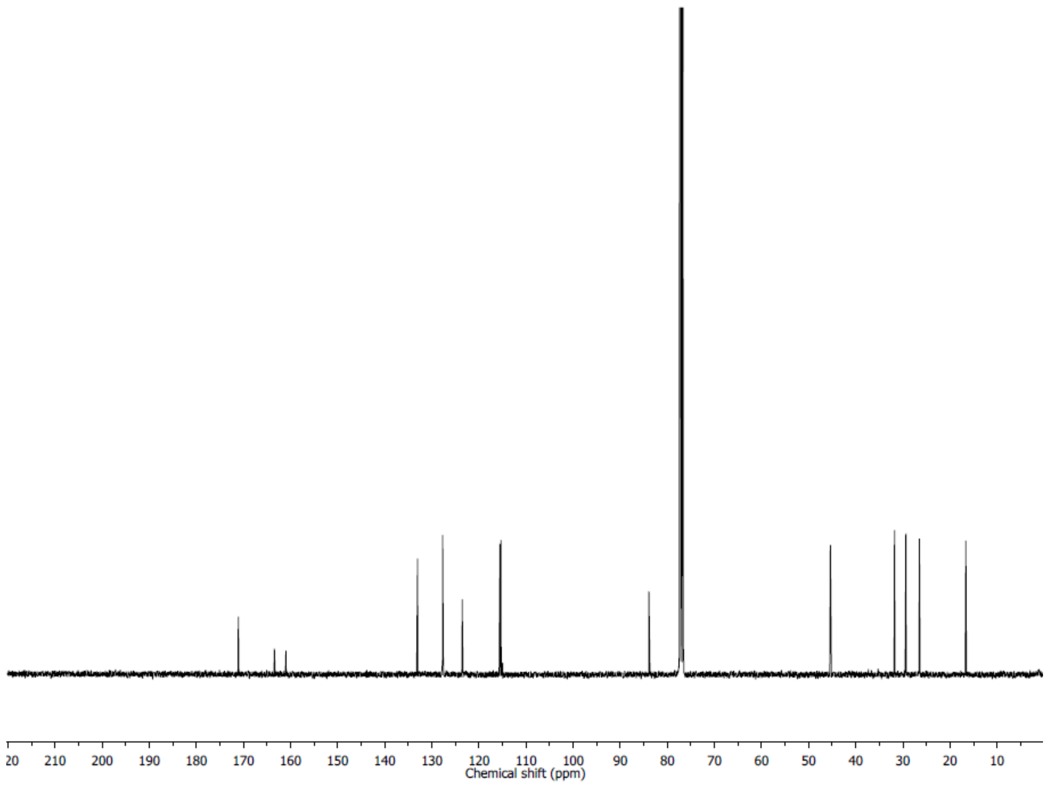

**(*E*)-6-Methyl-6-(3-(naphthalen-2-yl)allyl)tetrahydro-2H-pyran-2-one (4d)**

400 MHz, $CDCl_3$

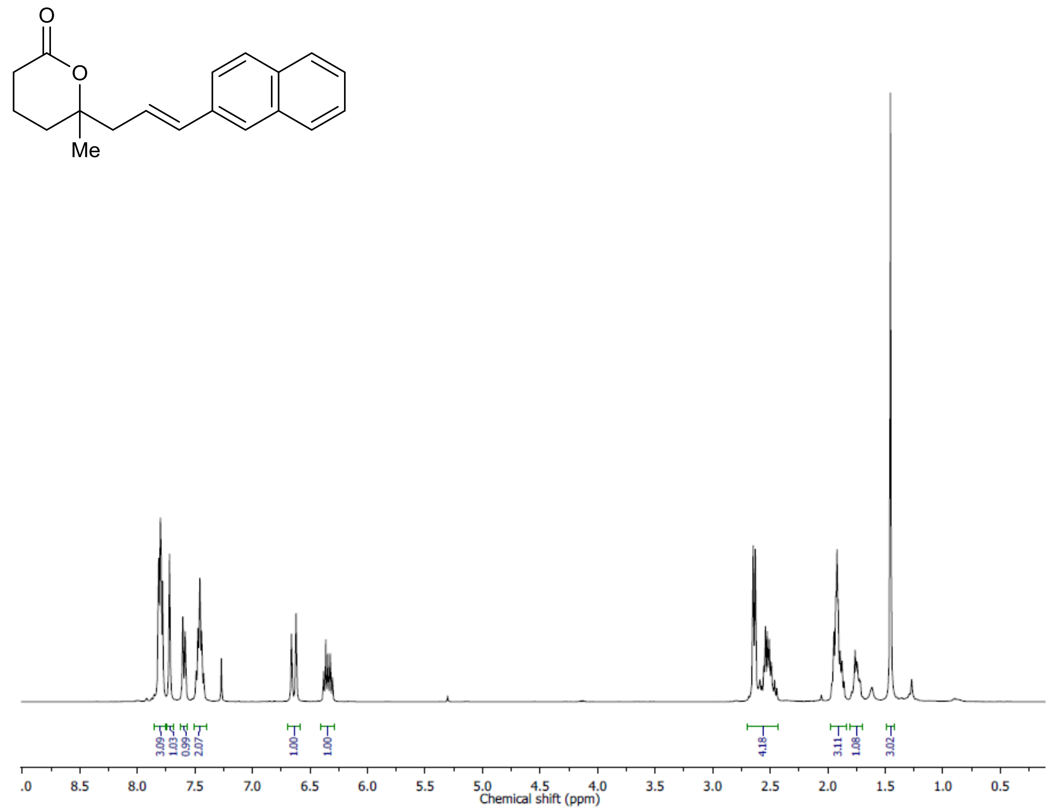


101 MHz, $CDCl_3$

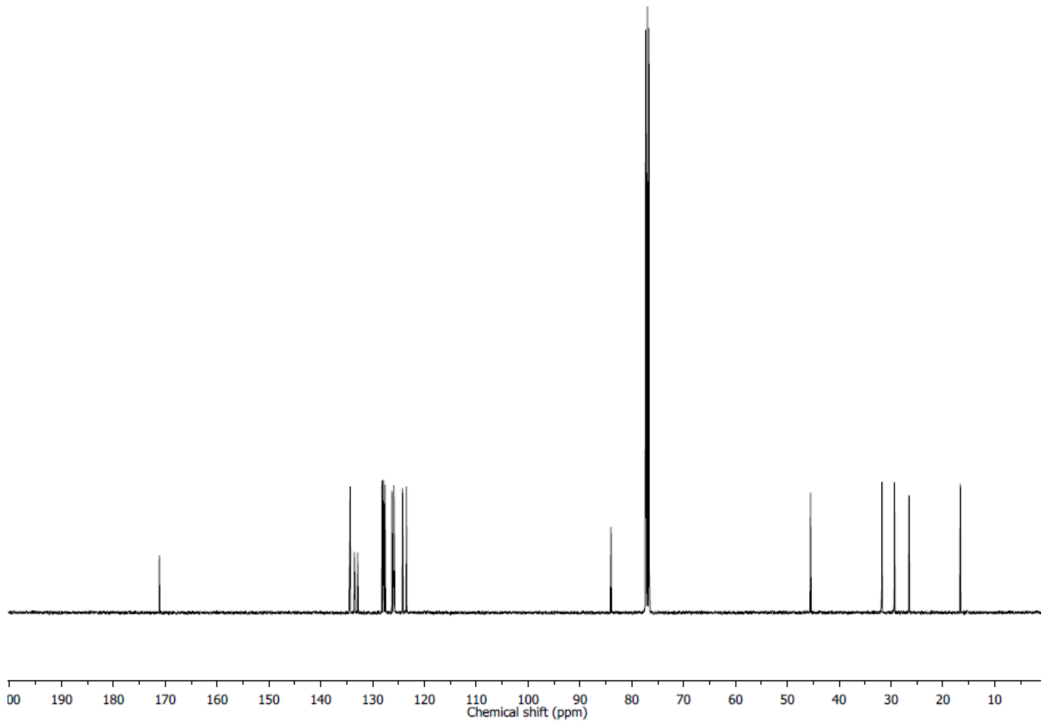

**(*E*)-6-(3-(3-Bromophenyl)allyl)-6-methyltetrahydro-2H-pyran-2-one (4e)**

400 MHz, $CDCl_3$

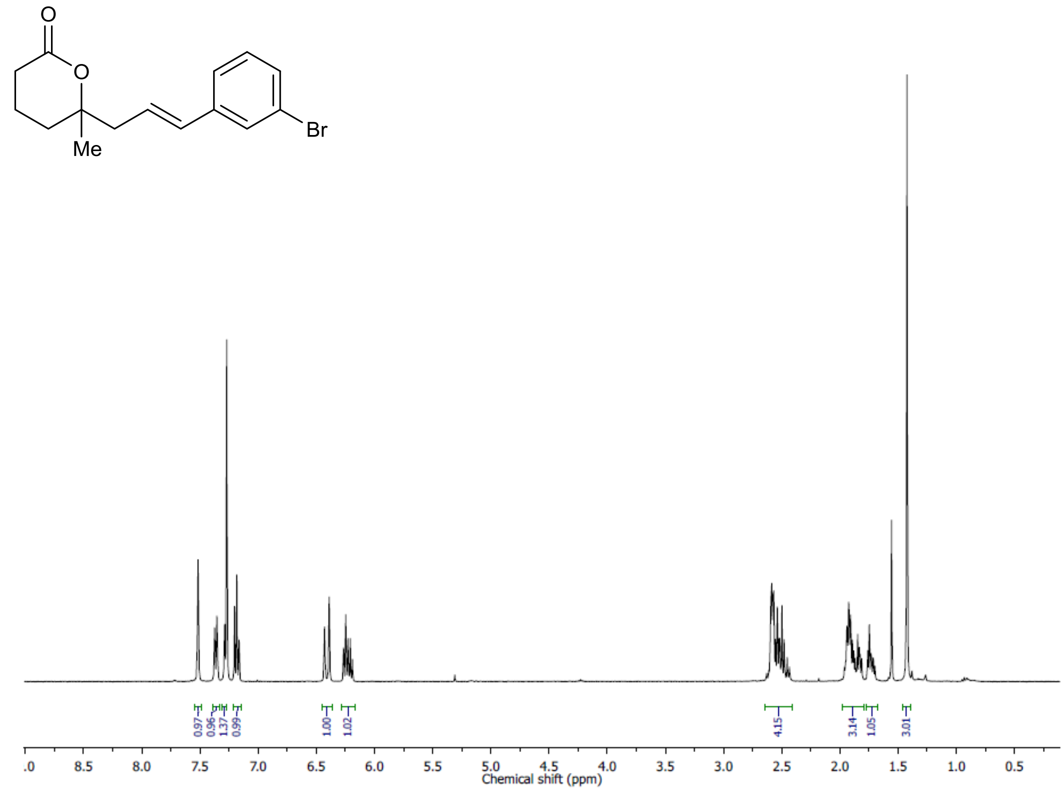


101 MHz, $CDCl_3$

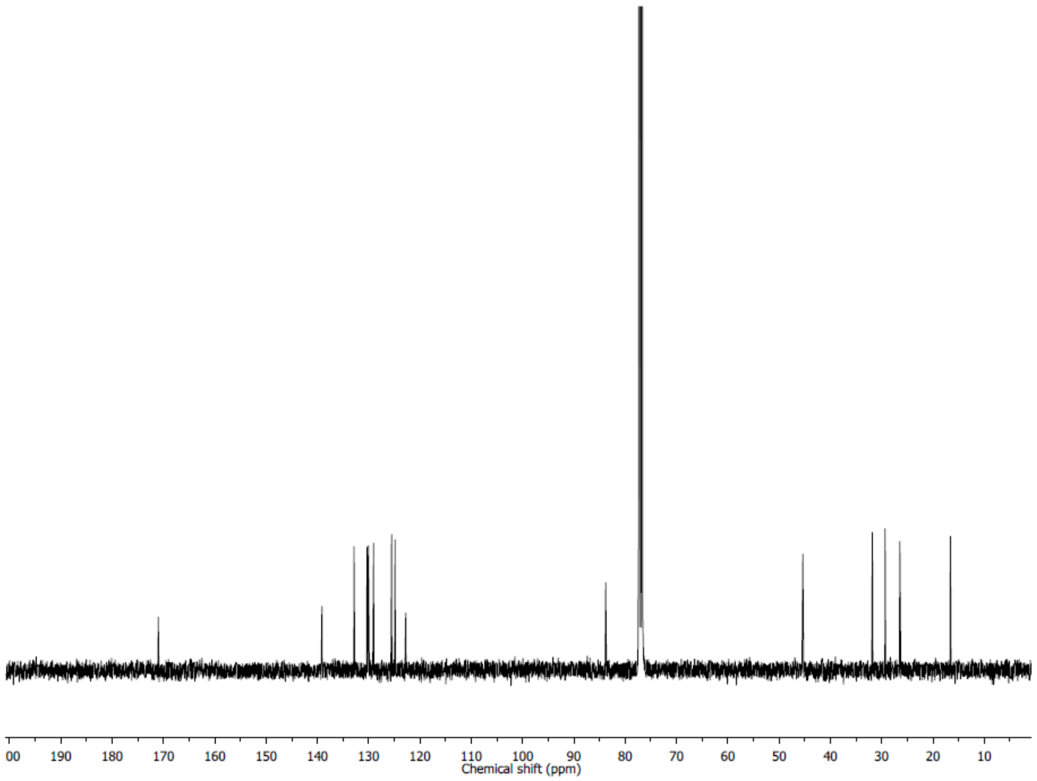

**(*R,E*)-6-(3-(2-Chlorophenyl)allyl)-6-methyltetrahydro-2H-pyran-2-one (4f)**

400 MHz, $CDCl_3$

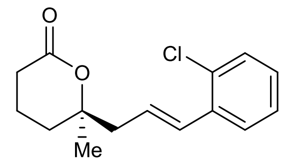


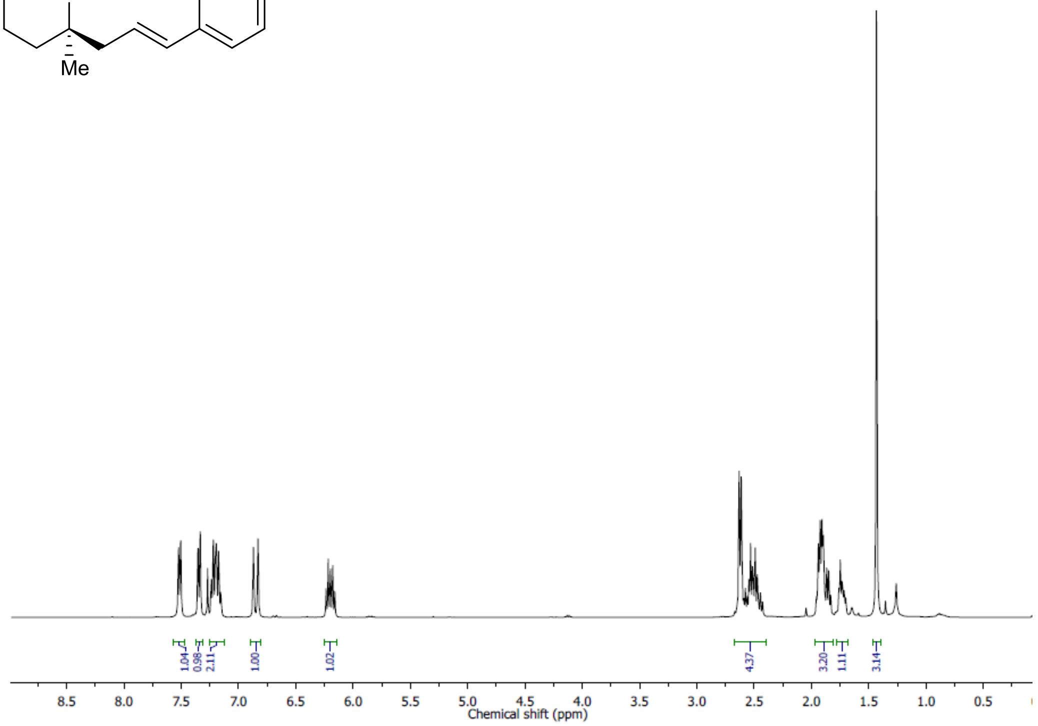


101 MHz, $CDCl_3$

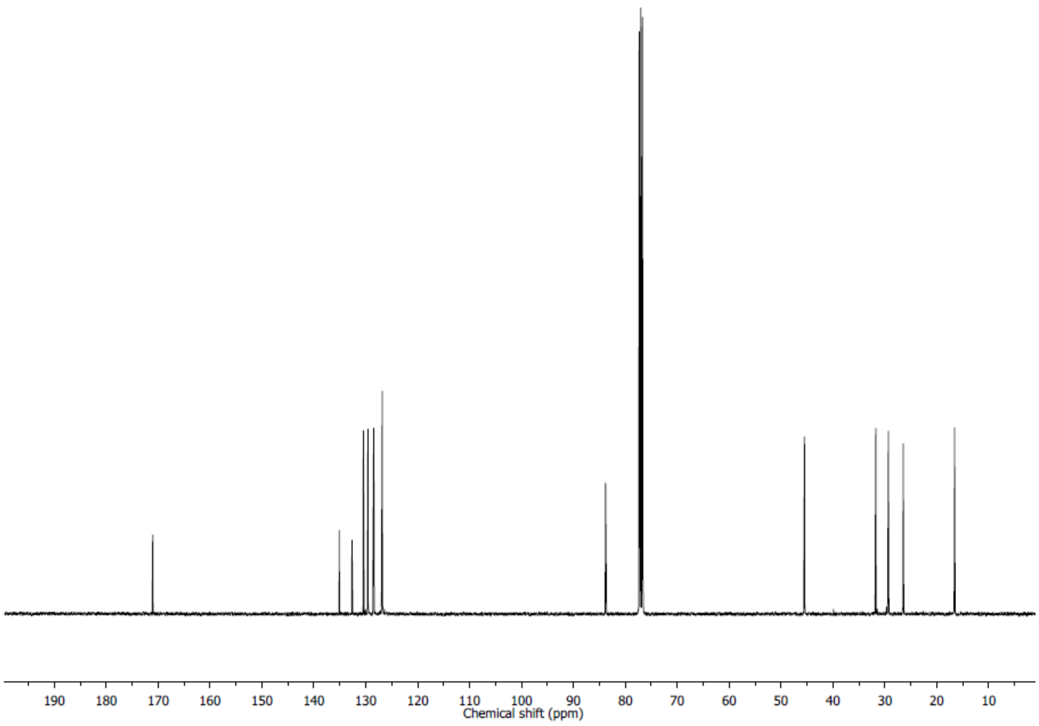

**(*E*)-6-Methyl-6-(3-(4-(trifluoromethyl)phenyl)allyl)tetrahydro-2H-pyran-2-one (4g)**

400 MHz, $CDCl_3$

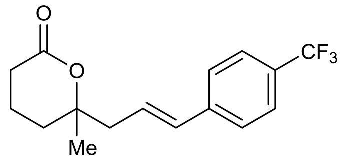


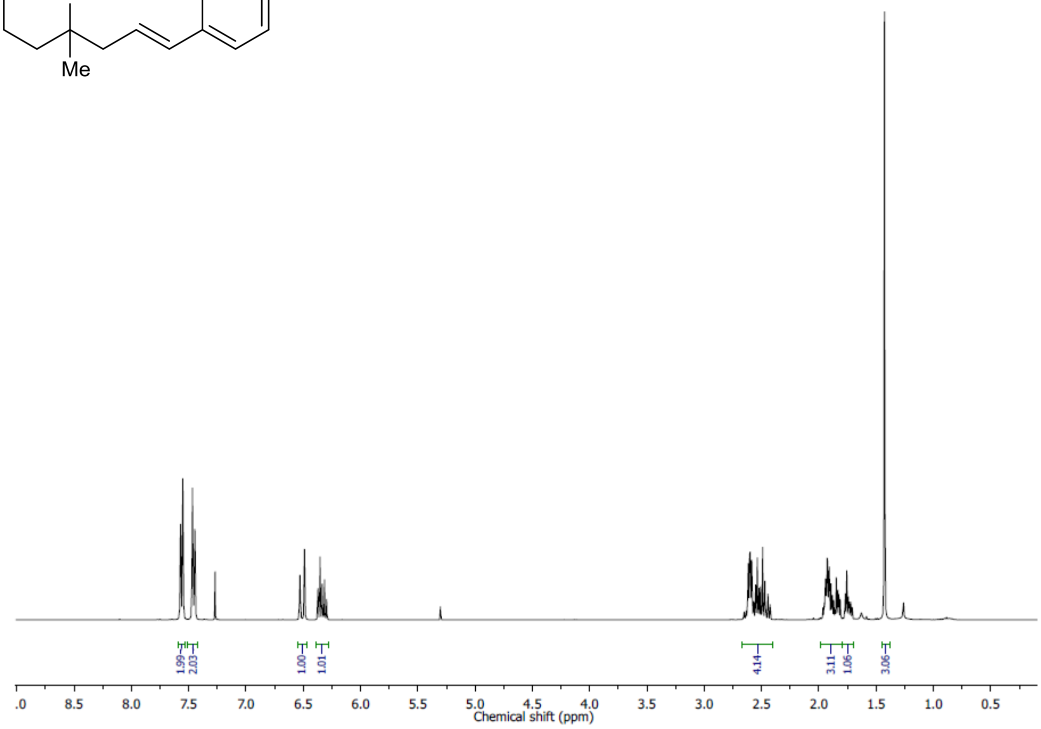


101 MHz, $CDCl_3$

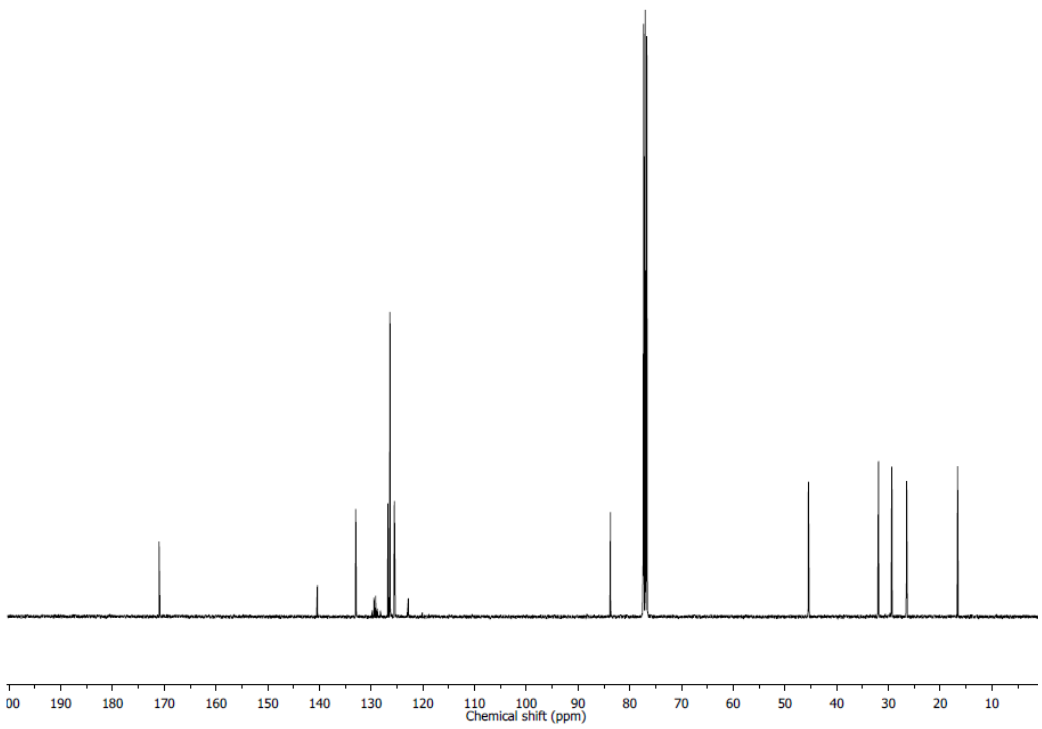

**(*R,E*)-6-Methyl-6-(3-(o-tolyl)allyl)tetrahydro-2H-pyran-2-one (4h)**

400 MHz, $CDCl_3$

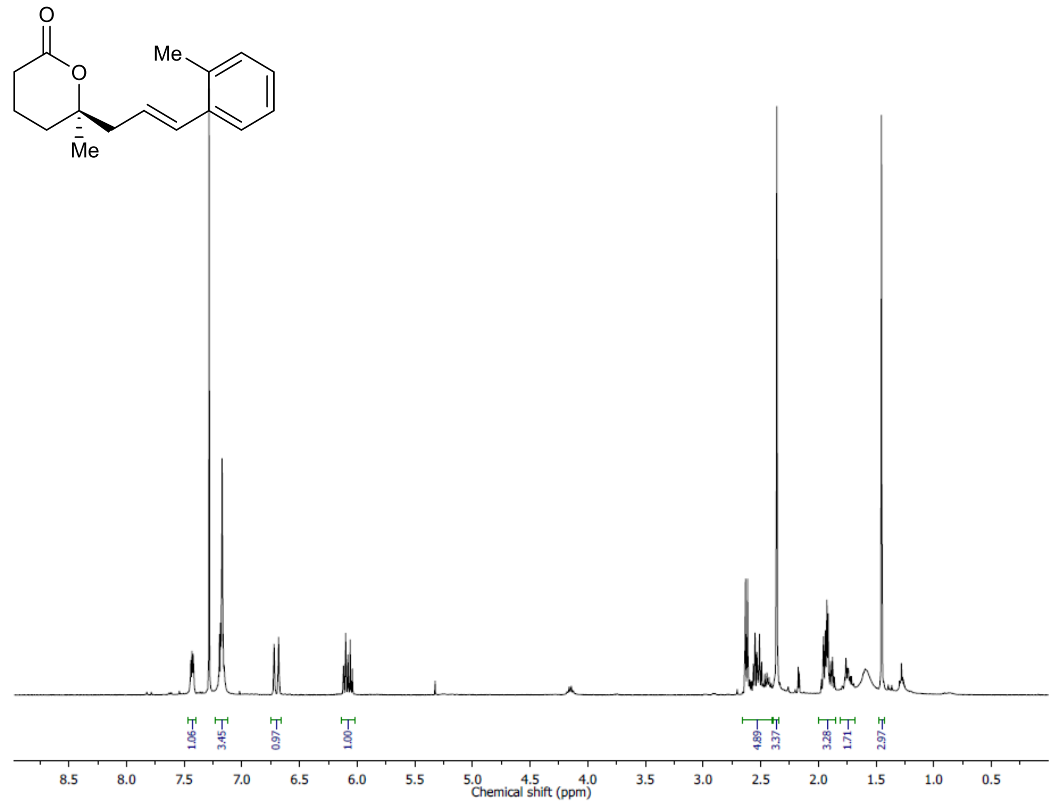


101 MHz, $CDCl_3$

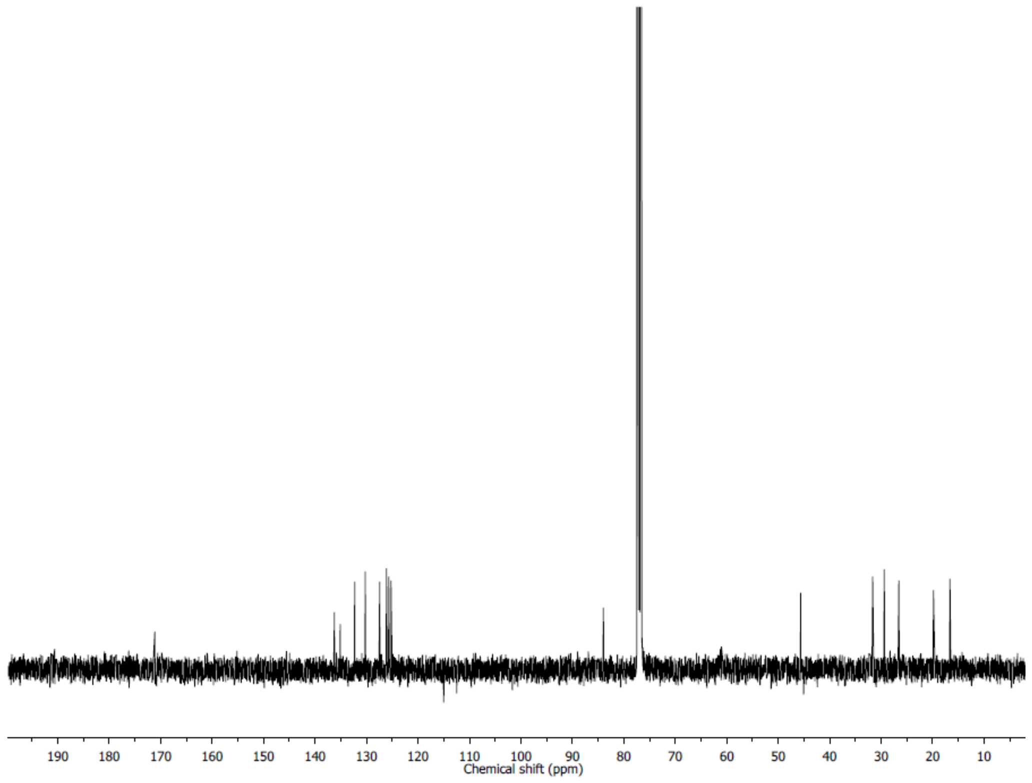

**(1*S*,6*R*,8*S*)-8-Benzyl-6-methyl-9-oxabicyclo[4.2.1]nonan-1-ol (6a)**

400 MHz, $CDCl_3$

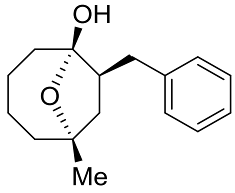


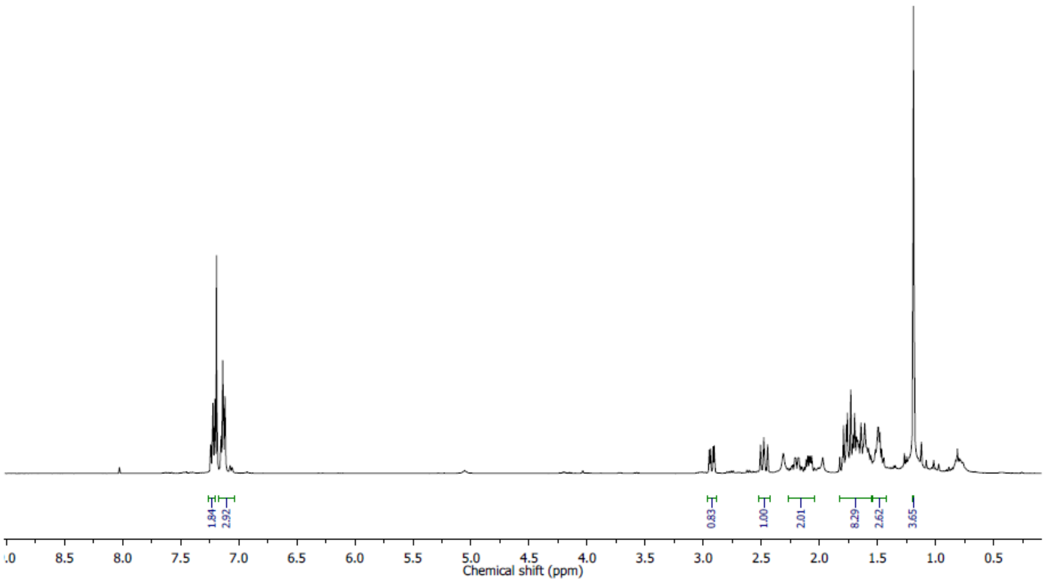


101 MHz, $CDCl_3$

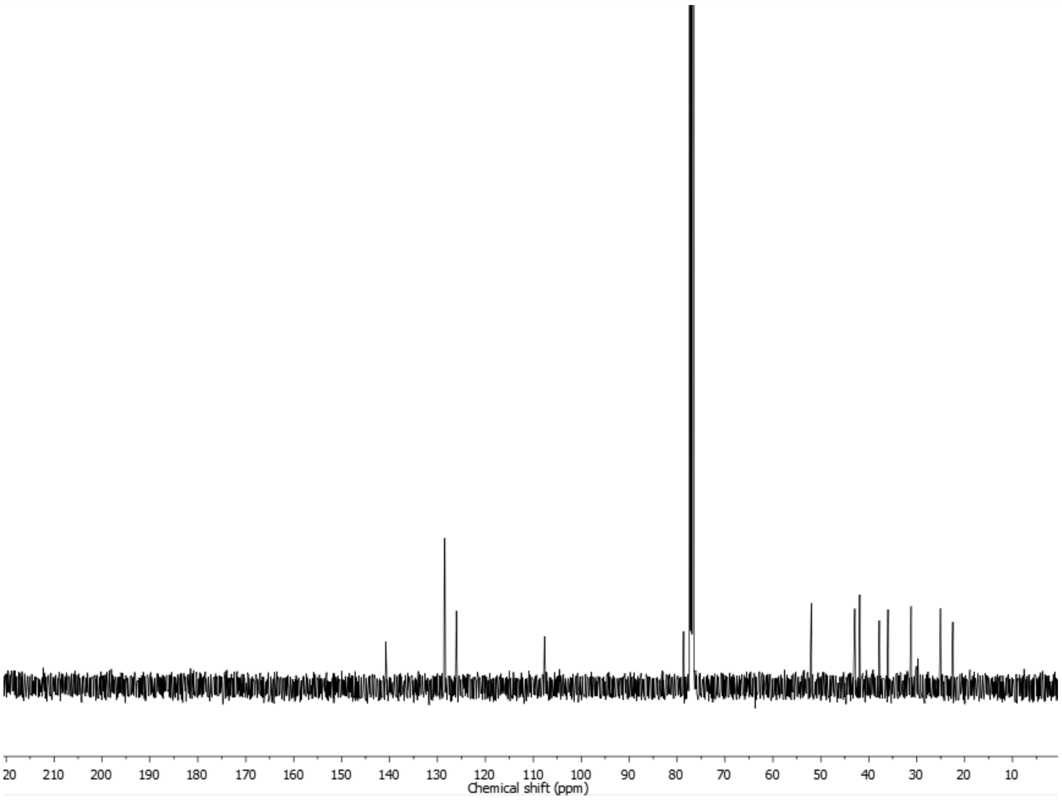

**(1*S*,6*R*,8*S*)-8-(2-Chlorobenzyl)-6-methyl-9-oxabicyclo[4.2.1]nonan-1-ol (6b)**

400 MHz, $CDCl_3$

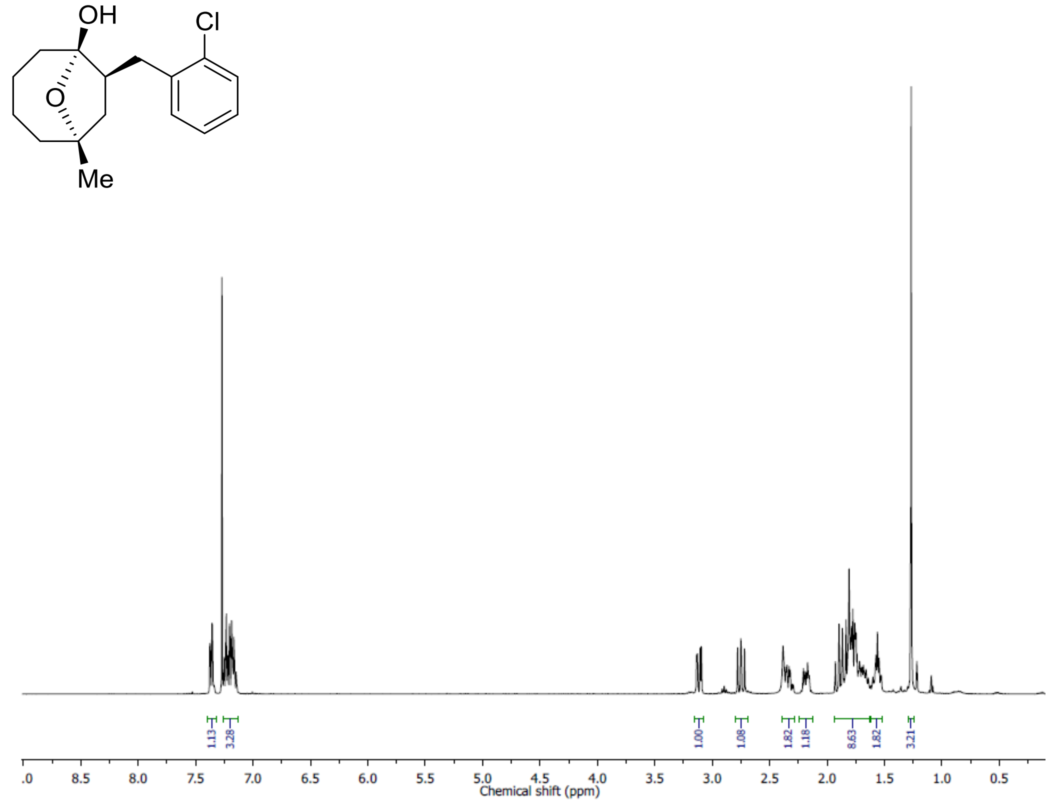


101 MHz, $CDCl_3$

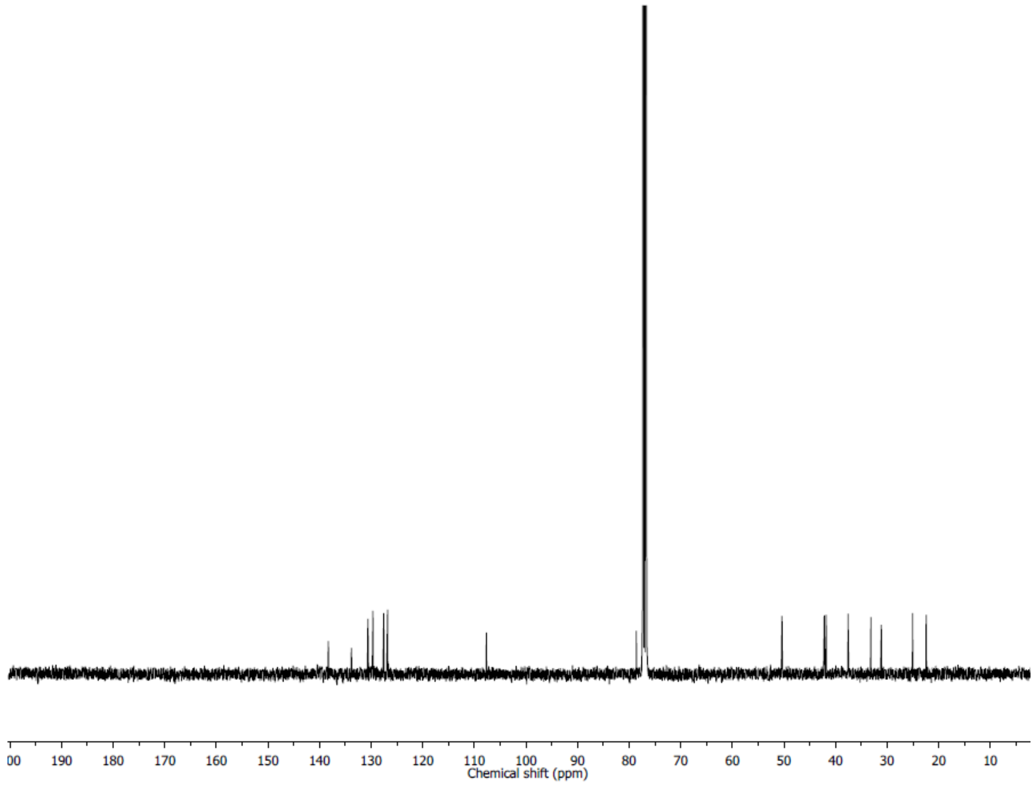

**(1*S*,6*R*,8*S*)-8-(4-Fluorobenzyl)-6-methyl-9-oxabicyclo[4.2.1]nonan-1-ol (6c)**

400 MHz, $CDCl_3$

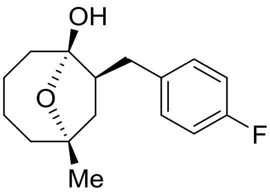


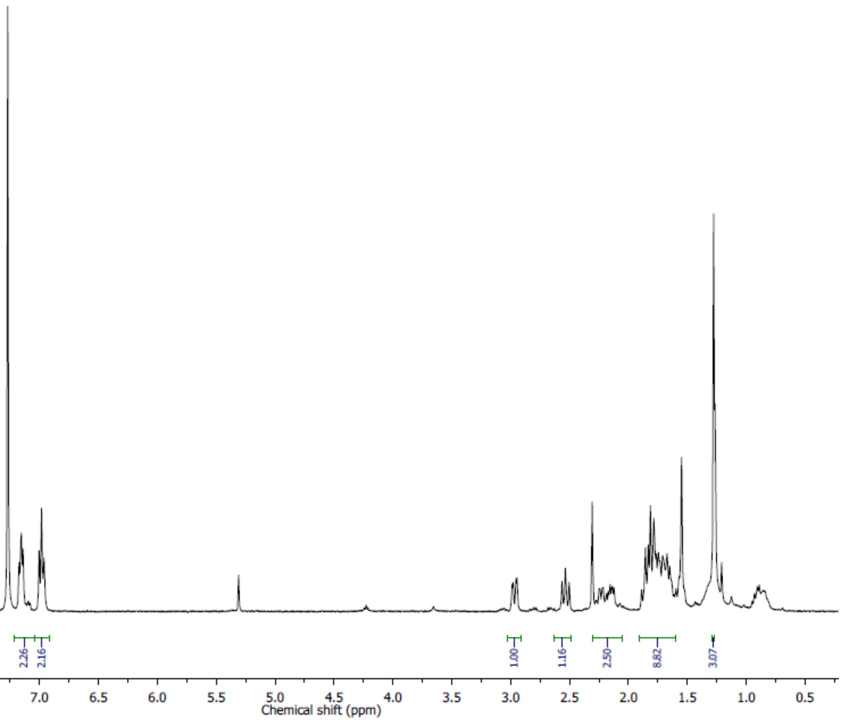


101 MHz, $CDCl_3$

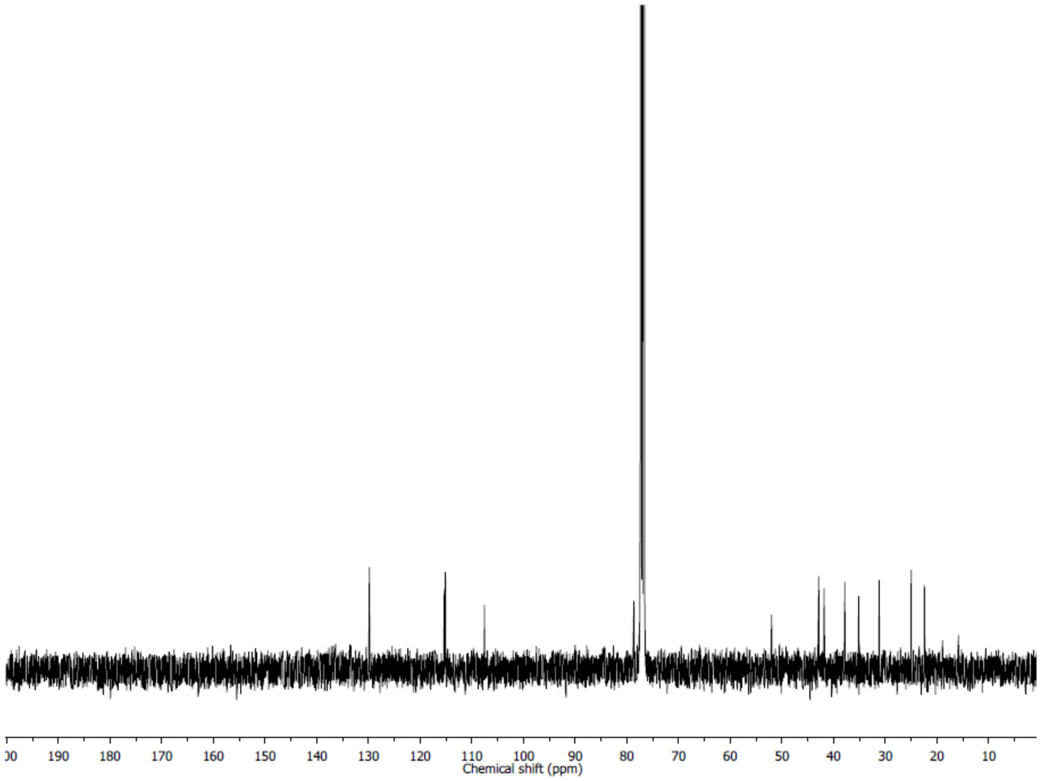

**(1*S*,6*R*,8*S*)-6-Methyl-8-(4-(trifluoromethyl)benzyl)-9-oxabicyclo[4.2.1]nonan-1-ol (6d)**

400 MHz, $CDCl_3$

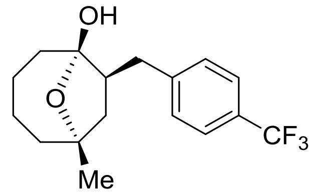


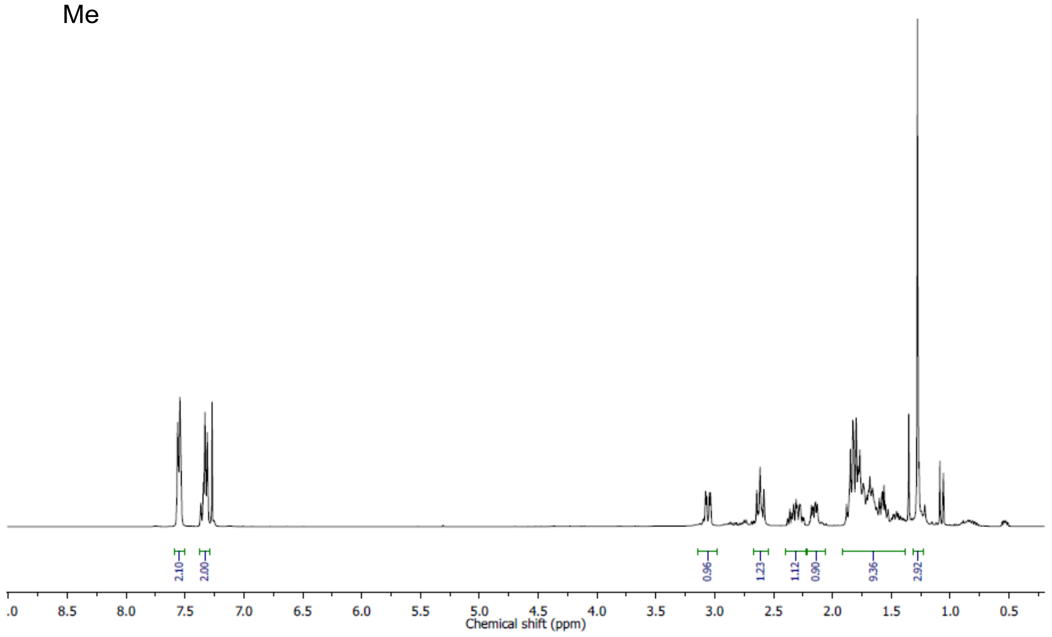


101 MHz, $CDCl_3$

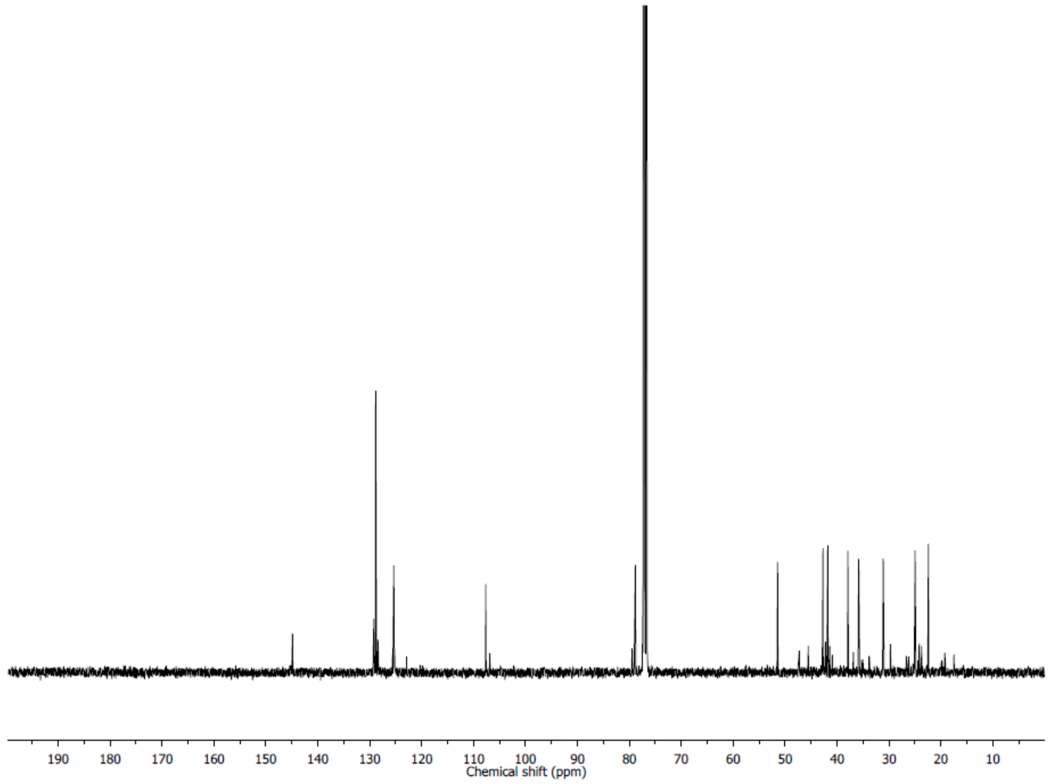

**(1*S*,5*R*,7*S*)-7-Benzyl-5-methyl-8-oxabicyclo[3.2.1]octan-1-ol (6e)**

400 MHz, $CDCl_3$

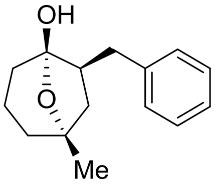


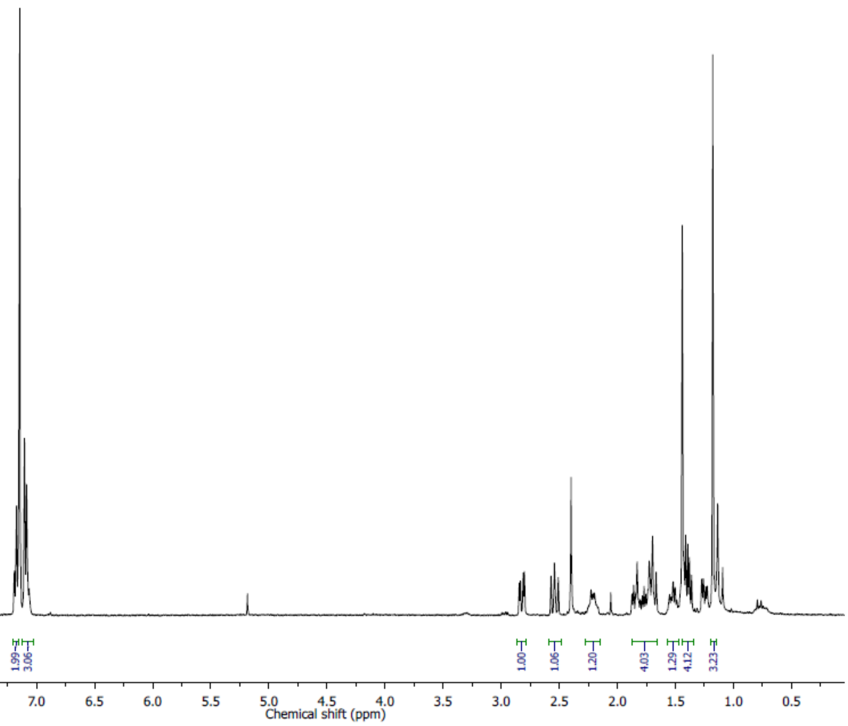


101 MHz, $CDCl_3$

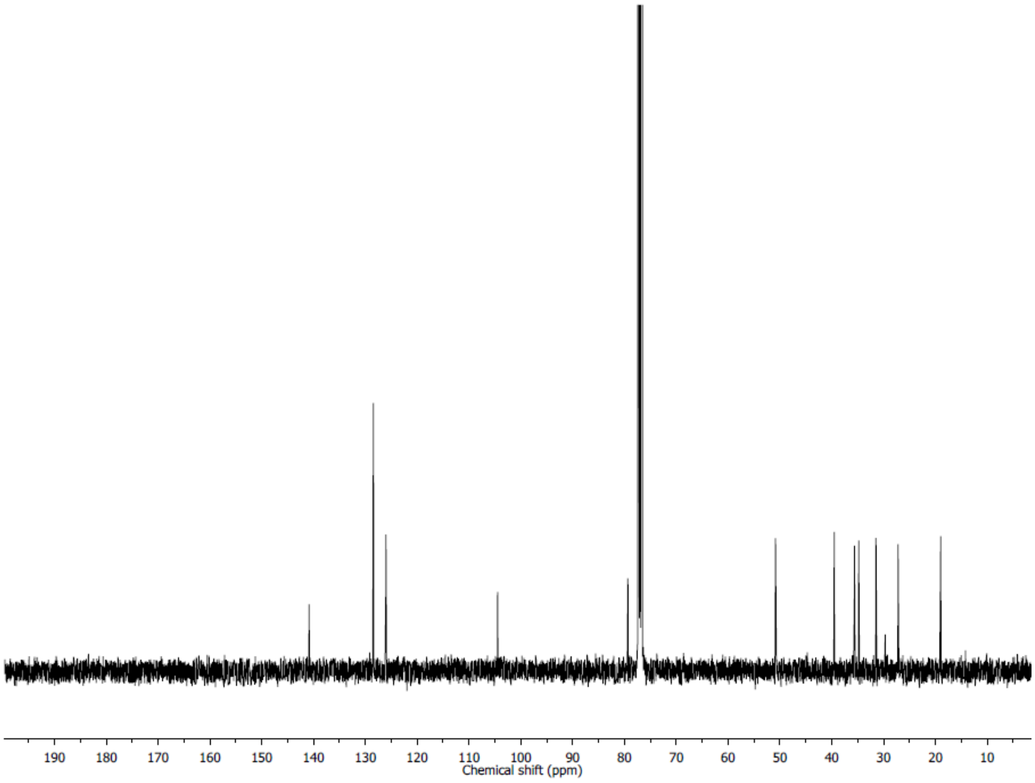

**(1*S*,6*S*,8*R*)-8-Benzyl-6-methylbicyclo[4.2.0]octan-1-ol (7a)**

500 MHz, $CDCl_3$

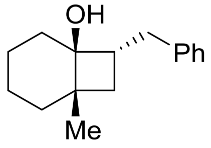


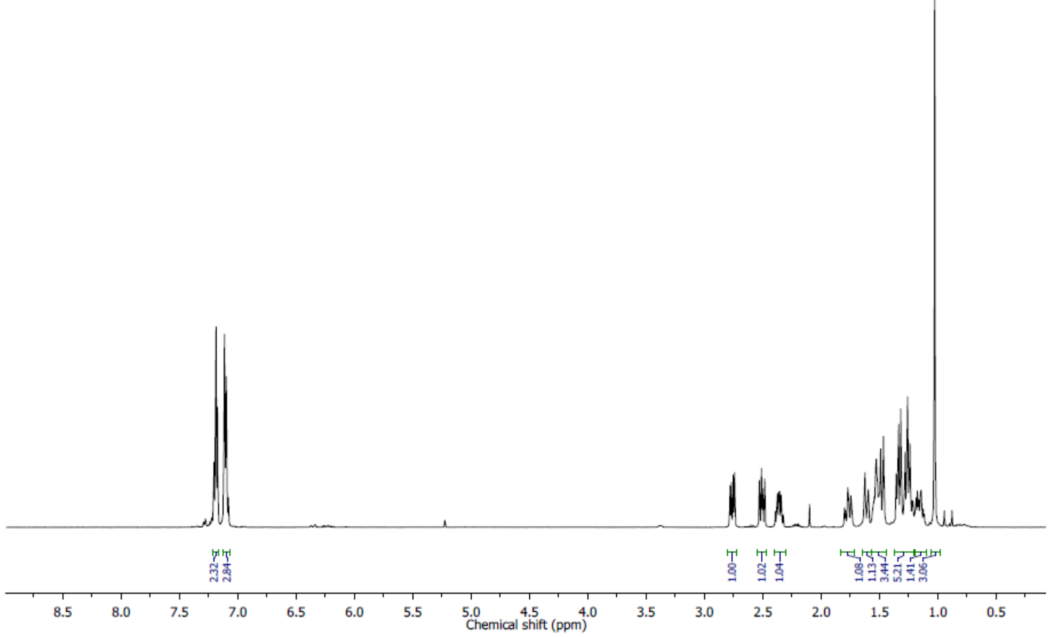


126 MHz, $CDCl_3$

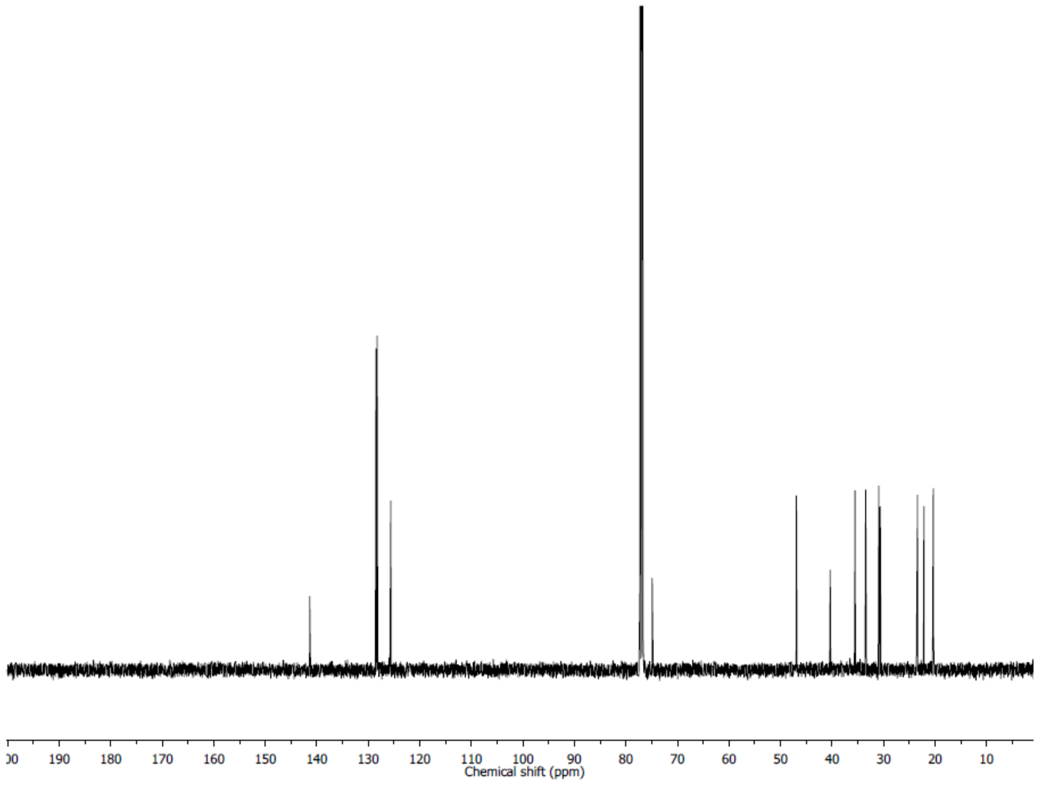

**(1*S*,6*S*,8*S*)-8-(2-Chlorobenzyl)-6-methylbicyclo[4.2.0]octan-1-ol (7b)**

500 MHz, $CDCl_3$

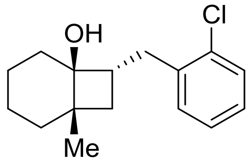


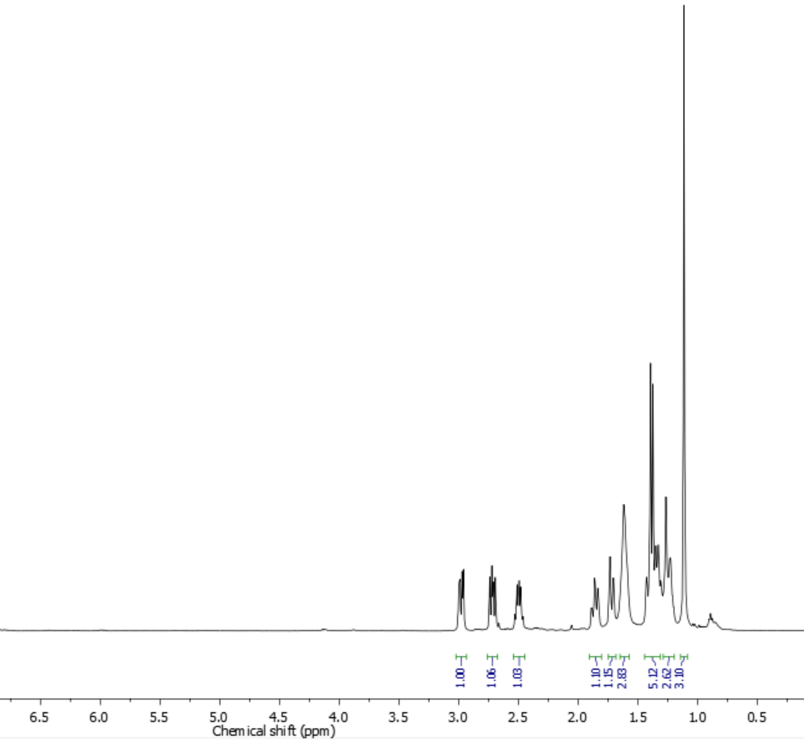


126 MHz, $CDCl_3$

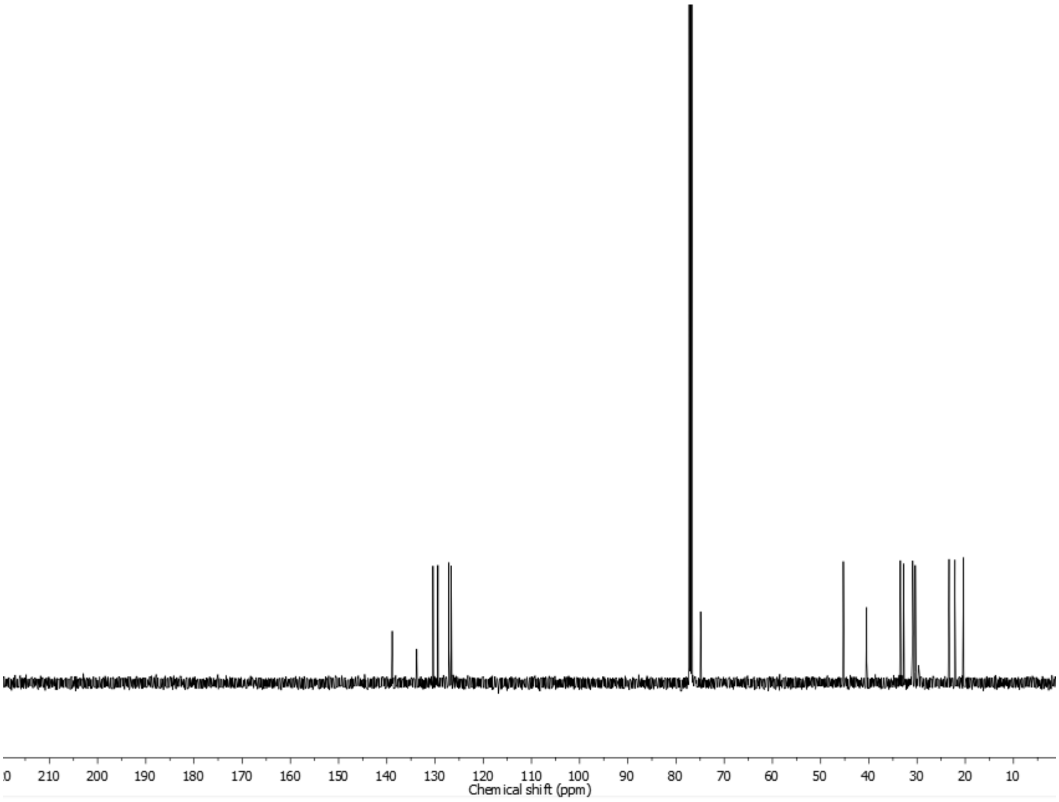

**(1*S*,5*S*,7*R*)-7-Benzyl-5-methylbicyclo[3.2.0]heptan-1-ol (7c)**

400 MHz, $CDCl_3$

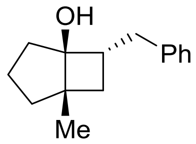


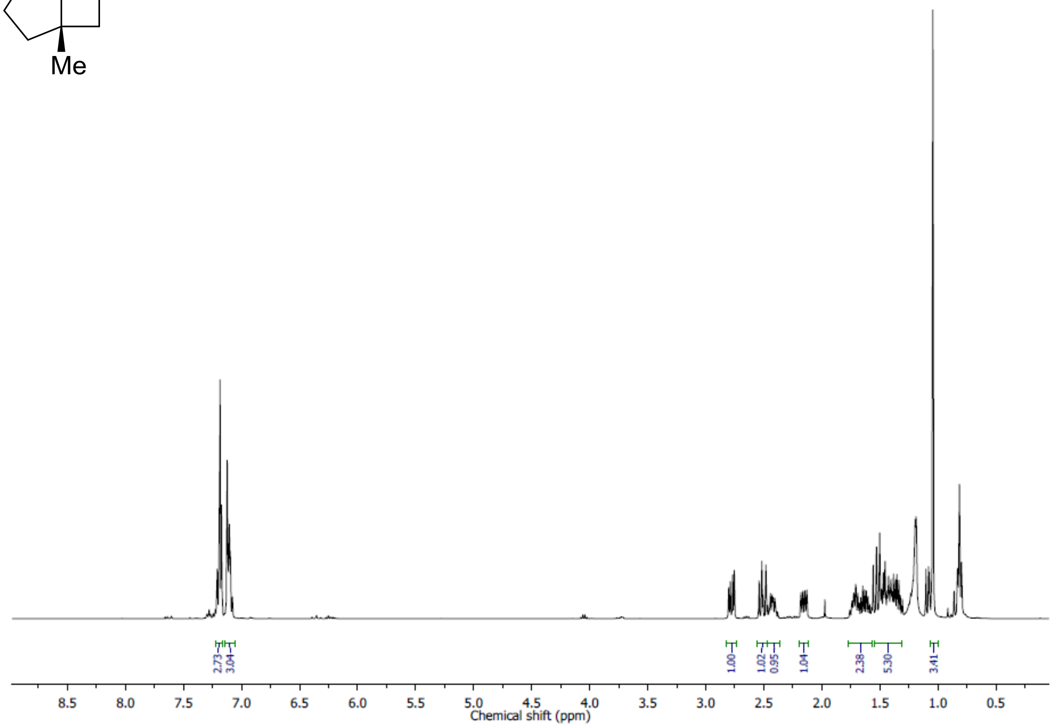


101 MHz, $CDCl_3$

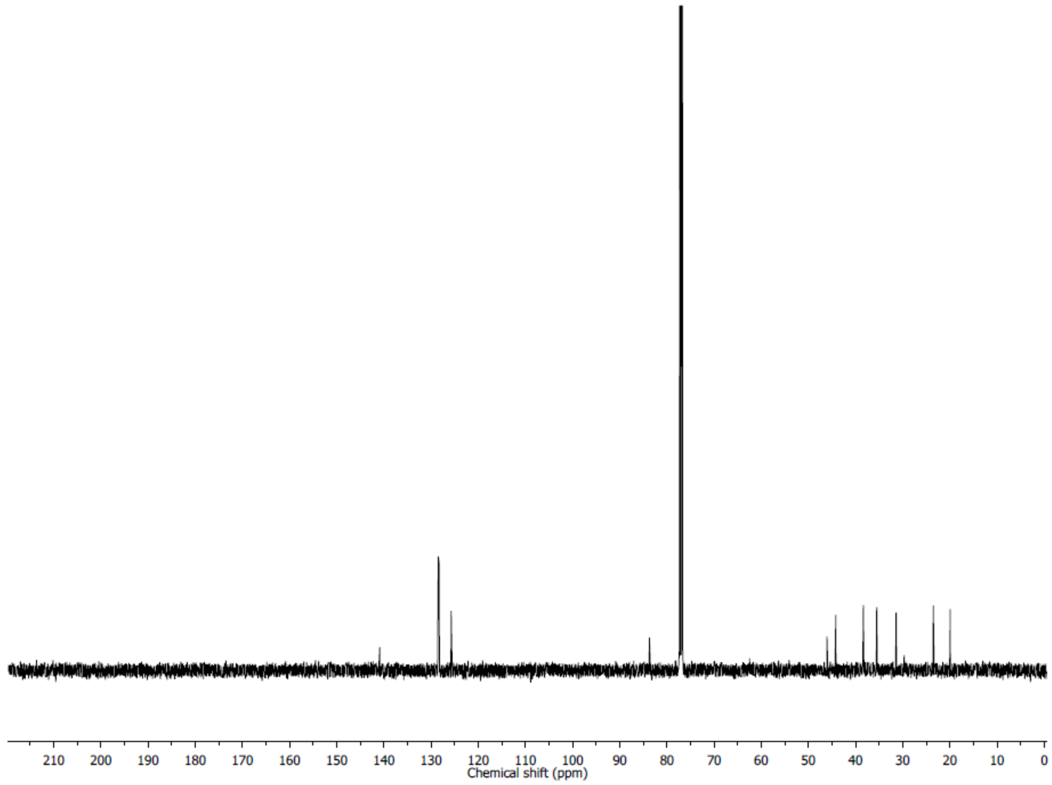

## 7. References


[1] L. A. Kelley, S. Mezulis, C. M. Yates, M. N. Wass, M. J. E. Sternberg, *Nat. Protoc.* **2015**, *10*, 845-858.

[2] B. J. Yachnin, T. Sprules, M. B. McEvoy, P. C. K. Lau, A. M. Berghuis, *J. Am. Chem. Soc.* **2012**, *134*, 7788-7795.

[3] O. Trott, A. J. Olson, *J. Comput. Chem.* **2010**, *31*, 455-461.

[4] F. Long, R. A. Nicholls, P. Emsley, S. Gražulis, A. Merkys, A. Vaitkus, G. N. Murshudov, *Acta. Crystallogr. D.* **2017**, *73*, 112-122.

[5] M. Nishizawa, A. Yadav, Y. Iwamoto, H. Imagawa, *Tetrahedron* **2004**, *60*, 9223-9234.

[6] J. T. Mohr, M. K. Krout, B. M. Stoltz, *Org. Synth.* **2009**, *86*, 194-211.

[7] Z. Xu, Q. Wang, J. Zhu, *J. Am. Chem. Soc.* **2015**, *137*, 6712-6724.

[8] R. A. Craig, S. A. Loskot, J. T. Mohr, D. C. Behenna, A. M. Harned, B. M. Stoltz, *Org. Lett.* **2015**, *17*, 5160-5163.

[9] X. Just-Baringo, J. Clark, M. J. Gutmann, D. J. Procter, *Angew. Chem. Int. Ed.* **2016**, *55*, 12499-12502.

[10] D. C. Behenna, J. T. Mohr, N. H. Sherden, S. C. Marinescu, A. M. Harned, K. Tani, M. Seto, S. Ma, Z. Novák, M. R. Krout, R. M. McFadden, J. L. Roizen, J. A. Enquist, D. E. White, S. R. Levine, K. V. Petrova, A. Iwashita, S. C. Virgil, B. M. Stoltz, *Chem. Eur. J.* **2011**, *17*, 14199-14223.

[11] D. Parmar, K. Price, M. Spain, H. Matsubara, P. A. Bradley, D. J. Procter, *J. Am. Chem. Soc.* **2011**, *133*, 2418-2420.

[12] A. N. Marziale, D. C. Duquette, R. A. Craig, K. E. Kim, M. Liniger, Y. Numajiri, B. M. Stoltz, *Adv. Synth. Catal.* **2015**, *357*, 2238-2245.

[1] L. A. Kelley, S. Mezulis, C. M. Yates, M. N. Wass, M. J. E. Sternberg, *Nat. Protocols* **2015**, *10*, 845-858.

[2] B. J. Yachnin, T. Sprules, M. B. McEvoy, P. C. K. Lau, A. M. Berghuis, *Journal of the American Chemical Society* **2012**, *134*, 7788-7795.

[3] O. Trott, A. J. Olson, *J. Comput. Chem.* **2010**, *31*, 455-461.

[4] F. Long, R. A. Nicholls, P. Emsley, S. Gražulis, A. Merkys, A. Vaitkus, G. N. Murshudov, *Acta Crystallographica. Section D, Structural Biology* **2017**, *73*, 112-122.

[5] M. Nishizawa, A. Yadav, Y. Iwamoto, H. Imagawa, *Tetrahedron* **2004**, *60*, 9223-9234.

[6] J. Mohr, *Organic syntheses* **2009**, *86*, 194.

[7] Z. Xu, Q. Wang, J. Zhu, *Journal of the American Chemical Society* **2015**, *137*, 6712-6724.

[8] R. A. Craig, S. A. Loskot, J. T. Mohr, D. C. Behenna, A. M. Harned, B. M. Stoltz, *Org. Lett.* **2015**, *17*, 5160-5163.

[9] X. Just-Baringo, J. Clark, M. J. Gutmann, D. J. Procter, *Angew. Chem. Int. Ed.* **2016**, *55*, 12499-12502.

[10] D. C. Behenna, J. T. Mohr, N. H. Sherden, S. C. Marinescu, A. M. Harned, K. Tani, M. Seto, S. Ma, Z. Novák, M. R. Krout, R. M. McFadden, J. L. Roizen, J. A. Enquist, D. E. White, S. R. Levine, K. V. Petrova, A. Iwashita, S. C. Virgil, B. M. Stoltz, *Chemistry – A European Journal* **2011**, *17*, 14199-14223.

[11] D. Parmar, K. Price, M. Spain, H. Matsubara, P. A. Bradley, D. J. Procter, *Journal of the American Chemical Society* **2011**, *133*, 2418-2420.

[12] A. N. Marziale, D. C. Duquette, R. A. Craig, K. E. Kim, M. Liniger, Y. Numajiri, B. M. Stoltz, *Adv. Synth. Catal.* **2015**, *357*, 2238-2245.
